# Improved HDAC Inhibition, Stronger Cytotoxic Effect and Higher Selectivity against Leukemias and Lymphomas of Novel, Tricyclic Vorinostat Analogues

**DOI:** 10.3390/ph14090851

**Published:** 2021-08-26

**Authors:** Bartosz Bieszczad, Damian Garbicz, Marta Świtalska, Marta K. Dudek, Dawid Warszycki, Joanna Wietrzyk, Elżbieta Grzesiuk, Adam Mieczkowski

**Affiliations:** 1 Institute of Biochemistry and Biophysics, Polish Academy of Sciences, 02-106 Warsaw, Poland; b.bieszczad@ibb.waw.pl (B.B.); dgarbicz@ibb.waw.pl (D.G.); 2 Hirszfeld Institute of Immunology and Experimental Therapy, Polish Academy of Sciences, 53-114 Wrocław, Poland; marta.switalska@hirszfeld.pl (M.Ś.); joanna.wietrzyk@hirszfeld.pl (J.W.); 3 Centre of Molecular and Macromolecular Studies, Polish Academy of Sciences, Sienkiewicza 112, 90-363 Lodz, Poland; mdudek@cbmm.lodz.pl; 4 Maj Institute of Pharmacology, Polish Academy of Sciences, 31-343 Cracow, Poland; warszyc@if-pan.krakow.pl

**Keywords:** Vorinostat, histone deacetylase, HDAC inhibitors, dibenzodiazocines, hydroxamic acid, selectivity

## Abstract

Histone deacetylase (HDAC) inhibitors are a class of drugs used in the cancer treatment. Here, we developed a library of 19 analogues of Vorinostat, an HDAC inhibitor used in lymphomas treatment. In Vorinostat, we replaced the hydrophobic phenyl group with various tricyclic ‘caps’ possessing a central, eight-membered, heterocyclic ring, and investigated the HDAC activity and cytotoxic effect on the cancer and normal cell lines. We found that 3 out of the 19 compounds, based on dibenzo[*b,f*]azocin-6(5*H*)-one, 11,12-dihydrodibenzo[*b,f*]azocin-6(5*H*)-one, and benzo[*b*]naphtho[2,3-*f*][1,5]diazocine-6,14(5*H*,13*H*)-dione scaffolds, showed better HDACs inhibition than the referenced Vorinostat. In leukemic cell line MV4-11 and in the lymphoma cell line Daudi, three compounds showed lower IC_50_ values than Vorinostat. These compounds had higher activity and selectivity against MV4-11 and Daudi cell lines than reference Vorinostat. We also observed a strong correlation between HDACs inhibition and the cytotoxic effect. Cell lines derived from solid tumours: A549 (lung carcinoma) and MCF-7 (breast adenocarcinoma) as well as reference BALB/3T3 (normal murine fibroblasts) were less susceptible to compounds tested. Developed derivatives show improved properties than Vorinostat, thus they could be considered as possible agents for leukemia and lymphoma treatment.

## 1. Introduction

Histone deacetylases (HDAC) are an important group of enzymes playing diverse biological roles in living cells [1,2,3,4]. Dysregulation of HDAC expression could be associated with various human malignancies [5,6,7]; thus, they focused the attention of medicinal chemists as potential molecular targets. To date, research efforts have been largely directed to the use of HDAC inhibitors as potential anti-cancer agents [8,9,10,11,12,13]. Nevertheless, other applications such as anti-inflammatory [14,15,16,17,18,19], antifibriotic [20,21,22,23,24], or neuroprotective effect in Huntington’s disease [25,26,27], Alzheimer disease [27,28], spinal muscular atrophy [29], or Friedreich’s ataxia were studied [30]. HDAC inhibitors were postulated as possible therapeutic agents in asthma and chronic obstructive pulmonary disease (COPD) [31], methamphetamine addiction [32], heart failure [33,34,35], diabetes [36,37], depression [38], or suppression of aging processes [39]. They were also tested for potential antimicrobial and anti-infective activities as antiviral [40,41,42], antibacterial [43], antifungal [44,45], or antiparasitic [46,47,48] agents. In anticancer therapy, the HDAC inhibitors were tested as therapeutic agents for different types of tumours including but not limited to glioblastoma [49], multiple myeloma [50,51,52], T-cell lymphoma [53], breast cancers [54], and lymphoproliferative disorders [55,56,57]. The anticancer effect of HDAC inhibitors could be further potentiated by development of dual mode, chimeric inhibitors [58] or by application of combined therapy together with other antitumour agents with a different mode of action such as epigallocatechin-3-gallate (EGCG), a DNA methyltransferase (DNMT) inhibitor [59], cisplatin, a metalating agent [60], gemcitabine interfering nucleic acid synthesis [61], decitabine, a hypomethylating agent inhibiting DNA methyltransferase [62], doxorubicin [63] and ellipticin [64] DNA intercalators and topoisomerase II inhibitors, Temozolomide, an alkylating agent [65], proteasome inhibitors [66], BET (bromodomain and extraterminal domain proteins) inhibitors [67], and RG7388, an inhibitor of tumour-associated protein MDM2 [68]. The antitumour effect of HDAC inhibitors was also combined with photodynamic therapy [69], radiation therapy (increasing radiation sensitivity) [70,71,72] and the application of oncolytic viruses [73,74].

Although several natural products were identified as HDAC inhibitors [75,76], most of them were obtained by chemical synthesis [77]. The first successful HDAC inhibitor bearing hydroxamic acid moiety, Vorinostat (SAHA, suberanilohydroxamic acid, Zolinza ^®^) (**1**) [78,79], was successfully used in the treatment of cutaneous T-cell lymphoma and its further analogues Belinostat (peripheral T-cell lymphoma), Panbinostat (multiple myeloma), and other types of HDAC inhibitors: Romidepsin (cutaneous T-cell lymphoma) and Chidamide (peripheral T-cell lymphoma) were approved by the FDA for cancer treatment [80]. However, it should be taken under consideration that HDAC inhibitors can cause a number of side effects [81] and their potential use and success in cancer therapy is highly dependent on difficulties to achieve selectivity, decrease toxicity, and reduce the adverse effects [82,83]. For this reason, new generations of HDAC inhibitors with an improved pharmacological profile and greater selectivity for cancer cells are intensively studied and developed [84].

**Figure 1:**
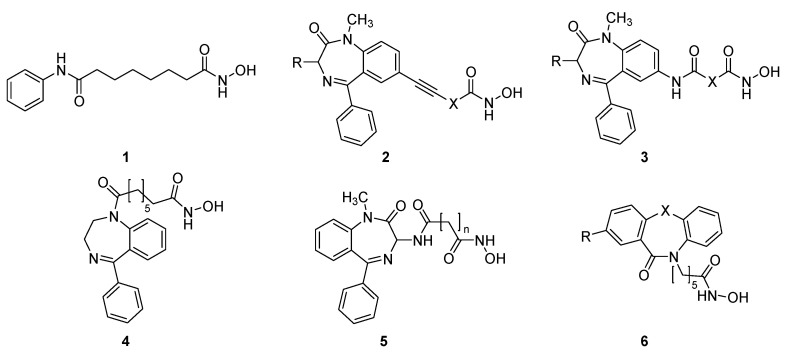
Vorinostat (**1**) and its analogues **2**–**6** based on benzodiazepine scaffolds.

The basic structural features of Vorinostat (**1**) and its analogues include the non-polar aromatic/heteroaromatic cap and a side chain with a terminal hydroxamic acid group capable of binding to zinc ions Zn^2+^. During research on new Vorinostat analogues, compounds **2**–**6** having a bicyclic or tricyclic benzodiazepine ring system (Figure 1) were also obtained [85,86,87,88], exhibiting marked HDAC inhibition and a selective antileukemic effect on tested cell lines.

**Figure 2:**
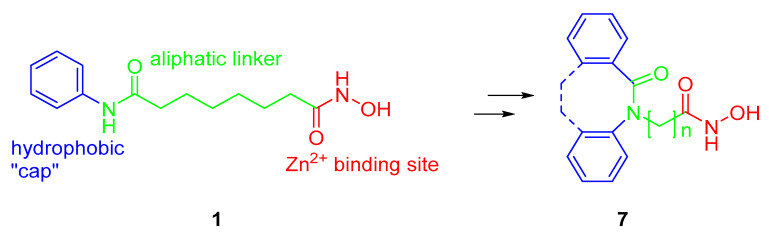
Concept of research and development of novel tricyclic analogues of HDAC inhibitor, Vorinostat.

For years, our research group has been working on the design and synthesis of various mono- and polycyclic dilactam derivatives [89,90,91,92,93,94,95,96,97,98] with potential biological activity. The studies resulted in the discovery of tricyclic benzodiazepines exhibiting selective antileukemic effects [92,93,94]. These compounds could be treated as structural analogues of antitumour antibiotic Anthramycin. Recently, we focused on the development of novel synthetic methods leading to asymmetrically substituted tricylic lactam and dilactam compounds with central, eight-membered heterocyclic rings [95,96,97]. Such structures were used by us for the development of novel analogues of tricyclic drugs exhibiting significant affinity to H_1_ receptors [98]. We envisioned, then, that tricyclic heterocycles with two outer benzene rings and central, azocine or diazocine ring **7** (Figure 2) could be useful scaffolds in the design of novel analogues of Vorinostat, a HDACs inhibitor used for lymphoma treatment [99]. We decided to replace the phenyl group in Vorinostat with various tricyclic ‘caps’ and investigate the HDACs activity as well as potency and selectivity of the cytotoxic effect tested on the cancer and non-cancer cell lines.

## 2. Results and Discussion

### 2.1. Synthesis and HDAC Inhibition

**Scheme 1:**
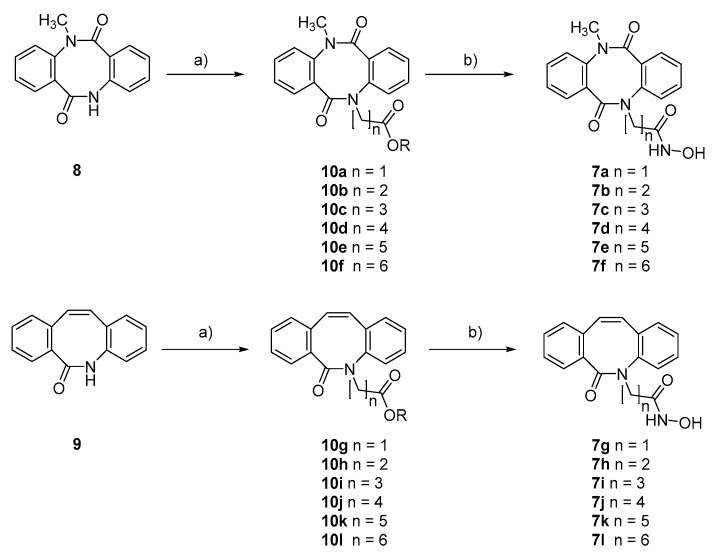
The synthesis of the first **7a**–**f** and second 7**g**–**l** series of compounds: (**a**) Br(CH_2_)nCOOR, NaH, DMSO, 18 h, rt, n = 1–6, R = Me, Et; (**b**) NH_2_OH x H_2_O, THF, MeOH, 18 h, rt.

Since increasing the size of the hydrophobic ‘cap’ in the Vorinostat structure could have had an impact on the optimal length of the side chain terminated with hydroxamic acid, in the first part of our research, we decided to synthesize two homologous series of compounds and used two selected tricyclic ‘caps’: 5-methyldibenzo[*b*,*f*][1,5]diazocine-6,12(5*H*,11*H*)-dione (**8**) [95] (Series 1) and dibenzo[*b*,*f*]azocin-6(5*H*)-one (**9**) [98] (Series 2) and different side chain lengths (Scheme 1).

Previously obtained compounds **8** and **9** were treated with appropriate ω-bromoester in the presence of sodium hydride, resulting in intermediate products **10a**–**l**. After chromatographic purification and isolation, esters **10a**–**l** were treated with hydroxylamine hydrate which led to the final hydroxamic acids **7a**–**l**.

**Figure 3:**
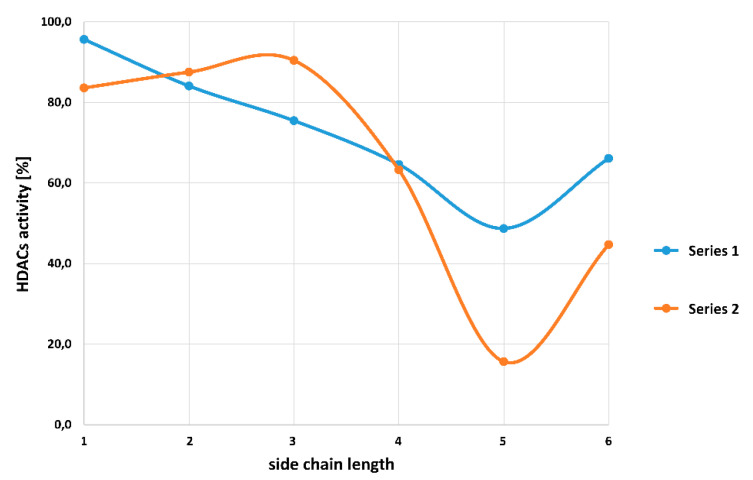
Structure-related activity of Vorinostat derivatives **7a**–**l** showing the percentage of HDACs activity at 2 µM depending on the side chain length.

**Scheme 2:**
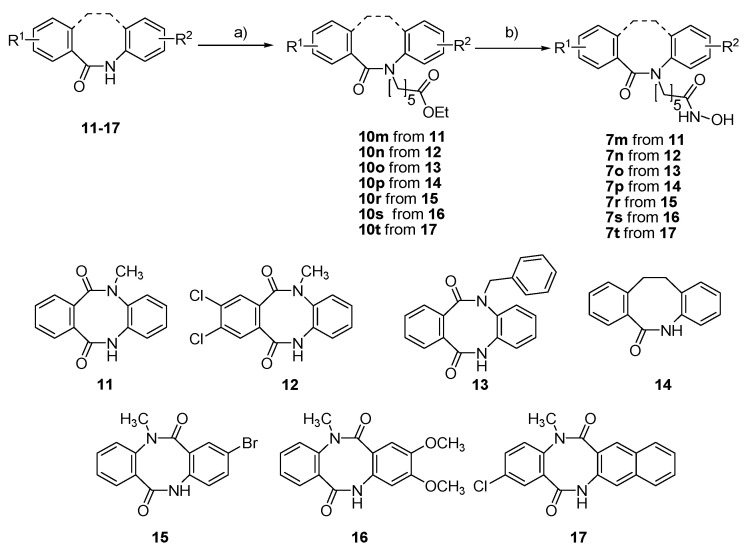
The synthesis of the Series 3 compounds 7**m**–**t**: (**a**) Br(CH_2_)_5_COOEt, NaH, DMSO, 18 h, rt, R = Me, Et; (**b**) NH_2_OH × H_2_O, THF, MeOH, 18 h, rt.

A standard fluorimetric HDACs inhibition kit (Sigma-Aldrich, Darmstadt, Germany) was to determine the inhibitory potency of novel Vorinostat analogues. It involves a two-step enzymatic reaction: deacetylation of the peptide acetylated lysine side chain by the HDACs containing HeLa cell extract followed by a cleavage of the deacetylated substrate by the developer solution and the release of the highly fluorescent group. We evaluated the efficacy of HDACs inhibition activity of 12 newly synthesized Vorinostat derivatives **7a**–**l** and observed a correlation between the side chain length and the HDACs inhibition activity (Figure 3). The obtained results for Series 1 and 2 are presented in Table 1. In both series, the compounds with five-carbon atom linkers (n = 5) were the most active ones; additionally, compound **7k** demonstrated superior inhibitory activity in comparison with reference compound Vorinostat. Because the compounds with a five-carbon side chain turned out to be the most active, thus, in Series 3 (Table 1, Scheme 2), only derivatives with five-carbon side chains were used.

In the next part of our research, we decided to synthesize the compounds of Series 3 and to use seven tricyclic ‘caps’: 5-methyl-5,12-dihydrodibenzo[*b,f*][1,4]diazocine-6,11-dione (**11**) [96], 2,3-dichloro-5-methyl-5,12-dihydrodibenzo[*b,f*][1,4]diazocine-6,11-dione (**12**) [96], 5-benzyl-5,12-dihydrodibenzo[*b,f*][1,4]diazocine-6,11-dione (**13**) [96], 11,12-dihydrodibenzo[*b,f*]azocin-6(5*H*)-one (**14**) [98], 2-bromo-11-methyldibenzo[*b,f*][1,5]diazocine-6,12(5*H*,11*H*)-dione (**15**) [95], 2,3-dimethoxy-11-methyldibenzo[*b,f*][1,5]diazocine-6,12(5*H*,11*H*)-dione (**16**) [95], and 2-chloro-5-methylbenzo[*b*]naphtho[2,3-*f*][1,5]diazocine-6,14(5*H*,13*H*)-dione (**17**) [95] (Scheme 2). Previously obtained compounds **11**–**17** were treated with ethyl 6-bromohexanoate in the presence of sodium hydride which resulted in the intermediate products **10m**–**t**. After chromatographic purification and isolation, esters **10m**–**t** were treated with hydroxylamine hydrate, resulting in the final hydroxamic acids **7m**–**t**. Using a standard fluorimetric HDAC inhibition kit (Sigma-Aldrich) to determine the inhibitory potency of the series 3 Vorinostat analogues, we observed that tested the compounds inhibited HDAC activity to a different degree; however, two of them, namely, **7p** and **7t**, showed even higher activity than the reference Vorinostat.

**Figure 4:**
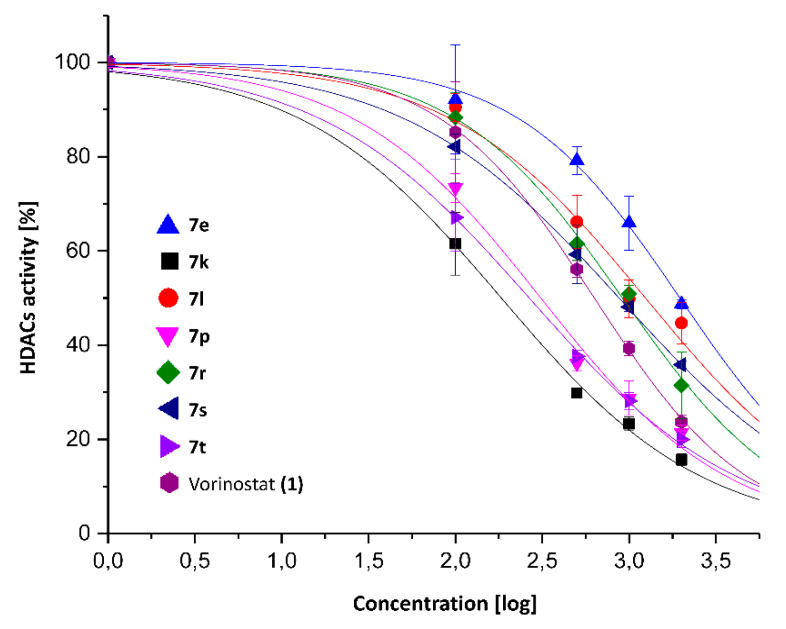
HDACs activity inhibition for the most active compounds.

Among 19 newly synthesized Vorinostat analogues, IC_50_ was determined for the seven most active and promising derivatives: **7e**, **7k**, **7l**, **7p**–**t** (Figure 4, Table 2). The lowest values were achieved for compounds **7k** with dibenzo[*b,f*]azocin-6(5*H*)-one (**9**) ‘cap’ (IC_50_ = 0.183 µM), **7t** with 11,12-dihydrodibenzo[*b,f*]azocin-6(5*H*)-one ‘cap’ (**14**) (IC_50_ = 0.266 µM), and **7p** with 2-chloro-5-methylbenzo[*b*]naphtho[2,3-*f*][1,5]diazocine-6,14(5*H*,13*H*)-dione ‘cap’ (**17**) (IC_50_ = 0.309 µM). These values exceeded the value obtained for the reference Vorinostat inhibiting HDAC with IC_50_ = 0.630 µM. Tricyclic ‘caps’ **9**, **14**, and **17** together with a five-carbon side chain were optimal for the high inhibitory activity of the compounds tested. At the same time, the two derivatives **7r** and **7s**, possessing a dibenzo[*b,f*][1,5]diazocine-6,12(5*H*,11*H*)-dione central unit showed a slightly weaker but still comparable activity to Vorinostat (IC_50_ = 0.875 µM and 0.914 µM, respectively). Compounds **11**–**13**, all based on the 5,12-dihydrodibenzo[*b,f*][1,4]diazocine-6,11-dione structure, exhibited very poor or no activity against HDAC homologues.

We performed *in silico* molecular docking which revealed that the HDAC8 homologue is the most promising target for compounds used: **7e**, **7k**, **7p** and **7t** (the most significant differences in the interaction pattern between active and inactive compounds along with Vorinostat, see Molecular Modelling section). For this reason, we evaluated the efficacy of the HDAC8 inhibition activity of **7e**, **7k**, **7p**, and **7t** with Vorinostat as the reference (Table 2). We observed that all tested compounds exhibited a significant inhibitory effect on HDAC8. The lowest value, IC_50_ = 1.51 ± 0.13 µM, was obtained for Vorinostat. All new compounds showed a comparable, yet slightly lower effect with IC_50_ values in the range of 1.95 ± 0.17 µM for **7t** to 5.67 ± 0.64 µM for **7e**. Lower IC_50_ values obtained for the mixture of HDAC homologues, compared with the IC_50_ values for HDAC8 protein, may indicate that other HDAC homologues found in the cell lysate are more susceptible to the tested compounds, and could show lower IC_50_ than HDAC8. An important factor that should also be taken into account are the different concentrations of individual HDAC homologues in the cell lysate influencing the total enzymatic activity of the mixture of HDAC homologues.

### 2.2. Cytotoxic Activity and Selectivity Index

For the selection of the most promising cytotoxic agents, all 19 newly synthesized compounds, as well as the reference HDAC inhibitor, Vorinostat, were initially tested on two cancer cell lines: MV4-11 (biphenotypic B myelomonocytic leukemia) and Daudi (Burkitt’s lymphoma) (Table 3). Five compounds: **7k** and **7p**–**t**, exhibited IC_50_ below 1 µM, in the range of 0.093 µM (**7t**) to 0.692 µM (**7s**) on MV4-11 and in the range of 0.137 µM (**7t**) to 0.944 µM (**7s**) on Daudi. In the case of leukemic cell line MV4-11, three out of five compounds, **7k**, **7p**, and **7t**, showed lower IC_50_ values than Vorinostat (0.220, 0.200, and 0.093 µM, respectively, versus 0.636 µM). Again, with the lymphoma cell line, Daudi, **7k**, **7p**, and **7t** showed lower (or comparable) IC_50_ values than Vorinostat (0.460, 0.318, and 0.137 µM, respectively, versus 0.493 µM). The most potent cytotoxic compounds, **7k** and **7p**–**t**, were further evaluated for their cytotoxic effect on two solid tumour cancer cell lines: A549 (lung carcinoma) and MCF-7 (breast adenocarcinoma). To determine the selectivity of the tested compounds, one reference cell line BALB/3T3 (mouse fibroblasts), derived from a non-cancerous cell line was also used. In the case of A549 cell line, two compounds, **7t** and **7p**, showed lower IC_50_ values than Vorinostat (1.05 and 1.21, respectively, versus 1.64 µM). Similarly, in the case of the MCF-7 cell line, two compounds, **7t** and **7p**, showed lower (or comparable) IC_50_ values than Vorinostat (0.368 and 0.661, respectively, versus 0.685 µM). Compounds **7p** and **7t** exhibited the strongest cytotoxic effect on cancer cell lines but the observed cytotoxicity also extended to the reference normal fibroblasts cell line. The two most active compounds **7t** and **7p** were also more toxic to BALB/3T3 than Vorinostat (0.69, 1.04, respectively, versus 1.42 µM).

The obtained results showed a close correlation between HDAC inhibition and the cytotoxic effect of the tested compounds. The three most potent HDAC inhibitors: **7k**, **7p**, and **7t** also showed the strongest cytotoxic effect on tested cell lines.

To determine the selectivity of tested compounds, we compared the cytotoxic effect observed for cancer cell lines (MV4-11, Daudi, A549, MCF-7) and the reference line BALB/3T3. The selectivity indexes were calculated for the five most active compounds (**7k**, **7p**–**7t**) and Vorinostat (Table 4). In all the cases, the Vorinostat selectivity index never exceeded the value of 3 and varied from 2.88 (Daudi) to 0.87 (A549). The highest selectivity indexes were obtained for compounds **7s** (17.4 for MV4-11 and 12.75 for Daudi) and **7t** (7.42 for MV4-11 and 5.05 for Daudi). The remaining compounds **7k**, **7p**, and **7r** (except for **7k** and Daudi) also possessed higher selectivity indexes for MV4-11 and Daudi than Vorinostat. We observed that while Vorinostat was slightly more selective for Daudi (lymphoma) than for MV4-11 (leukemia), our compounds exhibited better selectivity toward MV4-11 (leukemia) than for Daudi (lymphoma). In general, Vorinostat, as well as the newly synthesized compounds, exhibited relatively low selectivity toward solid tumour cancer cell lines (A549 and MCF-7) as compared with the BALB/3T3 reference line. MCF-7 line more strongly expresses HDAC 1, 2, 3, and 8 in comparison with the A549 line; this concerns especially HDAC 1 and 8 [100].

### 2.3. Molecular Modelling

To define and elucidate the binding modes of the synthesized compounds, molecular docking of these compounds in the active site of histone deacetylases 1, 2, 3, 4, 6, 7, and 8 (HDACs) was performed. Among all HDACs’ structures stored in PDB, only structures with the co-crystallized ligand and binding pocket exposed to the solvent were taken into account. Structures which were co-crystallized with Vorinostat and had the best resolution were preferred. Finally, one crystal structure per HDAC was selected (Table 5) and prepared in Protein Preparation Wizard [101,102] under default settings (coordination of zinc ion was set as constraint, centered on ligand). The three-dimensional structure, conformation, and protonation states of the evaluated compounds were generated by LigPrep (at pH 7.4) and Epik [102,103,104]. Finally, Glide [102,103,104,105,106,107] was used for docking each compound to every protein crystal. Each pose was ranked according to docking score (the lower value, the better), and the best scored pose per compound was chosen for further analysis.

**Figure 5:**
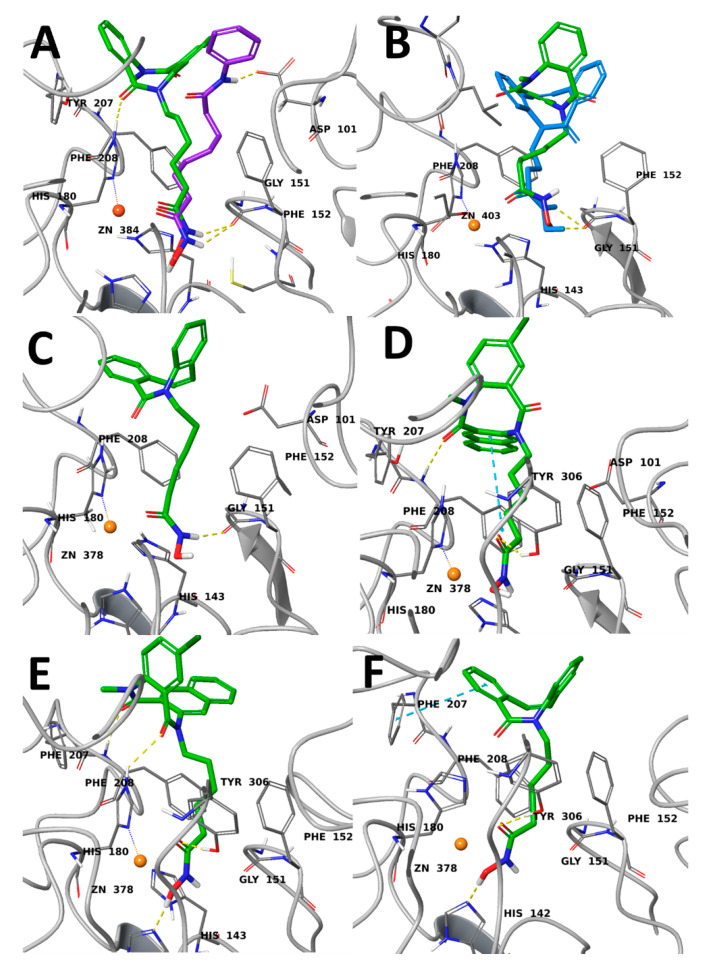
Representative L−R virtual complexes of (**A**) **7e** (rendered in green) and Vorinostat (violet) in the binding pocket of HDAC1 (PDBid: 5ICN), (**B**) **7e** (green), and **7i** (blue) in the binding pocket of HDAC4 (PDBid: 2VQM), (**C**) **7k** (green) in binding pocket of HDAC7 (PDBid: 3C0Z), (**D**) **7t** (green) in the binding pocket of HDAC2 (PDBid: 4LXZ), (**E**) **7t** (green) in the binding pocket of HDAC6 (PDBid: 5EDU) and (**F**) **7k** (green) in the binding pocket of HDAC8 (PDBid: 1T69). Yellow dashed lines indicate hydrogen bonds, whereas blue indicate aromatic interactions. Zinc ion is rendered in orange.

The majority of the evaluated compounds were docked to six out of seven HDAC types. Only HDAC3 was unable to form a protein-ligand complex with the synthesized compounds and only Vorinostat was docked to this crystal. Binding modes for these evaluated compounds in binding pockets of HDACs 1, 3, and 7 were both very similar for all docked compounds. Active compounds (**7e**, **7k**, **7t**, and **7p**) docked to the HDAC1 did not have optimized geometry of zinc ion coordination despite the fact that they had very low (below −8, see Table S1, SI file) values of the scoring function. Both active and inactive compounds shared the same interaction pattern, involving amino acid residues H180 and G151, and some of them even F208. In the binding mode of Vorinostat, the additional interaction with residue D101 could be observed, but there was no hydrogen bond with H180 (Figure 5A). Docking results for the HDAC3 structure allowed distinguishing between active from inactive compounds using the scoring function (four active compounds were in the five top scoring compounds; values below −6, see Table S1, SI file), but the interaction profile of active and inactive compounds were very similar. Almost all compounds interacted with the zinc ion and residue G151, while contacts with other residues were rarely formed. Nevertheless, the hydrophobic cap of inactive compounds (e.g., for **7i**) had a somewhat different orientation in the binding pocket than for active ones. (Figure 5B). The interaction pattern for Vorinostat with the HDAC3 binding pocket was extremely poor: the compound interacted only with the zinc ion. For HDAC7, binding poses did not allow for separating active from inactive compounds. Almost all compounds interacted with the zinc ion and residue G151, but there were no specific interactions observed for active compounds only (Figure 5C). In contrast, the binding mode of active compounds was different than inactive compounds for HDACs 2, 6, and 8. In the case of HDAC2, active compounds created hydrogen bonds with residues Y306 and Y207. Moreover, they interacted via π-π stacking interaction with F208 (Figure 5D). These interactions were not commonly observed neither for inactive compounds nor for Vorinostat. Similar observations can be seen for HDAC6. Active compounds, except commonly observed interactions with residues Y306 and H143, interacted with residue H180 and formed at least one hydrogen bond with an aromatic cluster, i.e., F207 and F208 (Figure 5E). This interaction with F207 or F208 enriches the interaction profile which characterizes Vorinostat in HDAC6 binding mode. In HDAC8 crystal structures, Vorinostat interacts only with zinc ion and residue H143 and this interaction profile was shared by inactive compounds. Active compounds showed additional interactions with Y306 and F207 (hydrogen bonds or π-π stacking, Figure 5F), which were not observed for the remaining compounds.

## 3. Materials and Methods

### 3.1. Chemistry

Commercially available chemicals were of reagent grade and used as received. The reaction progress was monitored using LR-ESI-MS spectra and thin layer chromatography (TLC) using silica gel plates (Kieselgel 60F_254_, E. Merck, Darmstadt, Germany). Column chromatography used for purification and isolation of compounds was performed on silica gel 60 M (0.040–0.063 mm, E. Merck, Darmstadt, Germany). Melting points were measured using Büchi (New Castle, DE, USA) Melting Point B-540 apparatus. All ^1^H and ^13^C NMR spectra were recorded on a Bruker Avance III spectrometer operating at 500.13 (^1^H) and 125.77 (^13^C) MHz and equipped with a 5 mm probe head with Z-gradient coils. The experiments were performed using pulse programs from standard Bruker library for samples dissolved in CDCl_3_, DMSO-*d_6_,* or MeOH-*d_4_*. In each case, spectra were calibrated at residual solvent resonances. High resolution mass spectra were performed by the Laboratory of Mass Spectrometry, Institute of Biochemistry and Biophysics PAS, on a LTQ Orbitrap Velos instrument, Thermo Scientific (Waltham, MA, USA). Synthetic procedures, physicochemical properties, and spectra related to synthesized compounds are included in the supplementary file.

### 3.2. Biology

#### 3.2.1. Cell Culturing

Human biphenotypic B myelomonocytic leukemia MV4-11 and normal mouse fibroblast BALB/3T3 cell line were obtained from American Type Culture Collection (USA); human lung carcinoma A549 cell line and human adenocarcinoma breast cancer MCF-7 cell line were obtained from European Collection of Authenticated Cell Cultures (UK). Human Burkitt’s lymphoma Daudi cell line was obtained from DSMZ-German Collection of Microorganisms and Cell Cultures (Germany). All the cell lines are being maintained at the Hirszfeld Institute of Immunology and Experimental Therapy, PAS, Wroclaw, Poland.

MV4-11 and Daudi cell lines were cultured in RPMI1640 medium (General Chemistry Laboratory of Hirszfeld Institute of Immunology and Experimental Therapy, Polish Academy of Science (HIIET PAN), Wrocław, Poland) supplemented with 10% fetal bovine serum (FBS), 2 mM L-glutamine, and 1 mM sodium pyruvate (all from Merck). A549 cell line was cultured in RPMI1640 + OptiMEM (50:50; GChL of HIIET PAN and Gibco) with 5% FBS, 2 mM L-glutamine (all from Merck). MCF-7 cell line was cultured in Eagle medium (GChL of HIIET PAN) with 10% FBS, 2 mM L-glutamine, 8 µg/mL insulin, 1% (*v*/*v*) MEM NON-ESSENTIAL amino acid solution 100× (all from Merck). BALB/3T3 cell line was cultured in DMEM medium (Gibco) supplemented with 10% FBS, 2 mM L-glutamine (all from Merck). All cultured media were supplemented with antibiotics: 100 units/mL penicillin (Polfa Tarchomin, Warsaw, Poland) and 100 µg/mL streptomycin (Merck). Cells were grown in a humidified atmosphere of CO_2_/air (5/95%) at 37 °C.

#### 3.2.2. Histone Deacetylase Activity Assay (HDACs Activity)

In order to measure histone deacetylase activity, Histone Deacetylase Activity Assay (HDACAA) (Sigma) was used (Catalog Number CS1010). The HDACAA kit is based on a two-step enzymatic reaction. The substrate for the reaction is a substituted peptide with an acetylated lysine residue and a bound fluorescent group. The first step of the reaction is deacetylation of the acetylated lysine side chain by the HDAC containing sample (HeLa cell extract). The second step is the cleavage of the deacetylated substrate with the developer solution and the release of the free highly fluorescent group. The reaction reagents were added to the wells of a 96 well plate according to Table 6. The plate was incubated at 30 °C for 30 min. Next, 10 µL of Developer Solution was added to each well and incubated at room temperature for 10 min. The fluorescence was measured with the fluorimeter plate reader (SpectraMax iD3, Molecular Devices): excitation wavelength of 360 nm and emission wavelength of 460 nm. The following concentrations were used: 0.1, 0.5, 1, and 2 µM. Each compound in each concentration was tested in triplicate. All calculations were performed using Origin 9.0 software.

#### 3.2.3. Histone Deacetylase 8 Activity Assay

In order to measure histone deacetylase 8 activity, Histone Deacetylase 8 Activity Assay (Sigma) was used. The HDAC8 acts with the supplied Developer to deacetylate and then cleave the HDAC8 Substrate (R-H-K(Ac)-K(Ac)-AFC). The reaction reagents were added to the wells of a 96-well plate according to Table 7. The plate was incubated at 37 °C for 1 h. Next, 10 µL of Developer Solution was added to each well and incubated at 37 °C for 5 min. The fluorescence was measured with the fluorimeter plate reader: excitation wave-length of 380 nm and emission wavelength of 500 nm. The following concentrations were used: 0.5, 1, 1.5, 2, 3, and 4 µM. Each compound in each concentration was tested in triplicate. All calculations were performed using Origin 9.0 software.

#### 3.2.4. Cytotoxicity Assay

Exponentially growing cells were seeded onto a 96-well plate (Sarstedt) at the density of 10^4^ cells/well (MV4-11, Daudi, BALB/3T3), 0.75 × 10^4^ cells/well (MCF-7), or 0.5 × 10^4^ cells/well (A549) in 100 μL of culture medium and cultured for 24 h (37 °C, 5% of CO_2_). The solutions of the newly synthesized Vorinostat derivatives (25 mM) were prepared by dissolving the substances in DMSO (Merck). Then, the tested compounds were diluted in culture medium (RPMI1640 + OptiMEM) to reach the final concentrations. Then, cells were treated for 72 h with derivatives at concentrations of 50, 10, 2, 0.4, and 0.008 µM (100 μL of each concentration per well), or with culture medium alone as a cells control or medium control. DMSO (at concentrations 0.2%, 0.04%, 0.008%, and 0.0016% (*v*/*v*) which correspond to concentrations of DMSO in compounds’ final concentrations: 50, 10, 2, 0.4 μM) was included in the experiments as a solvent control. We observed a very slight influence on cell growth: max. 20% of cell growth inhibition of 0.2% DMSO. Proliferation inhibition readings were performed using the MTT (MV4-11 and Daudi) or SRB (MCF-7, A549 and BALB/3T3) method.

Each compound in each concentration was tested in triplicate in a single experiment, which was repeated 3–5 times. The results were calculated as an IC_50_ (inhibitory concentration 50%) the concentration of tested agent, which is cytotoxic for 50% of the cancer cells. IC values were calculated for each experiment separately using Prolab-3 system based on Cheburator 0.4 software and data are presented as mean ± standard deviation (SD) [108].

SRB: 50 µL 50% cold trichloroacetic acid (TCA, Merck) solution was added to each wells and incubated for 1 h at 4 °C. The plates were rinsed with distilled water, and after drying on a paper towel, 50 µL of a 0.1% (*w*/*v*) solution of sulforodamine B (Merck) in 1% acetic acid (POCH, Poland) was added and incubated for 30 min at room temperature. Subsequently, the plates were washed with 1% (*v*/*v*) acetic acid and after desiccation of excess acid, 150 µL of 10 mM TRIS (Merck) was added. After another 30 min of incubation at RT, the optical density of individual samples was read at 540 nm using a plate reader (Synergy H4 Hybrid Reader, BioTek).

MTT: 20 µL MTT solution (5 mg/mL of PBS, Merck) was added to wells and plates were placed in an incubator. After 4 h of incubation at 37 °C, 80 µL of lysis buffer (SDS, DMF and water, Merck) was added and incubation continued for 24 h. The optical density of individual samples was read at a wavelength of 570 nm using a plate reader (Synergy H4 Hybrid Reader, BioTek).

### 3.3. In Silico Modelling

#### Compounds Preparation

For all compounds analyzed within this study, ionization states were generated at pH = 7.4 using Epik software. Ligprep software (under the default settings: generation of only one low energy ring conformation per ligand, retention of specified chiralities, and force field used OPLS2005) was applied for the generation of 3D structures (2.3.2. Docking protocol). All receptors have been centered on the ion zinc located inside the binding pocket. Grid box size was set to 25 × 25 × 25Å. All the docking calculations were run in Glide software at the SP level under the default settings (performing post-docking optimization, up to 100 steps during energy minimization, penalizing nonplanar conformation of amides, sampling ring conformations with energy window equal to 2.5 kcal·mol^−1^ and sampling nitrogen inversion). Docking was carried out with one constraint: mandatory coordination of the zinc ion.

## 4. Conclusions

We synthesized 19 novel HDAC inhibitors based on the Vorinostat structure. The introduction of a larger tricyclic hydrophobic ‘cap’ to the structure of Vorinostat in the place of the phenyl group was beneficial for biological properties and allowed for the development of compounds with improved HDAC inhibition, stronger cytotoxic effects, and higher selectivity against leukemia and lymphoma cell lines. We observed that the enlargement of the hydrophobic ‘cap’ from a single benzene ring to a heterocyclic three-ring system forced a shortening of the length of the linker connecting the hydrophobic group to the hydroxamic acid residue. We also observed that the biological properties of the tested compounds (HDAC inhibition and the resulting cytotoxic effect) strongly depended on the tricyclic core used. In general, compounds **7m**–**o** having a central, dilactam, 1,4-azocine ring in their structure showed relatively the lowest biological activity in terms of both HDAC inhibition and cytotoxic effect. Although only a limited number of such compounds have been obtained, it can be assumed that the tricyclic dibenzodiazocines with a central, dilactam 1,4-diazocine ring are rather unsuitable for the design of new Vorinostat analogues. Compounds with a central, dilactam 1,5-diazocine ring in their structure showed good (tricyclic **7e**, **7r**, **7s**) to excellent (tetracyclic **7t**) biological activity in terms of both HDAC inhibition and cytotoxic effect and could be considered in the design of new Vorinostat analogues. Tricyclic compounds **7k** and **7p**, possessing tricyclic caps with azocine monolactam central rings are also good candidates for future development of novel, more potent, and selective HDAC inhibitors. We also concluded that for optimal biological properties, an appropriate balance between the size/type of the hydrophobic group and the length of the side chain, terminated in the hydroxamic acid group, is necessary. We also observed a strong correlation between HDAC inhibition and cytotoxicity, so we conclude that HDAC inhibition should be the main factor responsible for the observed biological properties of the developed compounds. The tested HDAC inhibitors exhibited a stronger and more selective cytotoxic effect against MV4-11 and Daudi, while the cell lines derived from solid tumours and mouse fibroblasts proved to be much less sensitive to our compounds. Thus, we conclude that they can be considered as selective compounds against leukemias and lymphomas.

## Figures and Tables

**Table 1:** HDACs inhibition of newly synthesized Vorinostat derivatives **7a**–**l**. For Vorinostat (**1**) HDACs activity at 2 µM was 23.8 ± 2.2.

Series 1							
**Compound**	**7a**	**7b**	**7c**	**7d**	**7e**	**7f**	
HDAC activity at 2 µM [%]	95.6 ± 1.4	84.1 ± 2.3	75.4 ± 3.0	64.6 ± 3.6	48.7 ± 1.0	66.1 ± 4.1	
Series 2							
**Compound**	**7g**	**7h**	**7i**	**7j**	**7k**	**7l**	
HDAC activity at 2 µM [%]	83.6 ± 4.2	87.5 ± 1.2	90.4 ± 1.6	63.3 ± 0.5	15.7 ± 1.3	44.7 ± 4.4	
Series 3							
**Compound**	**7m**	**7n**	**7o**	**7p**	**7r**	**7s**	**7t**
HDAC activity at 2 µM [%]	72.7 ± 5.2	68.3 ± 2.8	64.5 ± 2.1	21.5 ± 1.6	31.5 ± 7.1	35.9 ± 0.8	19.9 ± 1.6

**Table 2:** Inhibition of HDAC homologues (HDACs) and HDAC8 activity (IC_50_ [µM]) for selected compounds. N/T—not tested.

Compound	HDACs	HDAC8
**7e**	1.959 ± 0.105	5.67 ± 0.64
**7k**	**0.183 ± 0.015**	3.37 ± 0.33
**7l**	1.241 ± 0.155	N/T
**7p**	**0.309 ± 0.035**	3.14 ± 0.42
**7r**	0.914 ± 0.053	N/T
**7s**	0.875 ± 0.017	N/T
**7t**	**0.266 ± 0.014**	1.95 ± 0.17
Vorinostat (**1**)	0.630 ± 0.011	1.51 ± 0.13

**Table 3:** IC_50_ [µM] of Vorinostat derivatives **7a-t** based on the survival of non-cancerous (BALB/3T3) and cancerous (MV4-11, Daudi, MCF-7 and A549) cells after 72 h of treatment. N/T—not tested.

	Compound	IC_50_ [µM]				
		MV4-11	Daudi	A549	MCF-7	BALB/3T3
**Series 1**	**7a**	>50.00	>50.00	N/T	N/T	N/T
	**7b**	>50.00	>50.00	N/T	N/T	N/T
	**7c**	>50.00	>50.00	N/T	N/T	N/T
	**7d**	7.58 ± 2.50 *	9.6 ± 2.10 *	N/T	N/T	N/T
	**7e**	2.33 ± 0.64 *	3.03 ± 0.55 *	N/T	N/T	N/T
	**7f**	4.45 ± 0.34 *	4.83 ± 1.96 *	N/T	N/T	N/T
**Series 2**	**7g**	33.97 ± 0.75 *	17.31 ± 6.2 *	N/T	N/T	N/T
	**7h**	>50.00	32.62 ± 4.78 *	N/T	N/T	N/T
	**7i**	>50.00	>50.00	N/T	N/T	N/T
	**7j**	5.30 ± 1.36 *	3.47 ± 0.66 *	N/T	N/T	N/T
	**7k**	0.220 ± 0.021 *	**0.460 ± 0.122**	1.27 ± 0.42	0.618 ± 0.095	1.28 ± 0.15
	**7l**	1.57 ± 0.12 *	1.44 ± 0.61 *	N/T	N/T	N/T
**Series 3**	**7m**	4.45 ± 0.96 *	5.09 ± 0.3 *	N/T	N/T	N/T
	**7n**	2.85 ± 0.34 *	2.56 ± 0.98 *	N/T	N/T	N/T
	**7o**	3.55 ± 0.75 *	3.52 ± 0.80 *	N/T	N/T	N/T
	**7p**	0.200 ± 0.073 *	**0.318 ± 0.098**	1.21 ± 0.24	0.661 ± 0.12	1.04 ± 0.28
	**7r**	**0.603 ± 0.132**	**0.785 ± 0.246**	4.61 ± 0.38 *	2.72 ± 0.57 *	3.37 ± 0.87 *
	**7s**	**0.692 ± 0.110**	0.944 ± 0.167 *	17.96 ± 5.77 *	4.24 ± 1.03 *	12.04 ± 5.9 *
	**7t**	0.093 ± 0.009 *	0.137 ± 0.04 *	1.05 ± 0.07 *	0.368 ± 0.015 *	0.69 ± 0.05 *
	Vorinostat (**1**)	0.636 ± 0.092	0.493 ± 0.093	1.64 ± 0.32	0.685 ± 0.06	1.42 ± 0.23

* statistically significant vs. Vorinostat; *p* < 0.05, Stastistica 10, non-parametric *t*-test.

**Table 4:** Selectivity index (IC_50_ of normal vs. cancer cells). SI > 1.0 indicates a compound of greater activity against cancer cells and lower cytotoxicity on normal cells.

Compound	MV4-11	Daudi	A549	MCF-7
**7k**	5.82	2.78	1.01	2.07
**7p**	5.20	3.27	0.86	1.57
**7r**	5.59	4.29	0.73	1.24
**7s**	17.4	12.75	0.67	2.84
**7t**	7.42	5.04	0.66	1.88
Vorinostat (**1**)	2.23	2.88	0.87	2.07

**Table 5:** PDBids of crystals used in docking studies.

Histone Deacetylase	PDBid	Ligand	Resolution [Å]
HDAC1	5ICN	GAXRH (peptide)	3.30
HDAC2	4LXZ	Vorinostat	1.85
HDAC3	4A69	I0P	2.06
HDAC4	2VQM	HA3	1.80
HDAC6	5EDU	Trichostatin A	2.79
HDAC7	3C0Z	Vorinostat	2.10
HDAC8	1T69	Vorinostat	2.91

**Table 6:** Reaction Scheme for HDAC Activity.

Component	Assay Buffer [µL]	HDAC Inhibitor Solution [µL]	HeLa Lysate [µL]	DMSO [µL]	HDAC Substrate Solution [µL]
HDAC activity assay	40	-	5	5	50
HDAC activity inhibition assay	40	5	5	-	50
Blank	45	-	-	5	50

**Table 7:** Reaction scheme for HDAC8 Activity.

Component	HDAC8 Assay Buffer [µL]	HDAC Inhibitor Solution [µL]	HDAC8	DMSO [µL]	HDAC8 Substrate Solution [µL]
HDAC activity assay	43	-	2	5	50
HDAC activity inhibition assay	43	5	2	-	50
Blank	45	-	-	5	50

## Data Availability

Data is contained within the article and Supplementary Material.

## Supplementary Materials

The following are available online at https://www.mdpi.com/article/10.3390/ph14090851/s1, Table S1: Results of molecular docking, Figures S1–S38: ^1^H NMR spectra, Figures S39–S76: ^13^C NMR spectra, Figures S77–S114: HRMS spectra, synthetic procedures and physicochemical data.
