# Supplementary material for: Improved HDAC Inhibition, Stronger Cytotoxic Effect and Higher Selectivity against Leukemias and Lymphomas of Novel, Tricyclic Vorinostat Analogues
Source: Pharmaceuticals (Basel). 2021 Aug 26;14(9):851. doi: 10.3390/ph14090851 (PMC8470702; doi:10.3390/ph14090851)
Supplement: Supplementary file 1 [file pharmaceuticals-14-00851-s001.zip › pharmaceuticals-1327412-supplementary.pdf]

Improved HDAC Inhibition, Stronger Cytotoxic Effect and Higher Selectivity against Leukemias and Lymphomas of Novel, Tricyclic Vorinostat Analogues

Bartosz Bieszczad,[1,†] Damian Garbicz,[1,†] Marta Świtalska,[2] Marta K. Dudek,[3] Dawid Warszycki,[4] Joanna Wietrzyk,[2] Elżbieta Grzesiuk [1,*] and Adam Mieczkowski [1,*]

1 Institute of Biochemistry and Biophysics, Polish Academy of Sciences, 02-106 Warsaw, Poland; b.bieszczad@ibb.waw.pl (B.B.)

2 Hirszfeld Institute of Immunology and Experimental Therapy, Polish Academy of Sciences, 53-114 Wrocław, Poland; marta.switalska@hirszfeld.pl (M.Ś), joanna.wietrzyk@hirszfeld.pl (J.W.)

3 Centre of Molecular and Macromolecular Studies, Polish Academy of Sciences, 90-363 Lodz, Poland; mdudek@cbmm.lodz.pl

4 Maj Institute of Pharmacology, Polish Academy of Sciences, 31-343 Cracow, Poland; warszyc@if-pan.krakow.pl

* Correspondence: elag@ibb.waw.pl (E.G.); amiecz@ibb.waw.pl (A.M.); Tel.: +48-22-592-3506 (A.M.)

† These authors contributed equally to this work.

1. Materials and Methods

1.1. *In silico* modelling

Table S1. Glide Score for the best scored pose (the lower the better) for every compound in seven different HDAC crystals (PDBids given in parenthesis).

Compound	HDAC1 (5ICN)	HDAC2 (4LXZ)	HDAC3 (4A69)	HDAC4 (2VQM)	HDAC6 (5EDU)	HDAC7 (3C0Z)	HDAC8 (1T69)
**7a**	−2.752	not docked	not docked	−2.938	not docked	−2.412	−3.697
**7b**	−5.643	−2.555	not docked	−4.142	−5.169	−4.862	−3.737
**7c**	−6.712	−4.997	not docked	−4.210	−8.164	−5.751	−7.762
**7d**	−7.615	−7.028	not docked	−5.237	−8.593	−6.613	−8.860
**7e**	−8.642	−7.826	not docked	−6.567	−8.517	−7.324	−9.251
**7f**	−8.477	−6.260	not docked	−5.816	−8.277	−6.883	−8.472
**7g**	−2.994	−1.488	not docked	−3.413	−3.831	−4.057	−4.118
**7h**	−6.009	−3.531	not docked	−3.817	−7.035	−6.403	−4.786
**7i**	−6.173	−4.353	not docked	−4.429	−8.649	−6.583	−8.015
**7j**	−8.655	−7.077	not docked	−5.092	−8.490	−7.329	−8.232
**7k**	−8.363	−8.129	not docked	−6.245	−8.368	−7.152	−8.839
**7l**	−8.161	−7.568	not docked	−5.701	−7.988	−7.074	−8.634
**7m**	−8.585	−6.634	not docked	−5.843	−8.663	−6.930	−8.532
**7n**	−8.470	−6.525	not docked	−5.582	−8.442	−6.800	−8.754
**7o**	−7.927	−7.892	not docked	−5.789	−8.430	−7.425	−8.423

**7p**	−8.653	−7.679	not docked	−6.143	−8.607	−7.134	−8.038
**7r**	−7.790	−7.353	not docked	−6.138	−8.533	−6.909	−8.374
**7s**	−9.187	−7.644	not docked	−6.446	−8.183	−7.354	−8.675
**7t**	−8.411	−7.450	not docked	−6.690	−9.083	−6.775	−9.070
Vorinostat	−5.259	−5.777	−3.798	−3.625	−6.043	−3.961	−5.736

2. Synthetic procedures and physicochemical data

2.1 General procedure for alkylation of 8-membered lactams

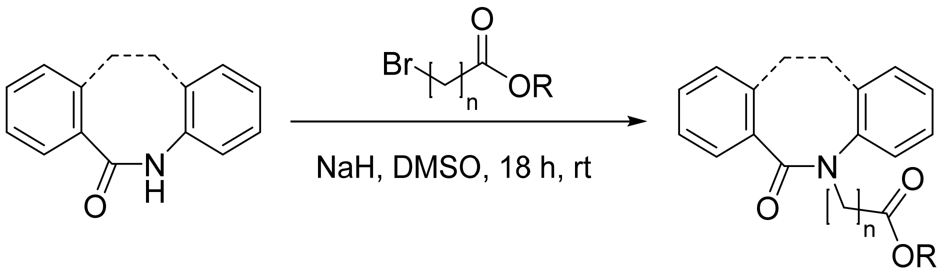

To a solution of appropriate heterocyclic amide (1.0 equiv.) in anhydrous DMSO (5 mL/mmol), 60% sodium hydride in mineral oil (1.5 equiv.) was added and the resulting mixture was stirred for 30 min. After the addition of alkyl halide (1.5 equiv.) the reaction mixture was stirred for 18 hrs at room temperature, then was poured into water and the crude product was extracted with ethyl acetate (3 x 50 mL). The organic phase was washed with brine (1 x 50 mL) and dried over anhydrous magnesium sulfate. The crude product was purified by column chromatography (hexane:ethyl acetate; gradient).

ethyl 2-(11-methyl-6,12-dioxo-11,12-dihydrodibenzo[b,f][1,5]diazocin-5(6H)-yl)acetate (**10a**): Yield: 78 %, white crystals, mp 125.0-126.0 °C, Rf = 0.33 (hexane:ethyl acetate 1:1). **^{1}H NMR** (500 MHz, $CDCl_3$) δ 7.31 – 7.12 (m, 7H, H_{Ar}), 7.04 (d, *J* = 8.0 Hz, 1H, H_{Ar}), 4.56 (d, *J* = 17.1 Hz, 1H, H_{CH}), 4.32 (d, *J* = 17.1 Hz, 1H, H_{CH}), 4.29 – 4.16 (m, 2H, H_{CH2}), 3.41 (s, 3H, H_{CH3}), 1.26 (t, *J* = 7.2 Hz, 3H, H_{CH3}); **^{13}C NMR** (126 MHz, $CDCl_3$) δ 168.6, 168.4, 168.1, 140.5, 139.5, 134.8, 133.8, 131.0, 131.0, 128.7, 128.4, 128.0, 128.0, 125.3, 125.2, 61.8, 51.6, 36.9, 14.3; **HRMS** (ESI): *m/z* $[M+H]^+$ calcd for $C_{19}H_{19}N_2O_4$: 339.13393, found: 339.13334.

ethyl 3-(11-methyl-6,12-dioxo-11,12-dihydrodibenzo[b,f][1,5]diazocin-5(6H)-yl)propanoate (**10b**): Yield 85 %, white crystals, mp 124.0-125.0 °C, Rf = 0.35 (hexane:ethyl acetate 1:1). **^{1}H NMR** (500 MHz, $CDCl_3$) δ 7.32 – 7.23 (m, 4H, H_{Ar}), 7.23 – 7.15 (m, 2H, H_{Ar}), 7.07 (dd, *J* = 17.2, 8.0 Hz, 2H, H_{Ar}), 4.75 (ddd, *J* = 13.6, 9.8, 6.1 Hz, 1H, H_{CH}), 4.09 (q, *J* = 7.1 Hz, 2H, H_{CH2}), 3.62 (ddd, *J* = 13.6, 9.8, 5.4 Hz, 1H, H_{CH}), 3.42 (s, 3H, , H_{CH3}), 2.70 (ddd, *J* = 16.1, 9.7, 6.1 Hz, 1H, H_{CH}), 2.53 (ddd, *J* = 16.4, 9.8, 5.4 Hz, 1H, H_{CH}), 1.20 (t, *J* = 7.2 Hz, 3H, H_{CH3}); **^{13}C NMR** (126 MHz, $CDCl_3$) δ 171.1, 168.2, 167.8, 140.4, 138.7, 135.5, 134.6, 131.0, 130.8, 128.7, 128.4, 127.6, 127.6, 125.9, 125.3, 60.7, 45.0, 36.7, 32.4, 14.2; **HRMS (ESI)**: *m/z* $[M+H]^+$ calcd for $C_{20}H_{21}N_2O_4$: 339.13393, found: 339.13333.

ethyl 4-(11-methyl-6,12-dioxo-11,12-dihydrodibenzo[b,f][1,5]diazocin-5(6H)-yl)butanoate (**10c**): Yield: 83 %, white crystals, mp 89.0-90.0 °C, Rf = 0.28 (hexane:ethyl acetate 1:1). **^{1}H NMR** (500 MHz, $CDCl_3$) δ 7.31 – 7.22 (m, 4H, H_{Ar}), 7.18 (qd, *J* = 7.6, 1.2 Hz, 2H, H_{Ar}), 7.09 – 7.03 (m, 2H, H_{Ar}), 4.48 (ddd, *J* = 13.4, 8.4, 7.3 Hz, 1H, H_{CH}), 4.09 (qd, *J* = 7.1, 1.4 Hz, 2H, H_{CH2}), 3.51 – 3.37 (m, 4H, H_{CH}+H_{CH3}), 2.34 (t, *J* = 7.2 Hz, 2H, H_{CH2}), 1.94 – 1.76 (m, 2H, H_{CH2}), 1.21 (t, *J* = 7.2 Hz, 3H, H_{CH3});
^{13}C NMR (126 MHz, $CDCl_3$) δ 172.8, 168.3, 168.0, 140.3, 138.9, 135.5, 134.8, 130.9, 130.7, 128.5, 128.4, 127.7, 127.5, 125.8, 125.2, 60.5, 48.3, 36.7, 31.4, 23.2, 14.3;
HRMS (ESI): *m/z* $[M+H]^+$ calcd for $C_{21}H_{23}N_2O_4$: 367.16523, found: 367.16461.

methyl 5-(11-methyl-6,12-dioxo-11,12-dihydrodibenzo[b,f][1,5]diazocin-5(6H)-yl)pentanoate (**10d**): Yield: 80 %, white crystals, mp 107.0-108.0 °C, Rf = 0.25 (hexane:ethyl acetate 1:1). **^{1}H NMR** (500 MHz, $CDCl_3$) δ 7.34 – 7.22 (m, 4H, H_{Ar}), 7.22 – 7.14 (m, 2H, H_{Ar}), 7.06 (t, *J* = 7.6 Hz, 2H, H_{Ar}), 4.51 (dt, *J* = 13.4, 7.7 Hz, 1H, H_{CH}), 3.62 (s, 3H, H_{CH3}), 3.43 (s, 3H, H_{CH3}), 3.32 (ddd, *J* = 13.1, 7.8, 5.2 Hz, 1H, H_{CH}), 2.44 – 2.24 (m, 2H, H_{CH2}), 1.75 – 1.44 (m, 4H, 2xH_{CH2}); **^{13}C NMR** (126 MHz, $CDCl_3$) δ 173.9, 168.4, 167.8, 140.4, 139.0, 135.6, 134.9, 130.9, 130.7, 128.5, 128.4, 127.7, 127.6, 125.9, 125.2, 51.6, 48.7, 36.7, 33.6, 27.3, 22.3; **HRMS (ESI)**: *m/z* $[M+H]^+$ calcd for $C_{21}H_{23}N_2O_4$: 367.16523, found: 367.16457.

methyl 6-(11-methyl-6,12-dioxo-11,12-dihydrodibenzo[b,f][1,5]diazocin-5(6H)-yl)hexanoate (**10e**): Yield: 76 %, white crystals, mp 95.0-96.0 °C, Rf = 0.27 (hexane:ethyl acetate 1:1). **^{1}H NMR** (500 MHz, $CDCl_3$) δ 7.32 – 7.21 (m, 4H, H_{Ar}), 7.18 (qd, *J* = 7.5, 1.2 Hz, 2H, H_{Ar}), 7.05 (dd, *J* = 7.9, 4.6 Hz, 2H, H_{Ar}), 4.49 (ddd, *J* = 13.4, 9.0, 7.1 Hz, 1H, H_{CH}), 3.62 (s, 3H, H_{CH3}), 3.42 (s, 3H, H_{CH3}), 3.29 (ddd, *J* = 13.7, 8.9, 5.0 Hz, 1H, H_{CH}), 2.27 (t, *J* = 7.5 Hz, 2H, H_{CH2}), 1.69 – 1.44 (m, 4H, 2xH_{CH2}), 1.39 – 1.26 (m, 2H, H_{CH2}); **^{13}C NMR** (126 MHz, $CDCl_3$) δ 174.1, 168.3, 167.7, 140.3, 139.1, 135.6, 134.9, 130.8, 130.6, 128.4, 128.3, 127.6, 127.6, 125.9, 125.2, 51.5, 49.0, 36.7, 34.0, 27.5, 26.4, 24.5; **HRMS (ESI)**: *m/z* $[M+H]^+$ calcd for $C_{22}H_{25}N_2O_4$: 381.18088, found: 381.18028.

ethyl 7-(11-methyl-6,12-dioxo-11,12-dihydrodibenzo[b,f][1,5]diazocin-5(6H)-yl)heptanoate (**10f**): Yield: 83%, pale oil, Rf = 0.41 (hexane:ethyl acetate 1:1). **^{1}H NMR** (500 MHz, $CDCl_3$) δ 7.32 – 7.22 (m, 4H, H_{Ar}), 7.18 (qd, *J* = 7.6, 1.2 Hz, 2H, H_{Ar}), 7.05 (t, *J* = 7.0 Hz, 2H, H_{Ar}), 4.48 (ddd, *J* = 13.3, 9.1, 7.1 Hz, 1H, H_{CH}), 4.09 (q, *J* = 7.1 Hz, 2H, H_{CH2}), 3.43 (s, 3H, H_{CH3}), 3.28 (ddd, *J* = 13.6, 9.1, 5.0 Hz, 1H, H_{CH}), 2.25 (t, *J* = 7.6 Hz, 2H, H_{CH2}), 1.63 – 1.43 (m, 4H, 2xH_{CH2}), 1.37 – 1.27 (m, 4H, 2xH_{CH2}), 1.22 (t, *J* = 7.1 Hz, 3H, H_{CH3}); **^{13}C NMR** (126 MHz, $CDCl_3$) δ 173.9, 168.4, 167.8, 140.4, 139.2, 135.6, 135.0, 130.9, 130.7, 128.4, 128.4, 127.7, 127.6, 125.9, 125.2, 60.3, 49.2, 36.7, 34.4, 28.9, 27.7, 26.7, 25.1, 14.4; **HRMS (ESI)**: *m/z* $[M+H]^+$ calcd for $C_{24}H_{29}N_2O_4$: 409.21218, found: 409.21156.

ethyl 2-(6-oxodibenzo[b,f]azocin-5(6H)-yl)acetate (**10g**): Yield: 90 %, white crystals, mp 86.0-87.0 °C, Rf = 0.78 (hexane:ethyl acetate 1:1). **^{1}H NMR** (500 MHz, $CDCl_3$) δ 7.45 – 7.33 (m, 2H,

$H_{CH=CH}$), 7.26 – 7.11 (m, 4H, H_{Ar}), 7.11 – 7.04 (m, 1H, H_{Ar}), 7.03 – 6.93 (m, 2H, H_{Ar}), 6.89 (d, *J* = 11.5 Hz, 1H, H_{Ar}), 4.57 (d, *J* = 17.0 Hz, 1H, H_{CH}), 4.34 – 4.18 (m, 3H, H_{CH}+H_{CH2}), 1.30 (t, *J* = 7.2 Hz, 3H, H_{CH3});
^{13}C NMR (126 MHz, $CDCl_3$) δ 171.5, 169.0, 141.7, 136.4, 135.4, 133.6, 132.8, 129.9, 129.0, 128.8, 128.7, 127.6, 127.6, 127.5, 127.3, 126.1, 61.4, 52.5, 14.3;
HRMS (ESI): *m/z* $[M+H]^+$ calcd for $C_{19}H_{18}NO_3$: 308.12812, found: 308.12810.

methyl 3-(6-oxodibenzo[b,f]azocin-5(6H)-yl)propanoate (**10h**): Yield: 90 %, white crystals, mp 89.0-90.0 °C, Rf = 0.57 (hexane:ethyl acetate 1:1). **^{1}H NMR** (500 MHz, $CDCl_3$) δ 7.39 – 7.31 (m, 1H, H_{Ar}), 7.25 – 7.12 (m, 5H, H_{Ar}), 7.07 (d, *J* = 7.7 Hz, 1H, H_{Ar}), 7.00 – 6.90 (m, 2H, H_{Ar}), 6.82 (d, *J* = 11.5 Hz, 1H, H_{Ar}), 4.74 (ddd, *J* = 13.5, 8.7, 5.9 Hz, 1H, H_{CH}), 3.64 – 3.50 (m, 4H, H_{CH}+H_{CH3}), 2.67 (ddd, *J* = 16.2, 8.6, 5.9 Hz, 1H, H_{CH}), 2.51 (ddd, *J* = 16.2, 8.7, 6.4 Hz, 1H, H_{CH}); **^{13}C NMR** (126 MHz, $CDCl_3$) δ 171.8, 171.0, 140.9, 137.0, 136.1, 133.5, 133.3, 129.5, 128.9, 128.8, 128.6, 127.7, 127.6, 127.3, 127.2, 127.0, 51.8, 46.5, 32.8; **HRMS (ESI)**: *m/z* $[M+H]^+$ calcd for $C_{19}H_{18}NO_3$: 308.12812, found: 308.12808.

ethyl 4-(6-oxodibenzo[b,f]azocin-5(6H)-yl)butanoate (**10i**): Yield: 77 %, white crystals, mp 90.0-91.0 °C, Rf = 0.68 (hexane:ethyl acetate 1:1). **^{1}H NMR** (500 MHz, $CDCl_3$) δ 7.39 – 7.31 (m, 1H, H_{Ar}), 7.23 – 7.10 (m, 5H, H_{Ar}), 7.07 (d, *J* = 7.6 Hz, 1H, H_{Ar}), 7.01 – 6.92 (m, 2H, H_{Ar}), 6.87 (d, *J* = 11.5 Hz, 1H, H_{Ar}), 4.49 (dt, *J* = 13.3, 7.7 Hz, 1H, H_{CH}), 4.08 (q, *J* = 7.1 Hz, 2H, H_{CH2}), 3.31 (ddd, *J* = 13.4, 7.7, 5.9 Hz, 1H, H_{CH}), 2.47 – 2.24 (m, 2H, H_{CH2}), 1.91 – 1.69 (m, 2H, H_{CH2}), 1.20 (t, *J* = 7.1 Hz, 3H, H_{CH3}); **^{13}C NMR** (126 MHz, $CDCl_3$) δ 173.1, 171.1, 141.1, 137.3, 136.1, 133.4, 133.4, 129.6, 128.7, 128.7, 128.6, 127.6, 127.6, 127.2, 127.1, 126.8, 60.4, 49.7, 31.8, 23.2, 14.3; **HRMS (ESI)**: *m/z* $[M+H]^+$ calcd for $C_{21}H_{22}NO_3$: 336.15942, found: 336.15944.

methyl 5-(6-oxodibenzo[b,f]azocin-5(6H)-yl)pentanoate (**10j**): Yield: 91 %, white crystals, m.p 99.0-100.0 °C, Rf = 0.60 (hexane:ethyl acetate 1:1). **^{1}H NMR** (500 MHz, $CDCl_3$) δ 7.39 – 7.31 (m, 1H, H_{Ar}), 7.25 – 7.10 (m, 5H, H_{Ar}), 7.08 (d, *J* = 7.6 Hz, 1H, H_{Ar}), 7.01 – 6.91 (m, 2H, H_{Ar}), 6.86 (d, *J* = 11.4 Hz, 1H, H_{Ar}), 4.47 (ddd, *J* = 13.3, 8.5, 6.8 Hz, 1H, H_{CH}), 3.63 (s, 3H, H_{CH3}), 3.26 (ddd, *J* = 13.5, 8.4, 5.3 Hz, 1H, H_{CH}), 2.29 (td, *J* = 7.5, 1.4 Hz, 2H, H_{CH2}), 1.73 – 1.56 (m, 2H, H_{CH2}), 1.56 – 1.39 (m, 2H, H_{CH2}); **^{13}C NMR** (126 MHz, $CDCl_3$) δ 173.9, 171.0, 141.2, 137.4, 136.2, 133.5, 133.3, 129.6, 128.7, 128.6, 128.6, 127.6, 127.6, 127.2, 127.0, 126.9, 51.6, 50.1, 33.8, 27.3, 22.4; **HRMS (ESI)**: *m/z* $[M+H]^+$ calcd for $C_{21}H_{22}NO_3$: 336.15942, found: 336.15931.

methyl 6-(6-oxodibenzo[b,f]azocin-5(6H)-yl)hexanoate (**10k**): Yield: 82 %, pale oil, Rf = 0.64 (hexane:ethyl acetate 1:1). **^{1}H NMR** (500 MHz, $CDCl_3$) δ 7.38 – 7.31 (m, 1H, H_{Ar}), 7.23 – 7.10 (m, 5H, H_{Ar}), 7.07 (d, *J* = 7.6 Hz, 1H, H_{Ar}), 7.01 – 6.90 (m, 2H, H_{Ar}), 6.84 (d, *J* = 11.4 Hz, 1H, H_{Ar}), 4.45 (ddd, *J* = 13.3, 8.7, 6.7 Hz, 1H, H_{CH}), 3.63 (s, 3H, H_{CH3}), 3.22 (ddd, *J* = 13.6, 9.0, 4.8 Hz, 1H, H_{CH}), 2.26 (t, *J* = 7.5 Hz, 2H, H_{CH2}), 1.69 – 1.54 (m, 2H, H_{CH2}), 1.54 – 1.45 (m, 1H, H_{CH}), 1.45 – 1.32 (m, 2H, H_{CH2}), 1.31 – 1.21 (m, 1H, H_{CH}); **^{13}C NMR** (126 MHz, $CDCl_3$) δ 174.1, 170.9,

141.2, 137.4, 136.2, 133.4, 133.2, 129.6, 128.6, 128.6, 128.5, 127.6, 127.5, 127.2, 127.0, 126.9, 51.6, 50.4, 34.0, 27.6, 26.6, 24.7; **HRMS (ESI)**: *m/z* $[M+H]^+$ calcd for $C_{22}H_{24}NO_3$: 350.17507, found: 350.17502.

ethyl 7-(6-oxodibenzo[b,f]azocin-5(6H)-yl)heptanoate (**10l**): Yield: 90 %, pale oil, Rf = 0.78 (hexane:ethyl acetate 1:1). **^{1}H NMR** (500 MHz, $CDCl_3$) δ 7.40 – 7.30 (m, 1H, H_{Ar}), 7.24 – 7.10 (m, 5H, H_{Ar}), 7.07 (d, *J* = 7.6 Hz, 1H, H_{Ar}), 7.01 – 6.91 (m, 2H, H_{Ar}), 6.84 (d, *J* = 11.4 Hz, 1H, H_{Ar}), 4.45 (ddd, *J* = 13.2, 8.8, 6.6 Hz, 1H, H_{CH}), 4.10 (q, *J* = 7.1 Hz, 2H, H_{CH2}), 3.22 (ddd, *J* = 13.6, 9.1, 4.8 Hz, 1H, H_{CH2}), 2.25 (t, *J* = 7.5 Hz, 2H, H_{CH2}), 1.57 (p, *J* = 7.4 Hz, 2H, H_{CH2}), 1.53 – 1.44 (m, 1H, H_{CH}), 1.44 – 1.32 (m, 2H, H_{CH2}), 1.31 – 1.21 (m, 6H, H_{CH}+H_{CH2}+H_{CH3}); **^{13}C NMR** (126 MHz, $CDCl_3$) δ 173.9, 170.9, 141.3, 137.5, 136.2, 133.4, 133.3, 129.6, 128.6, 128.6, 128.5, 127.6, 127.6, 127.2, 127.0, 126.9, 60.3, 50.5, 34.3, 29.0, 27.8, 26.7, 25.0, 14.4; **HRMS (ESI)**: *m/z* $[M+H]^+$ calcd for $C_{24}H_{28}N_3O$: 378.20637, found: 378.20618.

methyl 6-(12-methyl-6,11-dioxo-11,12-dihydrodibenzo[b,f][1,4]diazocin-5(6H)-yl)hexanoate (**10m**): Yield: 88 %, white crystals, mp 169.0-170.0 °C, Rf = 0.29 (hexane:ethyl acetate 1:1). **^{1}H NMR** (500 MHz, $CDCl_3$) δ 7.26 – 7.21 (m, 4H, H_{Ar}), 7.19 – 7.13 (m, 4H, H_{Ar}), 4.46 (ddd, *J* = 13.4, 9.5, 6.4 Hz, 1H, H_{CH}), 3.60 (s, 3H, H_{CH3}), 3.39 (s, 3H, H_{CH3}), 3.29 (ddd, *J* = 13.4, 9.8, 4.6 Hz, 1H, H_{CH}), 2.26 (t, *J* = 7.4 Hz, 2H, H_{CH2}), 1.69 – 1.49 (m, 3H, H_{CH2}+H_{CH}), 1.48 – 1.25 (m, 3H, H_{CH2}+H_{CH}); **^{13}C NMR** (126 MHz, $CDCl_3$) δ 173.9, 169.7, 169.1, 141.8, 139.5, 133.2, 132.6, 130.2, 130.0, 128.9, 128.8, 127.4, 126.7, 126.5, 126.5, 51.5, 48.9, 36.8, 33.9, 28.1, 26.3, 24.6; **HRMS (ESI)**: *m/z* $[M+H]^+$ calcd for $C_{22}H_{25}N_2O_4$: 381.18088, found: 381.18032.

methyl 6-(8,9-dichloro-12-methyl-6,11-dioxo-11,12-dihydrodibenzo[b,f][1,4]diazocin-5(6H)-yl) hexanoate (**10n**): Yield: 83 %, white crystals, mp 158.0-159.0 °C, Rf = 0.55 (hexane:ethyl acetate 1:1). **^{1}H NMR** (500 MHz, $CDCl_3$) δ 7.35 (d, *J* = 6.2 Hz, 2H, H_{Ar}), 7.29 – 7.17 (m, 4H, H_{Ar}), 4.43 (ddd, *J* = 13.5, 9.6, 6.3 Hz, 1H, H_{CH}), 3.62 (s, 3H, H_{CH3}), 3.39 (s, 3H, H_{CH3}), 3.30 (ddd, *J* = 14.1, 9.8, 4.6 Hz, 1H, H_{CH}), 2.27 (t, *J* = 7.4 Hz, 2H, H_{CH2}), 1.68 – 1.48 (m, 3H, H_{CH2}+H_{CH}), 1.48 – 1.25 (m, 3H, H_{CH2}+H_{CH}); **^{13}C NMR** (126 MHz, $CDCl_3$) δ 173.9, 167.5, 166.9, 141.3, 139.0, 134.7, 134.5, 132.8, 132.3, 129.4, 129.4, 129.0, 128.7, 127.6, 126.8, 51.6, 49.1, 37.0, 33.9, 28.0, 26.3, 24.6; **HRMS (ESI)**: *m/z* $[M+H]^+$ calcd for $C_{22}H_{23}Cl_2N_2O_4$: 449.10294, 451.09999, found: 449.10243, 451.09958.

methyl 6-(12-benzyl-6,11-dioxo-11,12-dihydrodibenzo[b,f][1,4]diazocin-5(6H)-yl)hexanoate (**10o**): Yield: 78 %, white crystals, mp 148.0-149.0 °C, Rf = 0.48 (hexane:ethyl acetate 1:1). **^{1}H NMR** (500 MHz, $CDCl_3$) δ 7.35 – 7.20 (m, 9H, H_{Ar}), 7.18 – 7.04 (m, 4H, H_{Ar}), 5.21 (d, *J* = 14.3 Hz, 1H, H_{Bn}), 4.88 (d, *J* = 14.3 Hz, 1H, H_{Bn}), 3.65 (s, 3H, H_{CH3}), 3.38 (ddd, *J* = 13.5, 10.7, 5.2 Hz, 1H, H_{CH}), 2.87 (ddd, *J* = 13.6, 10.7, 5.5 Hz, 1H, H_{CH}), 2.29 (t, *J* = 7.5 Hz, 2H, H_{CH2}), 1.65 – 1.52 (m, 3H, H_{CH2}+H_{CH}), 1.49 – 1.35 (m, 1H, H_{CH}), 1.32 – 1.14 (m, 2H, H_{CH2}); **^{13}C NMR** (126 MHz, $CDCl_3$) δ 174.0, 169.5, 169.1, 140.8, 140.0, 137.1, 133.3, 132.7, 130.2, 130.0, 129.14 (2xC), 128.9, 128.9

(2xC), 128.7, 128.0, 127.6, 127.4, 126.5, 126.5, 52.7, 51.6, 49.0, 34.0, 28.1, 26.5, 24.6; **HRMS (ESI)**: *m/z* $[M+H]^+$ calcd for $C_{28}H_{29}N_2O_4$: 457.21218, found: 457.21159.

methyl 6-(6-oxo-11,12-dihydrodibenzo[b,f]azocin-5(6H)-yl)hexanoate (**10p**): Yield: 85 %, white crystals, mp 77.0-78.0 °C, Rf = 0.75 (hexane:ethyl acetate 1:1). **^{1}H NMR** (500 MHz, $CDCl_3$) δ 7.15 (dd, *J* = 7.2, 1.7 Hz, 1H, H_{Ar}), 7.08 – 6.98 (m, 6H, H_{Ar}), 6.87 (d, *J* = 7.1 Hz, 1H, H_{Ar}), 4.17 (ddd, *J* = 13.2, 10.4, 5.6 Hz, 1H, H_{CH}), 3.63 (s, 3H, H_{CH3}), 3.52 (ddd, *J* = 13.2, 10.3, 5.3 Hz, 1H, H_{CH}), 3.43 – 3.34 (m, 1H, H_{CH}), 3.31 – 3.22 (m, 1H, H_{CH}), 2.95 – 2.83 (m, 2H, H_{CH2}), 2.29 (t, *J* = 7.5 Hz, 2H, H_{CH2}), 1.78 – 1.68 (m, 1H, H_{CH}), 1.64 (p, *J* = 7.6 Hz, 2H, H_{CH2}), 1.61 – 1.52 (m, 1H, H_{CH}), 1.45 – 1.31 (m, 2H, H_{CH2}); **^{13}C NMR** (126 MHz, $CDCl_3$) δ 174.0, 172.1, 142.2, 138.2, 137.8, 135.4, 130.3, 129.1, 129.0, 127.9, 127.5, 127.0, 126.7, 126.3, 51.5, 49.2, 34.0, 33.0, 30.8, 27.6, 26.7, 24.7; **HRMS (ESI)**: *m/z* $[M+H]^+$ calcd for $C_{22}H_{26}NO_3$: 352.19072, found: 352.19024.

methyl 6-(2-bromo-11-methyl-6,12-dioxo-11,12-dihydrodibenzo[b,f][1,5]diazocin-5(6H)-yl)hexanoate (**10r**): Yield: 87 %, pale oil, Rf = 0.42 (hexane:ethyl acetate 1:1). **^{1}H NMR** (500 MHz, $CDCl_3$) δ 7.43 (d, *J* = 2.3 Hz, 1H, H_{Ar}), 7.39 (dd, *J* = 8.5, 2.3 Hz, 1H, H_{Ar}), 7.35 – 7.18 (m, 3H, H_{Ar}), 7.08 (d, *J* = 8.0 Hz, 1H, H_{Ar}), 6.95 (d, *J* = 8.5 Hz, 1H, H_{Ar}), 4.49 (ddd, *J* = 13.4, 9.0, 7.1 Hz, 1H, H_{CH}), 3.64 (s, 3H, H_{CH3}), 3.42 (s, 3H, H_{CH3}), 3.25 (ddd, *J* = 13.7, 8.9, 5.0 Hz, 1H, H_{CH}), 2.29 (t, *J* = 7.5 Hz, 2H, H_{CH2}), 1.69 – 1.59 (m, 2H, H_{CH2}), 1.59 – 1.45 (m, 2H, H_{CH2}), 1.38 – 1.28 (m, 2H, H_{CH2}); **^{13}C NMR** (126 MHz, $CDCl_3$) δ 174.1, 167.5, 166.8, 140.0, 138.2, 137.3, 134.7, 134.0, 131.0, 130.7, 128.7, 127.7, 127.6, 125.4, 122.0, 51.6, 49.1, 36.8, 34.1, 27.5, 26.4, 24.6; **HRMS (ESI)**: *m/z* $[M+H]^+$ calcd for $C_{22}H_{24}BrN_2O_4$: 459.09140, 461.08935, found: 459.09106, 461.08887.

methyl 6-(2,3-dimethoxy-11-methyl-6,12-dioxo-11,12-dihydrodibenzo[b,f][1,5]diazocin-5(6H)-yl)hexanoate (**10s**): Yield: 74 %, white crystals, mp 115.0-116.0 °C, Rf = 0.15 (hexane:ethyl acetate 1:1). **^{1}H NMR** (500 MHz, $CDCl_3$) δ 7.31 – 7.21 (m, 2H, H_{Ar}), 7.18 (td, *J* = 7.5, 1.2 Hz, 1H, H_{Ar}), 7.02 (d, *J* = 8.0 Hz, 1H, H_{Ar}), 6.72 (s, 1H, H_{Ar}), 6.45 (s, 1H, H_{Ar}), 4.48 (ddd, *J* = 13.3, 8.8, 7.2 Hz, 1H, H_{CH}), 3.76 (d, *J* = 2.4 Hz, 6H, H_{OCH3}), 3.60 (s, 3H, H_{CH3}), 3.38 (s, 3H, H_{CH3}), 3.17 (ddd, *J* = 13.5, 8.8, 4.9 Hz, 1H, H_{CH}), 2.26 (t, *J* = 7.5 Hz, 2H, H_{CH2}), 1.66 – 1.56 (m, 2H, H_{CH2}), 1.55 – 1.41 (m, 2H, H_{CH2}), 1.36 – 1.25 (m, 2H, H_{CH2}); **^{13}C NMR** (126 MHz, $CDCl_3$) δ 174.1, 168.3, 167.9, 150.5, 148.6, 140.6, 134.8, 132.1, 130.6, 128.2, 127.6, 127.5, 125.1, 109.3, 108.4, 56.2, 56.0, 51.5, 48.9, 36.8, 34.0, 27.4, 26.4, 24.6; **HRMS (ESI)**: *m/z* $[M+H]^+$ calcd for $C_{24}H_{29}N_2O_6$: 441.20201, found: 441.20158.

methyl 6-(5-methyl-6,14-dioxo-5,14-dihydrobenzonaphtho[2,3-f][1,5]diazocin-13(6H)-yl)hexanoate (**10t**): Yield: 69 %, white crystals, mp 198.0-199.0 °C, Rf = 0.43 (hexane:ethyl acetate 1:1). **^{1}H NMR** (500 MHz, $CDCl_3$) δ 7.84 (s, 1H, H_{Ar}), 7.82 – 7.70 (m, 2H, H_{Ar}), 7.57 (s, 1H, H_{Ar}), 7.50 (pd, *J* = 6.9, 1.4 Hz, 2H, H_{Ar}), 7.27 (d, *J* = 2.4 Hz, 1H, H_{Ar}), 7.16 (dd, *J* = 8.6, 2.4 Hz, 1H, H_{Ar}), 7.01 (d, *J* = 8.6 Hz, 1H, H_{Ar}), 4.53 (ddd, *J* = 13.3, 9.1, 6.9 Hz, 1H, H_{CH}), 3.63 (s, 3H, H_{CH3}), 3.50 – 3.40 (m, 4H, H_{CH3}+H_{CH}), 2.28 (t, *J* = 7.5 Hz, 2H, H_{CH2}), 1.67 – 1.49 (m, 4H, 2xH_{CH2}), 1.42 – 1.28 (m, 2H,

H_{CH2}); **^{13}C NMR** (126 MHz, $CDCl_3$) δ 174.1, 168.3, 166.6, 138.6, 136.9, 136.5, 134.2, 133.8, 133.7, 132.2, 130.9, 128.3, 128.1, 128.0, 127.8, 127.6, 127.5, 126.9, 125.2, 51.6, 50.1, 36.9, 34.1, 27.6, 26.5, 24.6; **HRMS (ESI)**: *m/z* $[M+H]^+$ calcd for $C_{26}H_{26}ClN_2O_4$: 465.15756, 467.15461 found: 465.15716, 467.15450.

2.2 General procedure for synthesis of hydroxamic acids

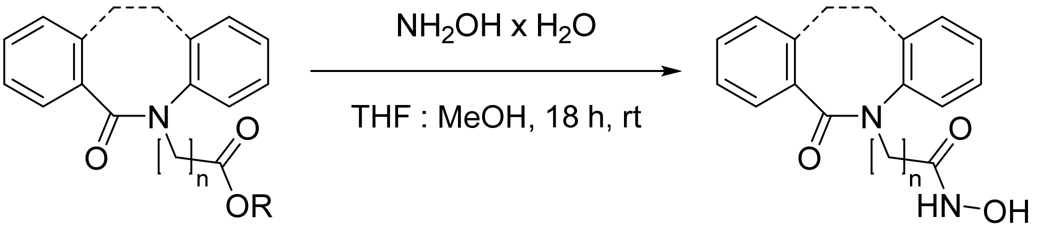

Ester from previous step was dissolved in mixture of THF (2 mL/mmol) and methanol (1 mL/mmol), then aqueous hydroxylamine solution (50% wt.; 2 ml/mmol) was added, reaction mixture was stirred for 18 hrs at room temperature. Volatiles were evaporated and resulting solid was dissolved in small amount of methanol, then small amount of chromatographic gel was added. Volatiles were evaporated, resulting solid was placed on top of chromatographic column and eluted with mixture of chloroform and methanol (1% to 10% of CH_3OH in $CHCl_3$).

N-hydroxy-2-(11-methyl-6,12-dioxo-11,12-dihydrodibenzo[b,f][1,5]diazocin-5(6H)-yl)acetamide (**7a**): Yield: 38 %, white crystals, mp 233.0-234.0 °C, Rf = 0.35 (methanol:chloroform 1:9). **^{1}H NMR** (500 MHz, CD_3OD) δ 7.41 – 7.25 (m, 8H, H_{Ar}), 4.57 (d, *J* = 15.7 Hz, 1H, H_{CH}), 4.16 (d, *J* = 15.7 Hz, 1H, H_{CH}), 3.44 (s, 3H, H_{CH3}); **^{13}C NMR** (126 MHz, CD_3OD) δ 170.5, 170.3, 167.3, 141.5, 140.8, 135.7, 134.9, 132.4, 132.3, 129.8, 129.6, 128.7, 128.6, 127.1, 126.6, 51.1, 37.0; **HRMS (ESI)**: *m/z* $[M+H]^+$ calcd for $C_{17}H_{16}N_3O_4$: 326.11353, found: 326.11332.

N-hydroxy-3-(11-methyl-6,12-dioxo-11,12-dihydrodibenzo[b,f][1,5]diazocin-5(6H)-yl)propanamide (**7b**): Yield: 55 %, white crystals, mp 105.0-106.0 °C, Rf = 0.54 (methanol:chloroform 1:9**)**. **^{1}H NMR** (500 MHz, CD_3OD) δ 7.42 – 7.34 (m, 2H, H_{Ar}), 7.32 – 7.25 (m, 6H, H_{Ar}), 4.70 (ddd, *J* = 13.6, 9.5, 6.0 Hz, 1H, H_{CH}), 3.71 (ddd, *J* = 13.5, 9.4, 5.8 Hz, 1H, H_{CH}), 3.43 (s, 3H, H_{CH3}), 2.45 (ddd, *J* = 14.2, 9.4, 6.0 Hz, 1H, H_{CH}), 2.32 (ddd, *J* = 14.2, 9.5, 5.9 Hz, 1H, H_{CH}); **^{13}C NMR** (126 MHz, CD_3OD) δ 170.5, 169.9, 169.6, 141.4, 139.8, 136.3, 135.5, 132.4, 132.2, 129.9, 129.7, 128.4, 128.3, 127.3, 126.6, 46.8, 37.0, 32.1; **HRMS (ESI)**: *m/z* $[M+H]^+$ calcd for $C_{18}H_{18}N_3O_4$: 340.12918, found: 340.12909.

N-hydroxy-4-(11-methyl-6,12-dioxo-11,12-dihydrodibenzo[b,f][1,5]diazocin-5(6H)-yl)butanamide (**7c**): Yield: 52 %, white crystals, mp 190.0-191.0 °C, Rf = 0.42 (methanol:chloroform 1:9). **^{1}H NMR** (500 MHz, CD_3OD) δ 7.41 – 7.33 (m, 2H, H_{Ar}), 7.31 – 7.25 (m, 6H, H_{Ar}), 4.54 – 4.40 (m,

1H, H_{CH}), 3.50 (ddd, *J* = 13.6, 8.2, 5.4 Hz, 1H, H_{CH}), 3.45 (s, 3H, H_{CH3}), 2.13 (t, *J* = 7.5 Hz, 2H, H_{CH2}), 1.94 – 1.84 (m, 1H, H_{CH}), 1.83 – 1.74 (m, 1H, H_{CH}); **^{13}C NMR** (126 MHz, CD_3OD) δ 171.9, 170.5, 170.2, 141.4, 140.0, 136.4, 135.7, 132.4, 132.2, 129.8, 129.7, 128.4, 128.3, 127.2, 126.6, 49.6, 36.9, 31.1, 25.1; **HRMS (ESI)**: *m/z* $[M+H]^+$ calcd for $C_{19}H_{20}N_3O_4$: 354.14483, found: 354.14478.

N-hydroxy-5-(11-methyl-6,12-dioxo-11,12-dihydrodibenzo[b,f][1,5]diazocin-5(6H)-yl)pentanamide (**7d**): Yield: 60 %, white crystals, mp 217.0-218.0 °C, Rf = 0.42 (methanol:chloroform 1:9). **^{1}H NMR** (500 MHz, CD_3OD) δ 7.43 – 7.33 (m, 2H, H_{Ar}), 7.32 – 7.22 (m, 6H, H_{Ar}), 4.53 (dt, *J* = 13.5, 7.7 Hz, 1H, H_{CH}), 3.46 – 3.39 (m, 4H, H_{CH} + H_{CH3}), 2.10 (hept, *J* = 7.2 Hz, 2H, H_{CH2}), 1.64 (p, *J* = 7.5 Hz, 2H, H_{CH2}), 1.57 – 1.47 (m, 2H, H_{CH2}); **^{13}C NMR** (126 MHz, DMSO) δ 168.9, 167.3, 166.8, 140.0, 138.5, 135.3, 134.6, 130.8, 130.6, 128.2, 128.1, 127.2, 127.0, 126.1, 125.5, 47.7, 36.0, 31.8, 26.9, 22.4; **HRMS (ESI)**: *m/z* $[M+H]^+$ calcd for $C_{20}H_{22}N_3O_4$: 368.16048, found: 368.16052.

N-hydroxy-6-(11-methyl-6,12-dioxo-11,12-dihydrodibenzo[b,f][1,5]diazocin-5(6H)-yl)hexanamide (**7e**): Yield: 57 %, white crystals, mp 155.0-156.0 °C, Rf = 0.43 (methanol:chloroform 1:9). **^{1}H NMR** (500 MHz, CD_3OD) δ 7.41 – 7.33 (m, 2H, H_{Ar}), 7.31 – 7.24 (m, 6H, H_{Ar}), 4.53 (dt, *J* = 13.5, 7.8 Hz, 1H, H_{CH}), 3.44 (s, 3H, H_{CH3}), 3.39 (ddd, *J* = 13.2, 7.2, 5.7 Hz, 1H, H_{CH}), 2.08 (t, *J* = 7.4 Hz, 2H, H_{CH2}), 1.71 – 1.58 (m, 2H, H_{CH2}), 1.57 – 1.47 (m, 2H, H_{CH2}), 1.43 – 1.28 (m, 2H, H_{CH2}); **^{13}C NMR** (126 MHz, CD_3OD) δ 172.8, 170.4, 170.0, 141.4, 140.1, 136.4, 135.7, 132.4, 132.1, 129.7, 129.6, 128.4, 128.3, 127.2, 126.6, 50.0, 36.9, 33.6, 28.5, 27.4, 26.3; **HRMS (ESI)**: *m/z* $[M+H]^+$ calcd for $C_{21}H_{24}N_3O_4$: 382.17613, found: 382.17611.

N-hydroxy-7-(11-methyl-6,12-dioxo-11,12-dihydrodibenzo[b,f][1,5]diazocin-5(6H)-yl)heptanamide (**7f**): Yield: 31 %, white crystals, mp 145.0-146.0 °C, Rf = 0.45 (methanol:chloroform 1:9). **^{1}H NMR** (500 MHz, CD_3OD) δ 7.41 – 7.33 (m, 2H, H_{Ar}), 7.31 – 7.24 (m, 6H, H_{Ar}), 4.54 (dt, *J* = 13.5, 7.8 Hz, 1H, H_{CH}), 3.43 (s, 3H, H_{CH3}), 3.39 (dt, *J* = 13.2, 6.4 Hz, 1H, H_{CH}), 2.08 (t, *J* = 7.4 Hz, 2H, H_{CH2}), 1.66 – 1.56 (m, 2H, H_{CH2}), 1.54 – 1.45 (m, 2H, H_{CH2}), 1.40 – 1.29 (m, 4H, 2xH_{CH2}); **^{13}C NMR** (126 MHz, CD_3OD) δ 172.9, 170.4, 170.0, 141.4, 140.1, 136.5, 135.8, 132.3, 132.1, 129.6, 129.6, 128.5, 128.3, 127.2, 126.6, 50.1, 36.9, 33.7, 29.7, 28.7, 27.6, 26.7; **HRMS (ESI)**: *m/z* $[M+H]^+$ calcd for $C_{22}H_{26}N_3O_4$: 396.19178, found: 396.19180.

N-hydroxy-2-(6-oxodibenzo[b,f]azocin-5(6H)-yl)acetamide (**7g**): Yield: 69 %, white crystals, mp 119.0-120.0 °C, Rf = 0.47 (methanol:chloroform 1:9). **^{1}H NMR** (500 MHz, CD_3OD) δ 7.33 – 6.90 (m, 10H, H_{Ar} + $H_{CH=CH}$), 4.45 (d, *J* = 15.5 Hz, 1H, H_{CH}), 4.12 (d, *J* = 15.5 Hz, 1H, H_{CH}); **^{13}C NMR** (126 MHz, CD_3OD) δ 173.8, 167.6, 142.7, 137.5, 136.6, 135.1, 133.6, 131.4, 130.3, 129.8, 129.7, 128.6, 128.6, 128.6, 128.1, 127.6, 52.1; **HRMS (ESI)**: *m/z* $[M+H]^+$ calcd for $C_{17}H_{15}N_2O_3$: 295.10772, found: 295.10774.

N-hydroxy-3-(6-oxodibenzo[b,f]azocin-5(6H)-yl)propanamide (**7h**): Yield: 66 %, white crystals, mp 173.0-174.0 °C, Rf = 0.47 (methanol:chloroform 1:9). **^{1}H NMR** (500 MHz, CD_3OD) δ 7.37 – 6.91

(m, 10H, H_{Ar} + $H_{CH=CH}$), 4.57 (ddd, *J* = 13.8, 8.4, 5.8 Hz, 1H, H_{CH}), 3.65 (ddd, *J* = 14.0, 8.2, 6.5 Hz, 1H, H_{CH}), 2.39 (qdd, *J* = 14.6, 8.3, 6.3 Hz, 2H, H_{CH2}); **^{13}C NMR** (126 MHz, CD_3OD) δ 173.4, 169.8, 142.2, 138.0, 137.2, 135.1, 133.8, 131.3, 130.1, 129.9, 129.7, 128.7, 128.6, 128.5, 127.8 (2xC), 48.4, 32.1; **HRMS (ESI)**: *m/z* $[M+H]^+$ calcd for $C_{18}H_{17}N_2O_3$: 309.12337, found: 309.12329.

N-hydroxy-4-(6-oxodibenzo[b,f]azocin-5(6H)-yl)butanamide (**7i**): Yield: 71 %, white crystals, mp 172.0-173.0 °C, Rf = 0.48 (methanol:chloroform 1:9). **^{1}H NMR** (500 MHz, CD_3OD) δ 7.33 – 6.96 (m, 10H, H_{Ar} + $H_{CH=CH}$), 4.40 (dt, *J* = 13.4, 7.8 Hz, 1H, H_{CH}), 3.39 (dt, *J* = 13.5, 6.8 Hz, 1H, H_{CH}), 2.14 (qt, *J* = 14.6, 7.6 Hz, 2H, H_{CH2}), 1.77 (p, *J* = 7.4 Hz, 2H, H_{CH2}); **^{13}C NMR** (126 MHz, CD_3OD) δ 173.5, 172.0, 141.9, 138.2, 137.4, 135.0, 134.0, 131.3, 130.1, 129.9, 129.7, 128.7, 128.6, 128.5, 127.8, 127.7, 51.0, 31.3, 24.9; **HRMS (ESI)**: *m/z* $[M+H]^+$ calcd for $C_{19}H_{19}N_2O_3$: 323.13902, found: 323.13893.

N-hydroxy-5-(6-oxodibenzo[b,f]azocin-5(6H)-yl)pentanamide (**7j**): Yield: 59 %, white crystals, mp 175.0-176.0 °C, Rf = 0.48 (methanol:chloroform 1:9). **^{1}H NMR** (500 MHz, CD_3OD) δ 7.35 – 6.90 (m, 10H, H_{Ar} + $H_{CH=CH}$), 4.43 (dt, *J* = 13.4, 7.6 Hz, 1H, H_{CH}), 3.35 (ddd, *J* = 13.5, 7.5, 5.9 Hz, 1H, H_{CH}), 2.08 (td, *J* = 7.2, 2.9 Hz, 2H, H_{CH2}), 1.75 – 1.55 (m, 2H, H_{CH2}), 1.53 – 1.43 (m, 2H, H_{CH2}); **^{13}C NMR** (126 MHz, CD_3OD) δ 173.4, 172.5, 142.1, 138.3, 137.4, 135.0, 133.9, 131.4, 130.0, 129.9, 129.7, 128.7, 128.6, 128.4, 127.8, 127.7, 51.2, 33.4, 28.3, 24.3; **HRMS (ESI)**: *m/z* $[M+H]^+$ calcd for $C_{20}H_{21}N_2O_3$: 337.15467, found: 337.15448.

N-hydroxy-6-(6-oxodibenzo[b,f]azocin-5(6H)-yl)hexanamide (**7k**): Yield: 63 %, white crystals, mp 180.0-181.0 °C, Rf = 0.47 (methanol:chloroform 1:9). **^{1}H NMR** (500 MHz, CD_3OD) δ 7.32 – 6.95 (m, 10H, H_{Ar} + $H_{CH=CH}$), 4.43 (dt, *J* = 13.4, 7.6 Hz, 1H, H_{CH}), 3.40 – 3.32 (m, 1H, H_{CH}), 2.06 (td, *J* = 7.3, 1.3 Hz, 2H, H_{CH2}), 1.66 – 1.52 (m, 2H, H_{CH2}), 1.52 – 1.42 (m, 2H, H_{CH2}), 1.42 – 1.26 (m, 2H, H_{CH2}); **^{13}C NMR** (126 MHz, CD_3OD) δ 173.4, 172.8, 142.1, 138.4, 137.4, 135.0, 134.0, 131.4, 130.0, 129.8, 129.7, 128.7, 128.6, 128.4, 127.8, 127.8, 51.5, 33.6, 28.5, 27.6, 26.5; **HRMS (ESI)**: *m/z* $[M+H]^+$ calcd for $C_{21}H_{23}N_2O_3$: 351.17032, found: 351.17046.

N-hydroxy-7-(6-oxodibenzo[b,f]azocin-5(6H)-yl)heptanamide (**7l**): Yield: 54 %, white crystals, mp 151.0-152.0°C, Rf = 0.47 (methanol: chloroform 1 : 9). **^{1}H NMR** (500 MHz, CD_3OD) δ 7.32 – 6.92 (m, 10H, H_{Ar} + $H_{CH=CH}$), 4.42 (dt, *J* = 13.3, 7.6 Hz, 1H, H_{CH}), 3.34 – 3.31 (m, 1H, H_{CH}), 2.06 (t, *J* = 7.4 Hz, 2H, H_{CH2}), 1.57 (p, *J* = 7.4 Hz, 2H, H_{CH2}), 1.50 – 1.41 (m, 2H, H_{CH2}), 1.40 – 1.25 (m, 4H, 2xH_{CH2}); **^{13}C NMR** (126 MHz, CD_3OD) δ 173.3, 172.9, 142.2, 138.4, 137.4, 135.0, 133.9, 131.3, 130.0, 129.8, 129.6, 128.7, 128.6, 128.4, 127.8, 127.8, 51.5, 33.6, 29.8, 28.6, 27.7, 26.6; **HRMS (ESI)**: *m/z* $[M+H]^+$ calcd for $C_{22}H_{25}N_2O_3$: 365.18597, found: 365.18616.

N-hydroxy-6-(12-methyl-6,11-dioxo-11,12-dihydrodibenzo[b,f][1,4]diazocin-5(6H)-yl)hexanamide (**7m**): Yield: 58 %, white crystals, mp 95.0-96.0 °C, Rf = 0.47 (methanol:chloroform 1:9). **^{1}H NMR** (500 MHz, CD_3OD) δ 7.42 – 7.33 (m, 4H, H_{Ar}), 7.33 – 7.22 (m, 4H, H_{Ar}), 4.49 (ddd, *J* =

13.4, 8.6, 7.2 Hz, 1H, H_{CH}), 3.53 – 3.39 (m, 4H, H_{CH} + H_{CH3}), 2.10 (t, *J* = 7.4 Hz, 2H, H_{CH2}), 1.73 – 1.60 (m, 2H, H_{CH2}), 1.60 – 1.47 (m, 2H, H_{CH2}), 1.45 – 1.32 (m, 2H, H_{CH2}); **^{13}C NMR** (126 MHz, CD_3OD) δ 172.7, 171.7, 171.3, 142.8, 140.4, 134.3, 133.9, 131.5, 131.4, 130.4, 130.4, 128.7, 127.8, 127.5, 127.2, 50.11, 37.3, 33.6, 29.0, 27.4, 26.4; **HRMS (ESI)**: *m/z* $[M+H]^+$ calcd for $C_{21}H_{24}N_3O_4$: 382.17613, found: 382.17558.

N-hydroxy-6-(8,9-dichloro-12-methyl-6,11-dioxo-11,12-dihydrodibenzo[b,f][1,4]diazocin-5(6H)-yl)hexanamide (**7n**): Yield: 64 %, white crystals, mp 104-105.0 °C, Rf = 0.48 (methanol:chloroform 1:9). **^{1}H NMR** (500 MHz, CD_3OD) δ 7.46 (d, *J* = 4.0 Hz, 2H, H_{Ar}), 7.45 – 7.33 (m, 4H, H_{Ar}), 4.46 (ddd, *J* = 13.5, 8.6, 7.3 Hz, 1H, H_{CH}), 3.52 – 3.39 (m, 4H, H_{CH} + H_{CH3}), 2.09 (t, *J* = 7.4 Hz, 2H, H_{CH2}), 1.74 – 1.60 (m, 2H, H_{CH2}), 1.59 – 1.46 (m, 2H, H_{CH2}), 1.46 – 1.31 (m, 2H, H_{CH2}); **^{13}C NMR** (126 MHz, CD_3OD) δ 172.7, 169.2, 168.8, 142.4, 140.0, 135.6, 135.5, 134.4, 134.0, 130.8, 130.8, 129.7, 129.5, 128.9, 128.1, 50.3, 37.4, 33.6, 29.0, 27.4, 26.3; **HRMS (ESI)**: *m/z* $[M+H]^+$ calcd for $C_{21}H_{22}Cl_2N_3O_4$: 450.09819, 452.09524, found: 450.09780, 452.09489.

N-hydroxy-6-(12-benzyl-6,11-dioxo-11,12-dihydrodibenzo[b,f][1,4]diazocin-5(6H)-yl)hexanamide (**7o**): Yield: 52 %, white crystals, mp 172.0-173.0 °C, Rf = 0.49 (methanol:chloroform 1:9). **^{1}H NMR** (500 MHz, CD_3OD) δ 7.41 – 7.18 (m, 13H, H_{Ar}), 5.49 (d, *J* = 14.3 Hz, 1H, H_{Bn}), 4.77 (d, *J* = 14.3 Hz, 1H, H_{Bn}), 3.37 (ddd, *J* = 13.6, 10.6, 5.3 Hz, 1H, H_{CH}), 2.55 (ddd, *J* = 13.5, 10.5, 5.5 Hz, 1H, H_{CH}), 2.08 (t, *J* = 7.4 Hz, 2H, H_{CH2}), 1.67 – 1.50 (m, 3H, H_{CH2}+ H_{CH}), 1.49 – 1.36 (m, 1H, H_{CH}), 1.31 – 1.12 (m, 2H, H_{CH2}); **^{13}C NMR** (126 MHz, CD_3OD) δ 172.7, 171.4, 171.2, 141.8, 140.7, 138.4, 134.4, 133.9, 131.5, 131.4, 130.4, 130.3, 130.3 (2xC), 130.0 (2xC), 129.3, 128.9, 128.6, 127.2, 127.2, 53.6, 50.3, 33.6, 28.8, 27.5, 26.2; **HRMS (ESI)**: *m/z* $[M+H]^+$ calcd for $C_{27}H_{28}N_3O_4$: 458.20743, found: 458.20681.

N-hydroxy-6-(6-oxo-11,12-dihydrodibenzo[b,f]azocin-5(6H)-yl)hexanamide (**7p**): Yield: 49 %, white crystals, mp 164.0-165.0 °C, Rf = 0.47 (methanol:chloroform 1:9). **^{1}H NMR** (500 MHz, CD_3OD) δ 7.09 (qd, *J* = 7.7, 6.0 Hz, 7H, H_{Ar}), 6.97 (d, *J* = 7.5 Hz, 1H, H_{Ar}), 4.15 (ddd, *J* = 13.3, 9.8, 5.9 Hz, 1H, H_{CH}), 3.60 (ddd, *J* = 11.8, 9.7, 5.5 Hz, 1H, H_{CH}), 3.41 – 3.31 (m, 2H, $H_{CH2\text{-}CH2}$), 3.10 – 2.90 (m, 2H, $H_{CH2\text{-}CH2}$), 2.10 (t, *J* = 7.4 Hz, 2H, H_{CH2}), 1.83 – 1.71 (m, 1H, H_{CH}), 1.71 – 1.53 (m, 3H, H_{CH2}+ H_{CH}), 1.52 – 1.34 (m, 2H, H_{CH2}); **^{13}C NMR** (126 MHz, CD_3OD) δ 174.6, 172.7, 143.2, 139.4, 138.8, 137.1, 131.6, 130.5, 130.3, 129.3, 128.7, 128.0, 127.4, 127.3, 50.5, 33.7, 33.6, 31.6, 28.6, 27.7, 26.5; **HRMS (ESI)**: *m/z* $[M+H]^+$ calcd for $C_{21}H_{25}N_2O_3$: 353.18597, found: 353.18549.

N-hydroxy-6-(2-bromo-11-methyl-6,12-dioxo-11,12-dihydrodibenzo[b,f][1,5]diazocin-5(6H)-yl)hexanamide (**7r**): Yield: 56 %, white crystals, mp 76.0-77.0 °C, Rf = 0.55 (methanol:chloroform 1:9). **^{1}H NMR** (500 MHz, CD_3OD) δ 7.52 (dd, *J* = 8.6, 2.3 Hz, 1H, H_{Ar}), 7.46 (d, *J* = 2.3 Hz, 1H, H_{Ar}), 7.44 – 7.37 (m, 1H, H_{Ar}), 7.34 – 7.25 (m, 3H, H_{Ar}), 7.22 (d, *J* = 8.6 Hz, 1H, H_{Ar}), 4.52 (dt, *J* = 13.5, 7.8 Hz, 1H, H_{CH}), 3.43 (s, 3H, H_{CH3}), 3.37 (dt, *J* = 13.3, 6.4 Hz, 1H, H_{CH}), 2.08 (t, *J* = 7.4 Hz, 2H, H_{CH2}), 1.70 – 1.57 (m, 2H, H_{CH2}), 1.56 – 1.46 (m, 2H, H_{CH2}), 1.41

– 1.27 (m, 2H, H_{CH2}); **^{13}C NMR** (126 MHz, CD_3OD) δ 172.8, 169.8, 168.6, 141.1, 139.3, 138.3, 135.5, 135.3, 132.3, 131.3, 129.9, 129.2, 128.4, 126.7, 122.8, 49.9, 37.0, 33.6, 28.5, 27.4, 26.3; **HRMS (ESI)**: *m/z* $[M+H]^+$ calcd for $C_{21}H_{23}BrN_3O_4$: 460.08665, 462.08460, found: 460.08622, 462.08401.

N-hydroxy-6-(2,3-dimethoxy-11-methyl-6,12-dioxo-11,12-dihydrodibenzo[b,f][1,5]diazocin-5(6H)-yl)hexanamide (**7s**): Yield: 50 %, white crystals, mp 82.0-83.0 °C, Rf = 0.49 (methanol:chloroform 1:9). **^{1}H NMR** (500 MHz, CD_3OD) δ 7.42 – 7.34 (m, 1H, H_{Ar}), 7.34 – 7.22 (m, 3H, H_{Ar}), 6.81 (d, *J* = 11.5 Hz, 2H, H_{Ar}), 4.54 (dt, *J* = 13.5, 7.8 Hz, 1H, H_{CH}), 3.76 (d, *J* = 5.1 Hz, 6H, H_{OCH3}), 3.42 (s, 3H, H_{CH3}), 3.38 – 3.32 (m, 1H, H_{CH}), 2.09 (t, *J* = 7.4 Hz, 2H, H_{CH2}), 1.74 – 1.56 (m, 2H, H_{CH2}), 1.57 – 1.44 (m, 2H, H_{CH2}), 1.44 – 1.27 (m, 2H, H_{CH2}); **^{13}C NMR** (126 MHz, CD_3OD) δ 172.8, 170.5, 170.2, 152.4, 150.3, 141.7, 135.8, 133.2, 132.0, 129.5, 128.3, 128.2, 126.5, 110.4, 110.0, 56.6, 56.5, 49.8, 37.1, 33.6, 28.4, 27.4, 26.4; **HRMS (ESI)**: *m/z* $[M+H]^+$ calcd for $C_{23}H_{28}N_3O_6$: 442.19726, found: 442.19664.

N-hydroxy-6-(5-methyl-6,14-dioxo-5,14-dihydrobenzonaphtho[2,3-f][1,5]diazocin-13(6H)-yl)hexanamide (**7t**): Yield: 50 %, white crystals, mp 161.0-162.0 °C, Rf = 0.41 (methanol:chloroform 1:9). **^{1}H NMR** (500 MHz, DMSO-d_6) δ 7.91 (dd, *J* = 24.5, 8.4 Hz, 4H, H_{Ar}), 7.56 (pd, *J* = 6.8, 1.5 Hz, 2H, H_{Ar}), 7.44 – 7.30 (m, 3H, H_{Ar}), 4.42 (dt, *J* = 13.3, 7.8 Hz, 1H, H_{CH}), 3.53 – 3.40 (m, 4H, H_{CH} + H_{CH3}), 1.91 (t, *J* = 7.4 Hz, 2H, H_{CH2}), 1.57 – 1.30 (m, 4H, 2xH_{CH2}), 1.30 – 1.18 (m, 2H, H_{CH2}); **^{13}C NMR** (126 MHz, DMSO) δ 168.9, 167.2, 165.5, 138.5, 136.7, 136.0, 133.7, 133.2, 132.4, 131.5, 130.7, 128.0, 127.9, 127.9, 127.8, 127.4, 127.2, 126.7, 125.1, 48.9, 36.1, 32.2, 27.2, 26.0, 24.8; **HRMS (ESI)**: *m/z* $[M+H]^+$ calcd for $C_{25}H_{25}ClN_3O_4$: 466.15281, 468.14986, found: 466.15250, 468.14997.

3. Spectra

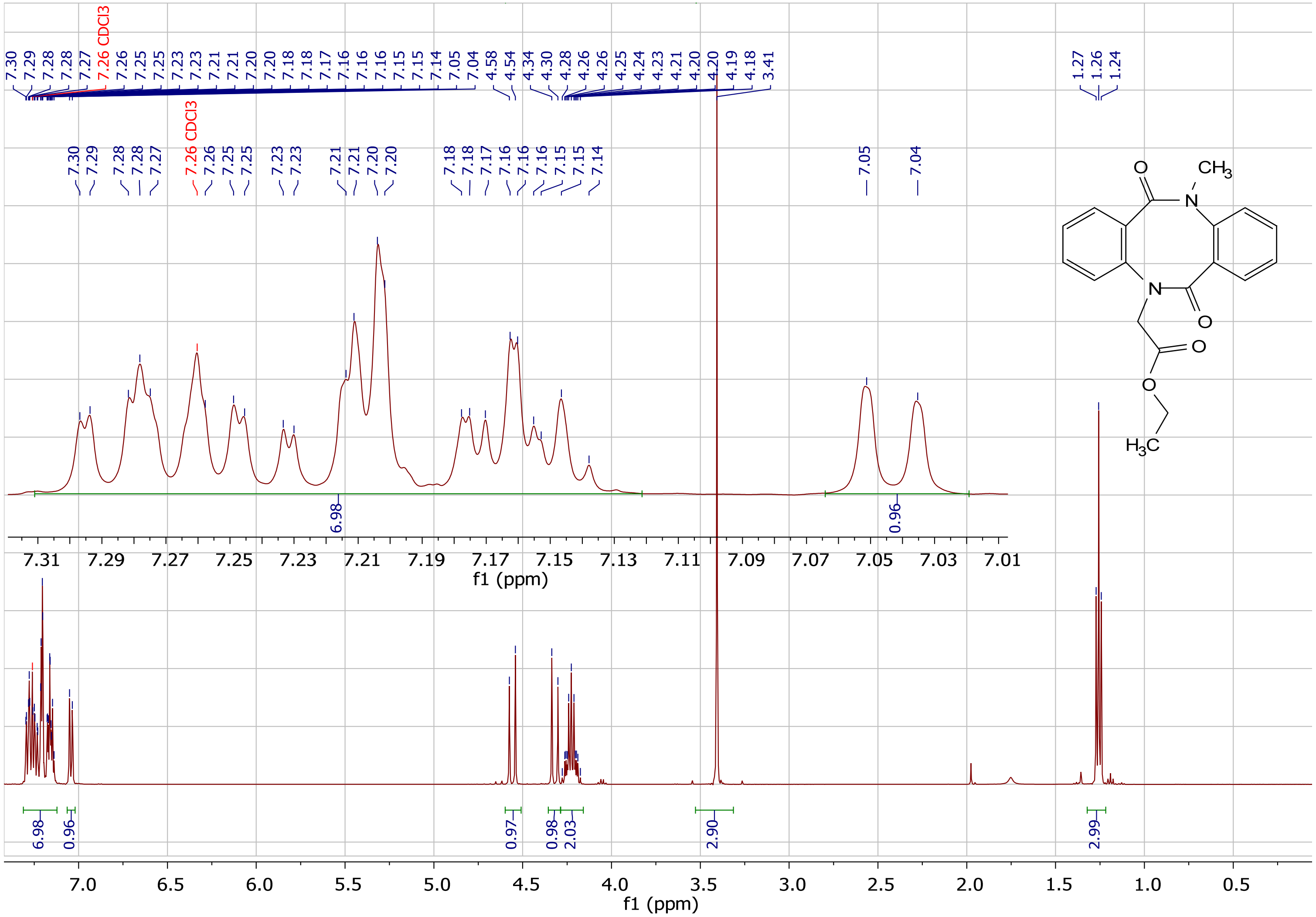

Figure S1. 1H NMR spectrum of ethyl 2-(11-methyl-6,12-dioxo-11,12-dihydrodibenzo[*b*,*f*][1,5]diazocin-5(6*H*)-yl)acetate (**10a**).

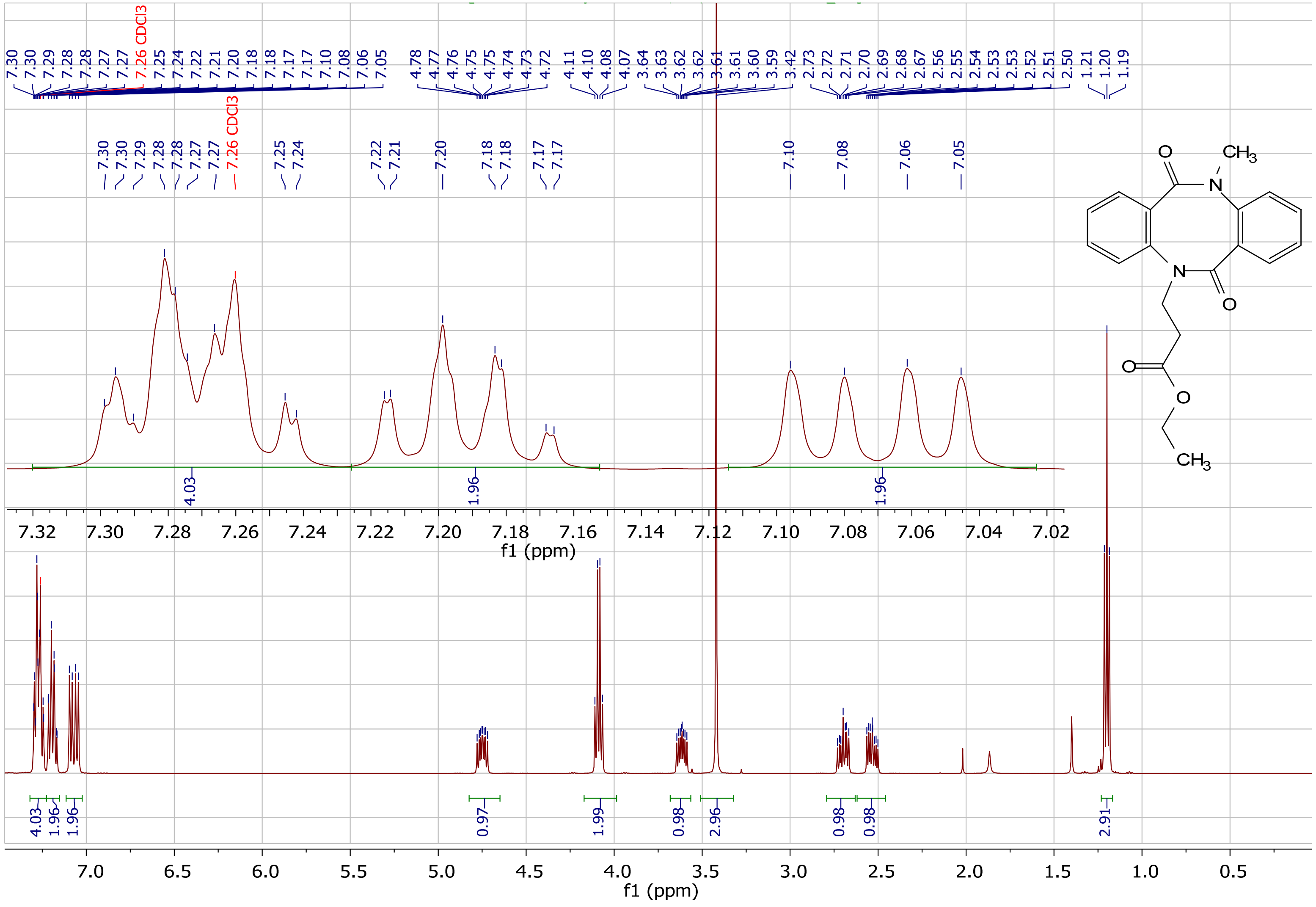

Figure S2. 1H NMR spectrum of ethyl 3-(11-methyl-6,12-dioxo-11,12-dihydrodibenzo[*b*,*f*][1,5]diazocin-5(6*H*)-yl)propanoate (**10b**).

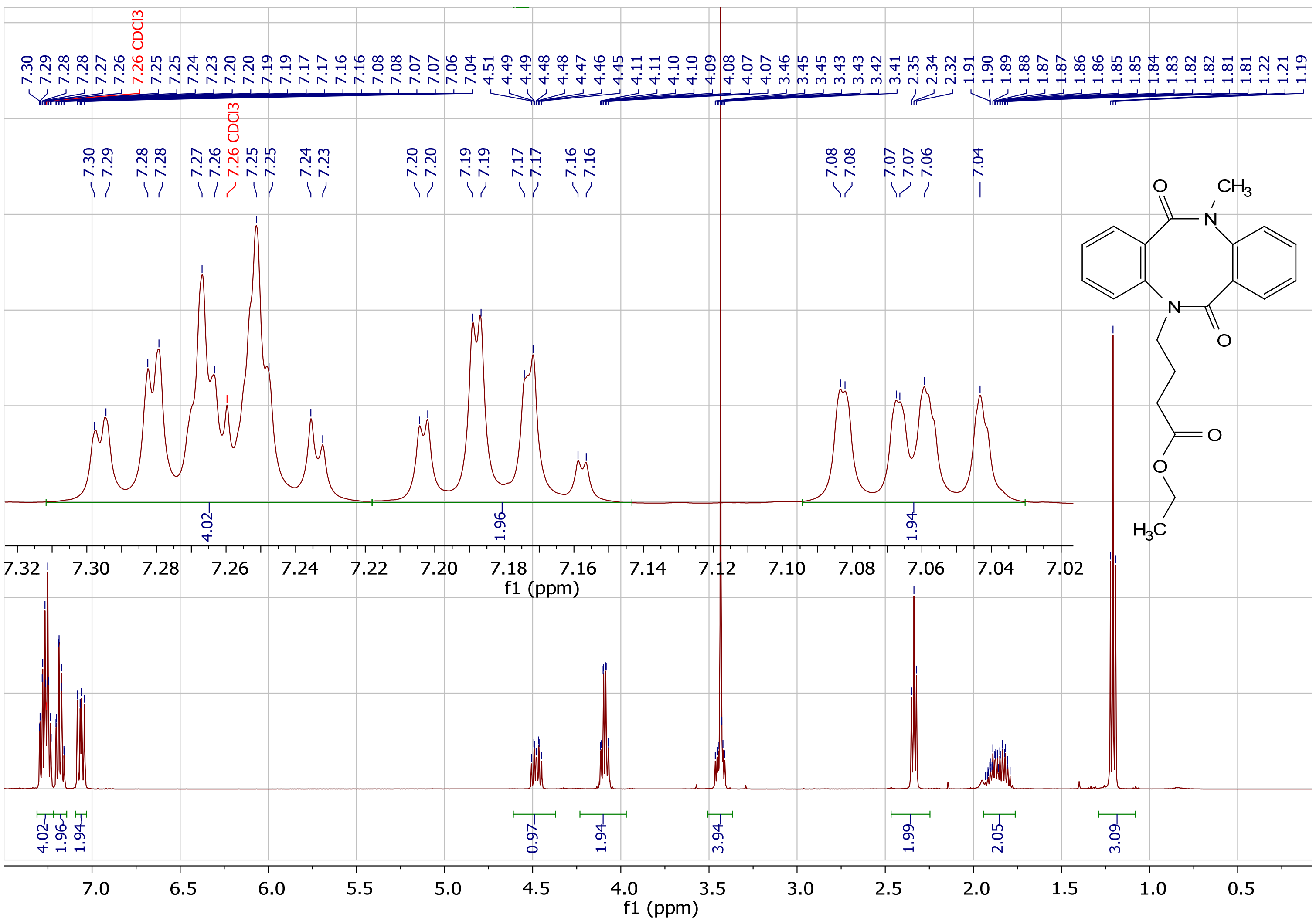

Figure S3. 1H NMR spectrum of ethyl 4-(11-methyl-6,12-dioxo-11,12-dihydrodibenzo[*b,f*][1,5]diazocin-5(6*H*)-yl)butanoate (**10c**).

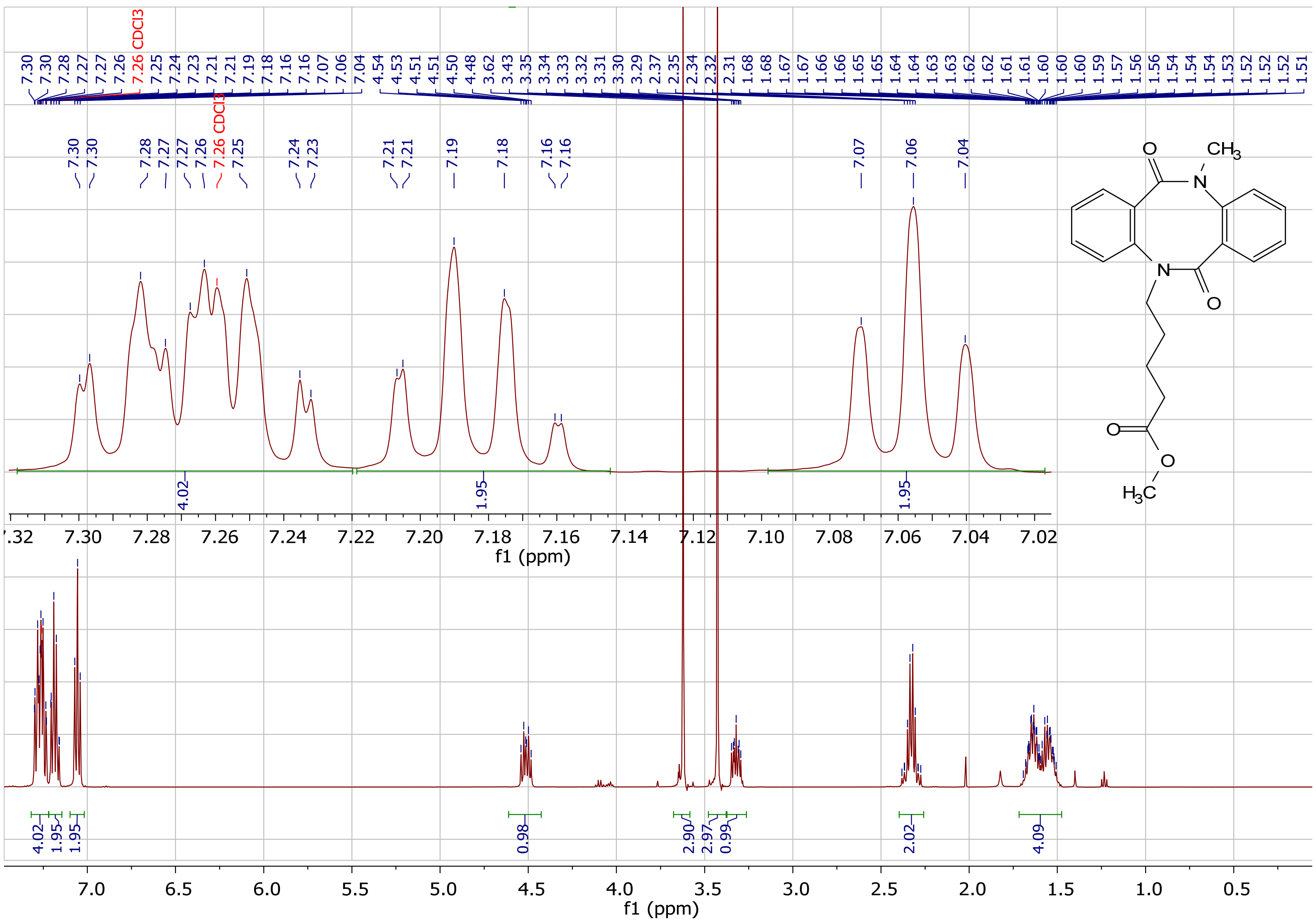

Figure S4. 1H NMR spectrum of methyl 5-(11-methyl-6,12-dioxo-11,12-dihydrodibenzo[*b*,*f*][1,5]diazocin-5(6*H*)-yl)pentanoate (**10d**).

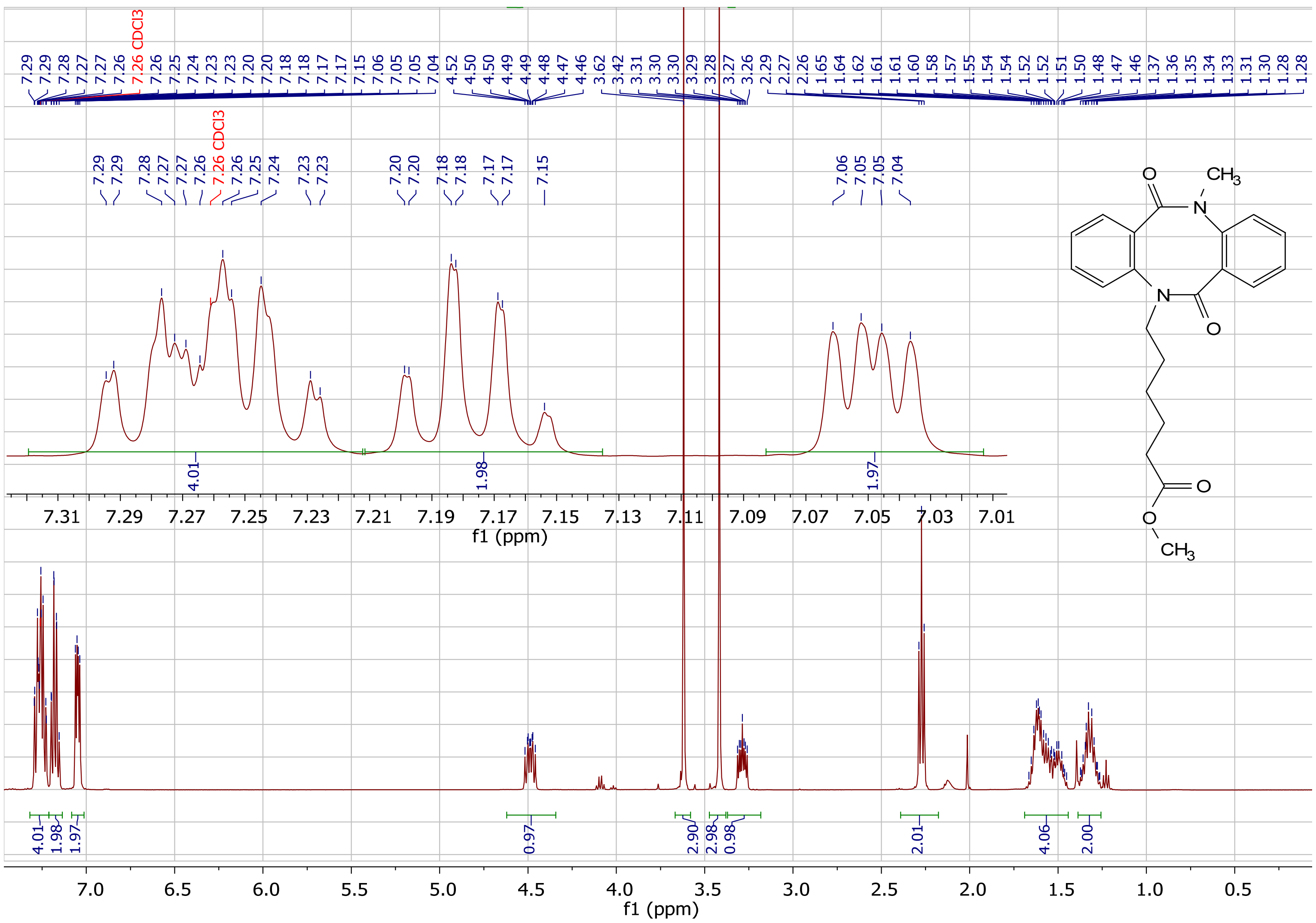

Figure S5. 1H NMR spectrum of methyl 6-(11-methyl-6,12-dioxo-11,12-dihydrodibenzo[*b*,*f*][1,5]diazocin-5(6*H*)-yl)hexanoate (**10e**).

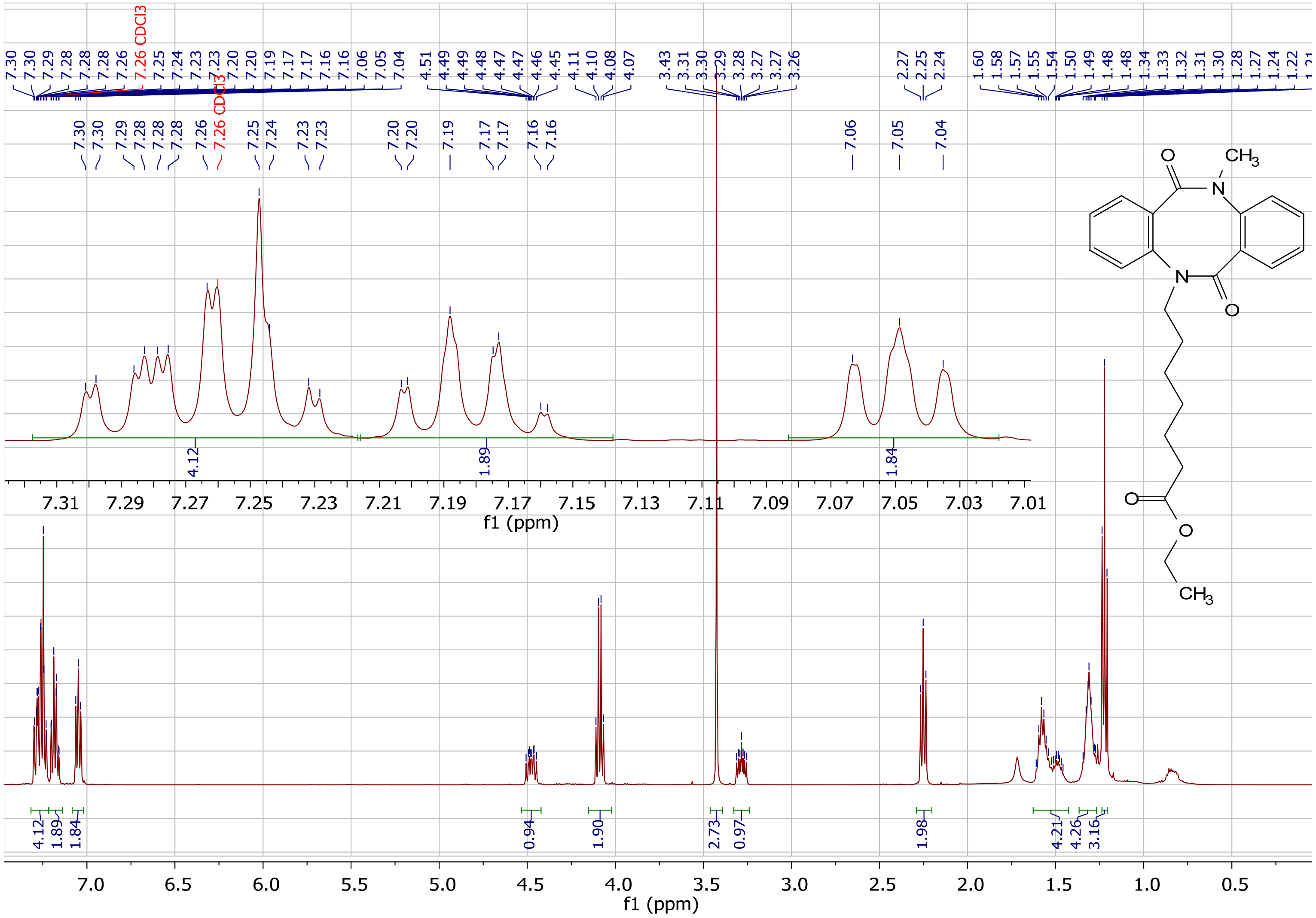

Figure S6. 1H NMR spectrum of ethyl 7-(11-methyl-6,12-dioxo-11,12-dihydrodibenzo[*b*,*f*][1,5]diazocin-5(6*H*)-yl)heptanoate (**10f**).

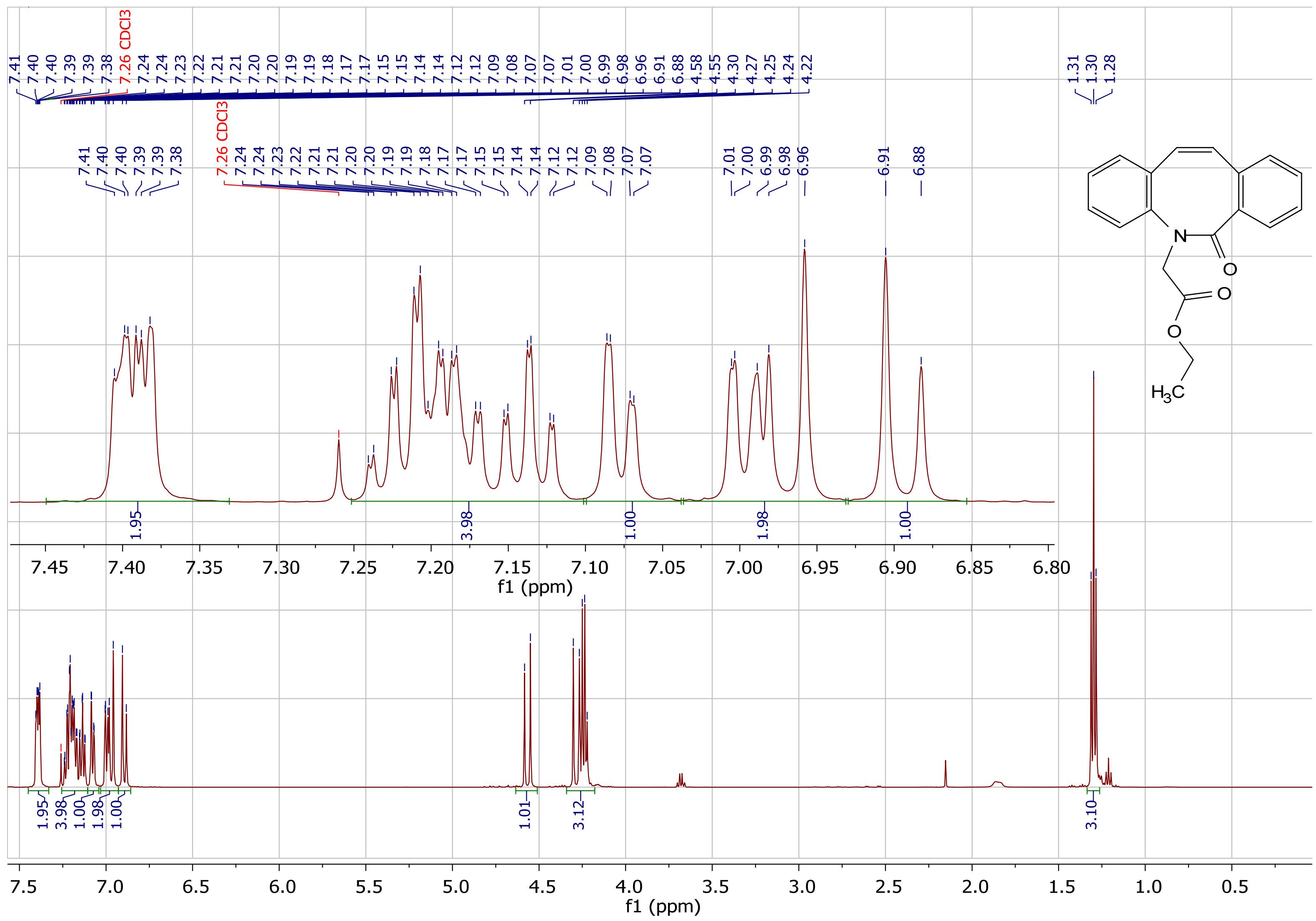

Figure S7. 1H NMR spectrum of ethyl 2-(6-oxodibenzo[*b*,*f*]azocin-5(6*H*)-yl)acetate (**10g**).

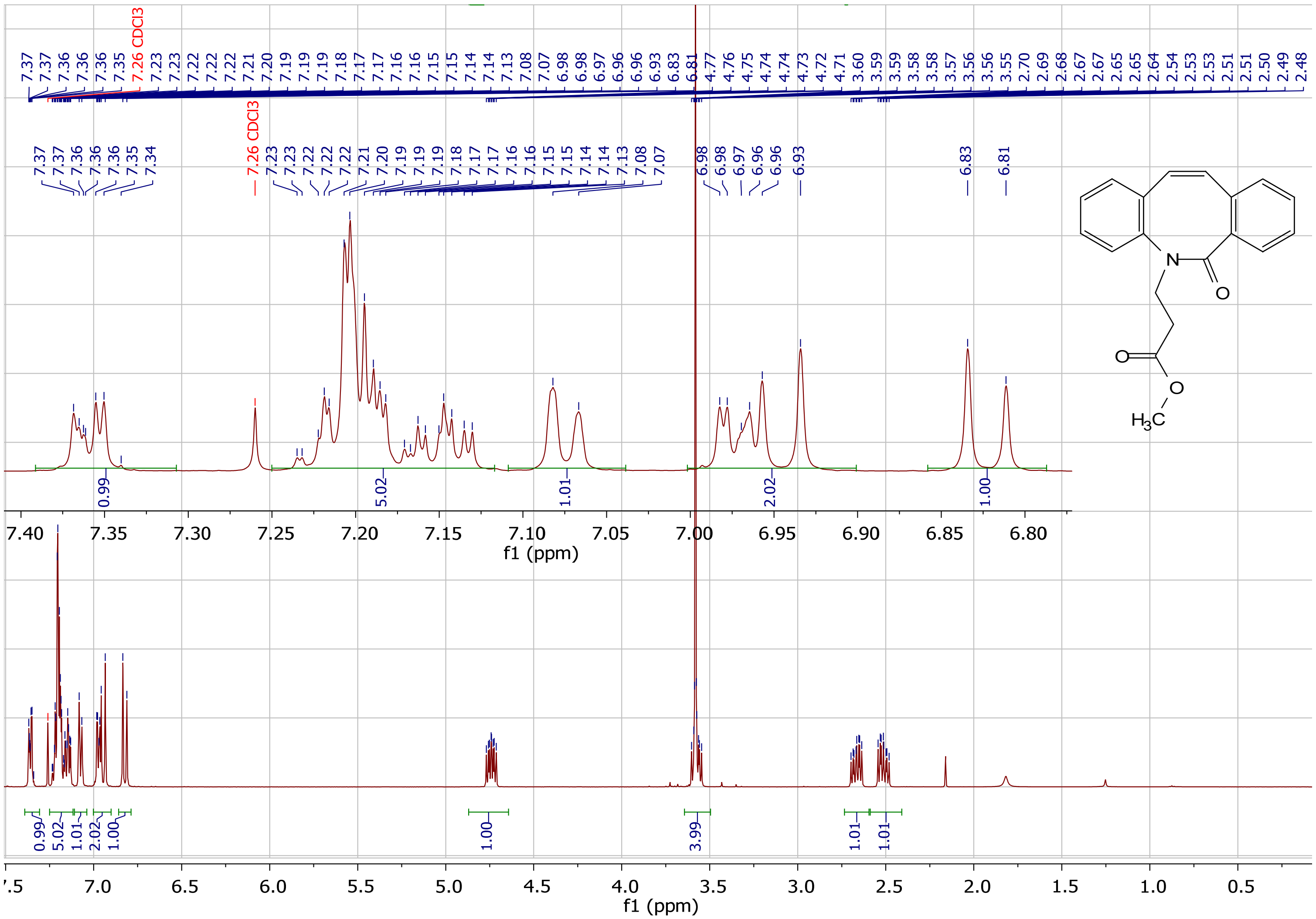

Figure S8. 1H NMR spectrum of methyl 3-(6-oxodibenzo[*b*,*f*]azocin-5(6*H*)-yl)propanoate (**10h**).

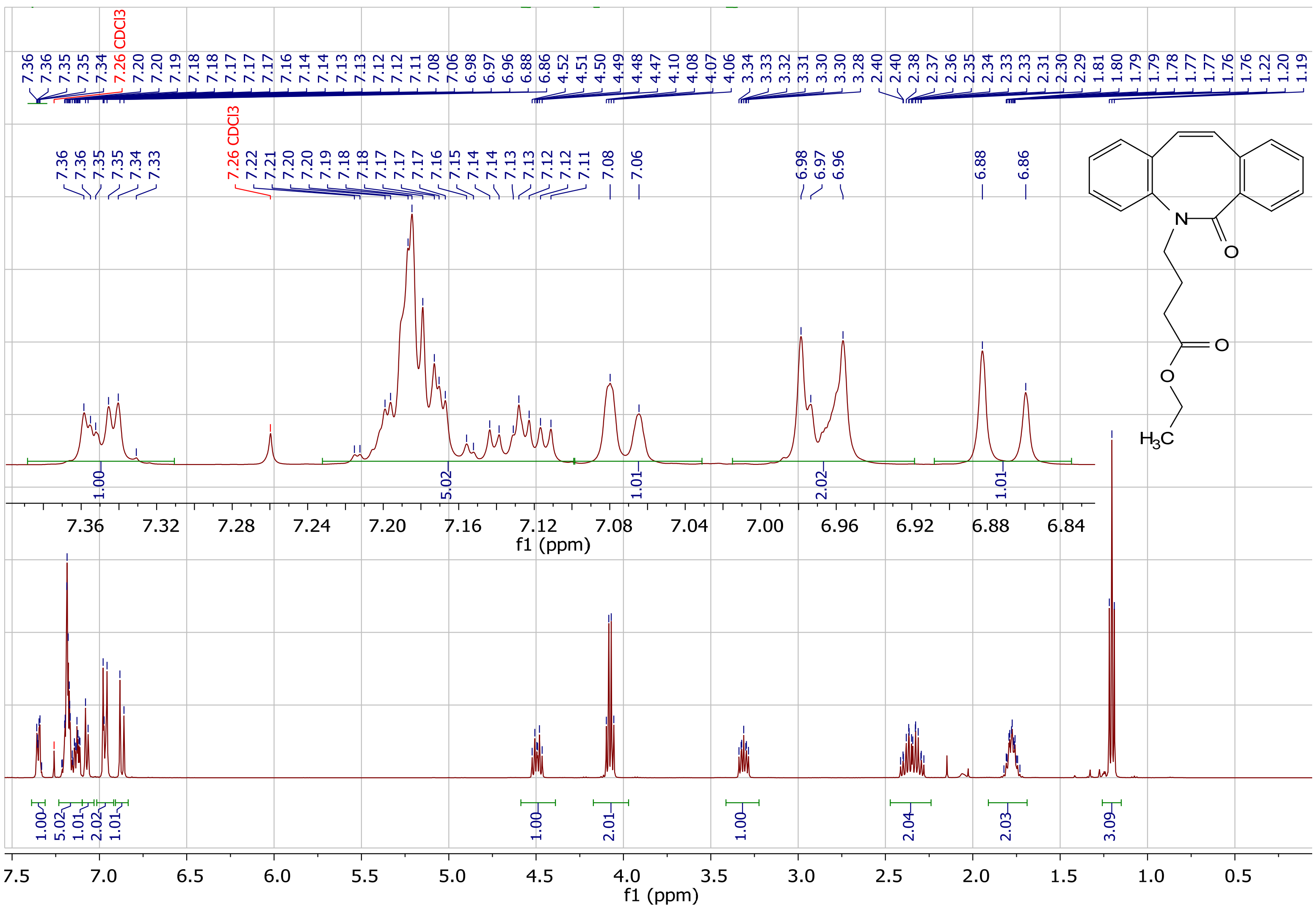

Figure S9. 1H NMR spectrum of ethyl 4-(6-oxodibenzo[*b,f*]azocin-5(6*H*)-yl)butanoate (**10i**).

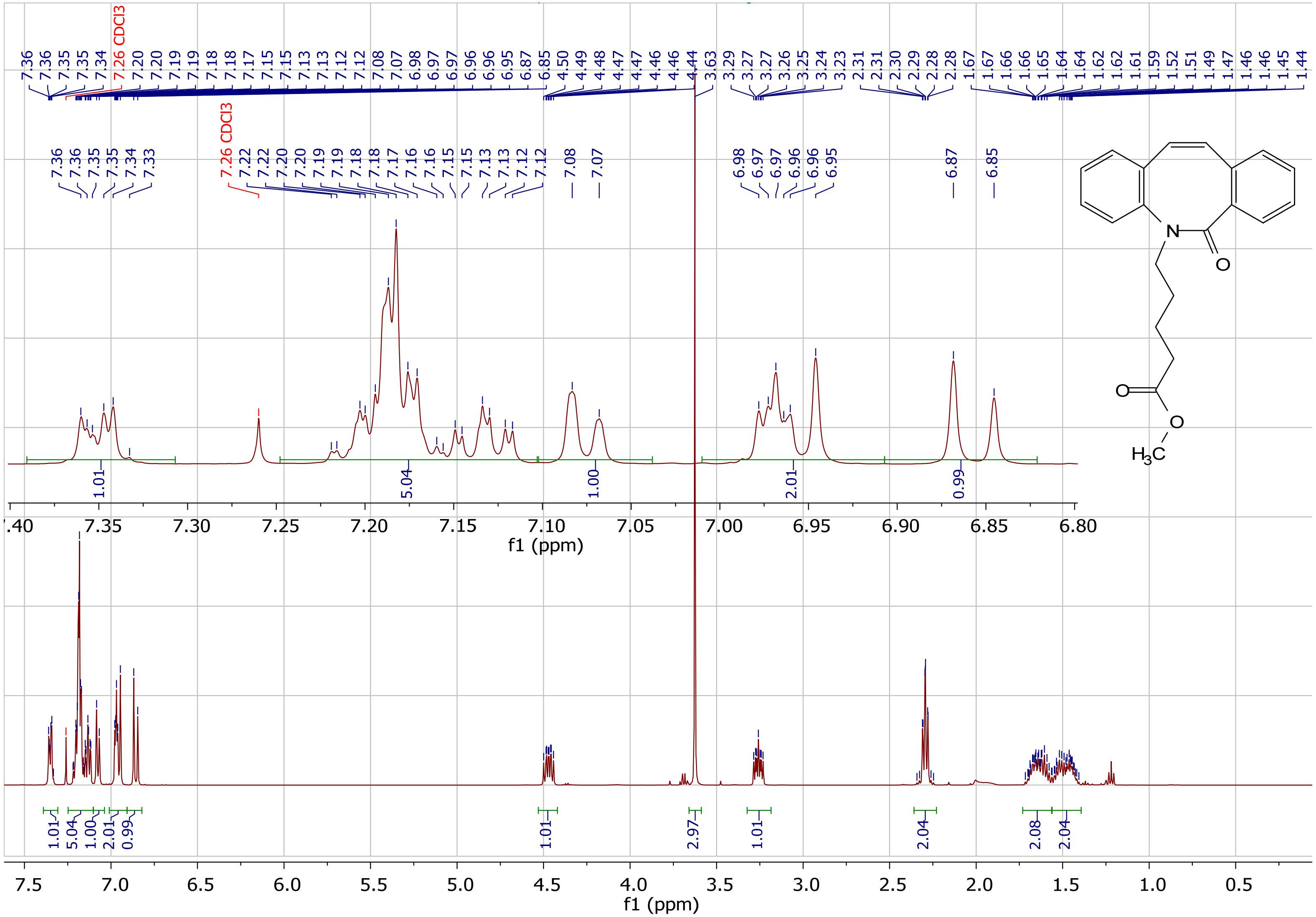

Figure S10. 1H NMR spectrum of methyl 5-(6-oxodibenzo[*b*,*f*]azocin-5(6*H*)-yl)pentanoate (**10j**).

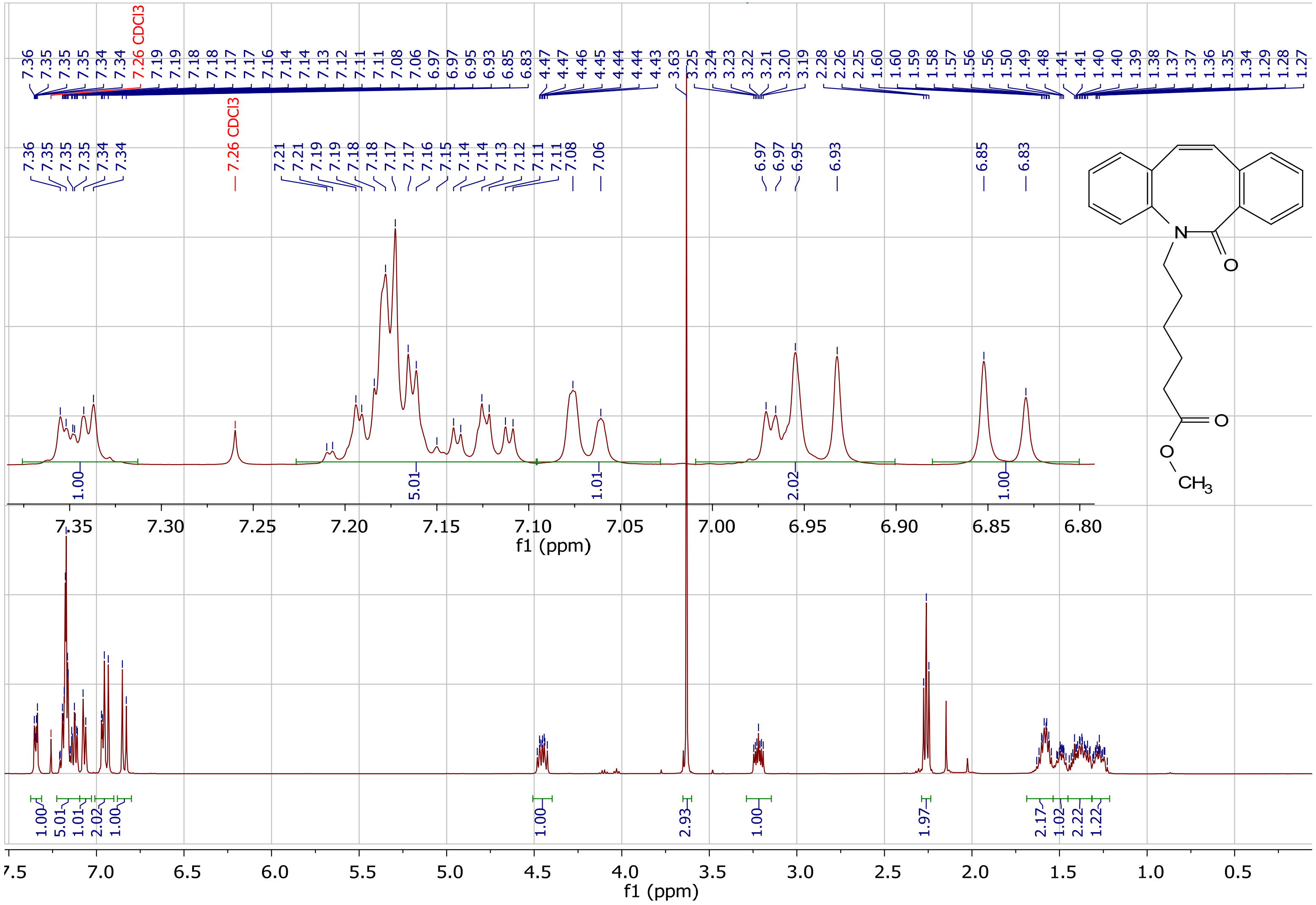

Figure S11. 1H NMR spectrum of methyl 6-(6-oxodibenzo[*b*,*f*]azocin-5(6*H*)-yl)hexanoate (**10k**).

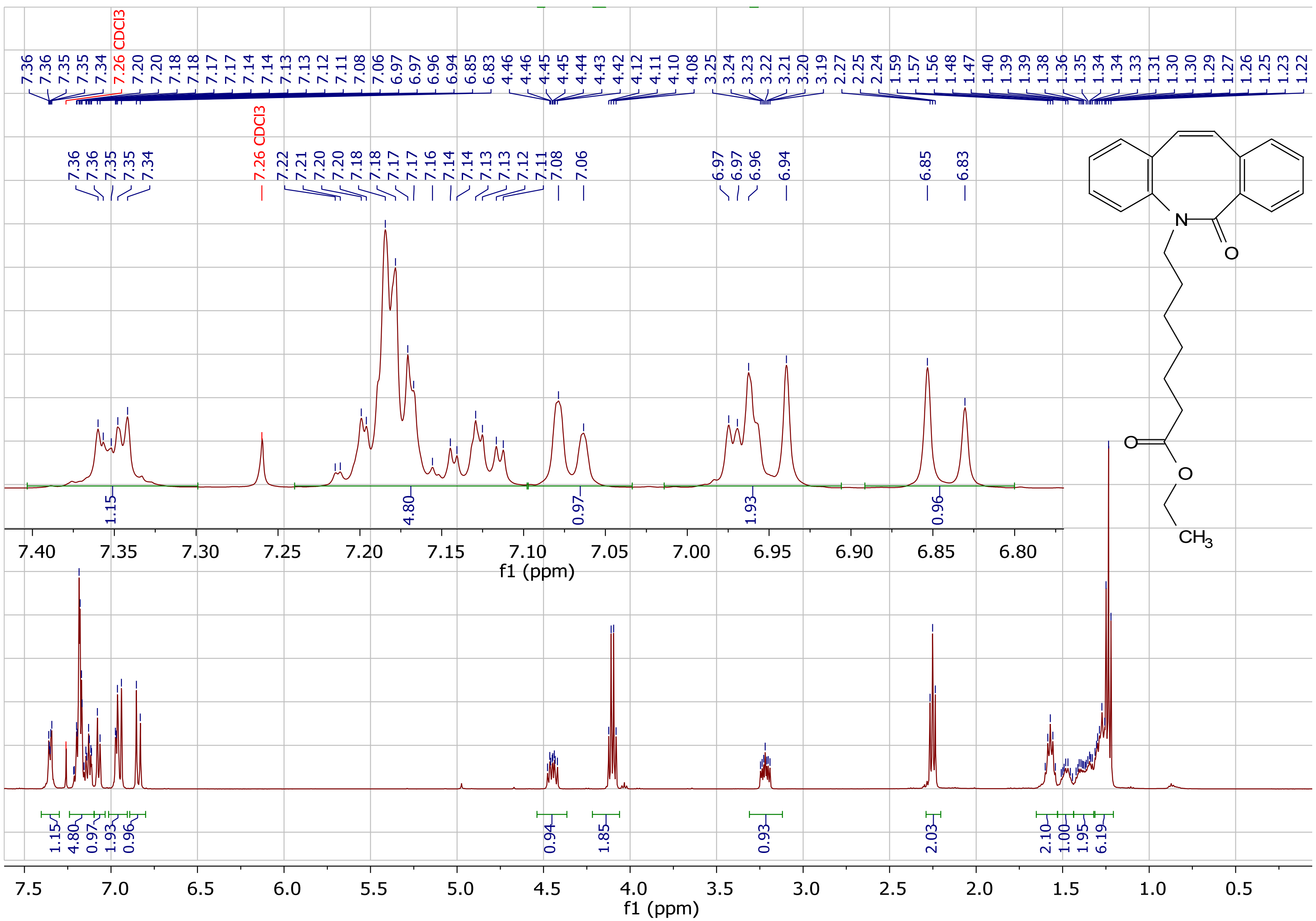

Figure S12. 1H NMR spectrum of ethyl 7-(6-oxodibenzo[*b*,*f*]azocin-5(6*H*)-yl)heptanoate (**10l**).

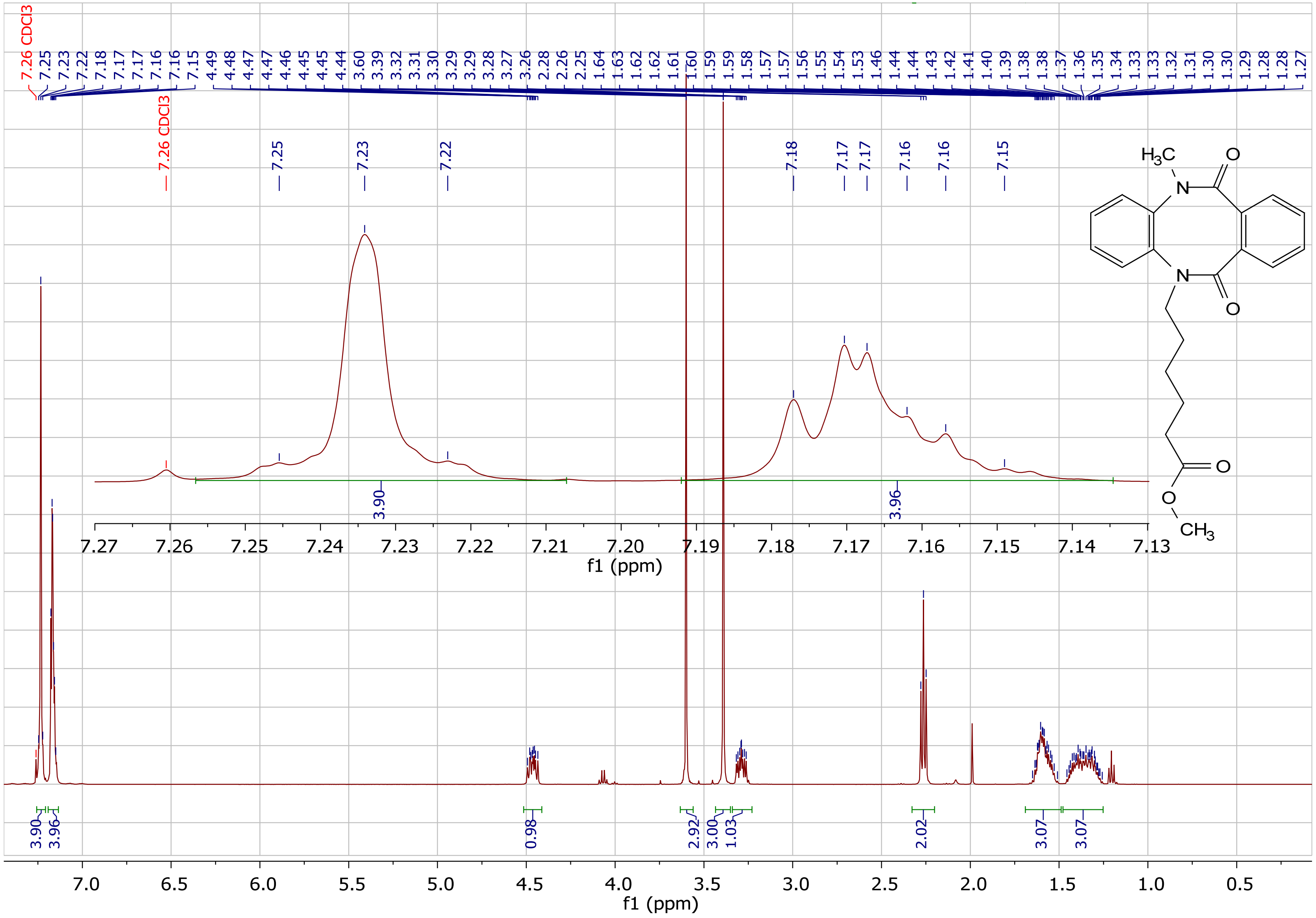

Figure S13. 1H NMR spectrum of methyl 6-(12-methyl-6,11-dioxo-11,12-dihydrodibenzo[*b*,*f*][1,4]diazocin-5(6*H*)-yl)hexanoate (**10m**).

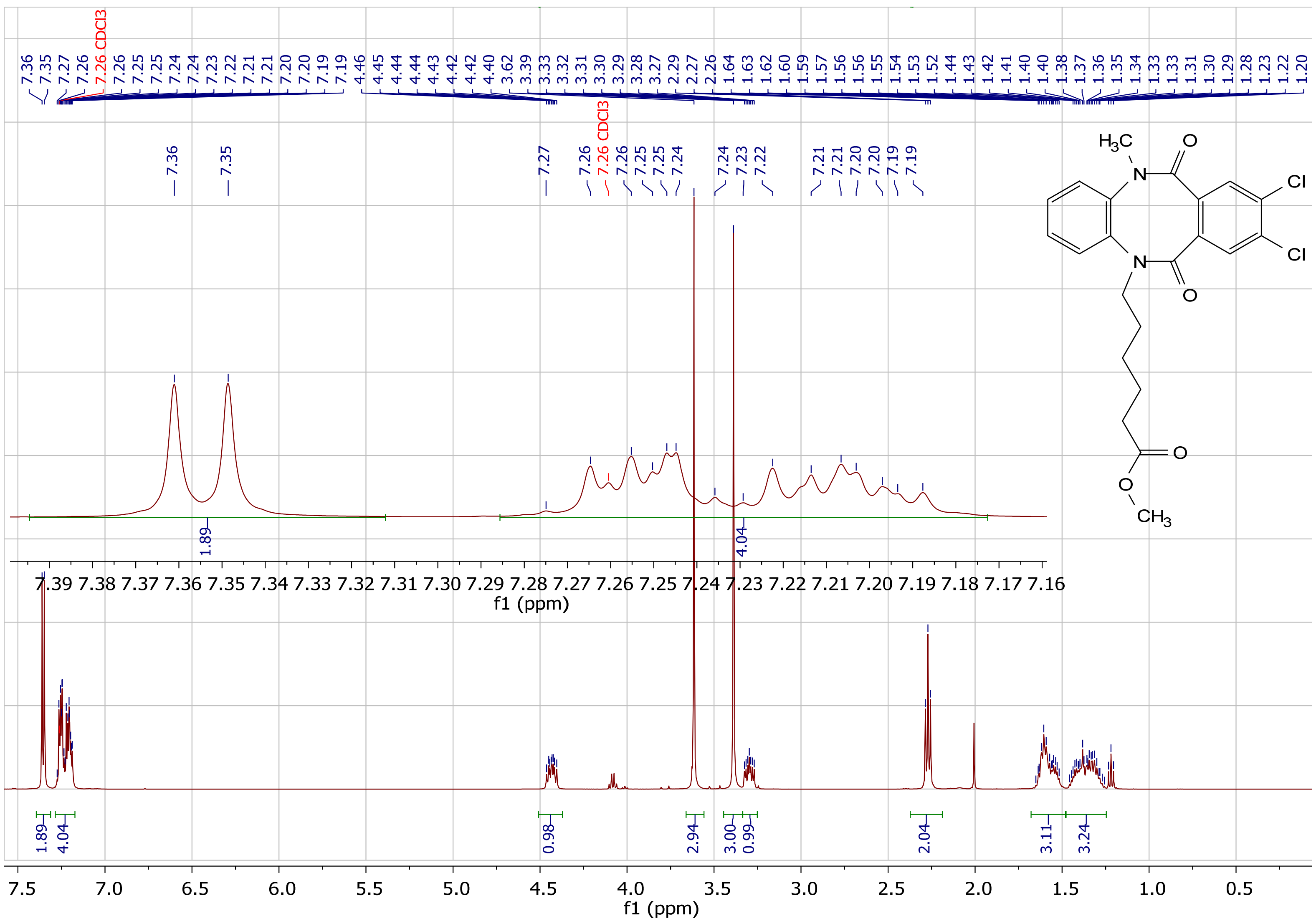

Figure S14. 1H NMR spectrum of methyl 6-(8,9-dichloro-12-methyl-6,11-dioxo-11,12-dihydrodibenzo[*b*,*f*][1,4]diazocin-5(6*H*)-yl) hexanoate (**10n**).

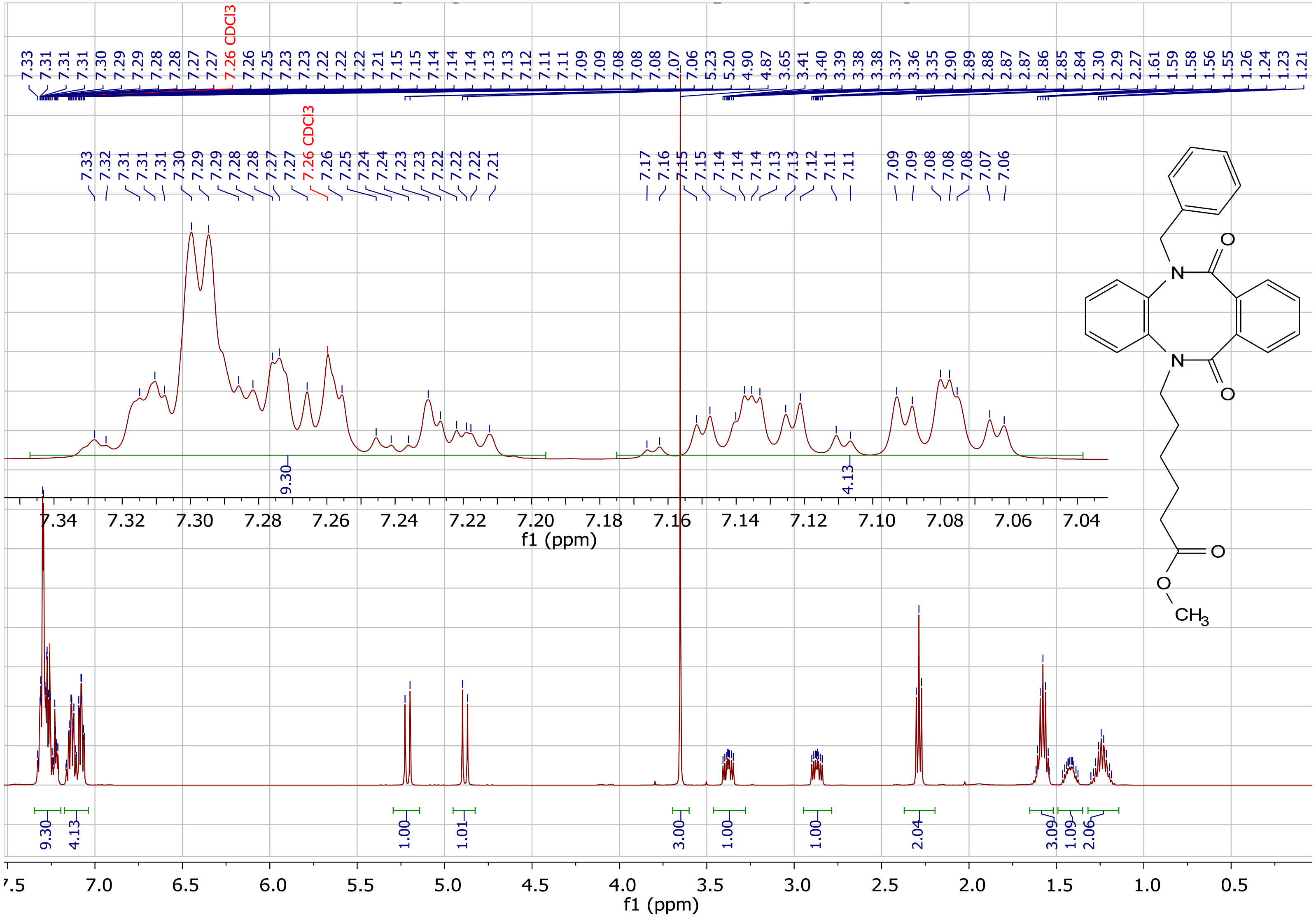

Figure S15. 1H NMR spectrum of methyl 6-(12-benzyl-6,11-dioxo-11,12-dihydrodibenzo[*b*,*f*][1,4]diazocin-5(6*H*)-yl)hexanoate (**10o**).

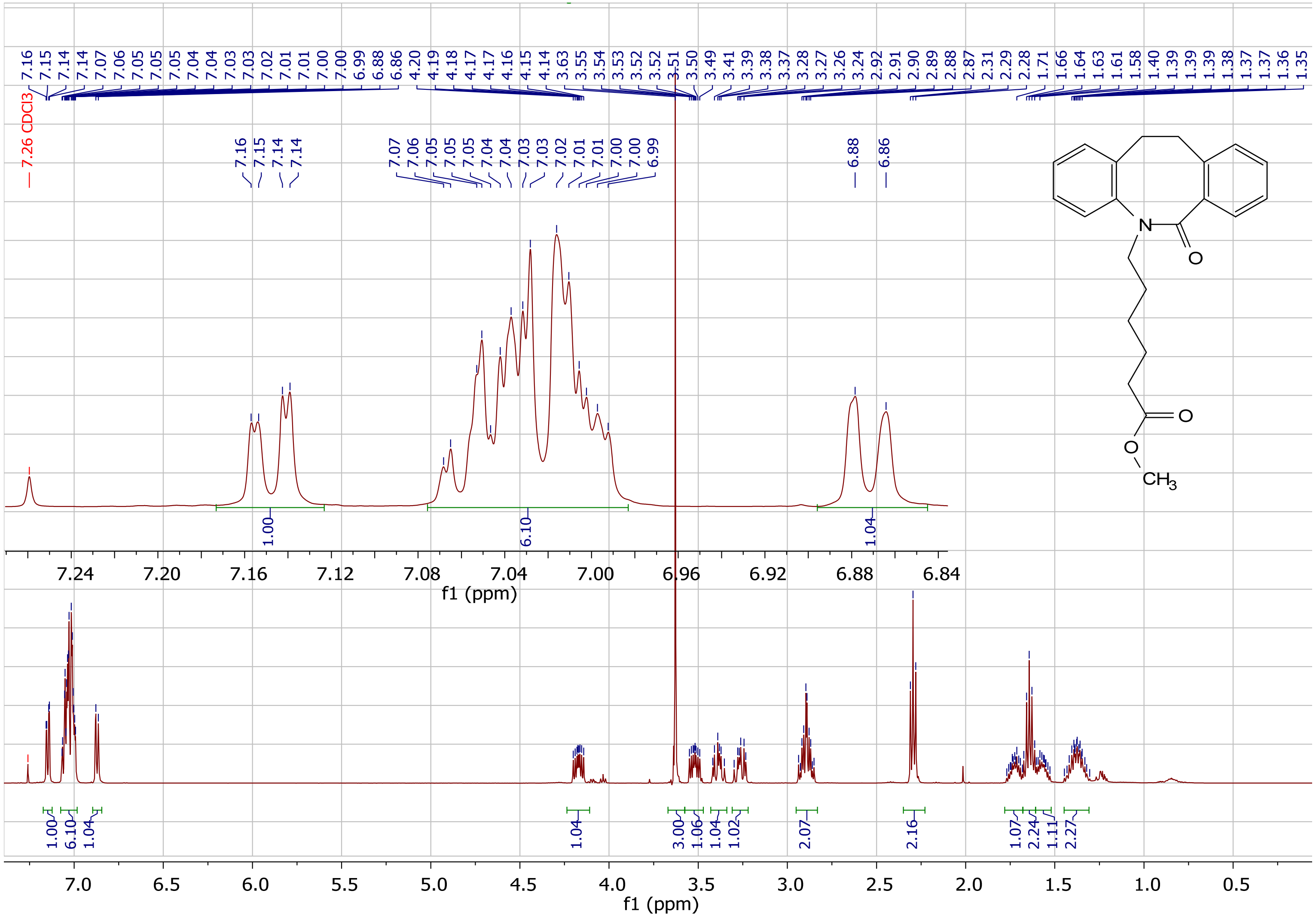

Figure S16. 1H NMR spectrum of methyl 6-(6-oxo-11,12-dihydrodibenzo[*b*,*f*]azocin-5(6*H*)-yl)hexanoate (**10p**).

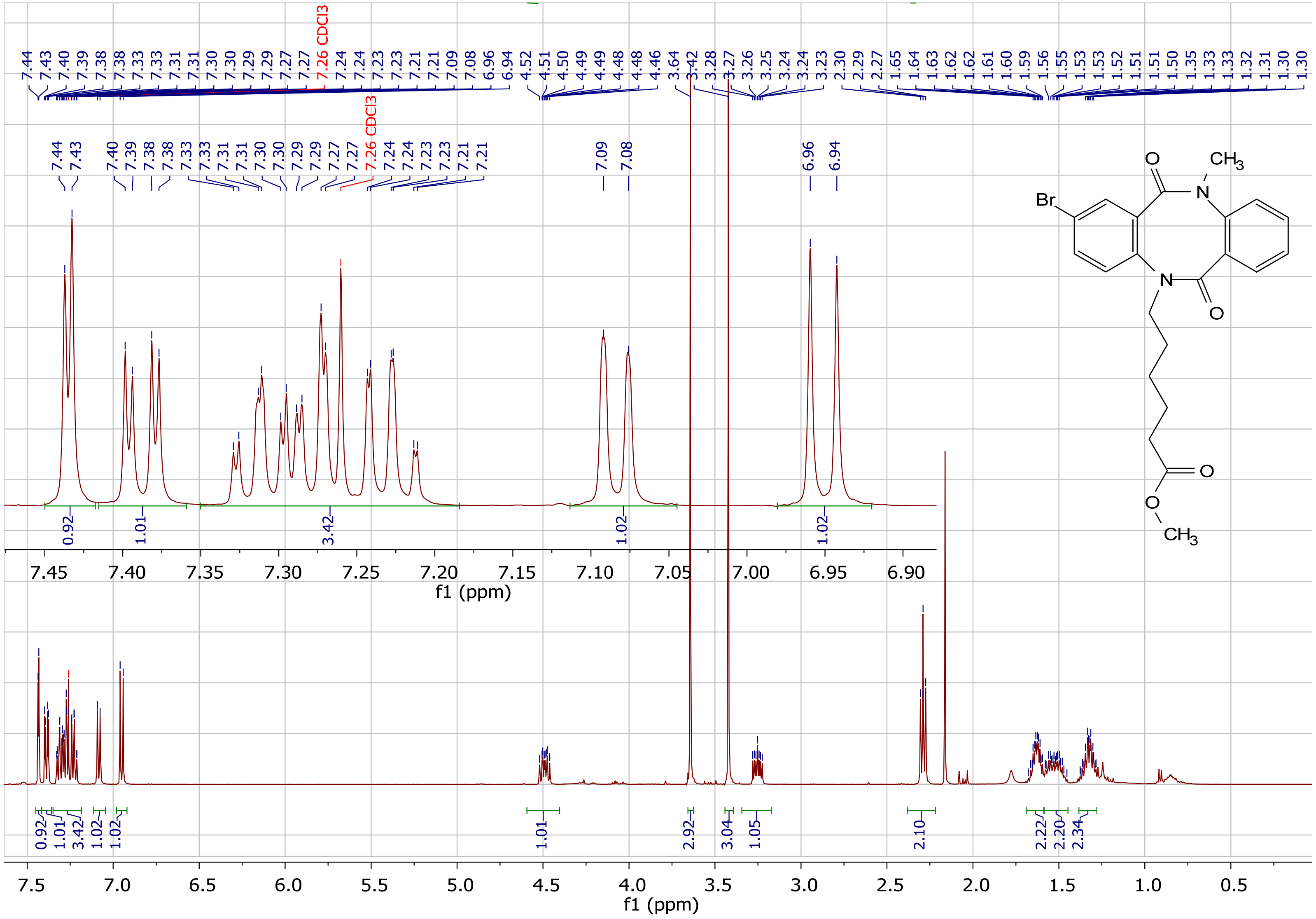

Figure S17. 1H NMR spectrum of methyl 6-(2-bromo-11-methyl-6,12-dioxo-11,12-dihydrodibenzo[*b*,*f*][1,5]diazocin-5(6*H*)-yl)hexanoate (**10r**).

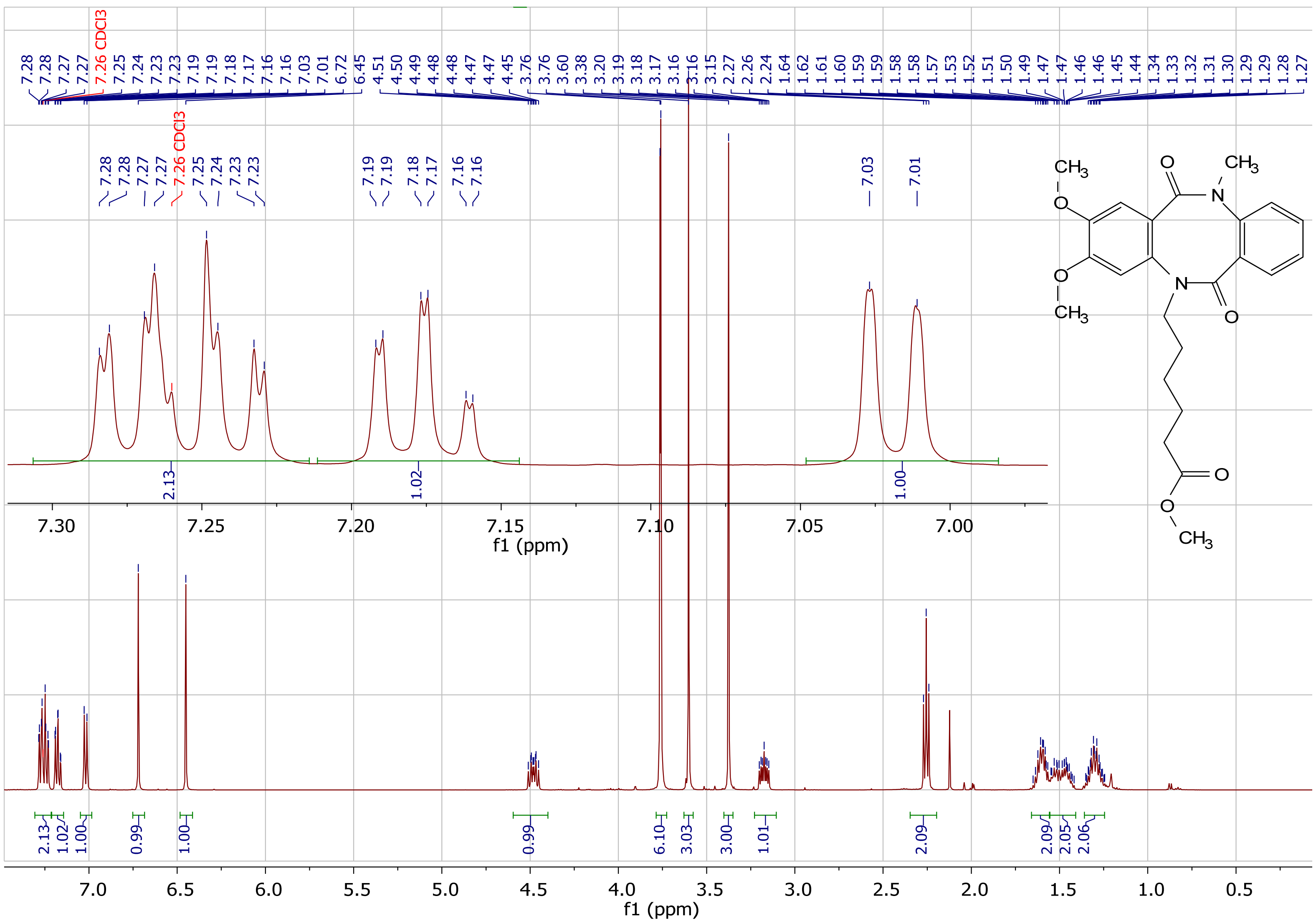

Figure S18. 1H NMR spectrum of methyl 6-(2,3-dimethoxy-11-methyl-6,12-dioxo-11,12-dihydrodibenzo[*b*,*f*][1,5]diazocin-5(6*H*)-yl)hexanoate (**10s**).

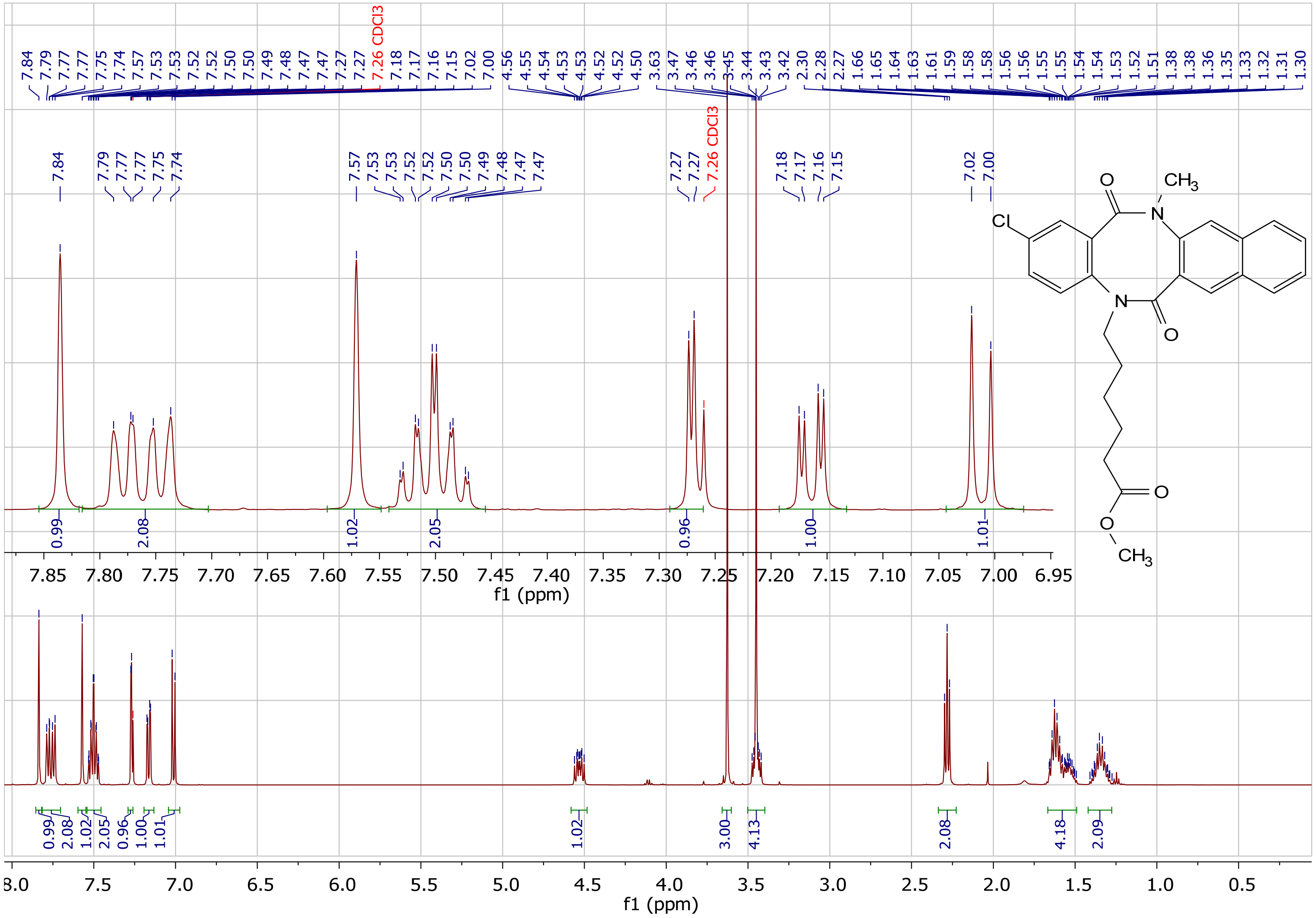

Figure S19. 1H NMR spectrum of methyl 6-(5-methyl-6,14-dioxo-5,14-dihydrobenzonaphtho[2,3-*f*][1,5]diazocin-13(6*H*)-yl)hexanoate (**10t**).

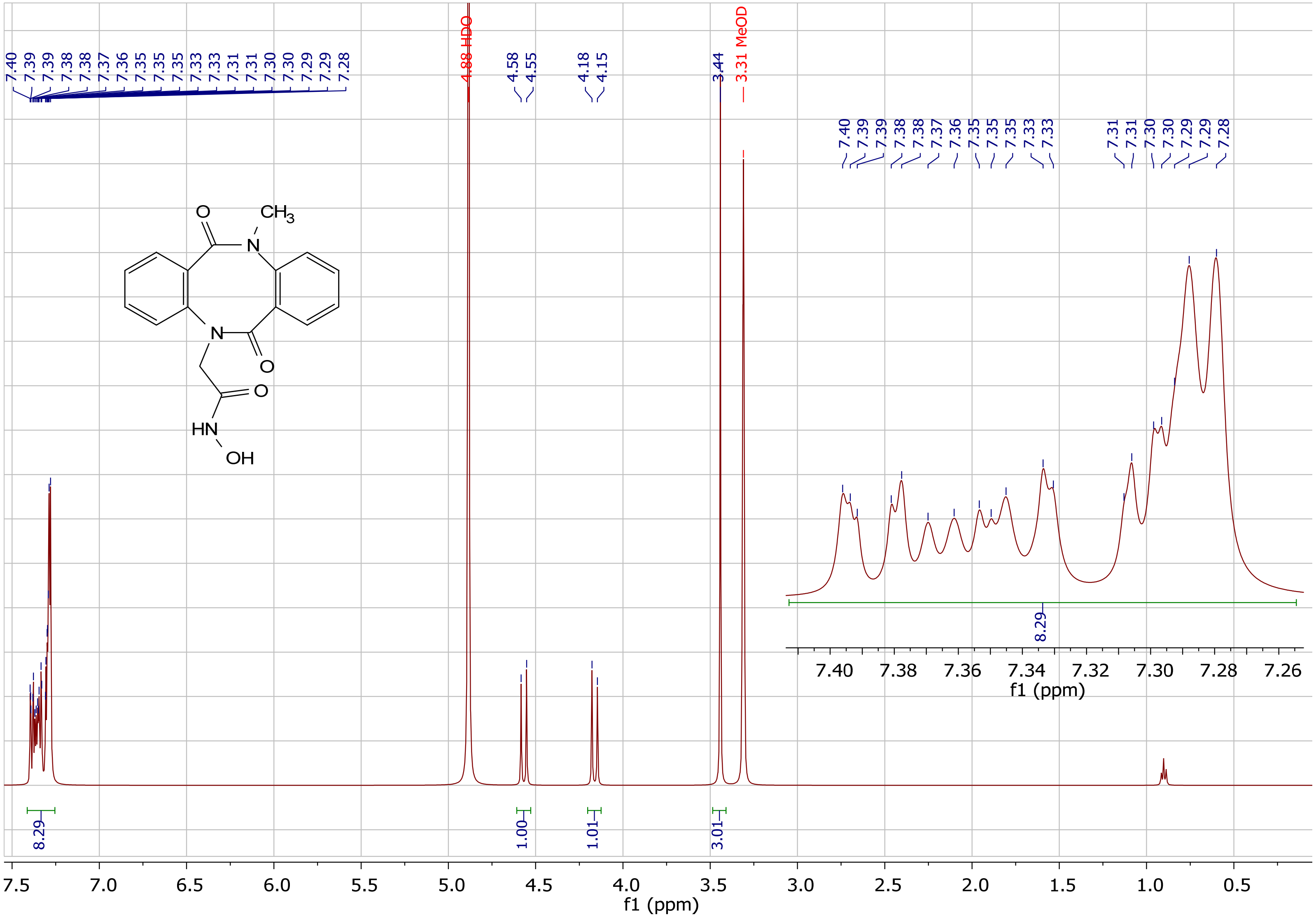

Figure S20. 1H NMR spectrum of *N*-hydroxy-2-(11-methyl-6,12-dioxo-11,12-dihydrodibenzo[*b*,*f*][1,5]diazocin-5(6*H*)-yl)acetamide (**7a**).

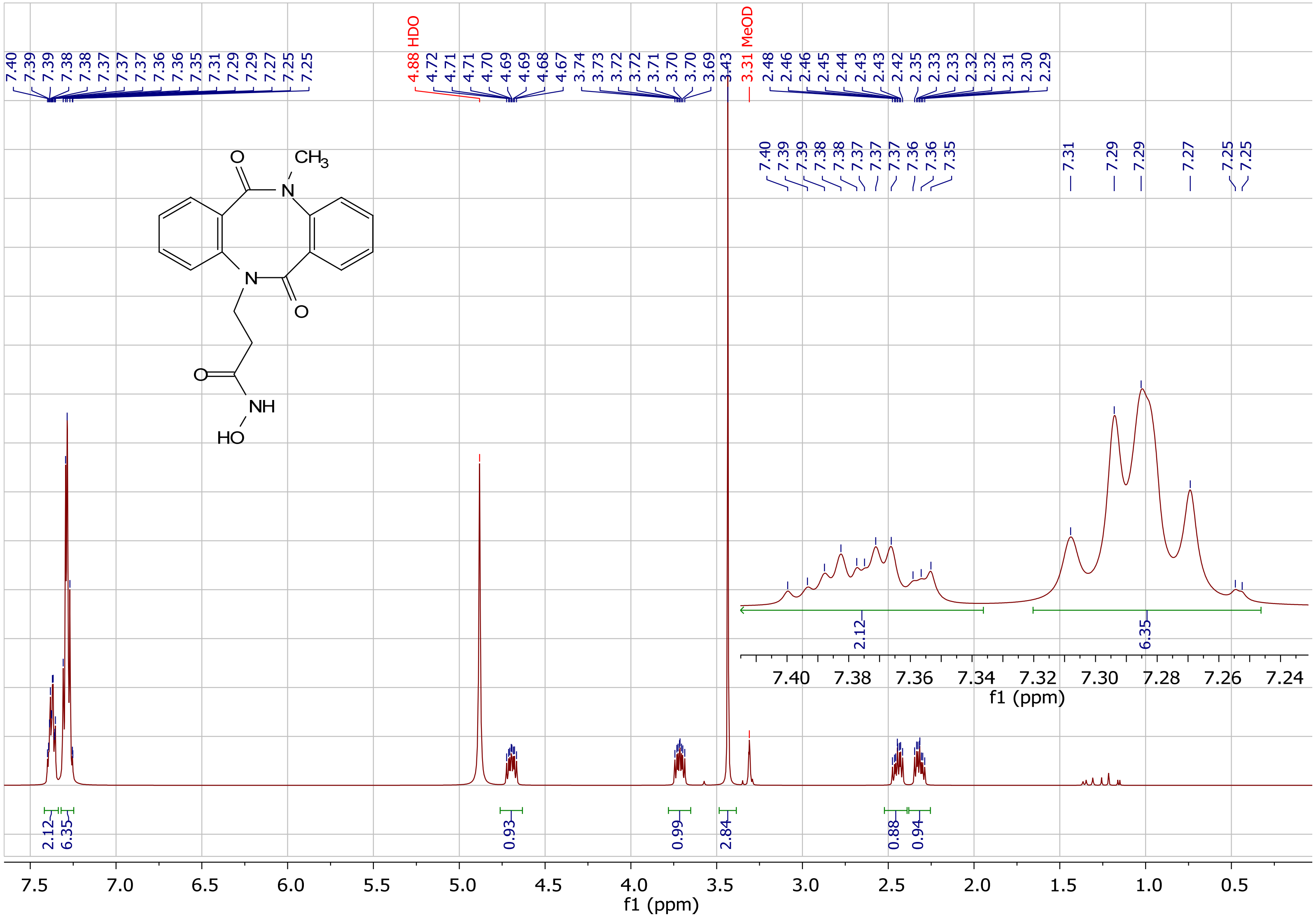

Figure S21. 1H NMR spectrum of *N*-hydroxy-3-(11-methyl-6,12-dioxo-11,12-dihydrodibenzo[*b*,*f*][1,5]diazocin-5(6*H*)-yl)propanamide (**7b**).

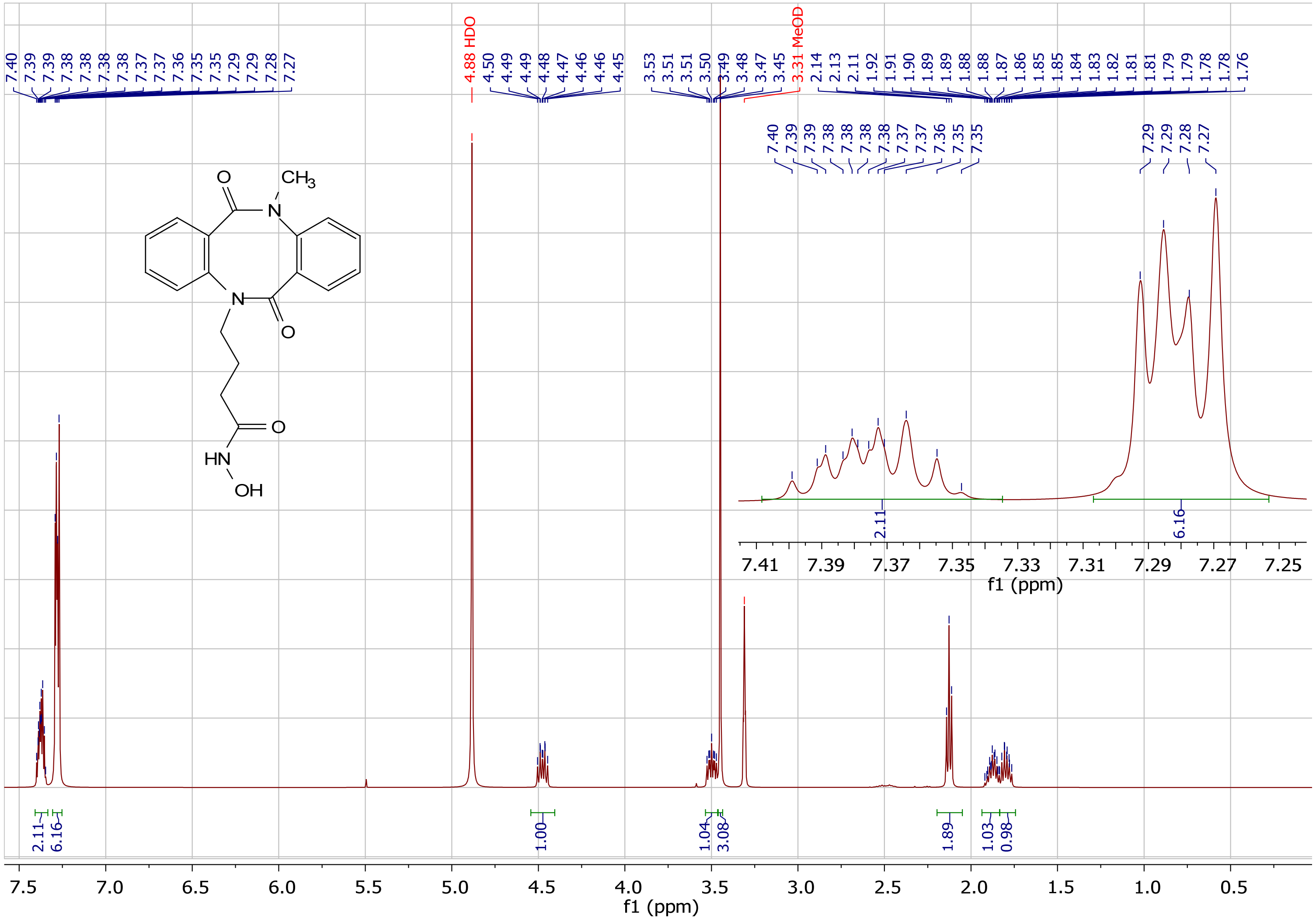

Figure S22. 1H NMR spectrum of *N*-hydroxy-4-(11-methyl-6,12-dioxo-11,12-dihydrodibenzo[*b*,*f*][1,5]diazocin-5(6*H*)-yl)butanamide (**7c**).

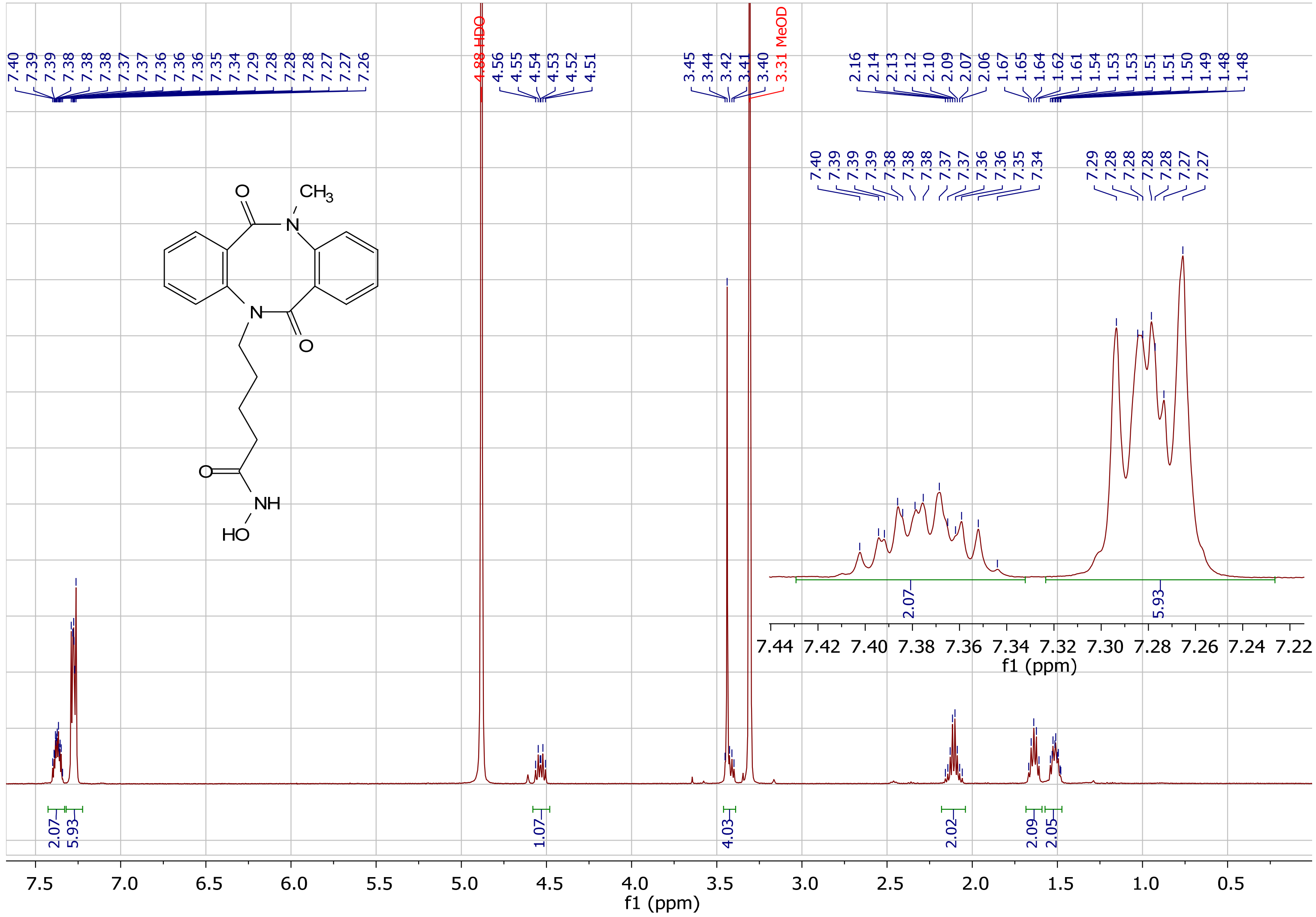

Figure S23. 1H NMR spectrum of *N*-hydroxy-5-(11-methyl-6,12-dioxo-11,12-dihydrodibenzo[*b*,*f*][1,5]diazocin-5(6*H*)-yl)pentanamide (**7d**).

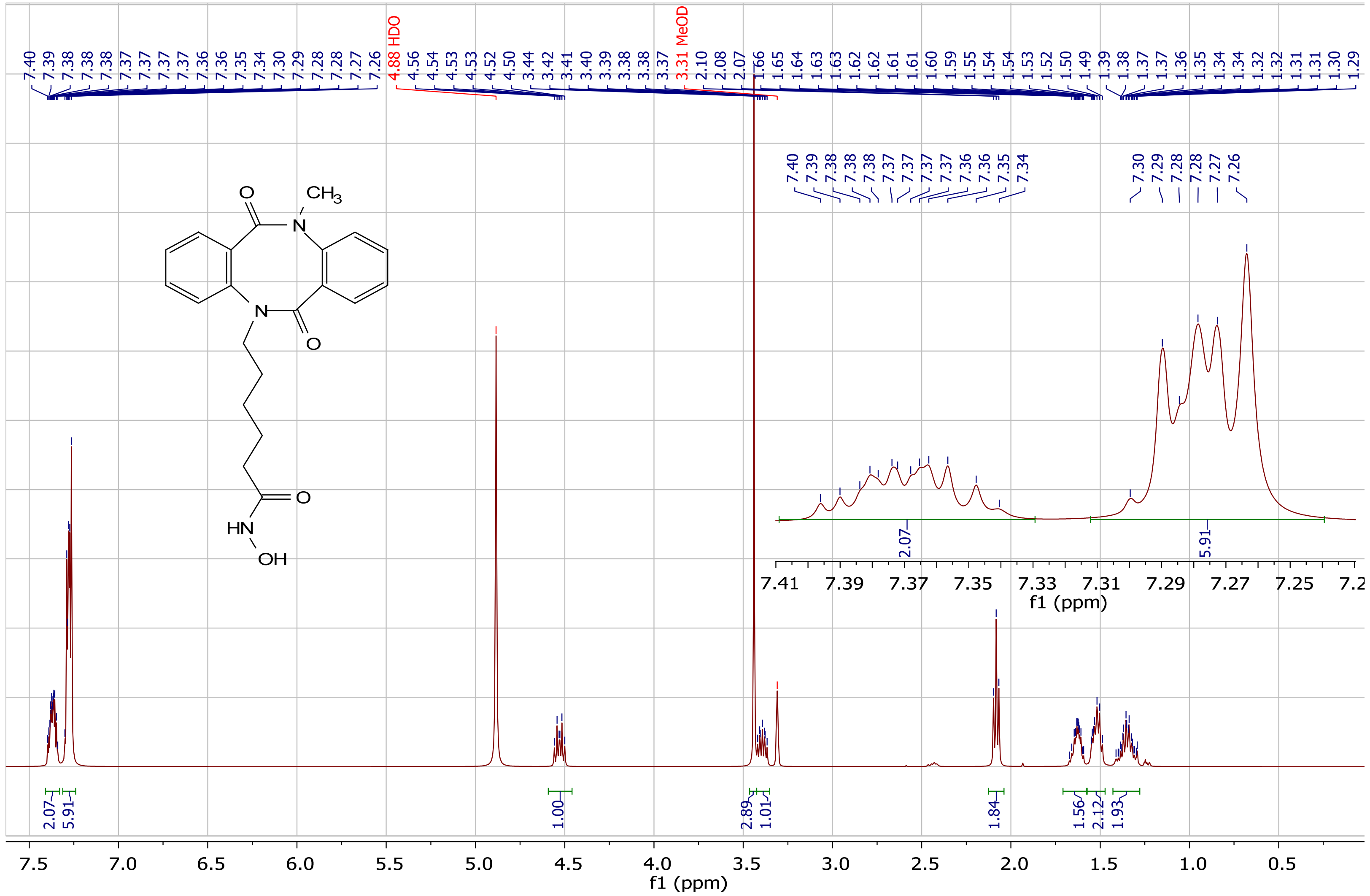

Figure S24. 1H NMR spectrum of *N*-hydroxy-6-(11-methyl-6,12-dioxo-11,12-dihydrodibenzo[*b*,*f*][1,5]diazocin-5(6*H*)-yl)hexanamide (**7e**).

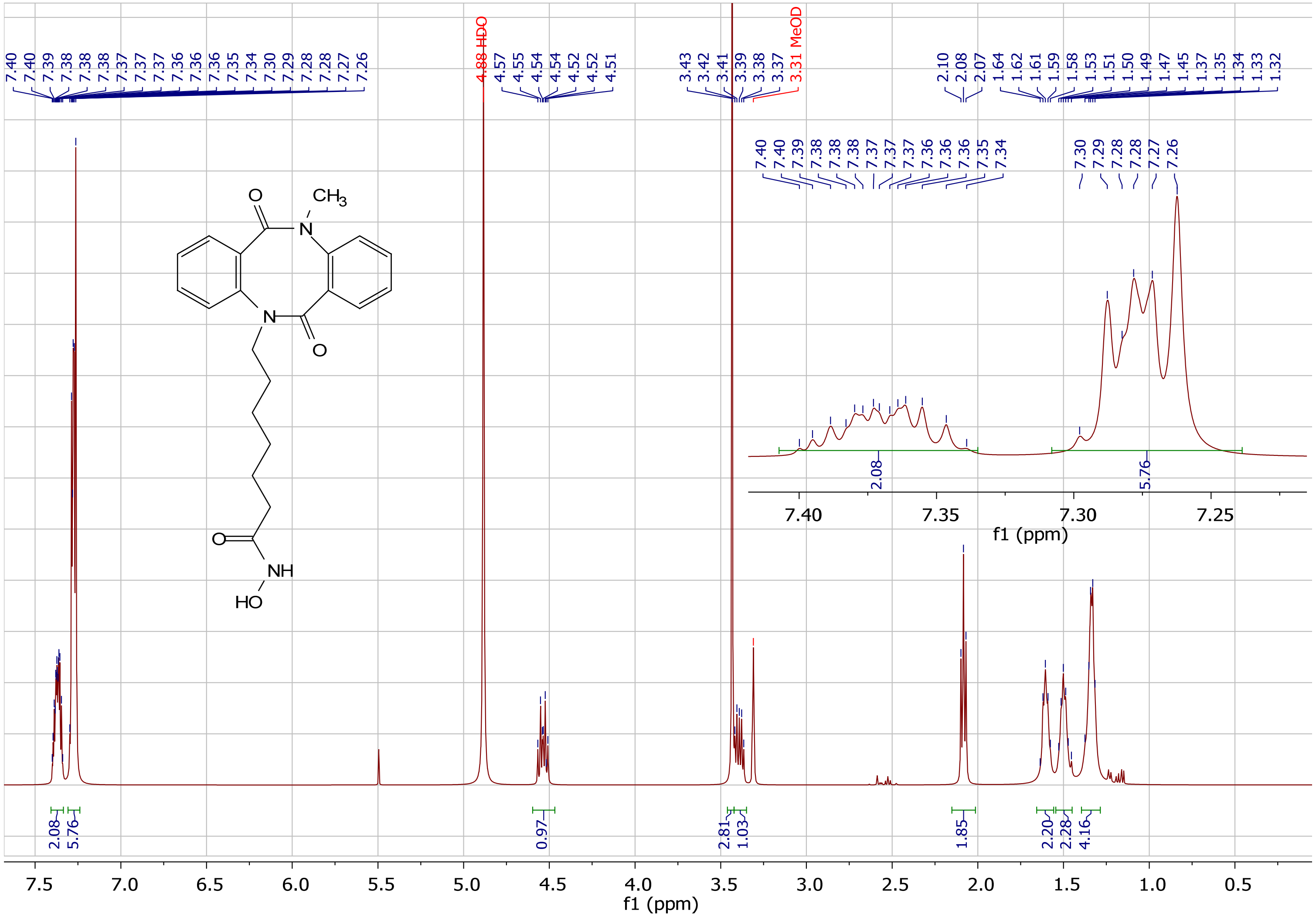

Figure S25. 1H NMR spectrum of *N*-hydroxy-7-(11-methyl-6,12-dioxo-11,12-dihydrodibenzo[*b*,*f*][1,5]diazocin-5(6*H*)-yl)heptanamide (**7f**).

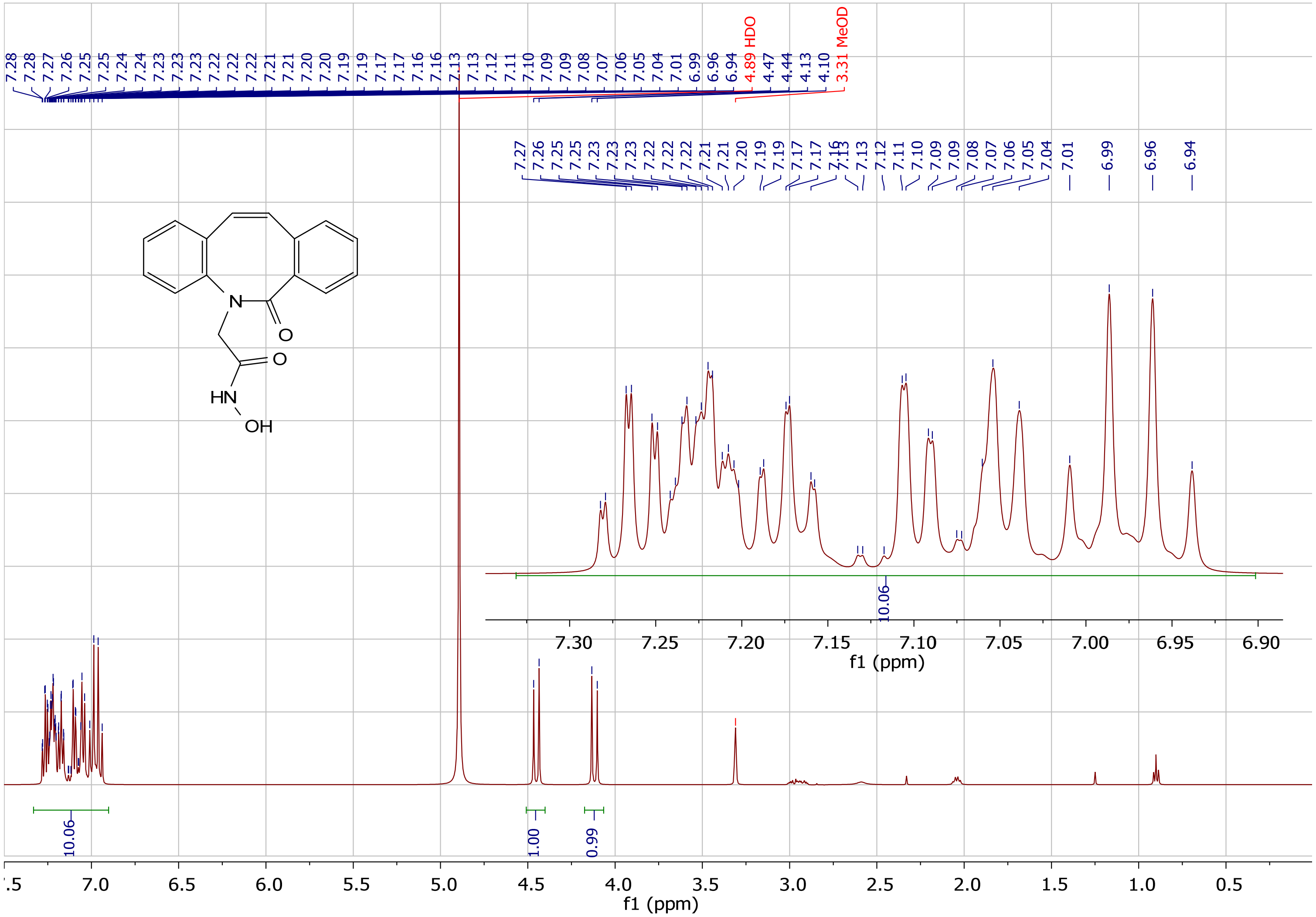

Figure S26. 1H NMR spectrum of *N*-hydroxy-2-(6-oxodibenzo[*b,f*]azocin-5(6*H*)-yl)acetamide (**7g**).

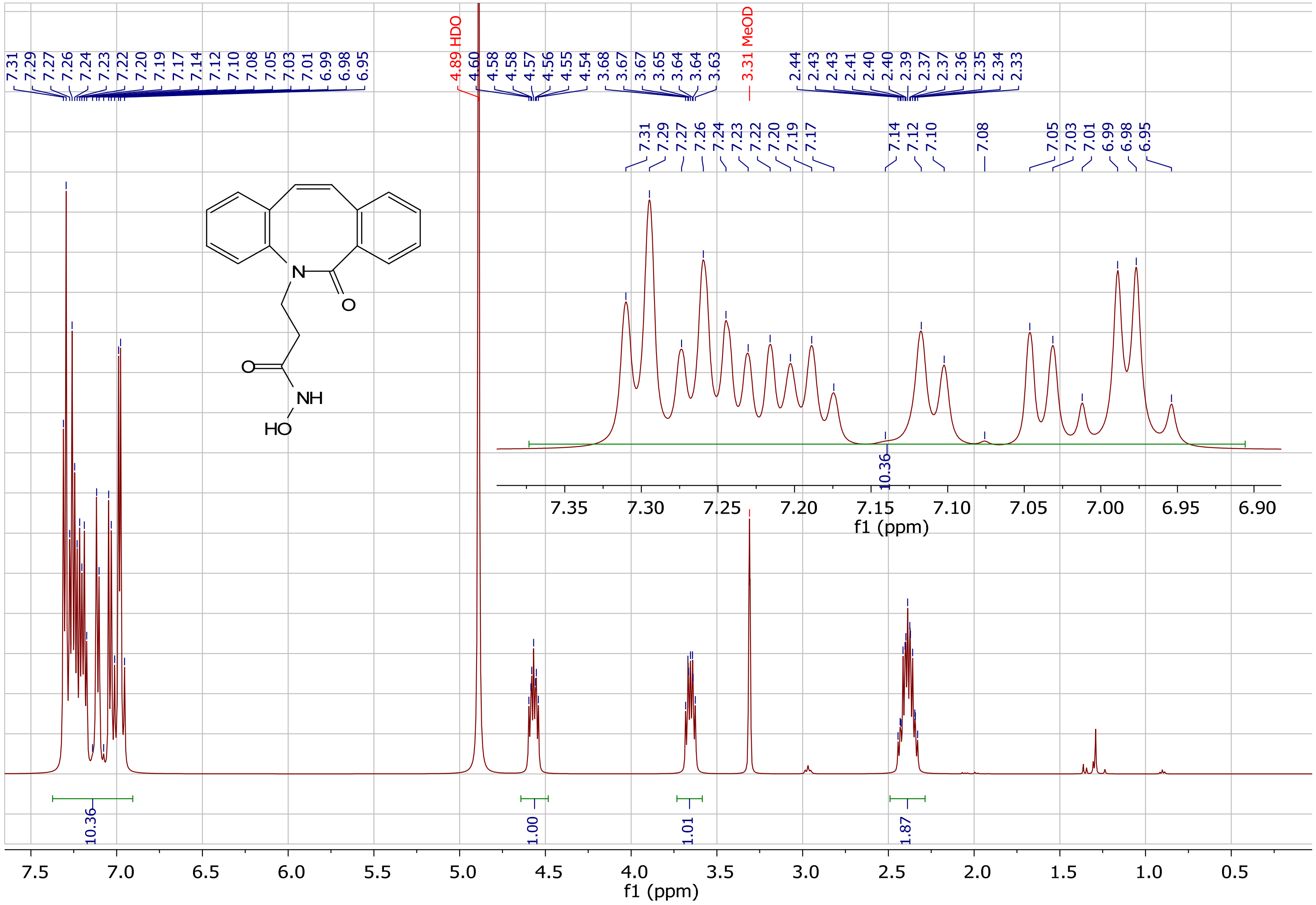

Figure S27. 1H NMR spectrum of *N*-hydroxy-3-(6-oxodibenzo[*b*,*f*]azocin-5(6*H*)-yl)propanamide (**7h**).

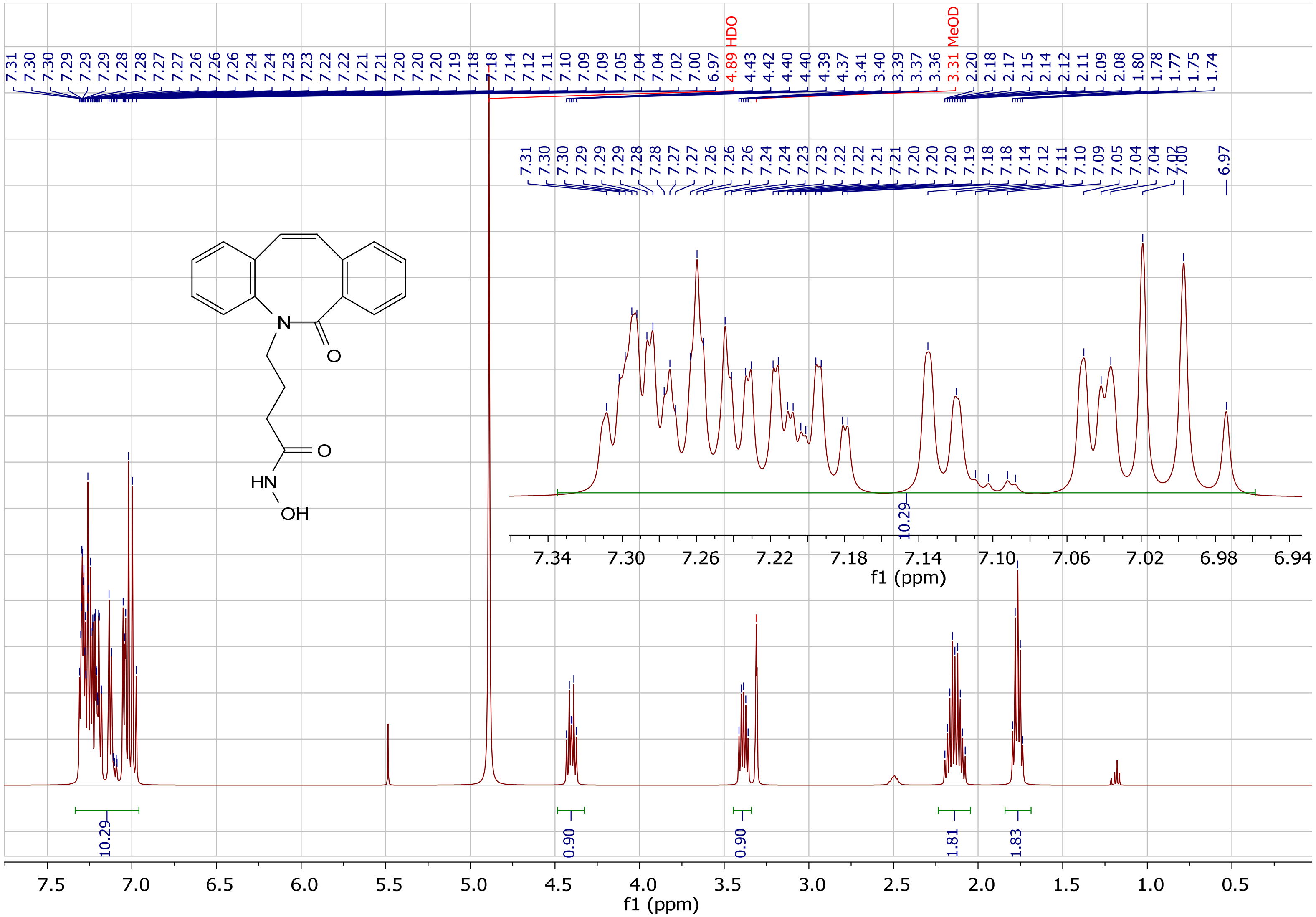

Figure S28. 1H NMR spectrum of *N*-hydroxy-4-(6-oxodibenzo[*b*,*f*]azocin-5(6*H*)-yl)butanamide (**7i**).

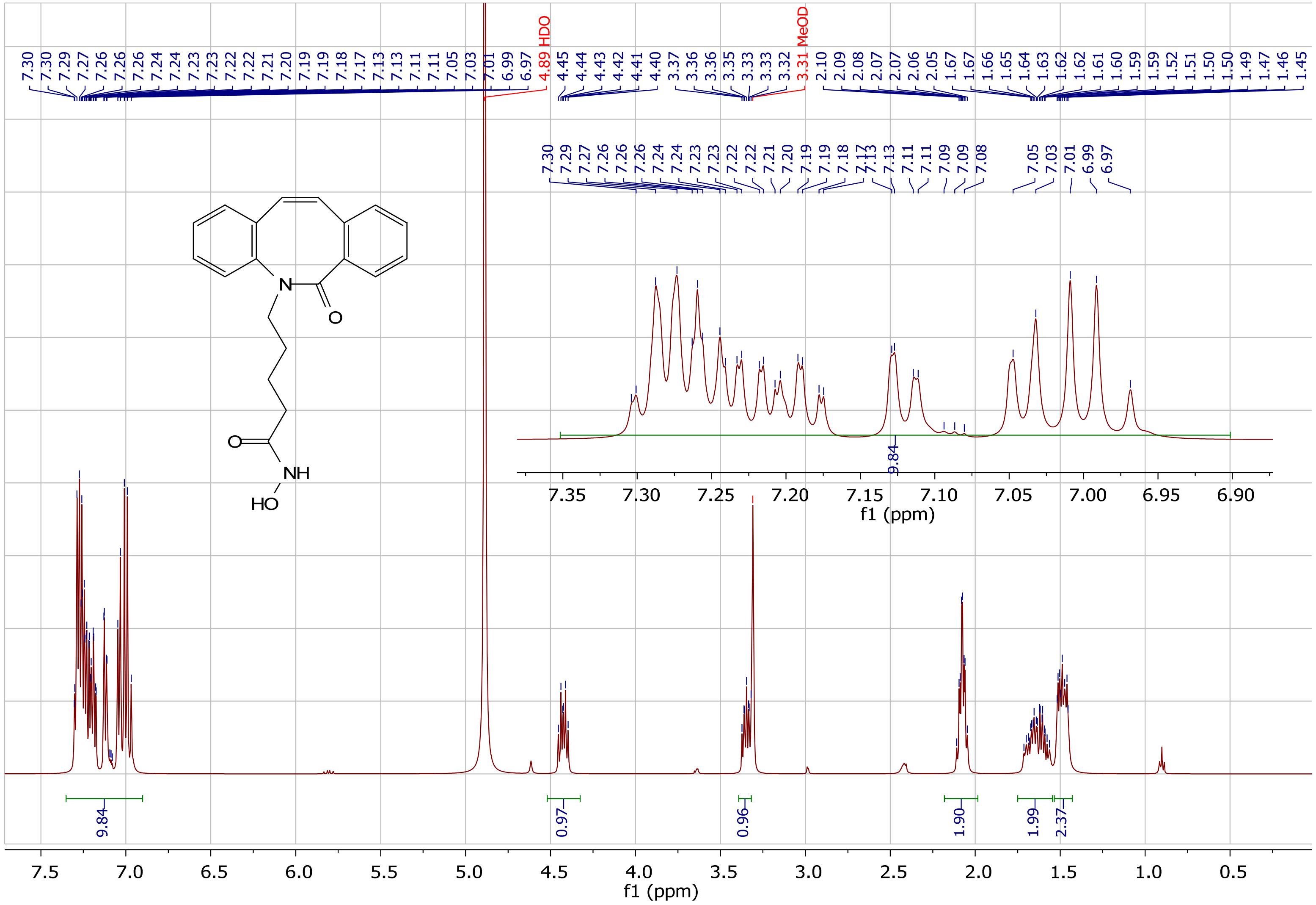

Figure S29. 1H NMR spectrum of *N*-hydroxy-5-(6-oxodibenzo[*b*,*f*]azocin-5(6*H*)-yl)pentanamide (**7j**).

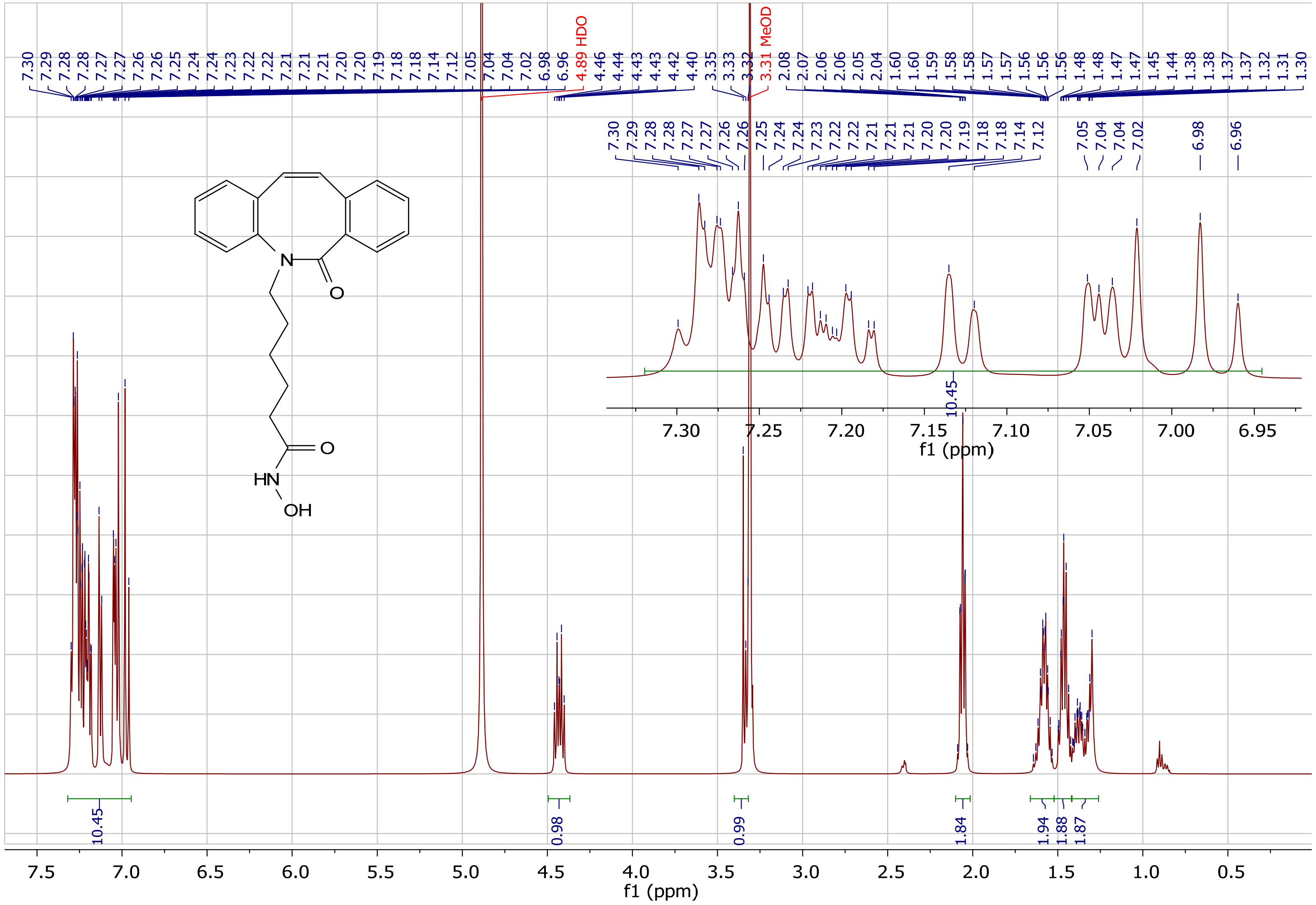

Figure S30. 1H NMR spectrum of *N*-hydroxy-6-(6-oxodibenzo[*b*,*f*]azocin-5(6*H*)-yl)hexanamide (**7k**).

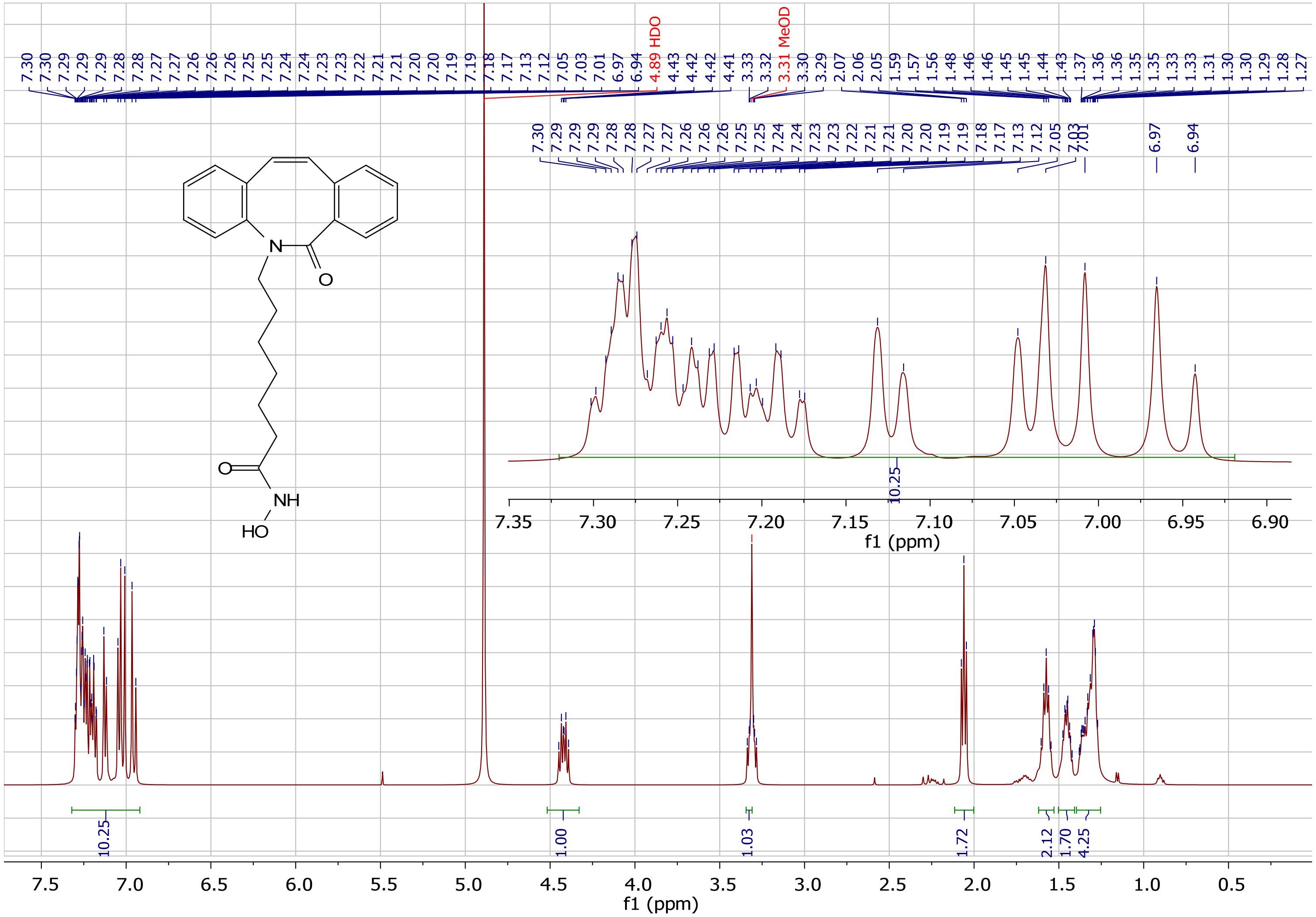

Figure S31. 1H NMR spectrum of *N*-hydroxy-7-(6-oxodibenzo[*b,f*]azocin-5(6*H*)-yl)heptanamide (**7l**).

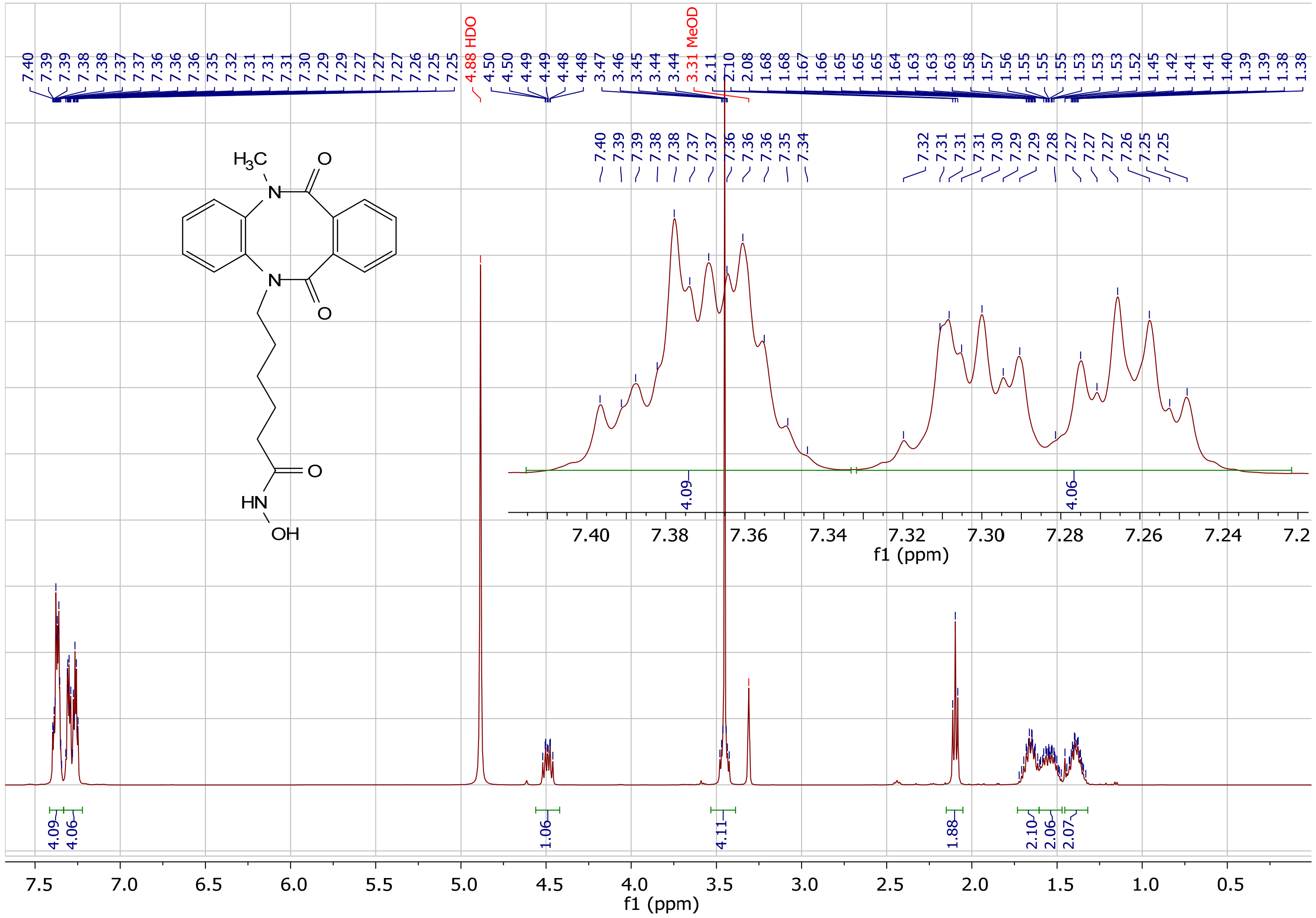

Figure S32. 1H NMR spectrum of *N*-hydroxy-6-(12-methyl-6,11-dioxo-11,12-dihydrodibenzo[*b*,*f*][1,4]diazocin-5(6*H*)-yl)hexanamide (**7m**).

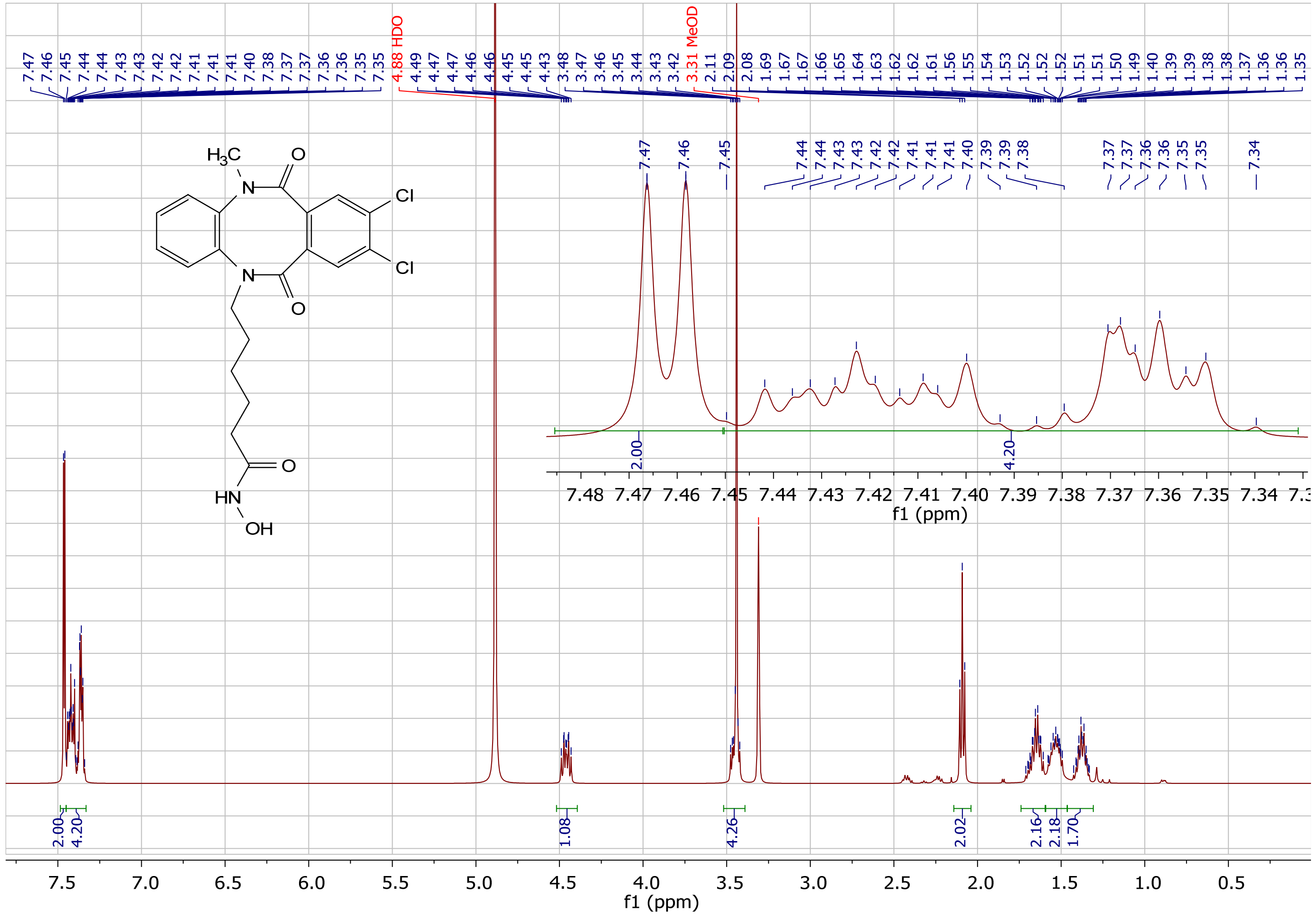

Figure S33. 1H NMR spectrum of *N*-hydroxy-6-(8,9-dichloro-12-methyl-6,11-dioxo-11,12-dihydrodibenzo[*b*,*f*][1,4]diazocin-5(6*H*)-yl)hexanamide (**7n**).

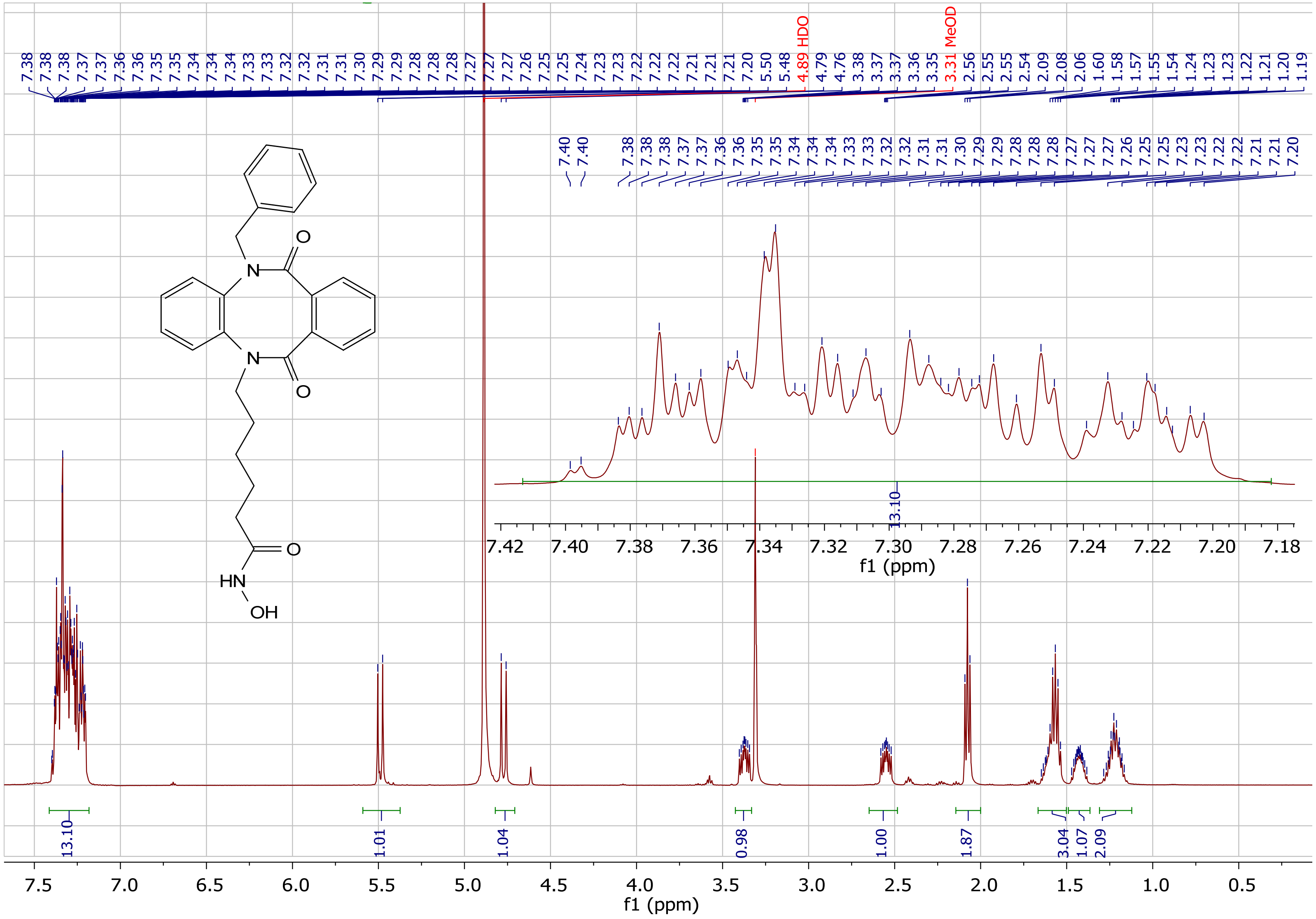

Figure S34. 1H NMR spectrum of *N*-hydroxy-6-(12-benzyl-6,11-dioxo-11,12-dihydrodibenzo[*b*,*f*][1,4]diazocin-5(6*H*)-yl)hexanamide (**7o**).

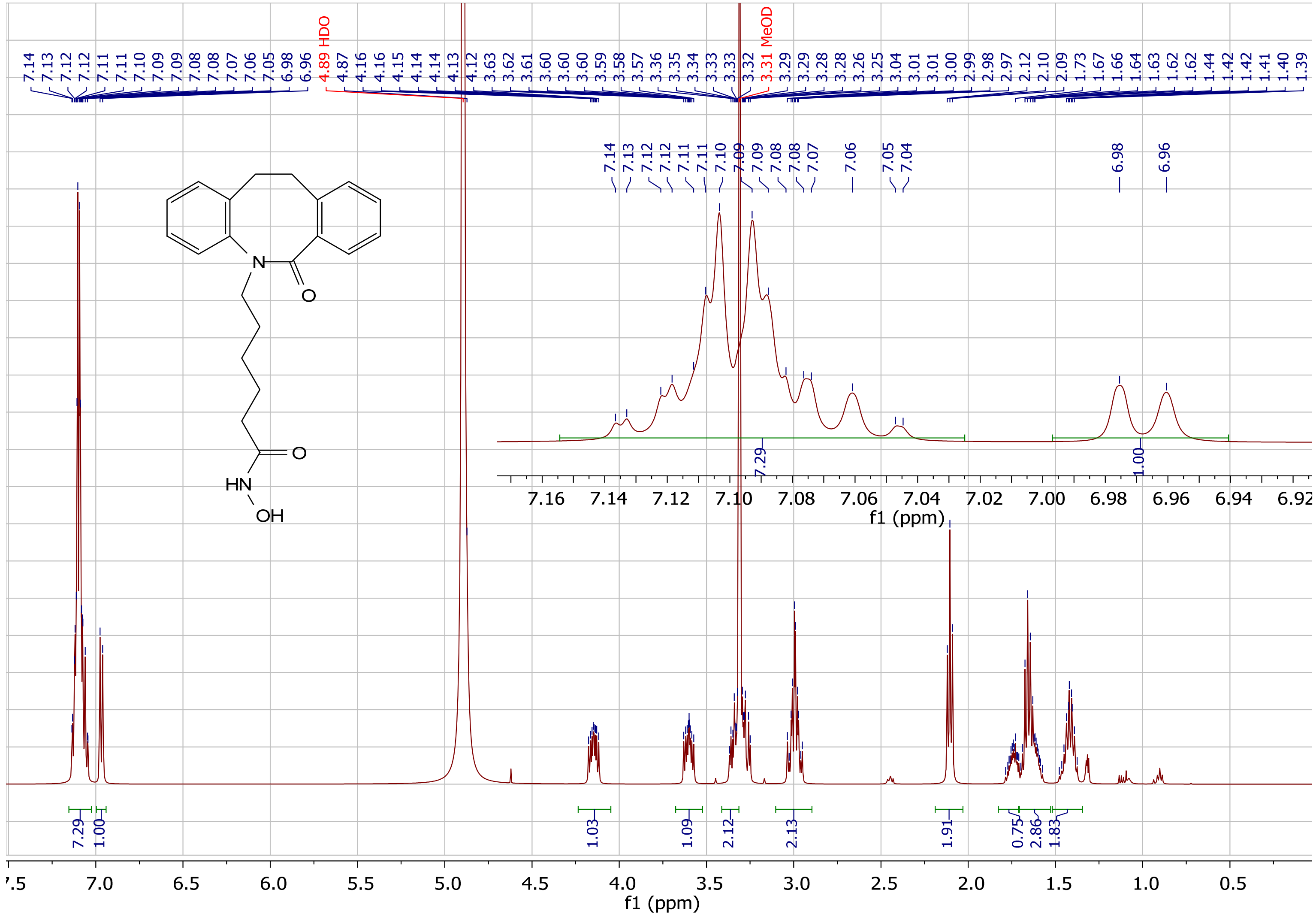

Figure S35. 1H NMR spectrum of *N*-hydroxy-6-(6-oxo-11,12-dihydrodibenzo[*b*,*f*]azocin-5(6*H*)-yl)hexanamide (**7p**).

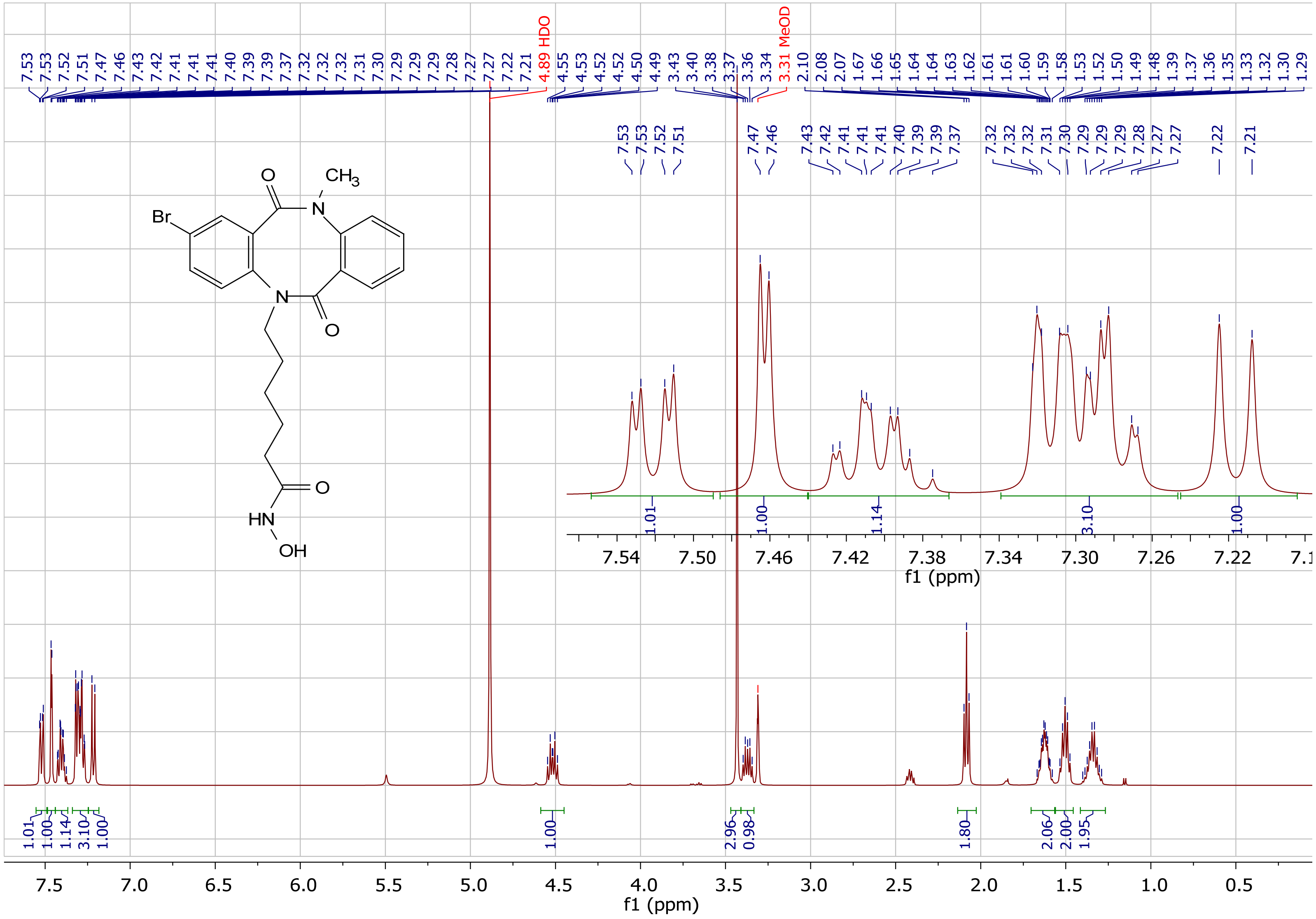

Figure S36. 1H NMR spectrum of *N*-hydroxy-6-(2-bromo-11-methyl-6,12-dioxo-11,12-dihydrodibenzo[*b*,*f*][1,5]diazocin-5(6*H*)-yl)hexanamide (**7r**).

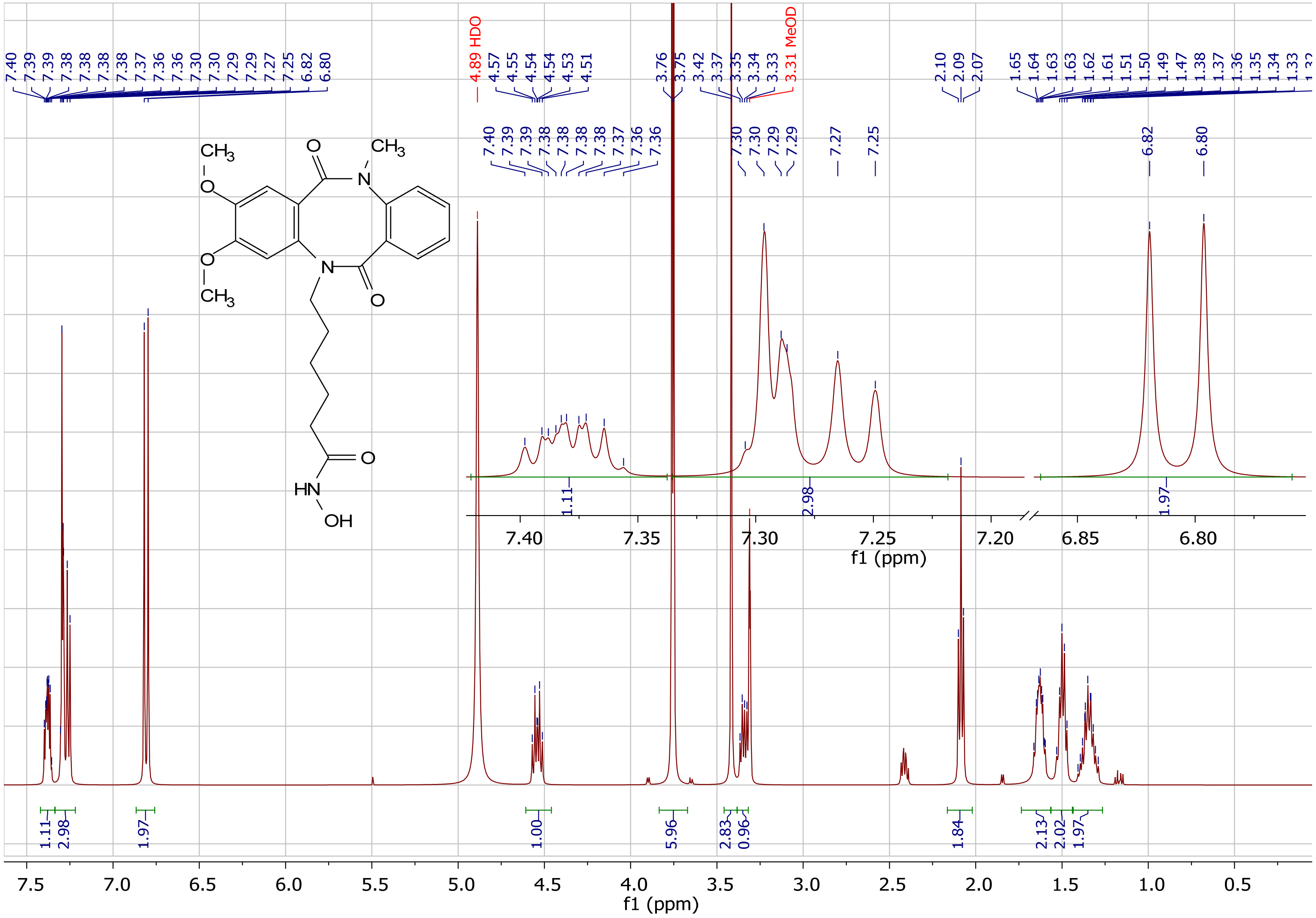

Figure S37. 1H NMR spectrum of *N*-hydroxy-6-(2,3-dimethoxy-11-methyl-6,12-dioxo-11,12-dihydrodibenzo[*b*,*f*][1,5]diazocin-5(6*H*)-yl)hexanamide (**7s**).

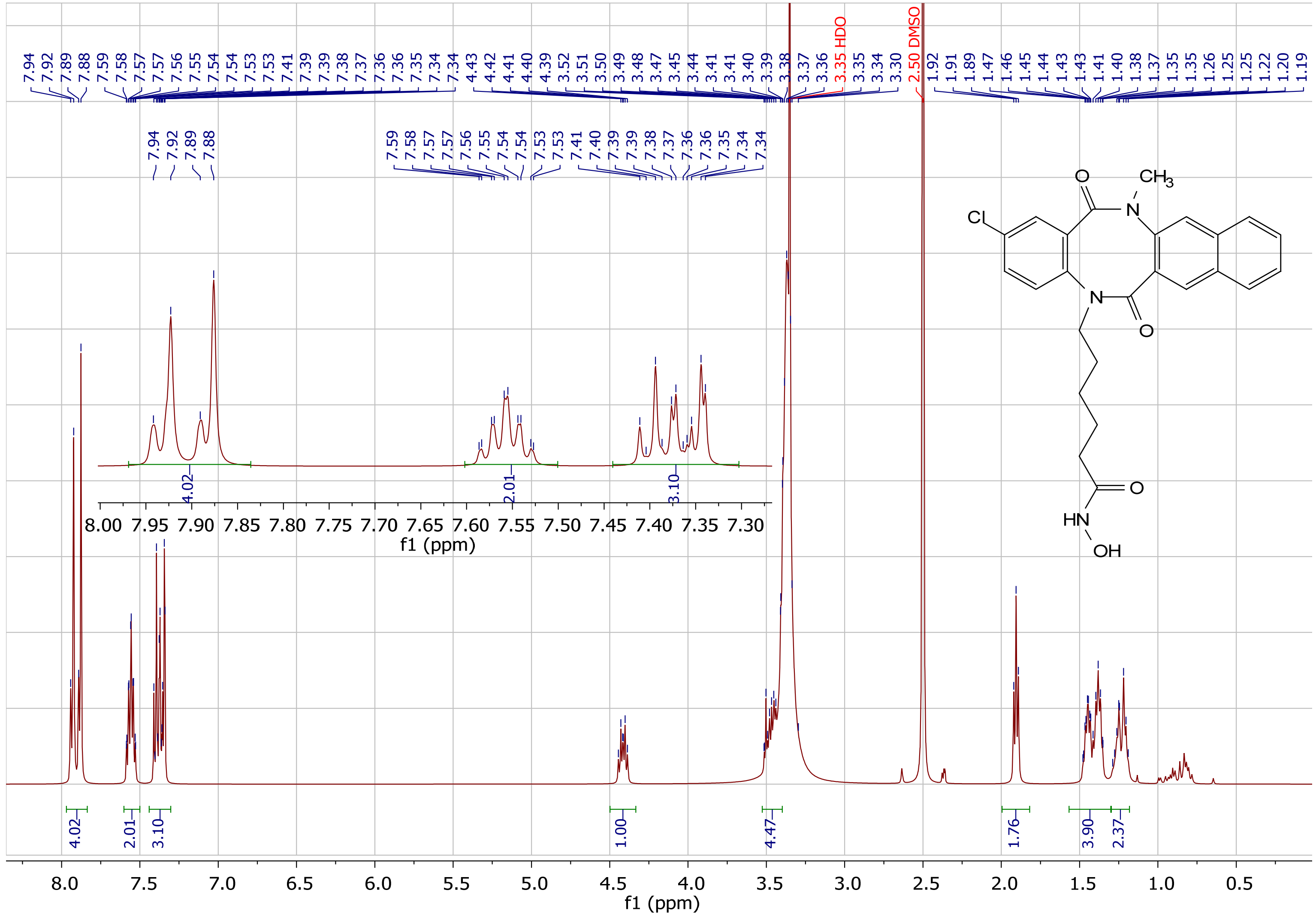

Figure S38. 1H NMR spectrum of *N*-hydroxy-6-(5-methyl-6,14-dioxo-5,14-dihydrobenzonaphtho[2,3-*f*][1,5]diazocin-13(6*H*)-yl)hexanamide (**7t**).

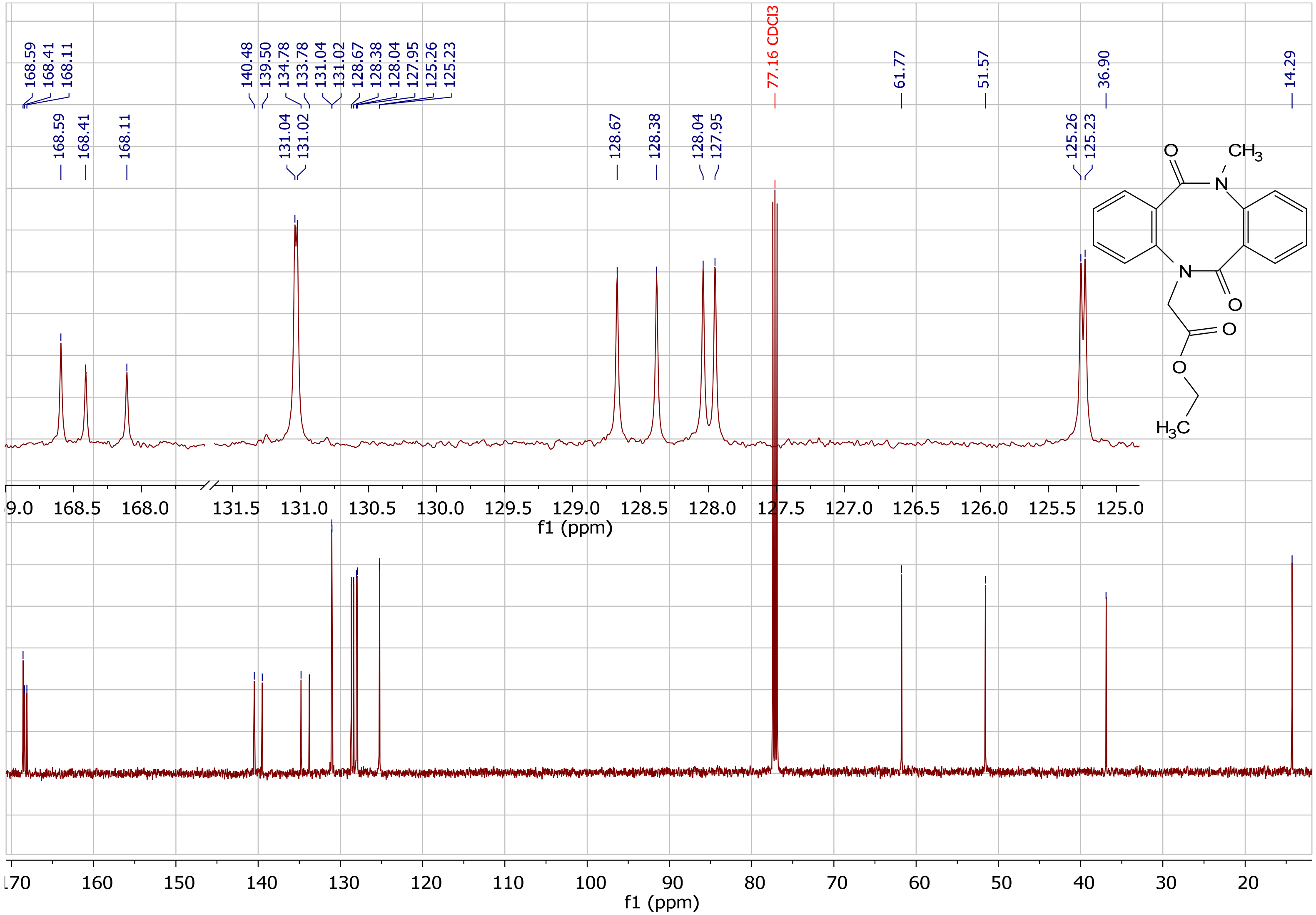

Figure S39. 13C NMR spectrum of ethyl 2-(11-methyl-6,12-dioxo-11,12-dihydrodibenzo[*b*,*f*][1,5]diazocin-5(6*H*)-yl)acetate (**10a**).

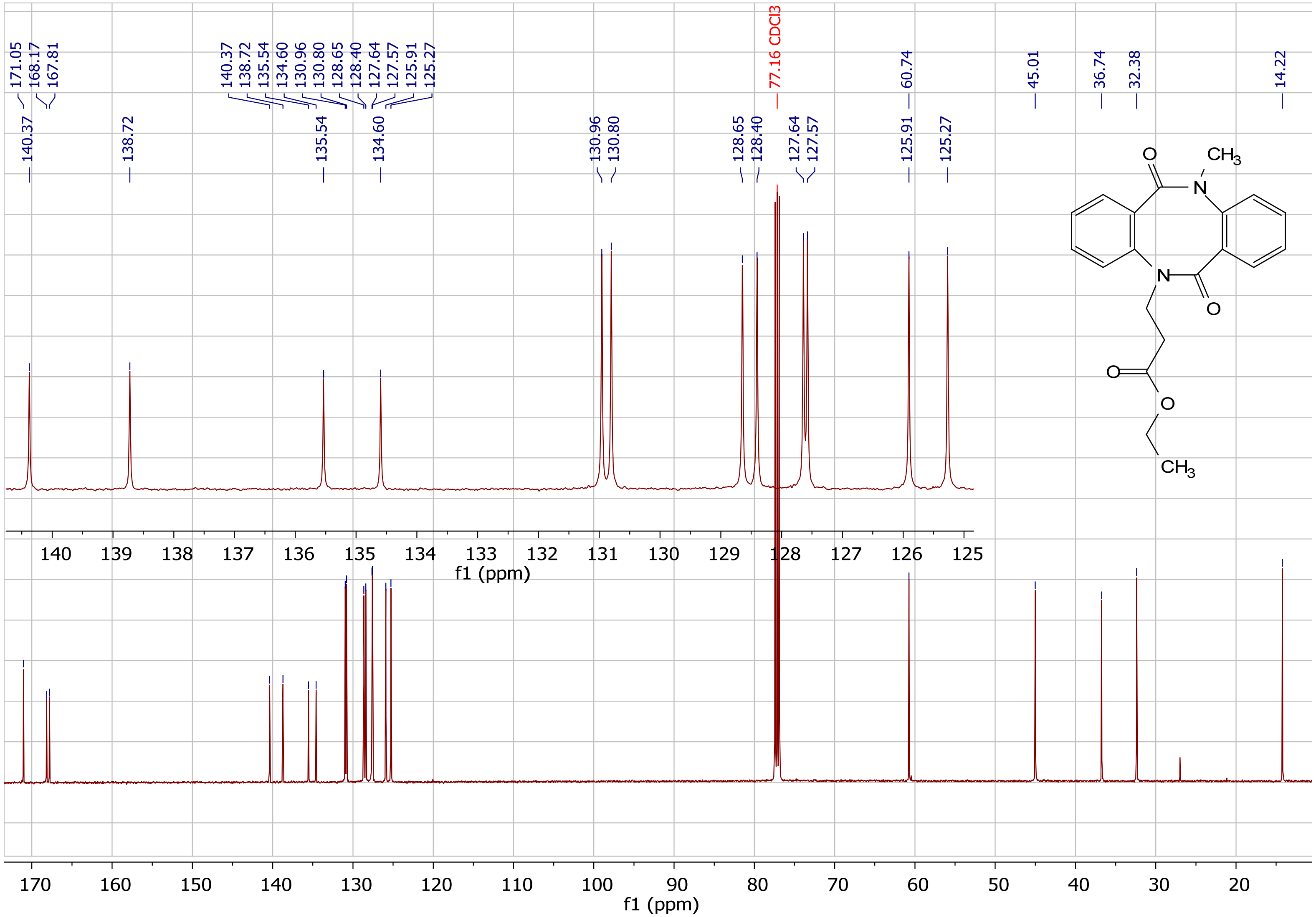

Figure S40. 13C NMR spectrum of ethyl 3-(11-methyl-6,12-dioxo-11,12-dihydrodibenzo[*b*,*f*][1,5]diazocin-5(6*H*)-yl)propanoate (**10b**).

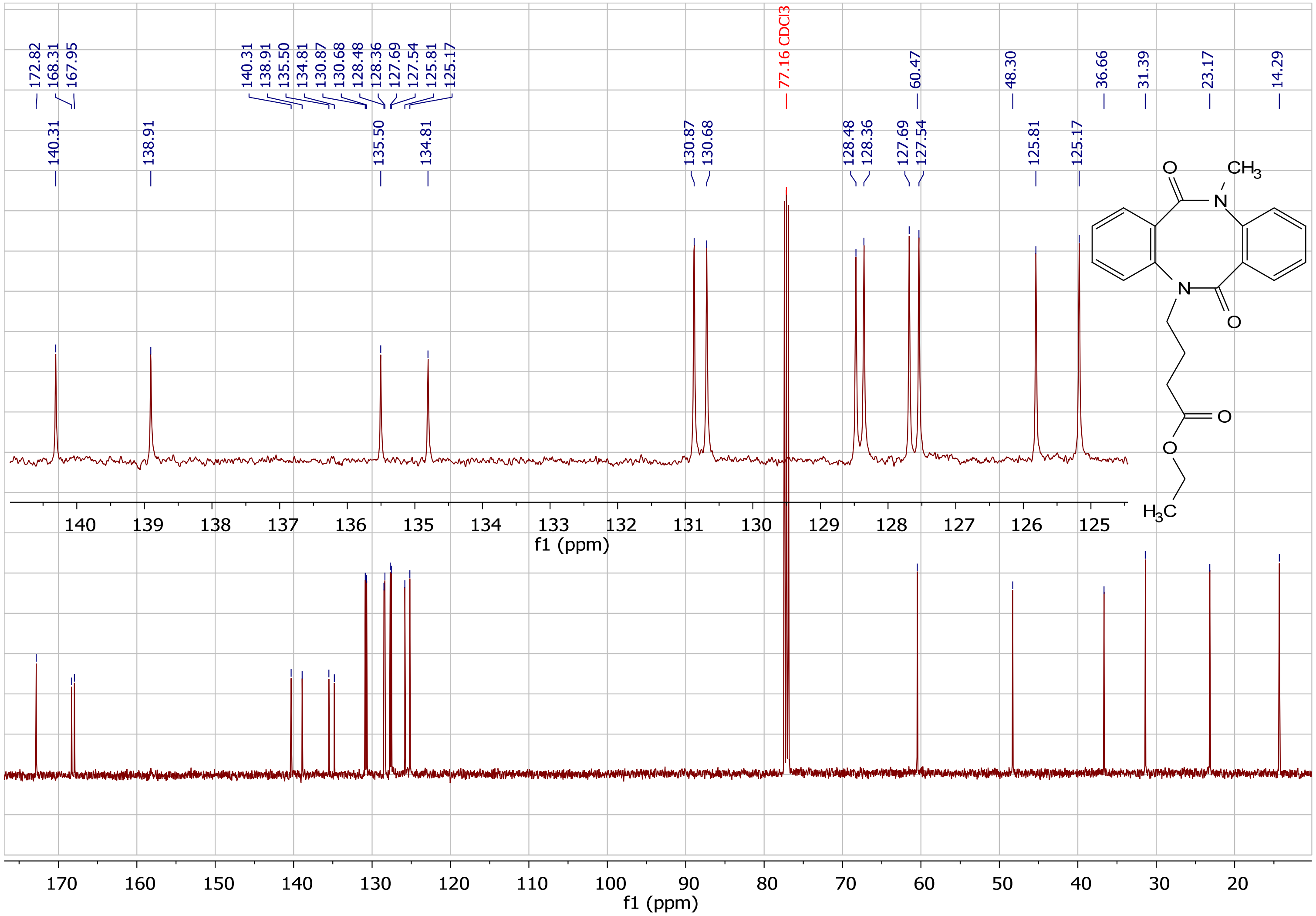

Figure S41. 13C NMR spectrum of ethyl 4-(11-methyl-6,12-dioxo-11,12-dihydrodibenzo[*b*,*f*][1,5]diazocin-5(6*H*)-yl)butanoate (**10c**).

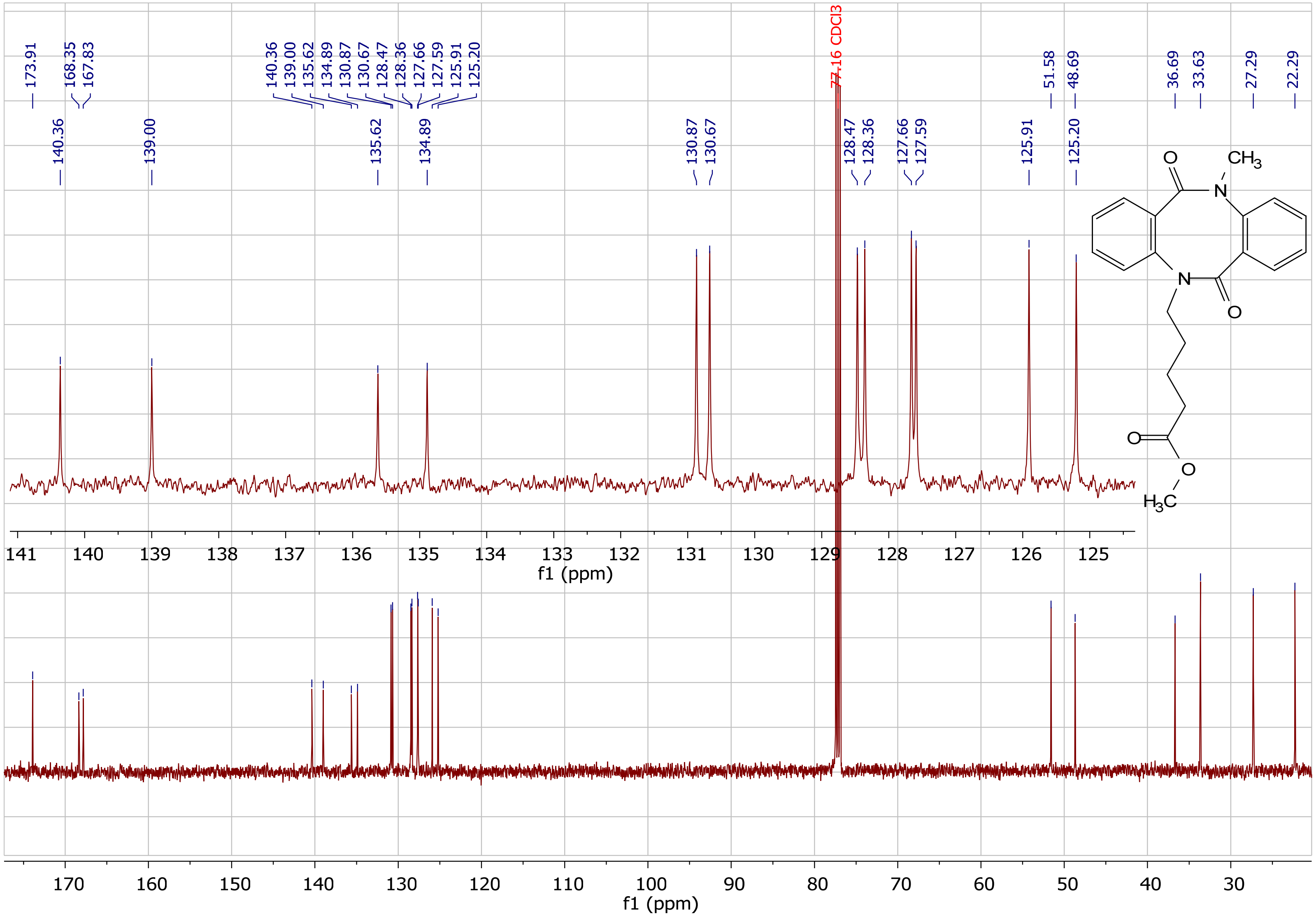

Figure S42. 13C NMR spectrum of methyl 5-(11-methyl-6,12-dioxo-11,12-dihydrodibenzo[*b*,*f*][1,5]diazocin-5(6*H*)-yl)pentanoate (**10d**).

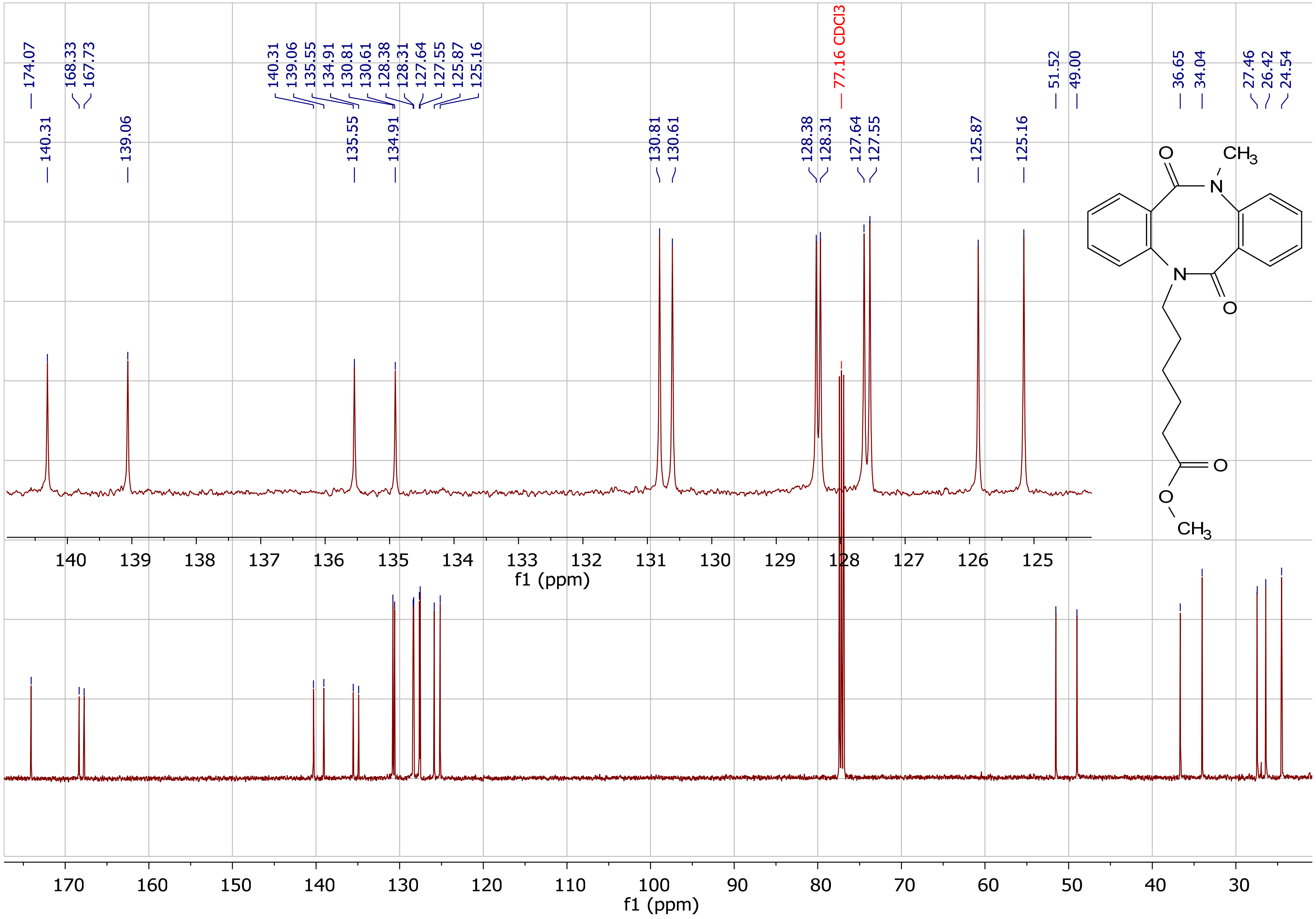

Figure S43. 13C NMR spectrum of methyl 6-(11-methyl-6,12-dioxo-11,12-dihydrodibenzo[*b*,*f*][1,5]diazocin-5(6*H*)-yl)hexanoate (**10e**).

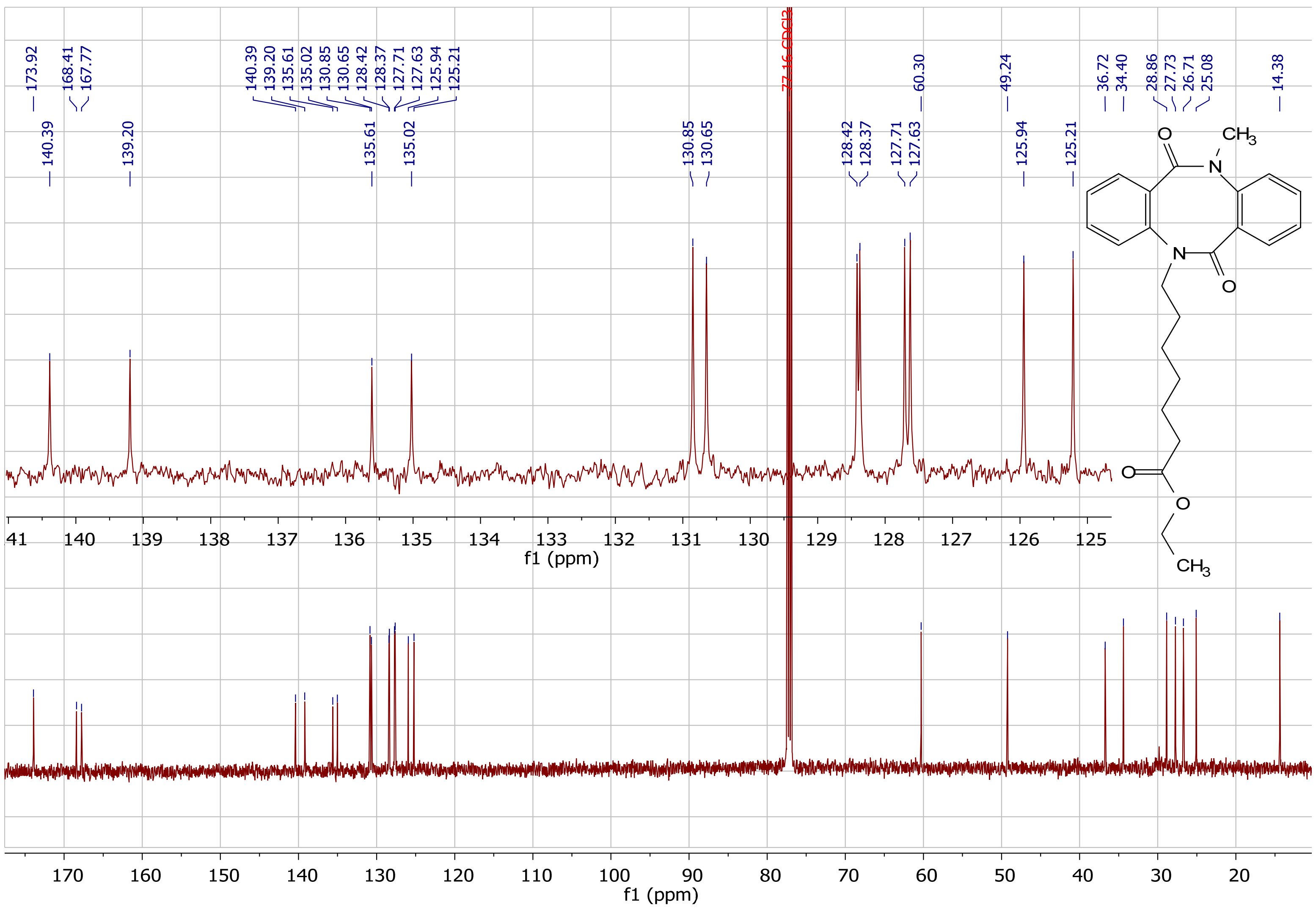

Figure S44. 13C NMR spectrum of ethyl 7-(11-methyl-6,12-dioxo-11,12-dihydrodibenzo[*b*,*f*][1,5]diazocin-5(6*H*)-yl)heptanoate (**10f**).

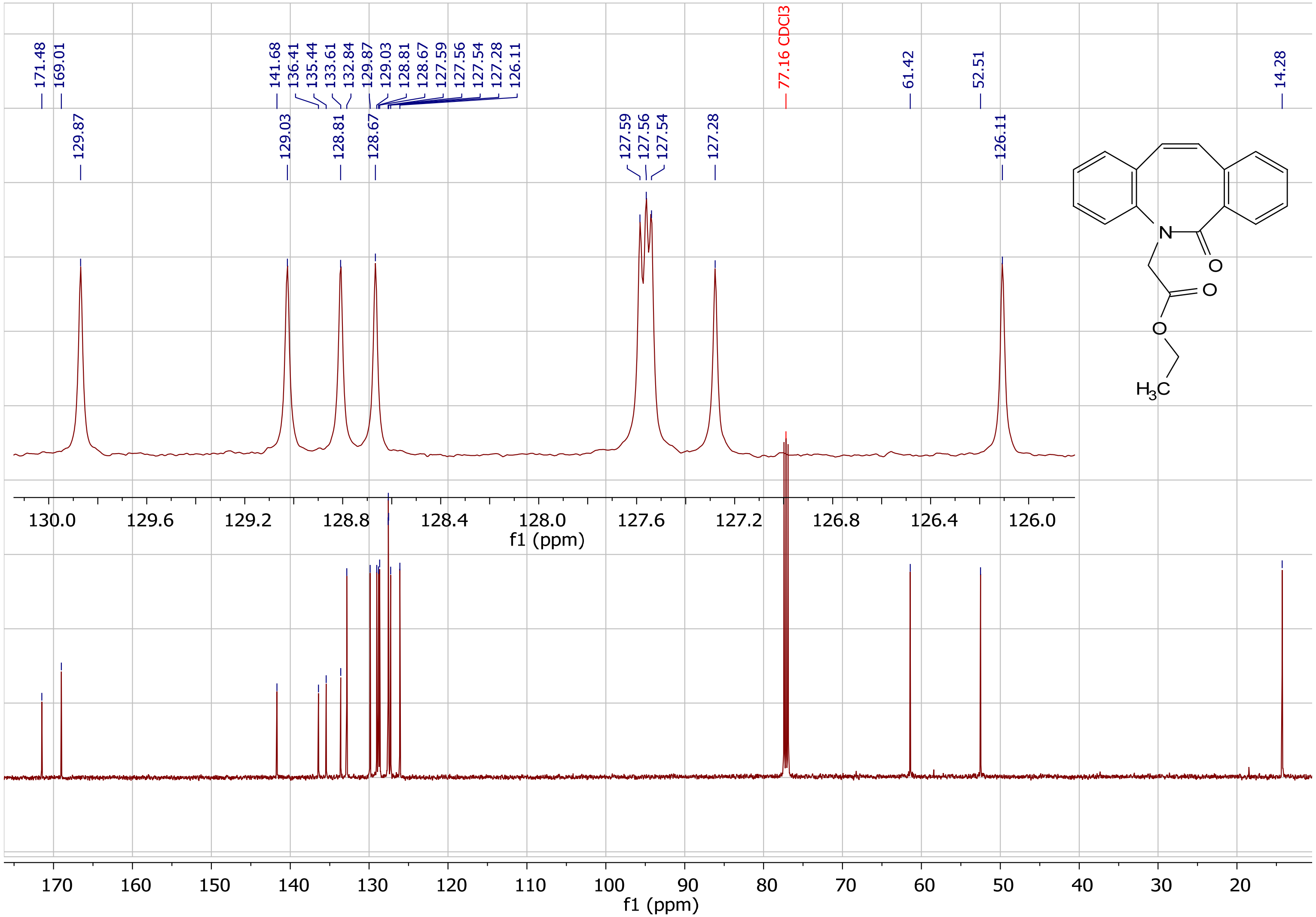

Figure S45. 13C NMR spectrum of ethyl 2-(6-oxodibenzo[*b,f*]azocin-5(6*H*)-yl)acetate (**10g**).

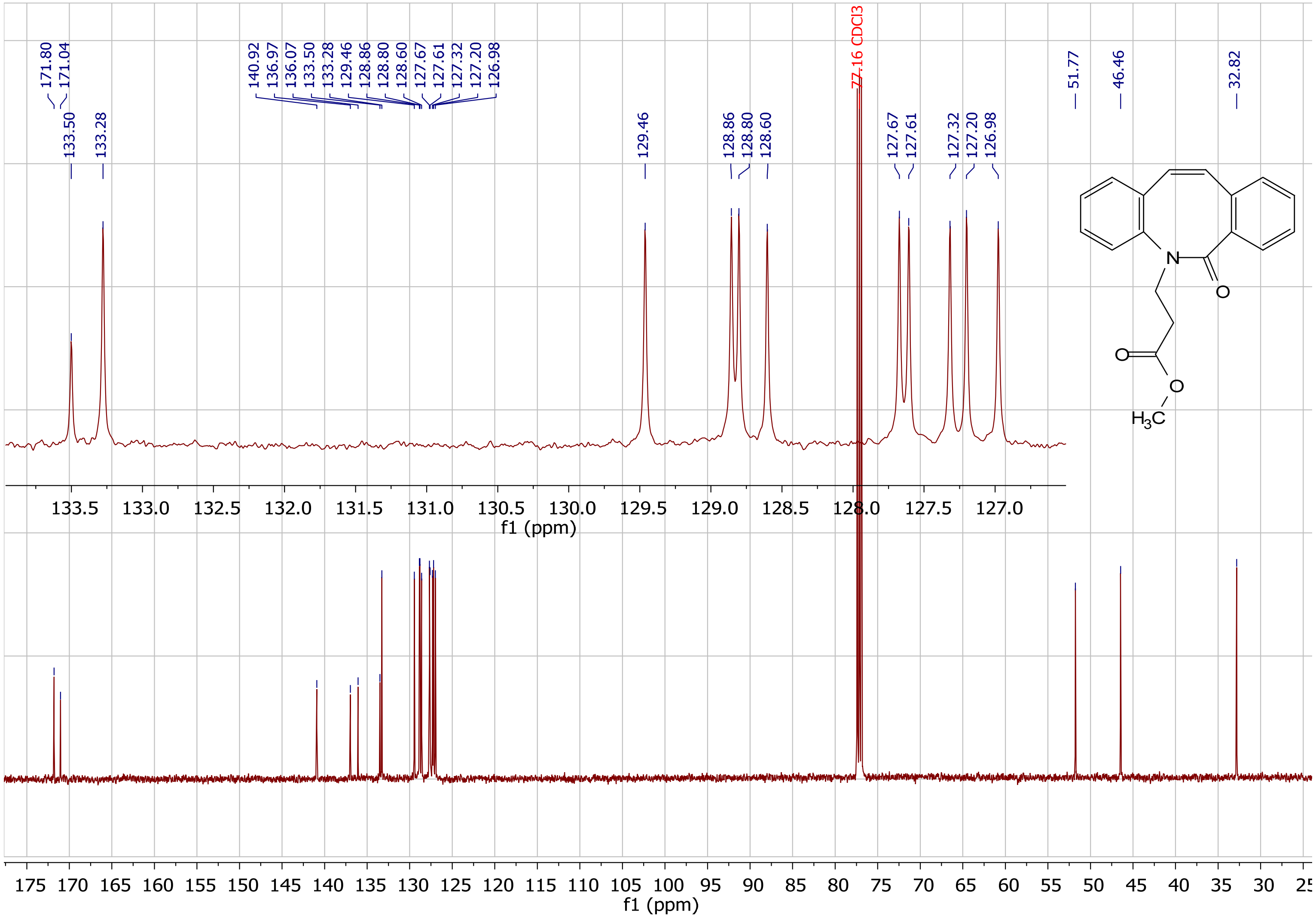

Figure S46. 13C NMR spectrum of methyl 3-(6-oxodibenzo[*b*,*f*]azocin-5(6*H*)-yl)propanoate (**10h**).

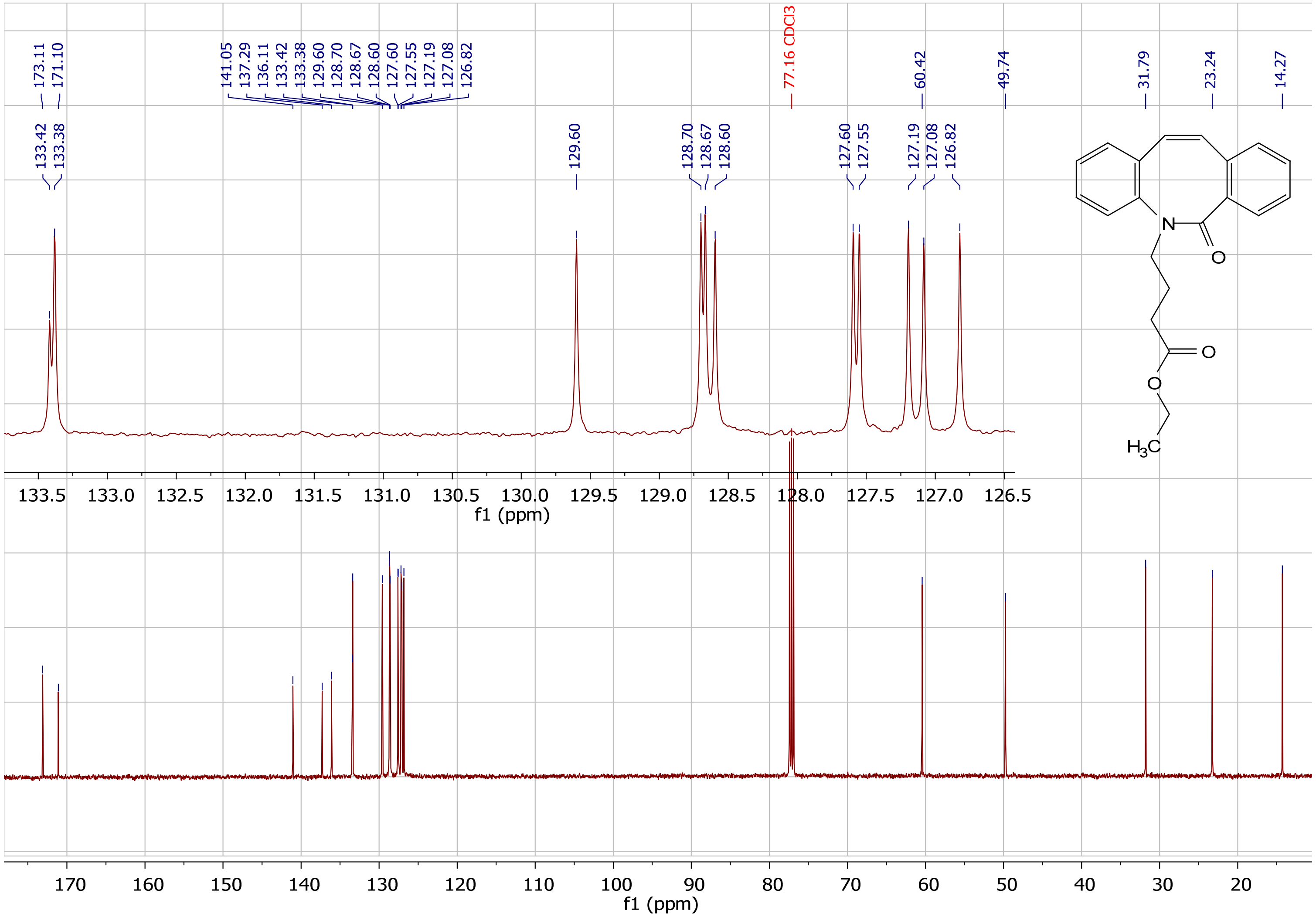

Figure S47. 13C NMR spectrum of ethyl 4-(6-oxodibenzo[*b*,*f*]azocin-5(6*H*)-yl)butanoate (**10i**).

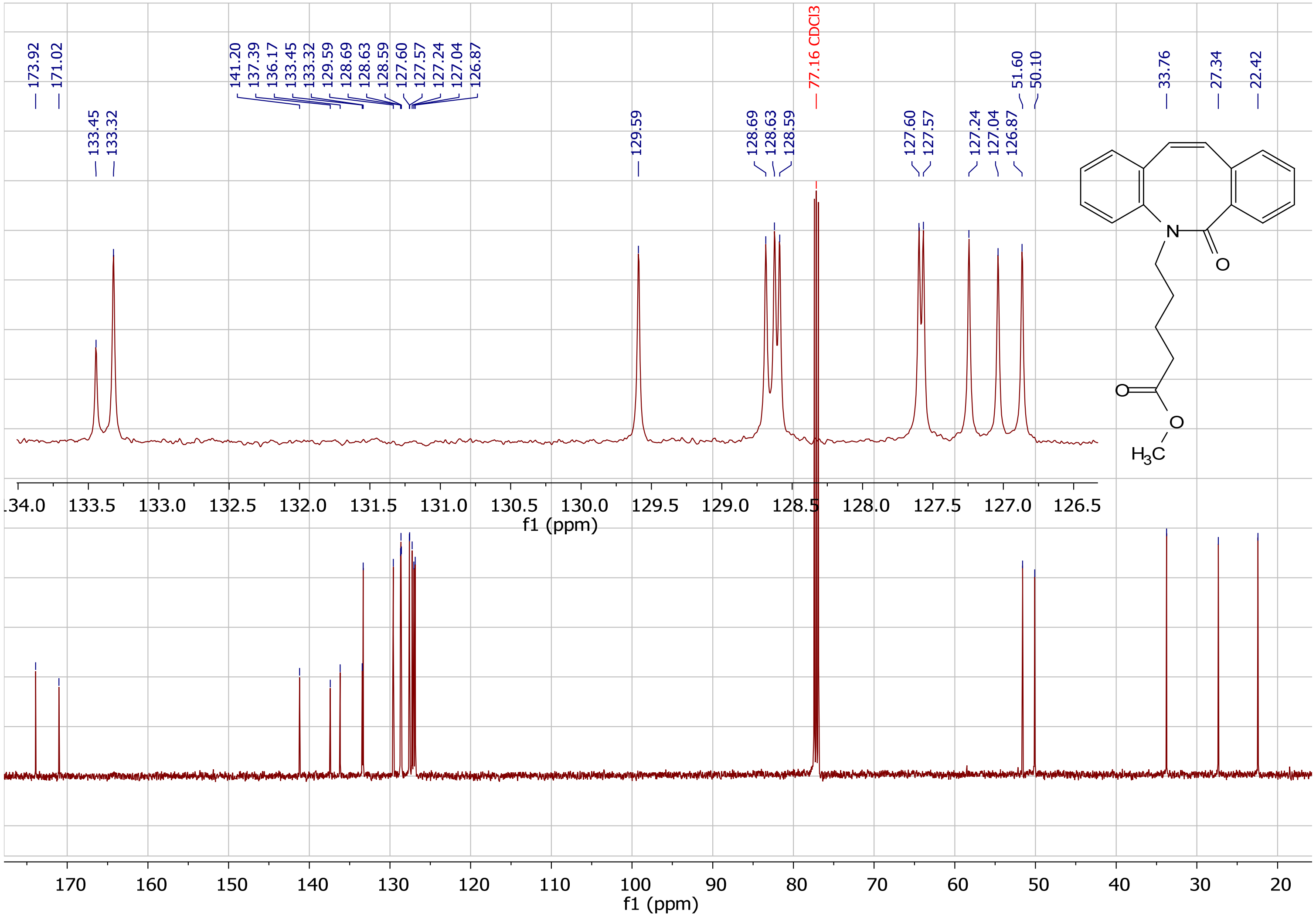

Figure S48. 13C NMR spectrum of methyl 5-(6-oxodibenzo[*b*,*f*]azocin-5(6*H*)-yl)pentanoate (**10j**).

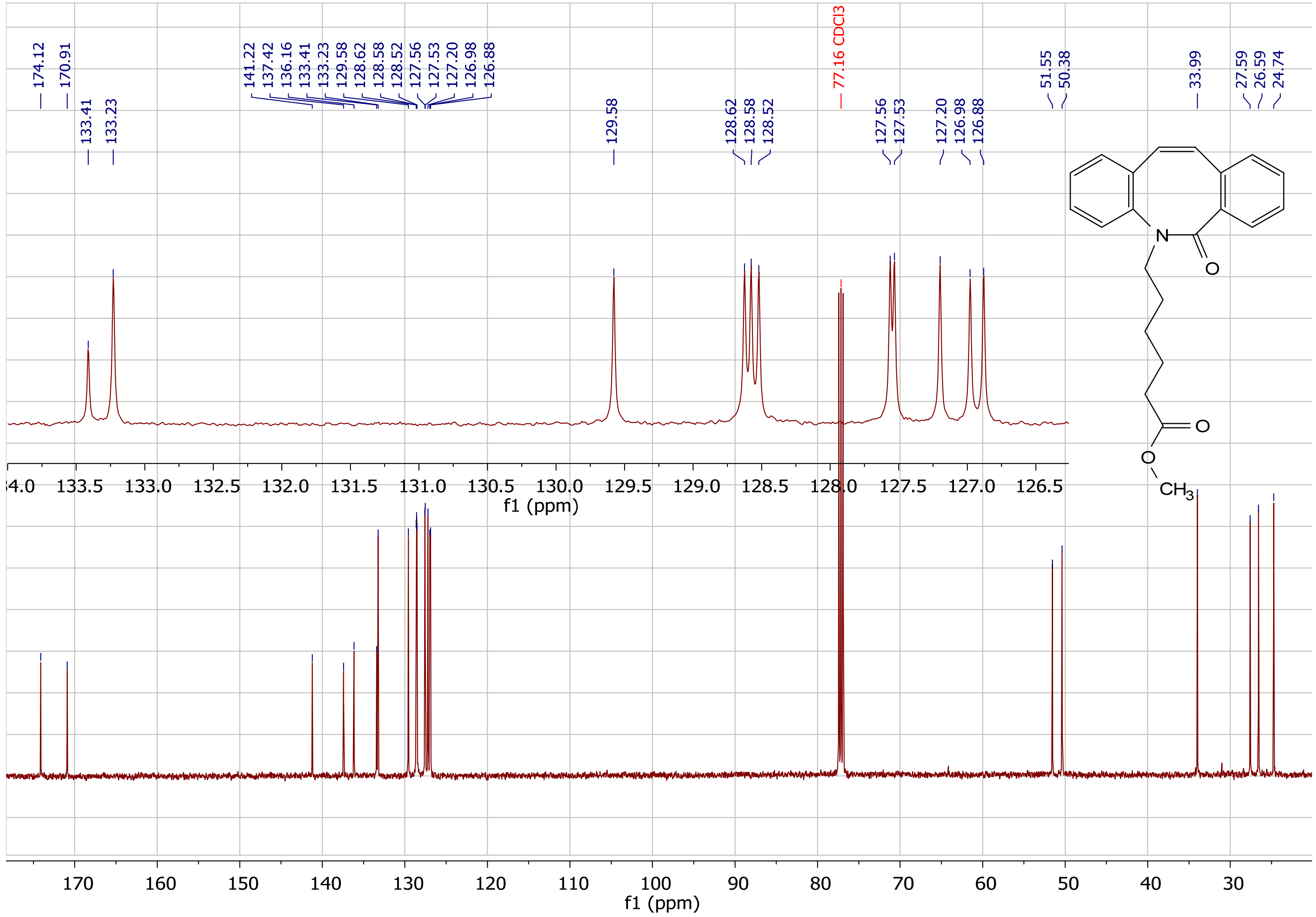

Figure S49. 13C NMR spectrum of methyl 6-(6-oxodibenzo[*b*,*f*]azocin-5(6*H*)-yl)hexanoate (**10k**).

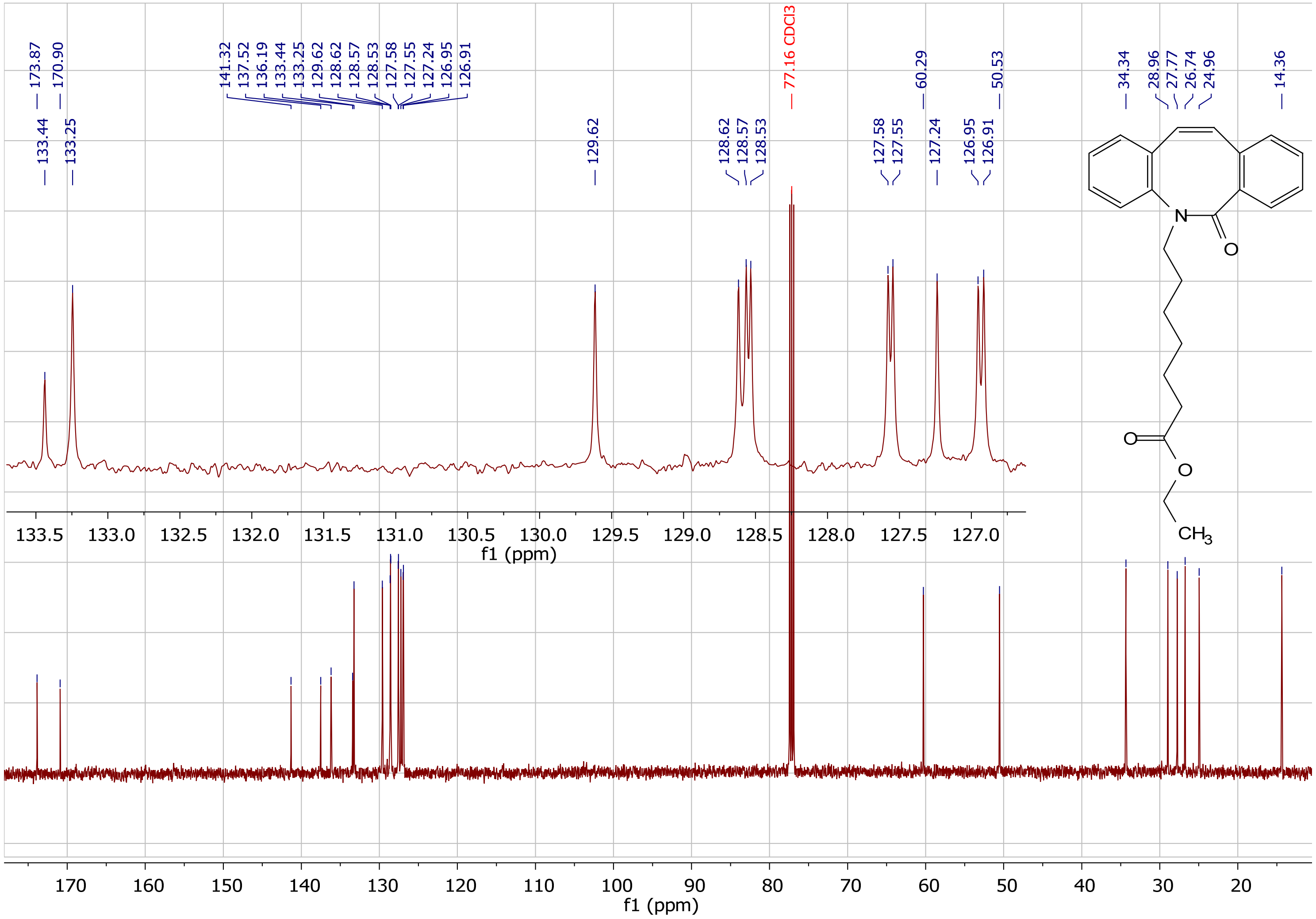

Figure S50. 13C NMR spectrum of ethyl 7-(6-oxodibenzo[*b*,*f*]azocin-5(6*H*)-yl)heptanoate (**10l**).

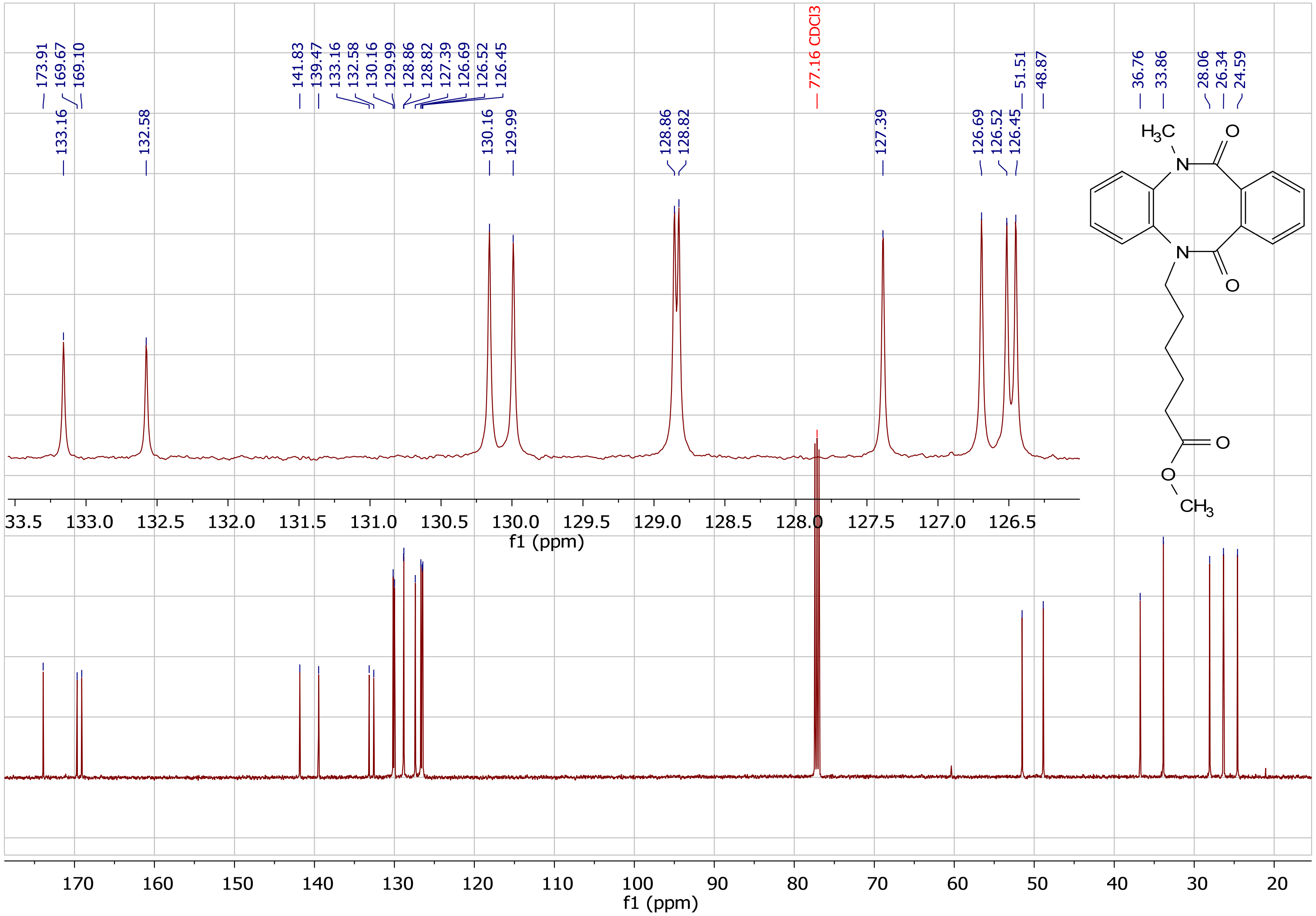

Figure S51. 13C NMR spectrum of methyl 6-(12-methyl-6,11-dioxo-11,12-dihydrodibenzo[*b*,*f*][1,4]diazocin-5(6*H*)-yl)hexanoate (**10m**).

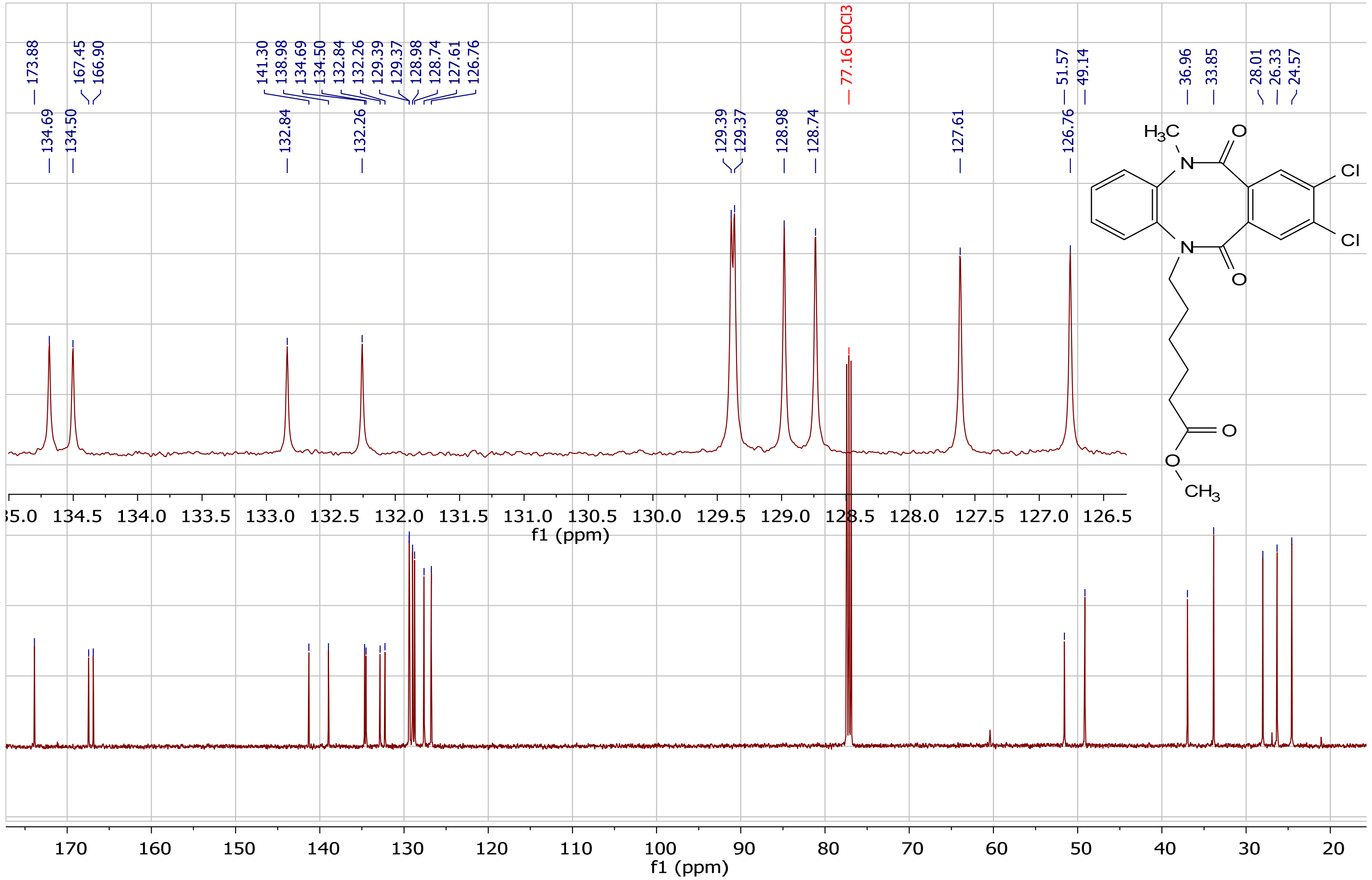

Figure S52. 13C NMR spectrum of methyl 6-(8,9-dichloro-12-methyl-6,11-dioxo-11,12-dihydrodibenzo[*b*,*f*][1,4]diazocin-5(6*H*)-yl) hexanoate (**10n**).

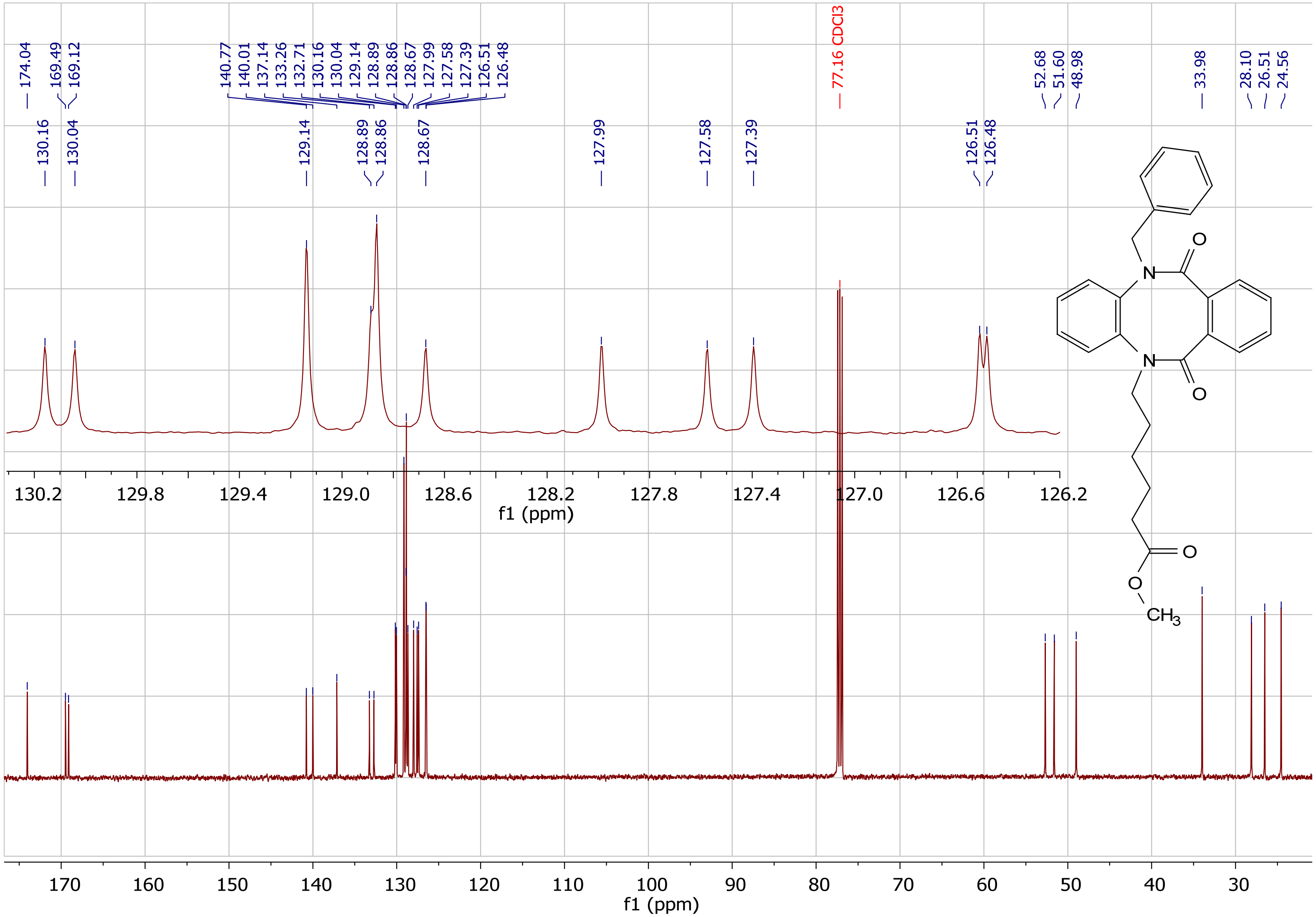

Figure S53. 13C NMR spectrum of methyl 6-(12-benzyl-6,11-dioxo-11,12-dihydrodibenzo[*b*,*f*][1,4]diazocin-5(6*H*)-yl)hexanoate (**10o**).

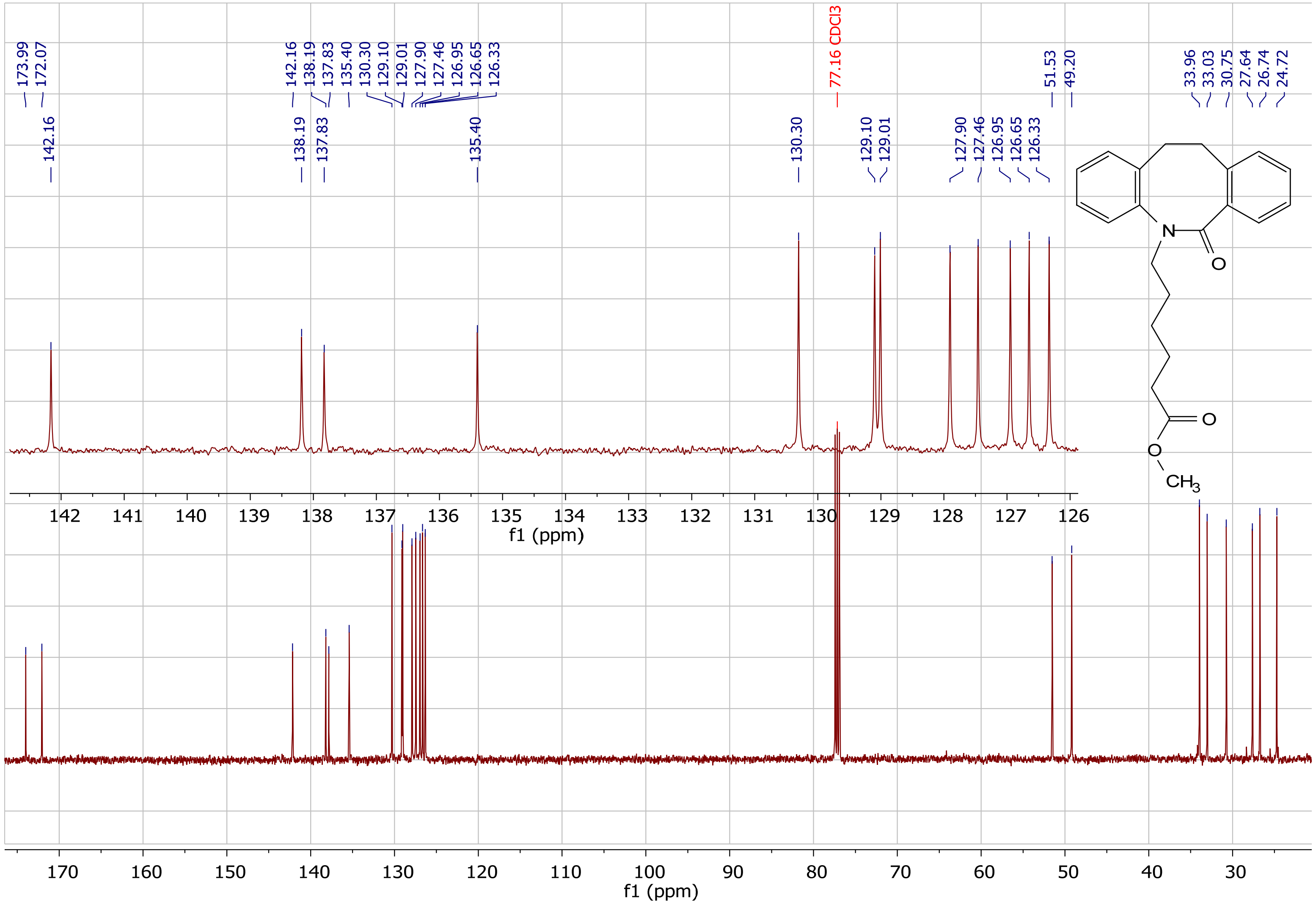

Figure S54. 13C NMR spectrum of methyl 6-(6-oxo-11,12-dihydrodibenzo[*b*,*f*]azocin-5(6*H*)-yl)hexanoate (**10p**).

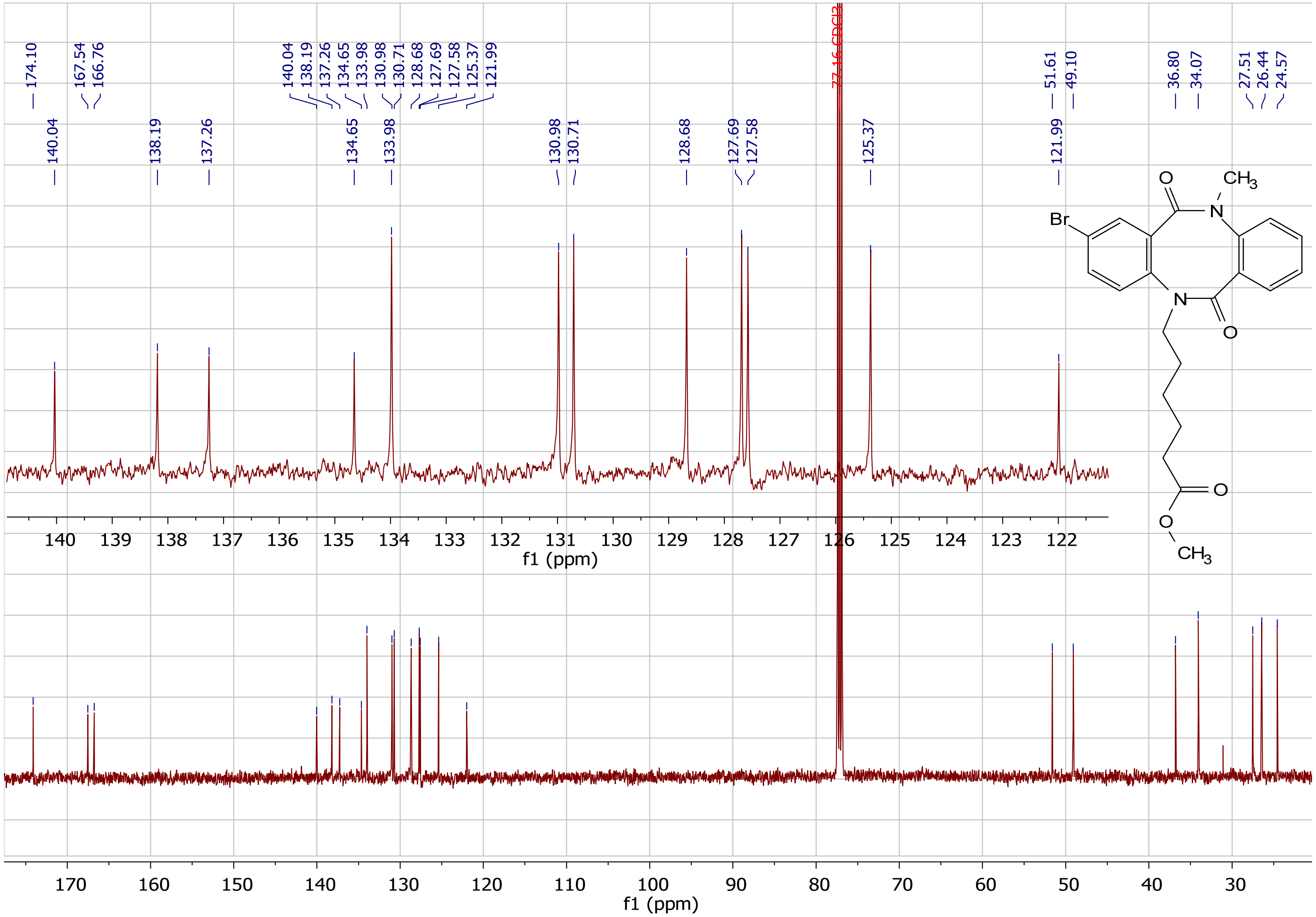

Figure S55. 13C NMR spectrum of methyl 6-(2-bromo-11-methyl-6,12-dioxo-11,12-dihydrodibenzo[*b*,*f*][1,5]diazocin-5(6*H*)-yl)hexanoate (**10r**).

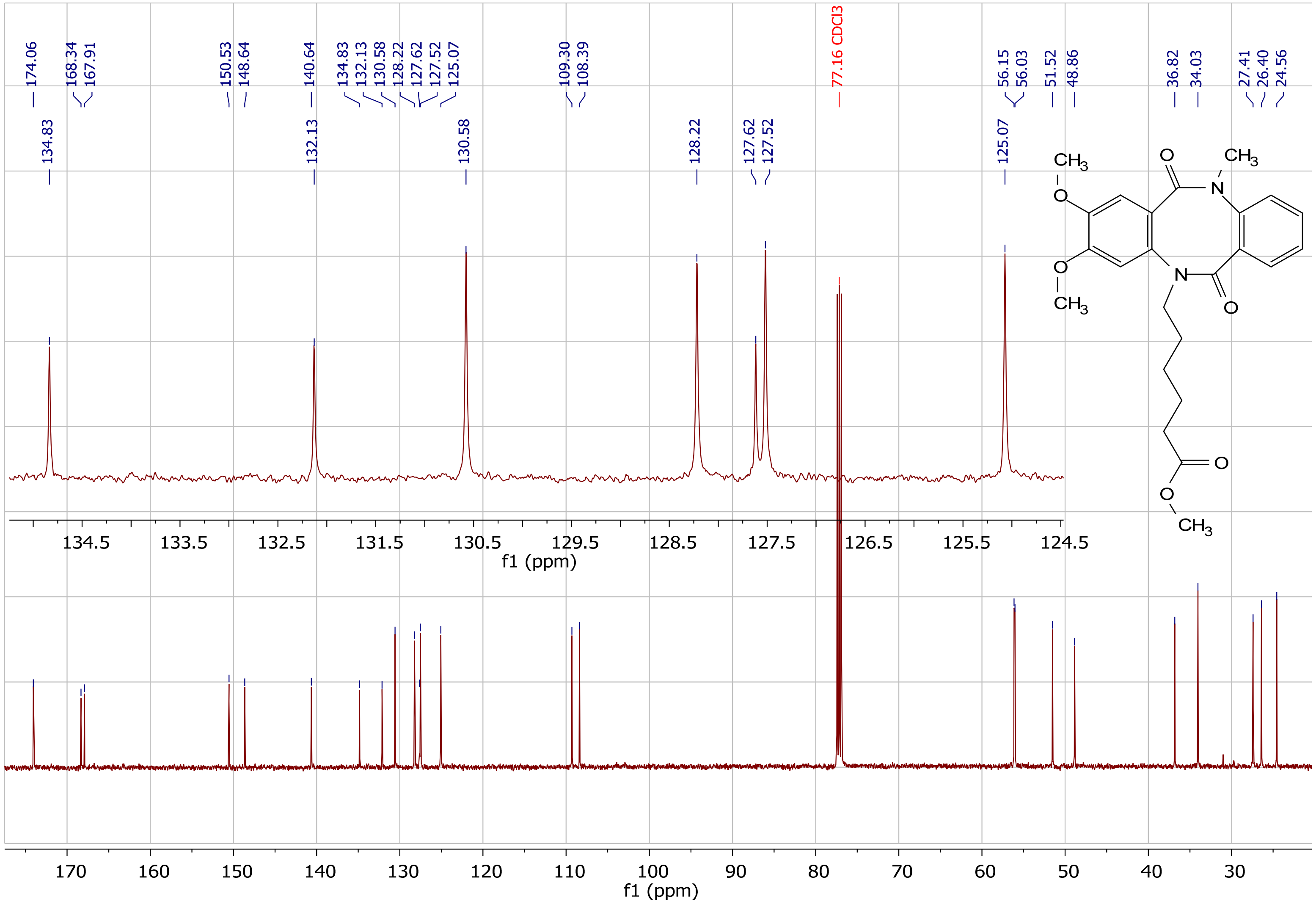

Figure S56. 13C NMR spectrum of methyl 6-(2,3-dimethoxy-11-methyl-6,12-dioxo-11,12-dihydrodibenzo[*b*,*f*][1,5]diazocin-5(6*H*)-yl)hexanoate (**10s**).

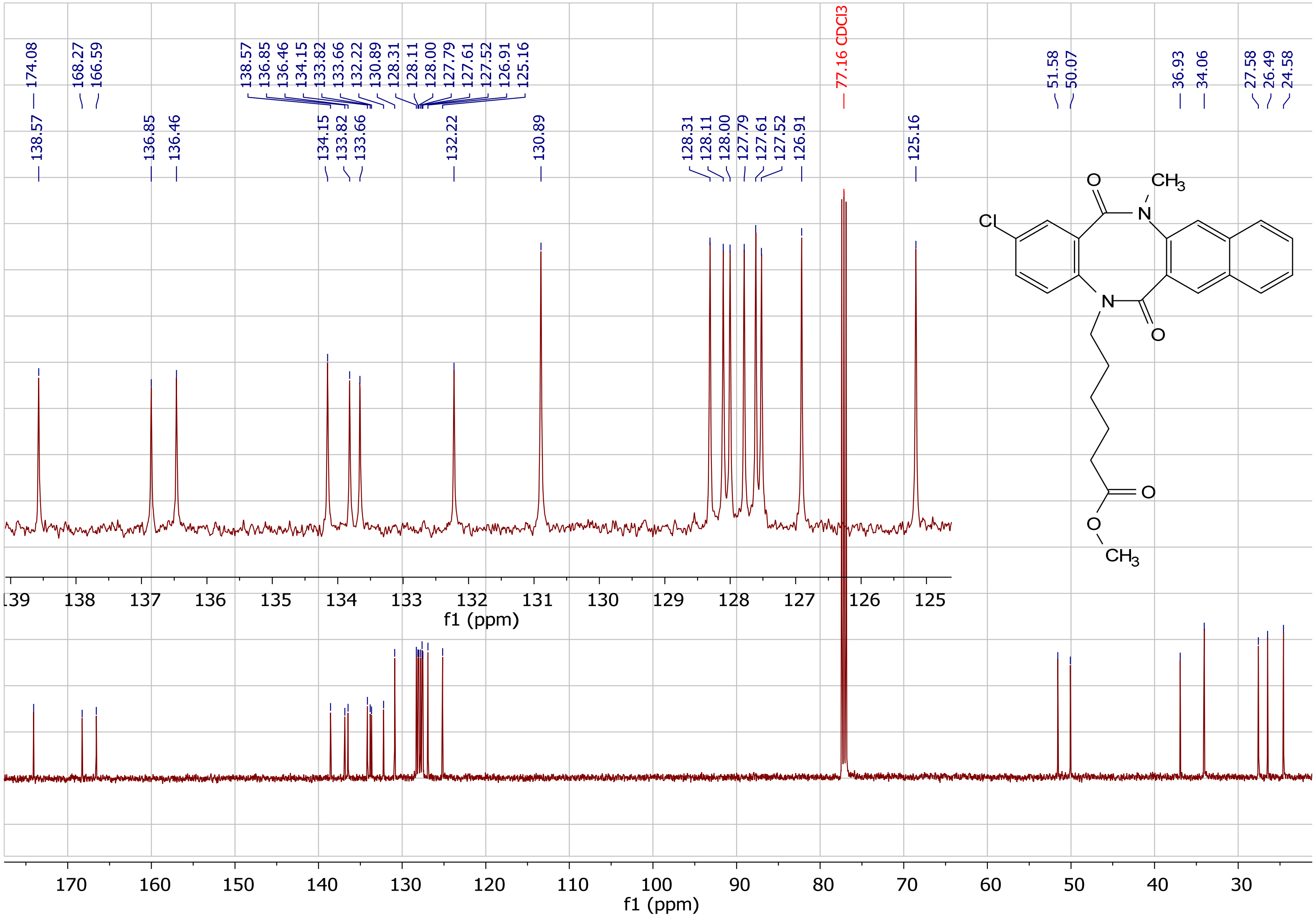

Figure S57. 13C NMR spectrum of methyl 6-(5-methyl-6,14-dioxo-5,14-dihydrobenzonaphtho[2,3-*f*][1,5]diazocin-13(6*H*)-yl)hexanoate (**10t**).

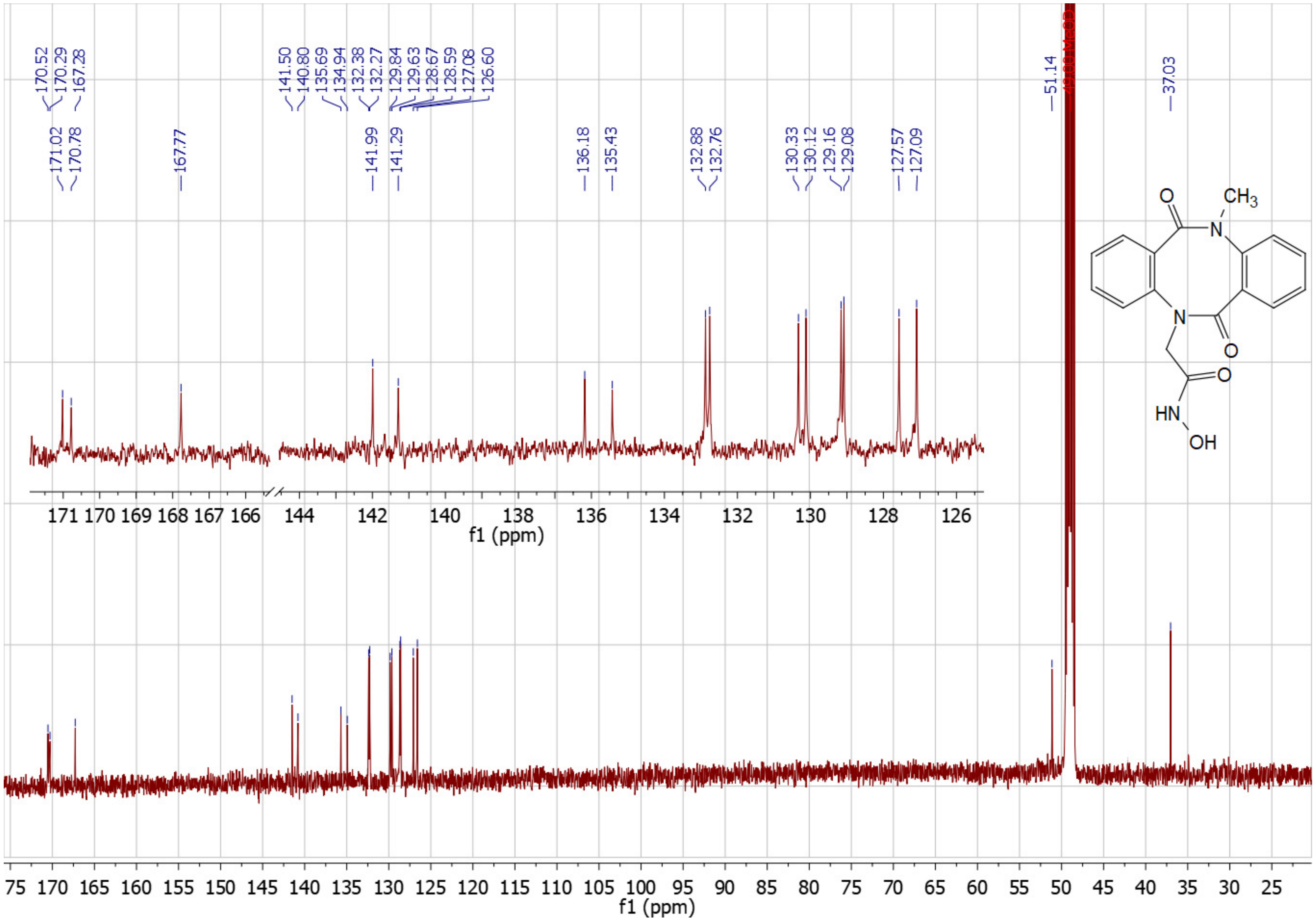

Figure S58. 13C NMR spectrum of *N*-hydroxy-2-(11-methyl-6,12-dioxo-11,12-dihydrodibenzo[*b*,*f*][1,5]diazocin-5(6*H*)-yl)acetamide (**7a**).

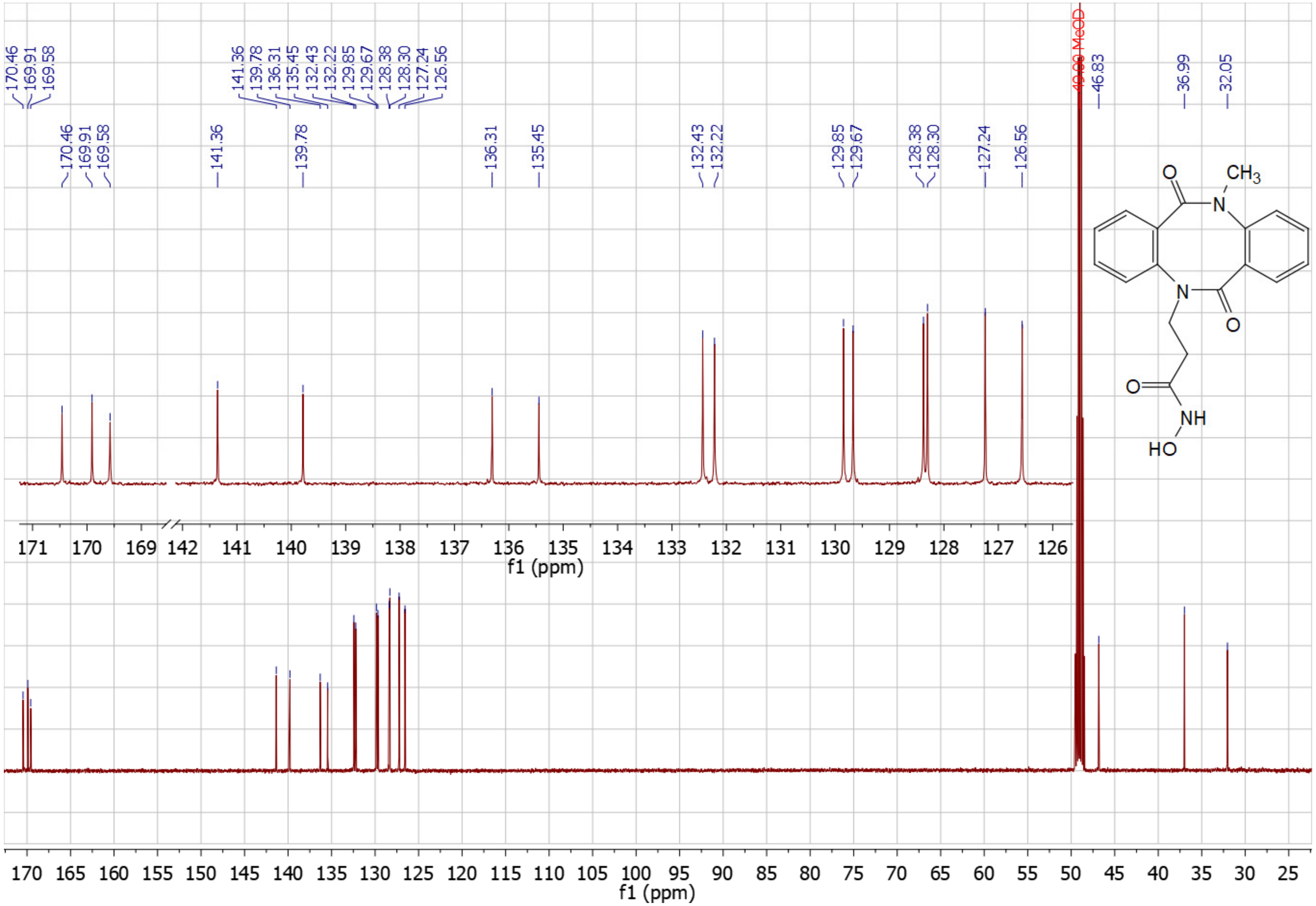

Figure S59. 13C NMR spectrum of *N*-hydroxy-3-(11-methyl-6,12-dioxo-11,12-dihydrodibenzo[*b*,*f*][1,5]diazocin-5(6*H*)-yl)propanamide (**7b**).

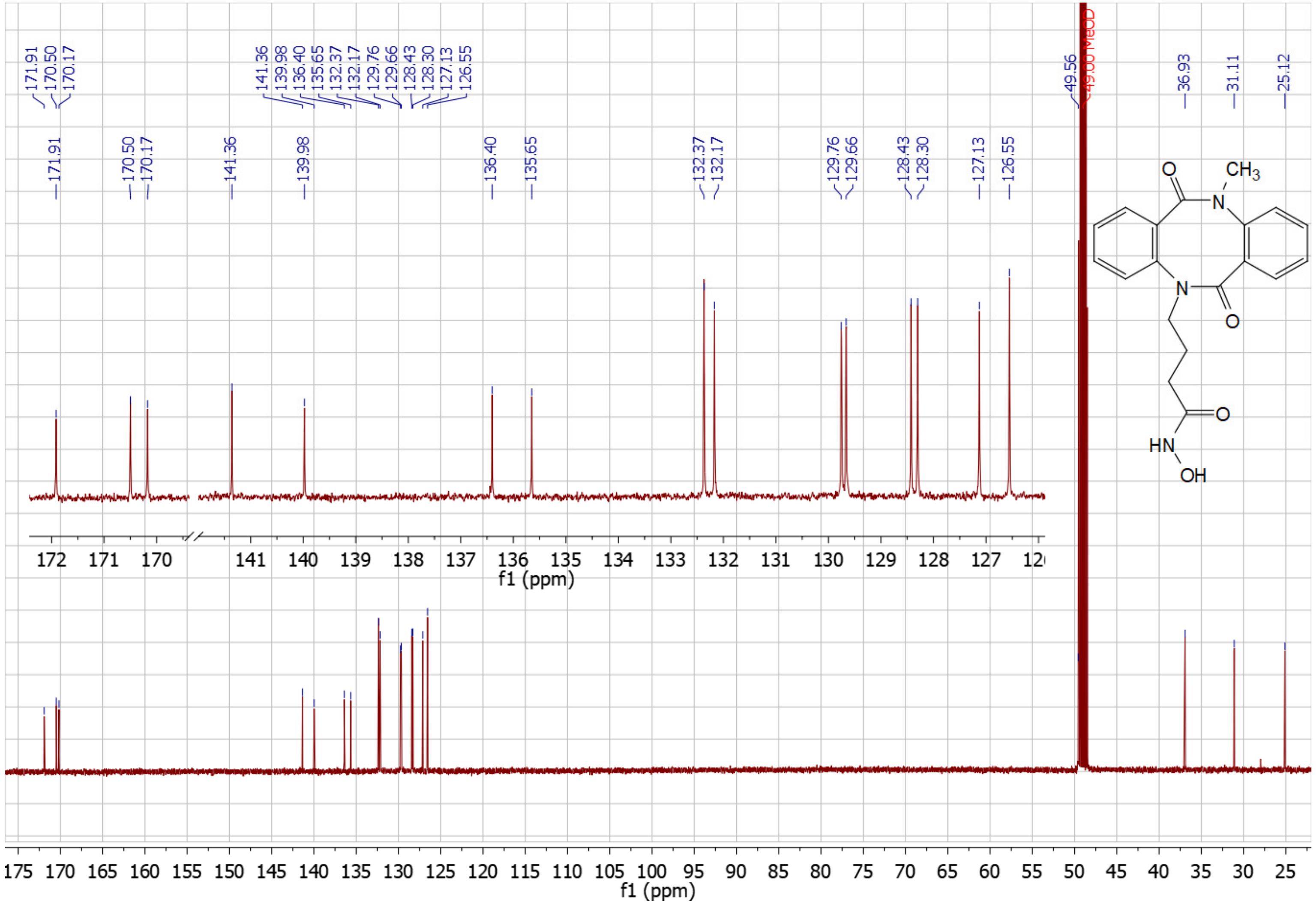

Figure S60. 13C NMR spectrum of *N*-hydroxy-4-(11-methyl-6,12-dioxo-11,12-dihydrodibenzo[*b*,*f*][1,5]diazocin-5(6*H*)-yl)butanamide (**7c**).

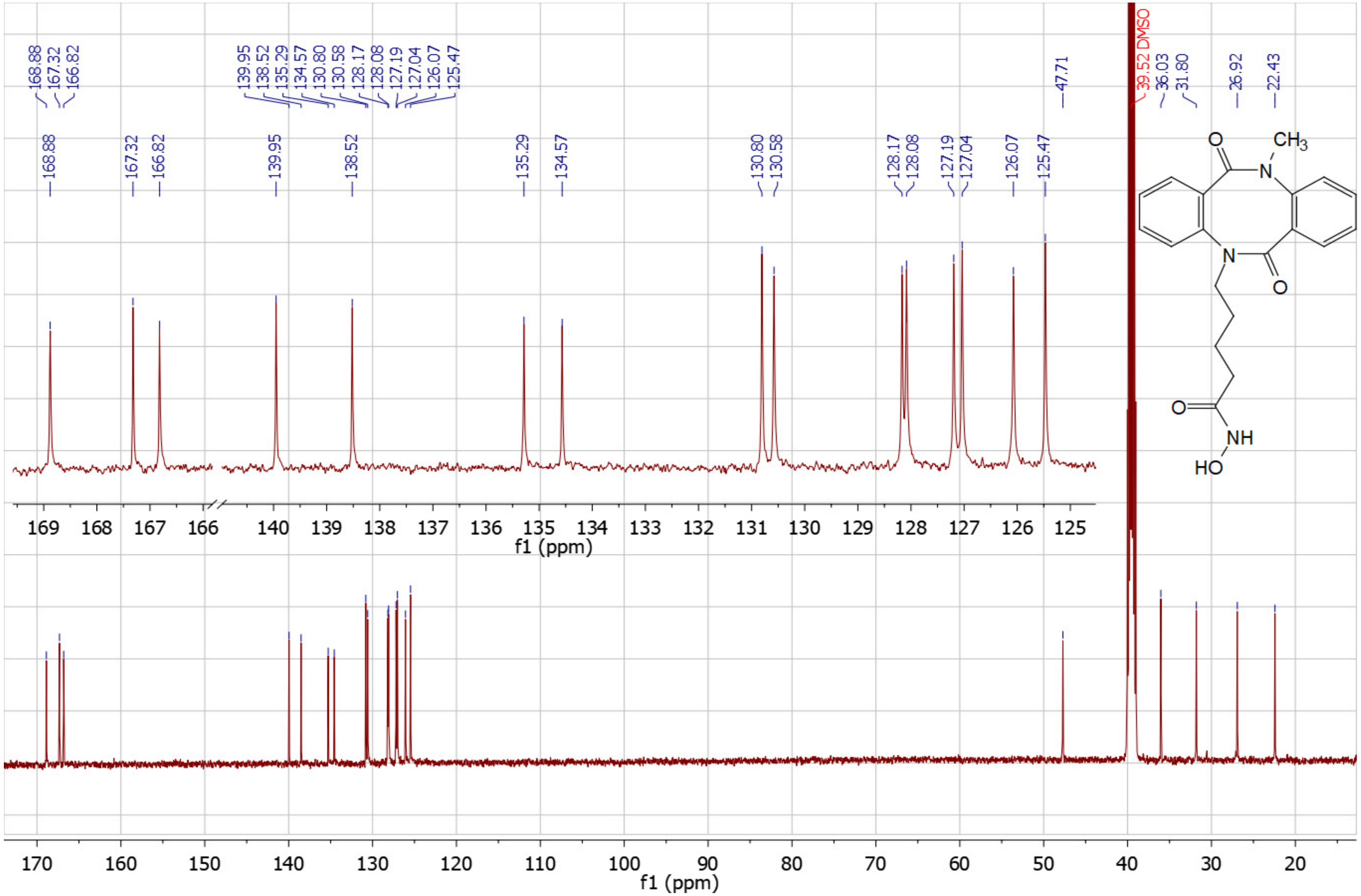

Figure S61. 13C NMR spectrum of *N*-hydroxy-5-(11-methyl-6,12-dioxo-11,12-dihydrodibenzo[*b*,*f*][1,5]diazocin-5(6*H*)-yl)pentanamide (**7d**).

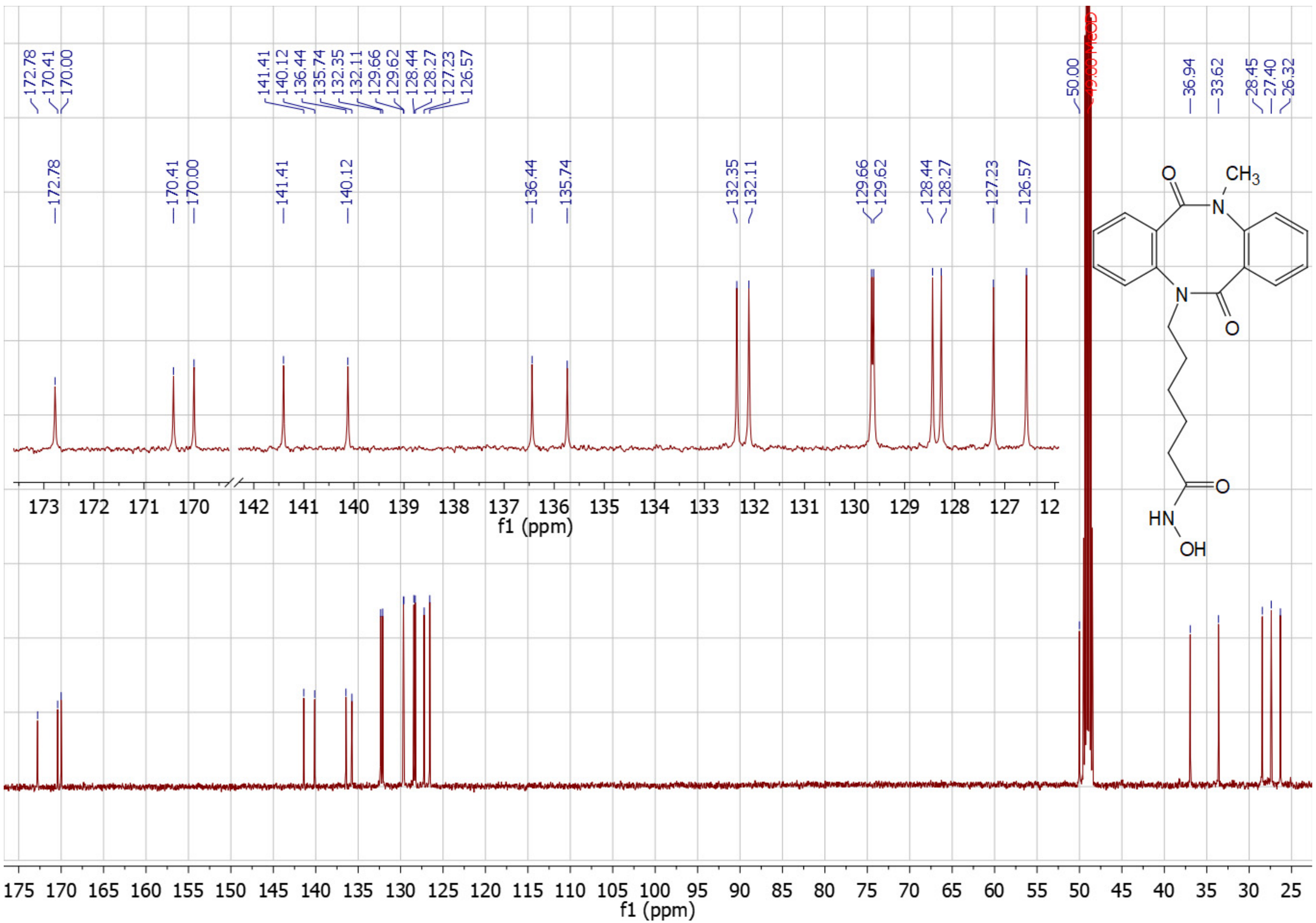

Figure S62. 13C NMR spectrum of *N*-hydroxy-6-(11-methyl-6,12-dioxo-11,12-dihydrodibenzo[*b*,*f*][1,5]diazocin-5(6*H*)-yl)hexanamide (**7e**).

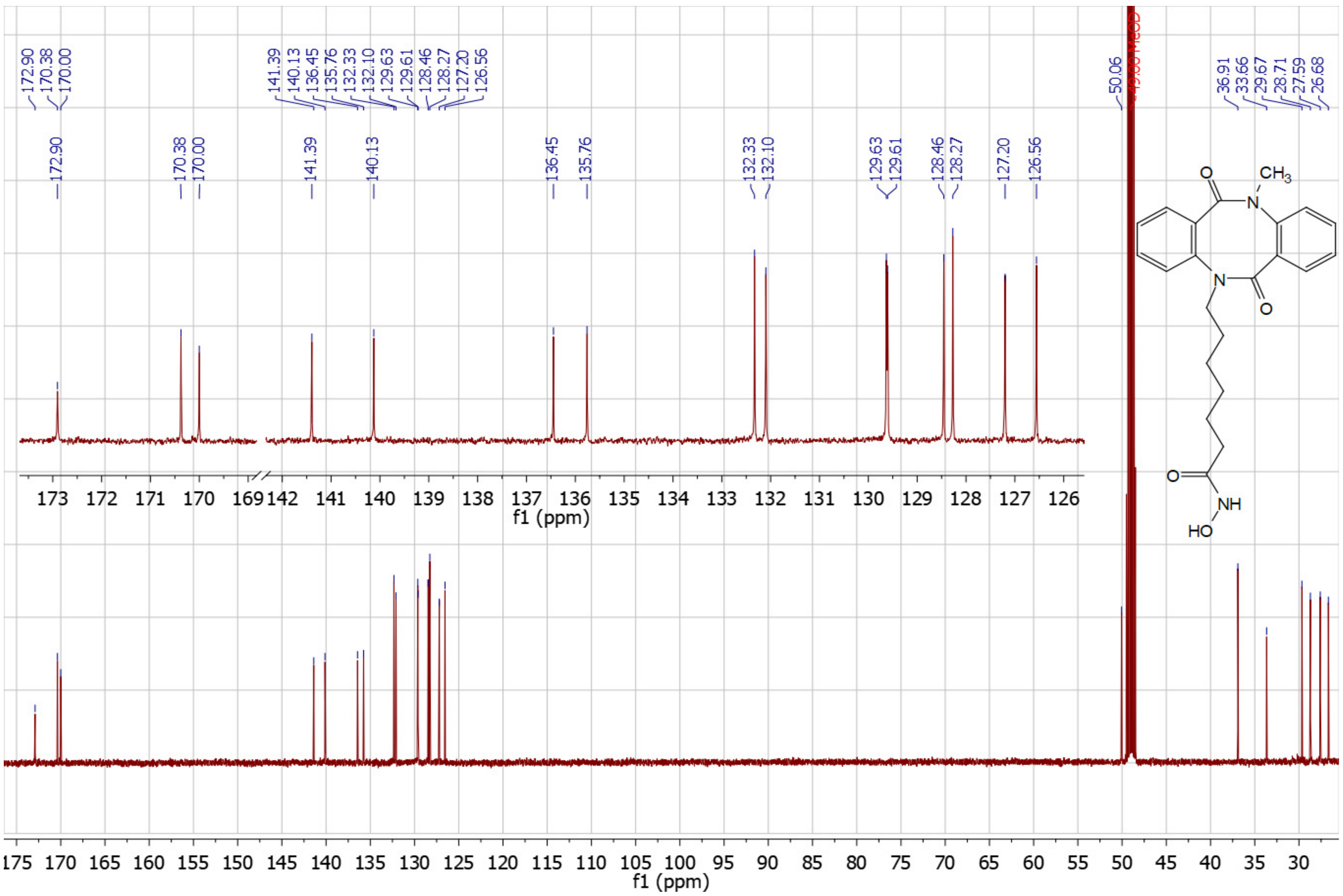

Figure S63. 13C NMR spectrum of *N*-hydroxy-7-(11-methyl-6,12-dioxo-11,12-dihydrodibenzo[*b*,*f*][1,5]diazocin-5(6*H*)-yl)heptanamide (**7f**).

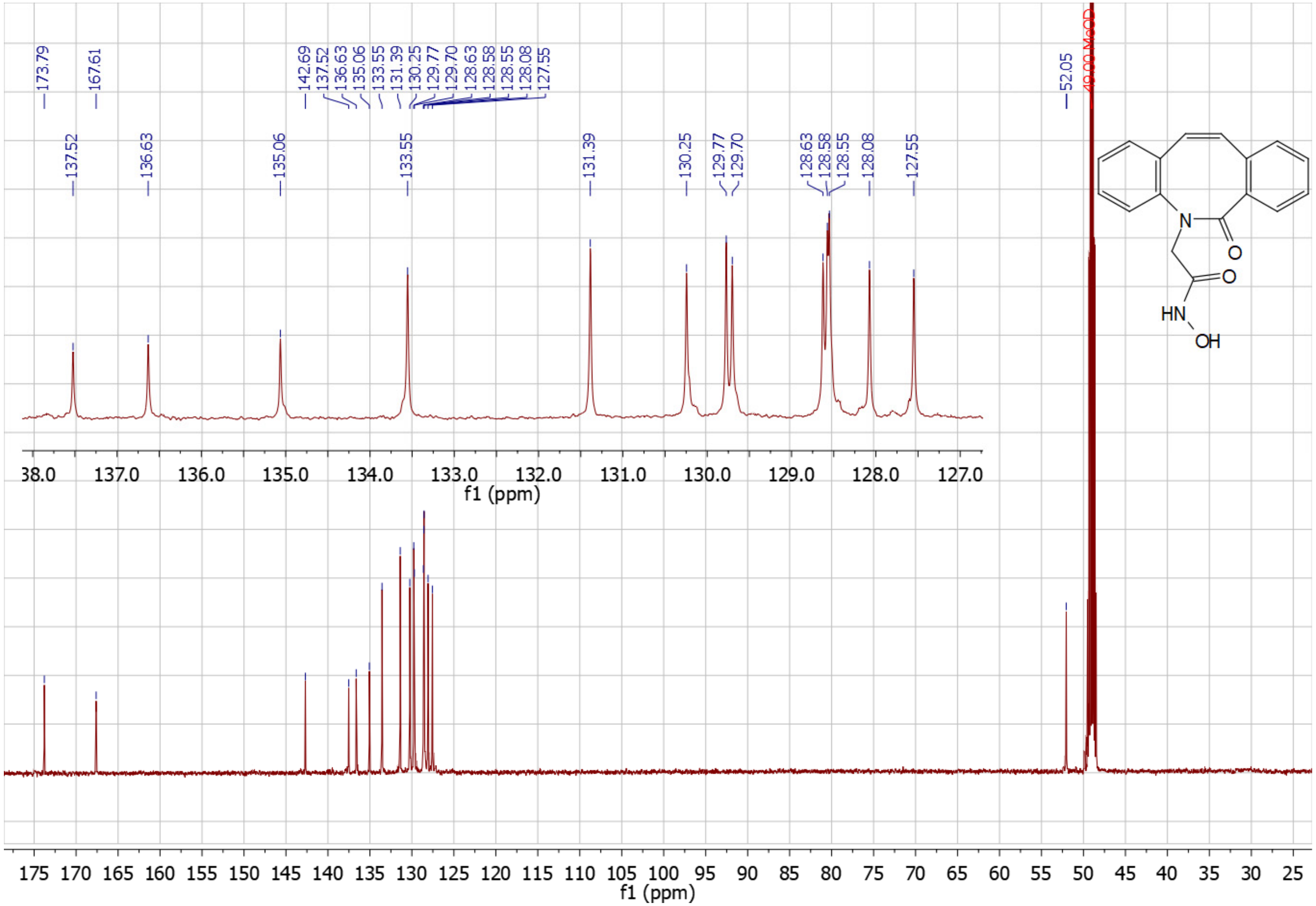

Figure S64. 13C NMR spectrum of *N*-hydroxy-2-(6-oxodibenzo[*b*,*f*]azocin-5(6*H*)-yl)acetamide (**7g**).

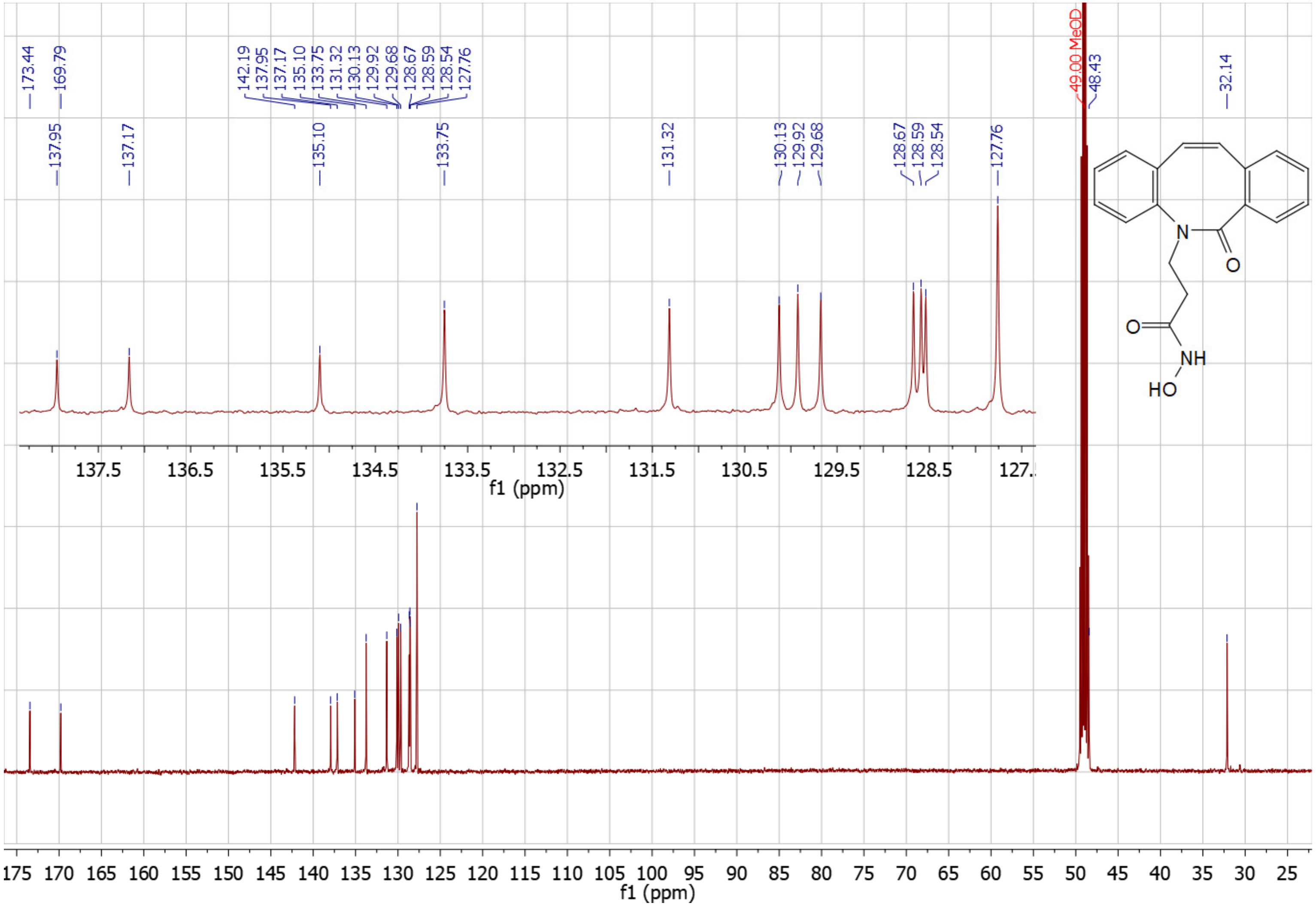

Figure S65. 13C NMR spectrum of *N*-hydroxy-3-(6-oxodibenzo[*b*,*f*]azocin-5(6*H*)-yl)propanamide (**7h**).

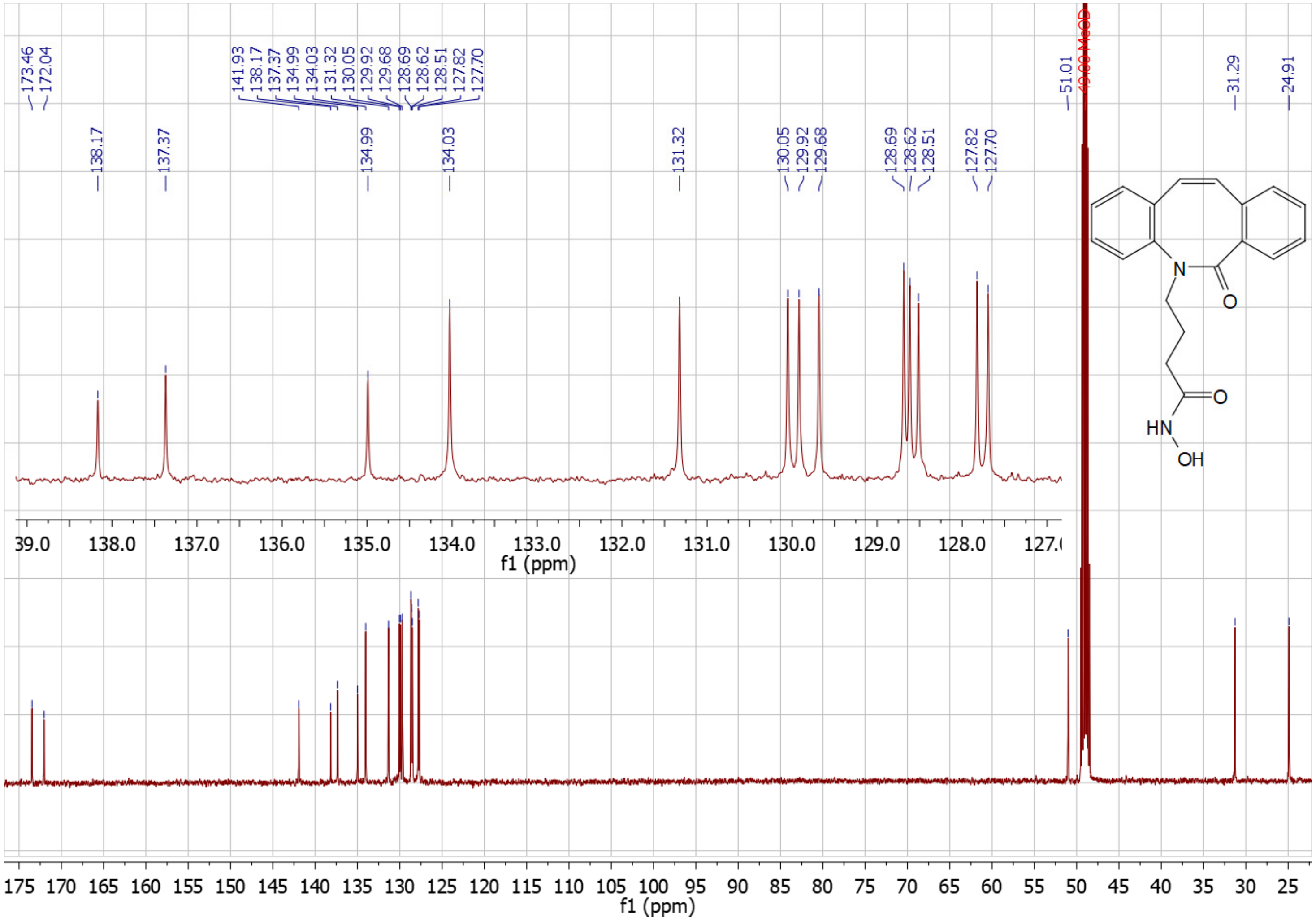

Figure S66. 13C NMR spectrum of *N*-hydroxy-4-(6-oxodibenzo[b,f]azocin-5(6*H*)-yl)butanamide (**7i**).

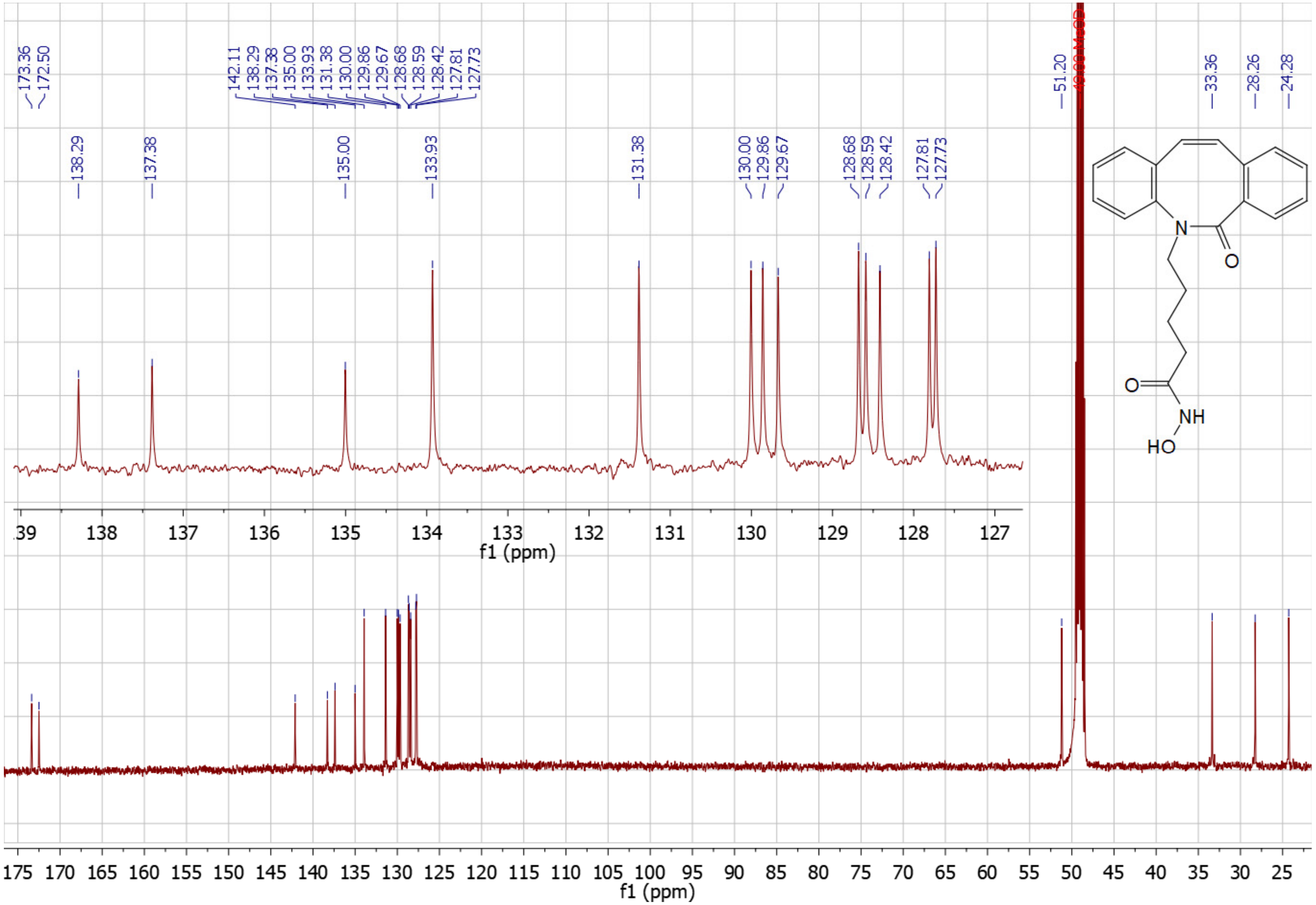

Figure S67. 13C NMR spectrum of *N*-hydroxy-5-(6-oxodibenzo[*b,f*]azocin-5(6*H*)-yl)pentanamide (**7j**).

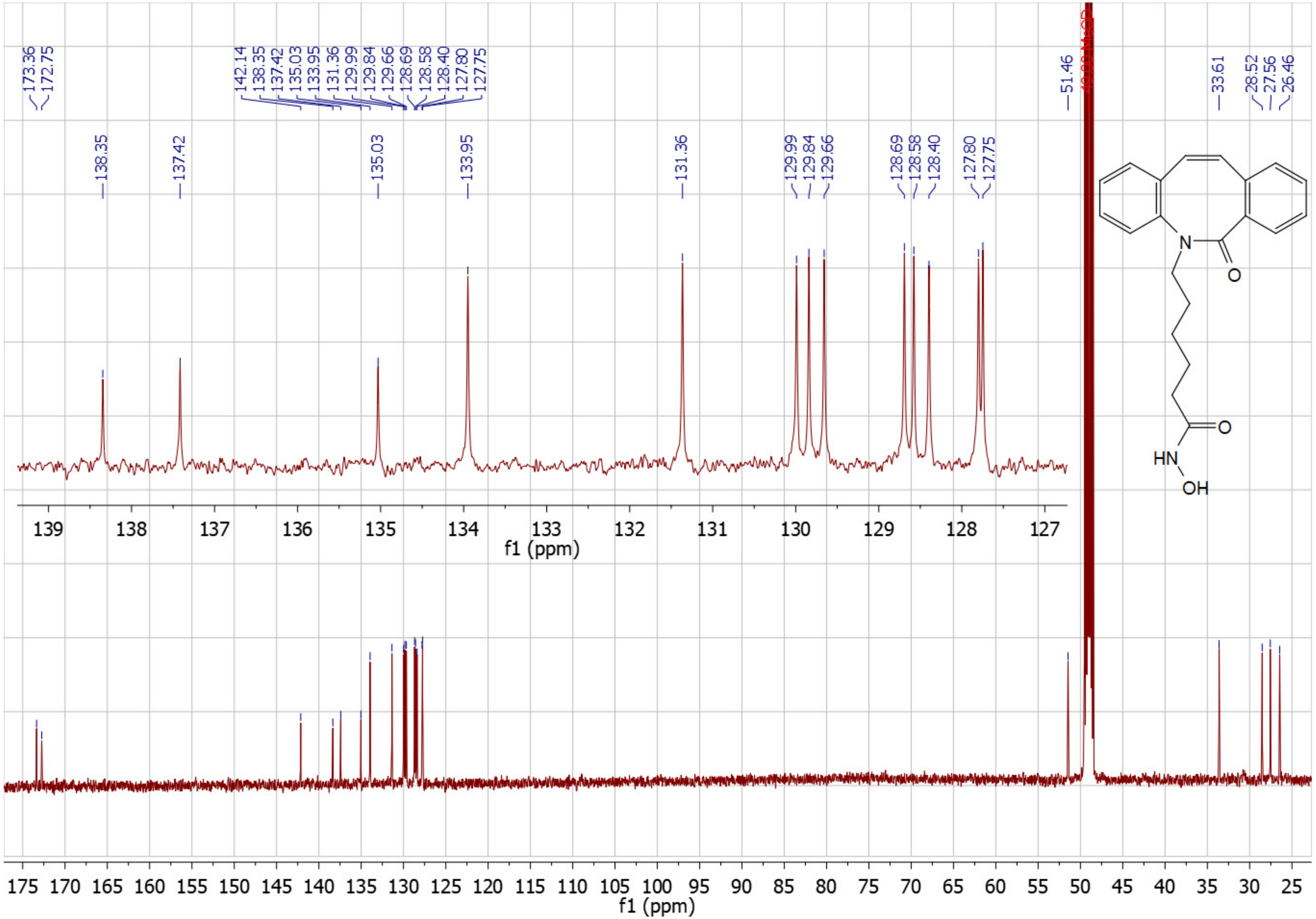

Figure S68. 13C NMR spectrum of *N*-hydroxy-6-(6-oxodibenzo[*b*,*f*]azocin-5(6*H*)-yl)hexanamide (**7k**).

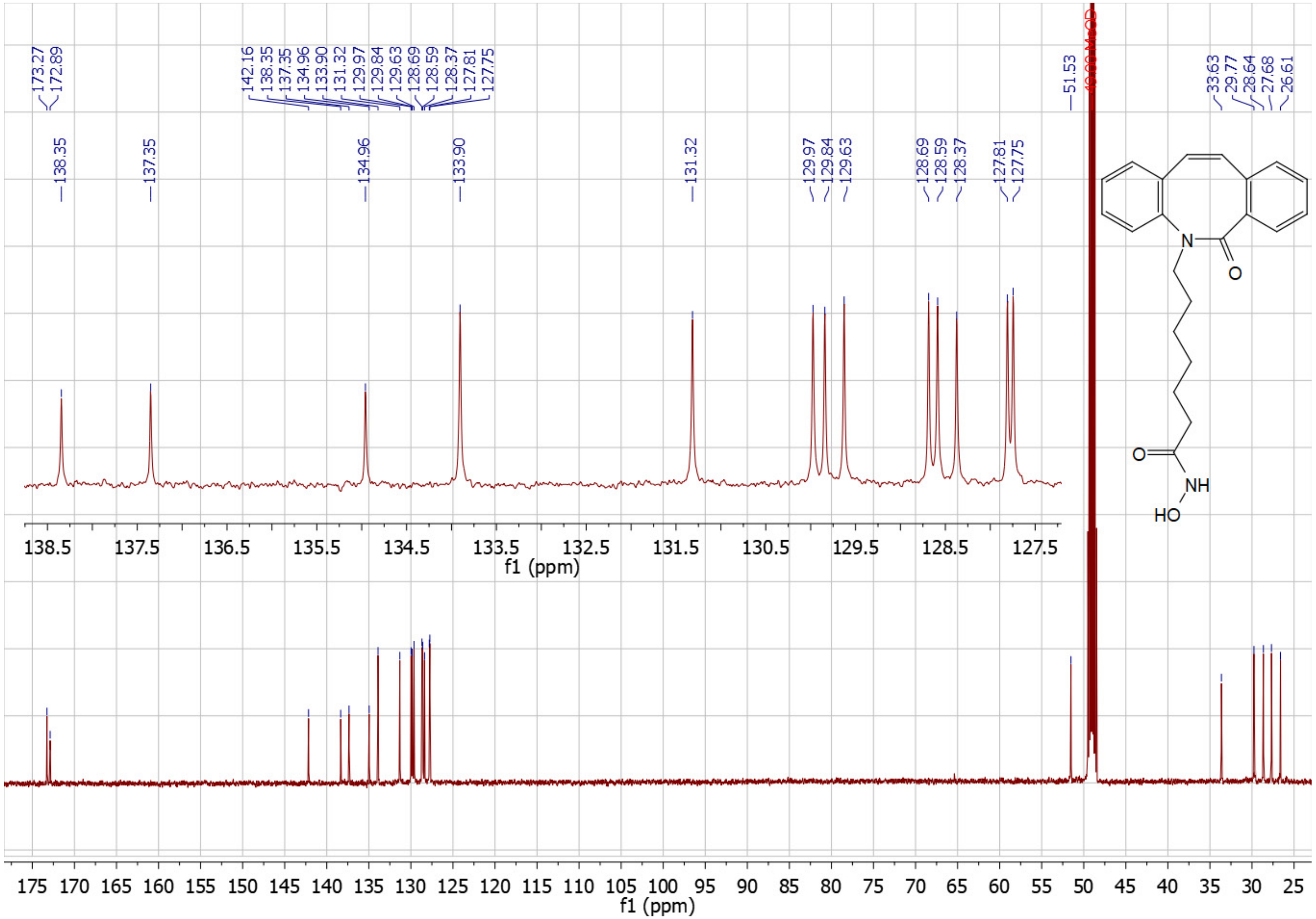

Figure S69. 13C NMR spectrum of *N*-hydroxy-7-(6-oxodibenzo[*b*,*f*]azocin-5(6*H*)-yl)heptanamide (**7l**).

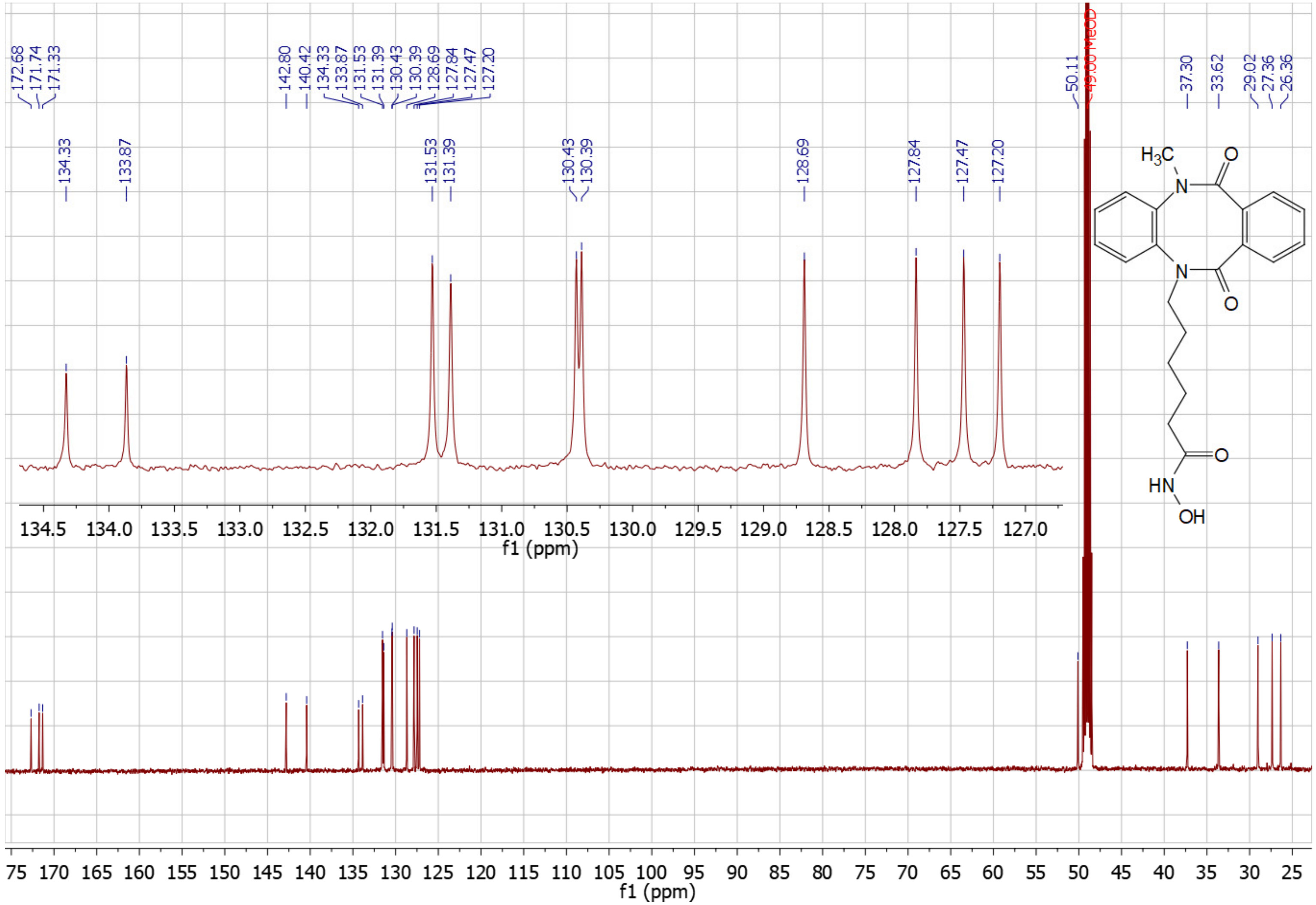

Figure S70. 13C NMR spectrum of *N*-hydroxy-6-(12-methyl-6,11-dioxo-11,12-dihydrodibenzo[*b*,*f*][1,4]diazocin-5(6*H*)-yl)hexanamide (**7m**).

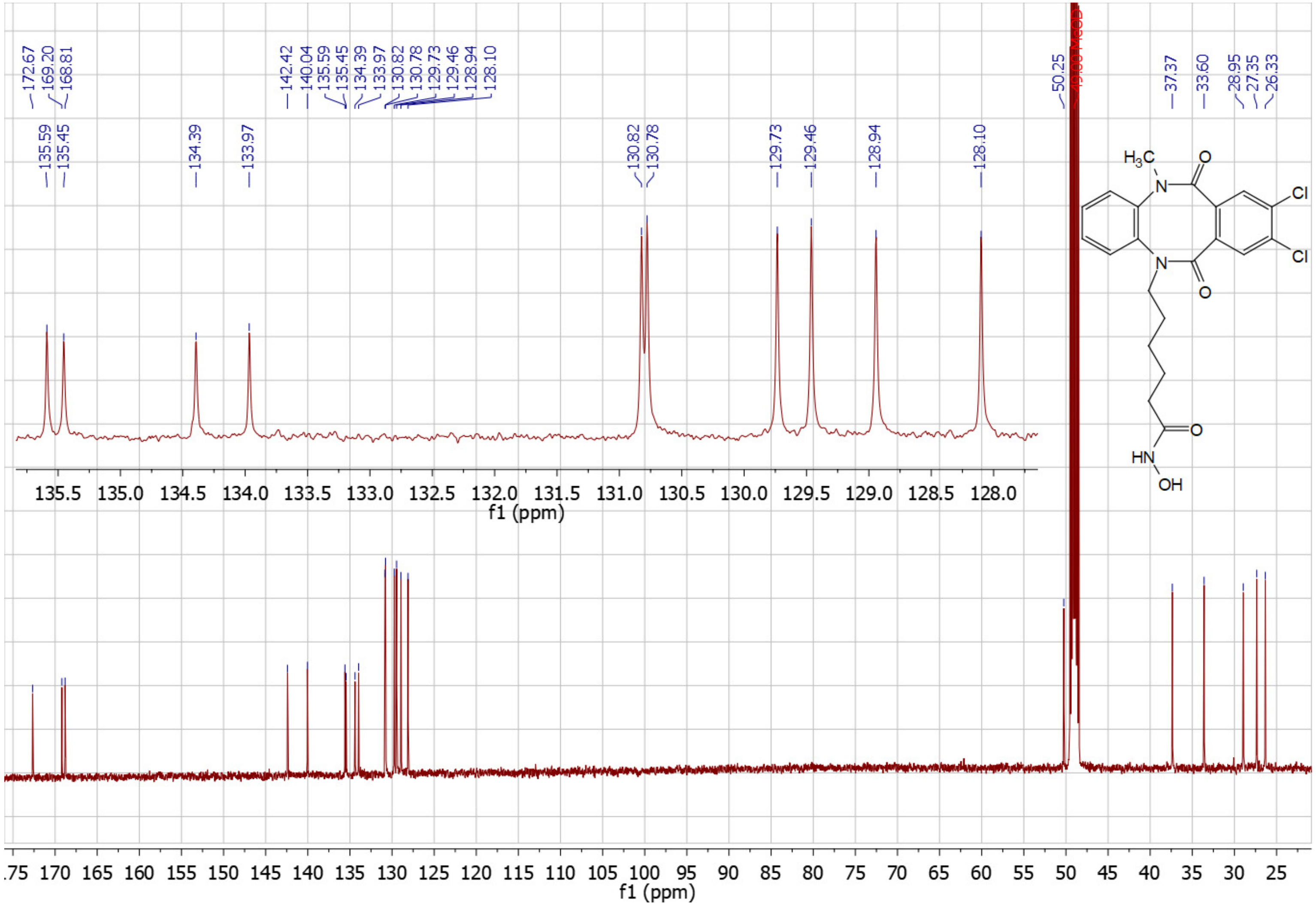

Figure S71. 13C NMR spectrum of *N*-hydroxy-6-(8,9-dichloro-12-methyl-6,11-dioxo-11,12-dihydrodibenzo[*b*,*f*][1,4]diazocin-5(6*H*)-yl)hexanamide (**7n**).

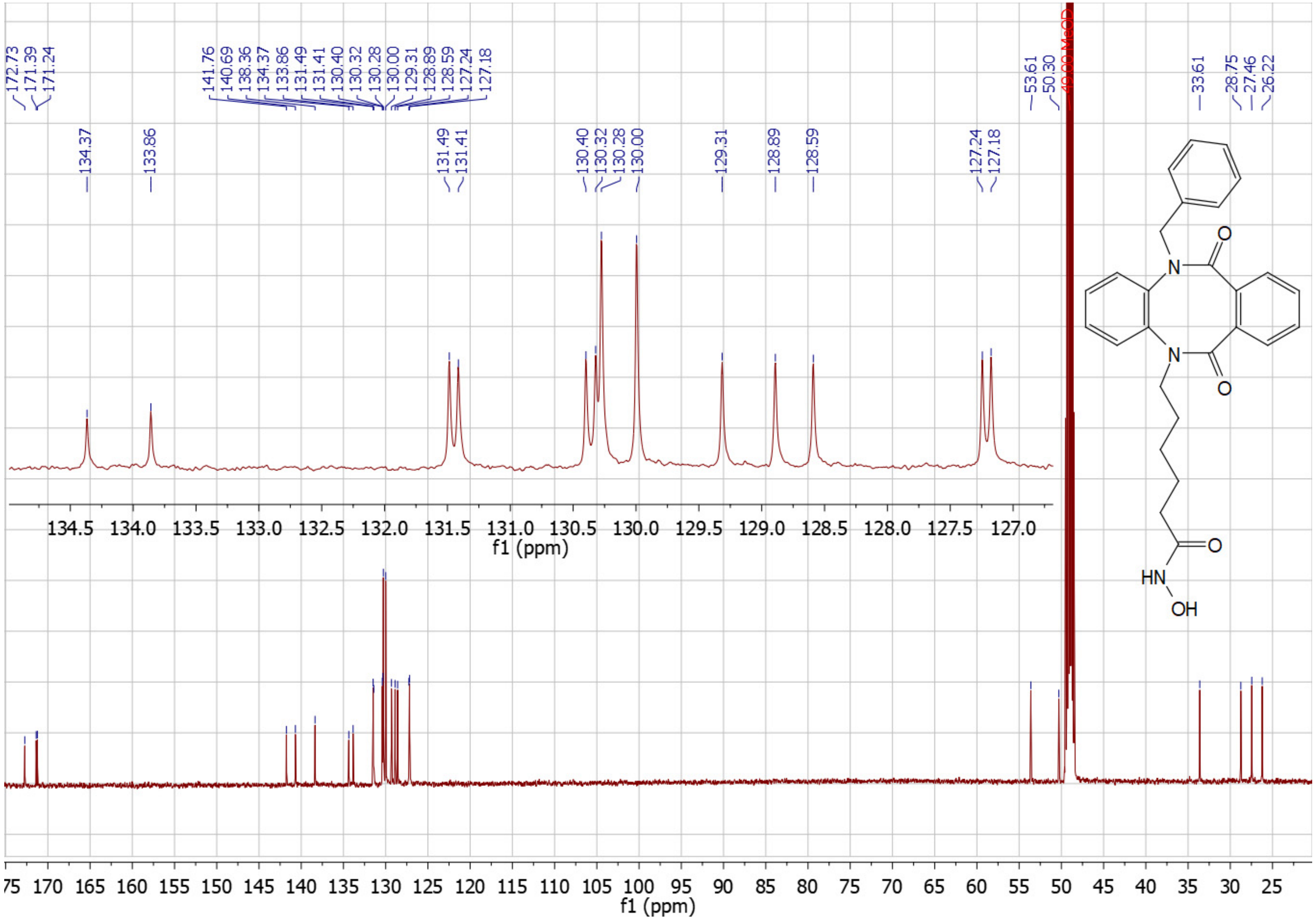

Figure S72. 13C NMR spectrum of *N*-hydroxy-6-(12-benzyl-6,11-dioxo-11,12-dihydrodibenzo[*b*,*f*][1,4]diazocin-5(6*H*)-yl)hexanamide (**7o**).

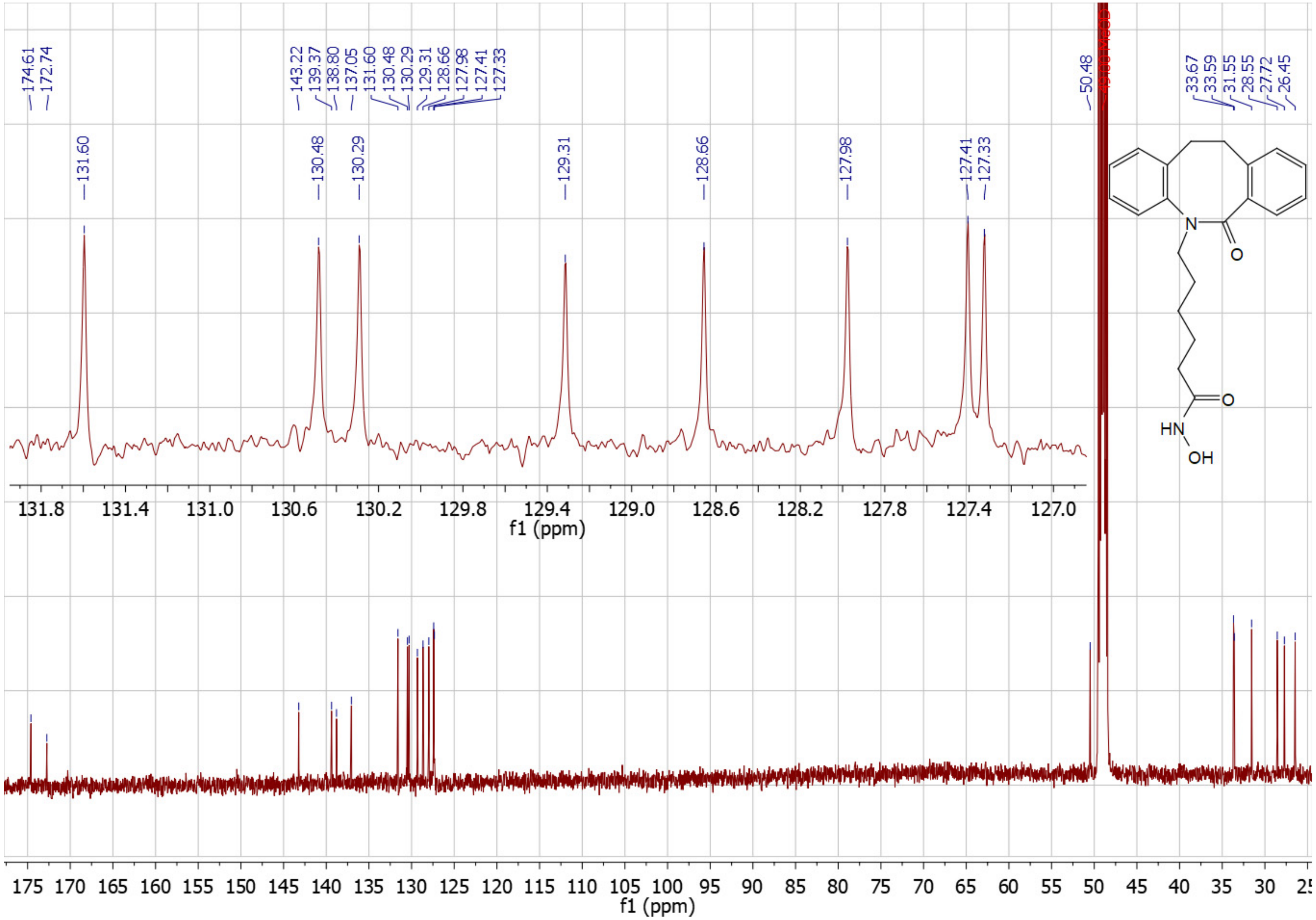

Figure S73. 13C NMR spectrum of *N*-hydroxy-6-(6-oxo-11,12-dihydrodibenzo[*b*,*f*]azocin-5(6*H*)-yl)hexanamide (**7p**).

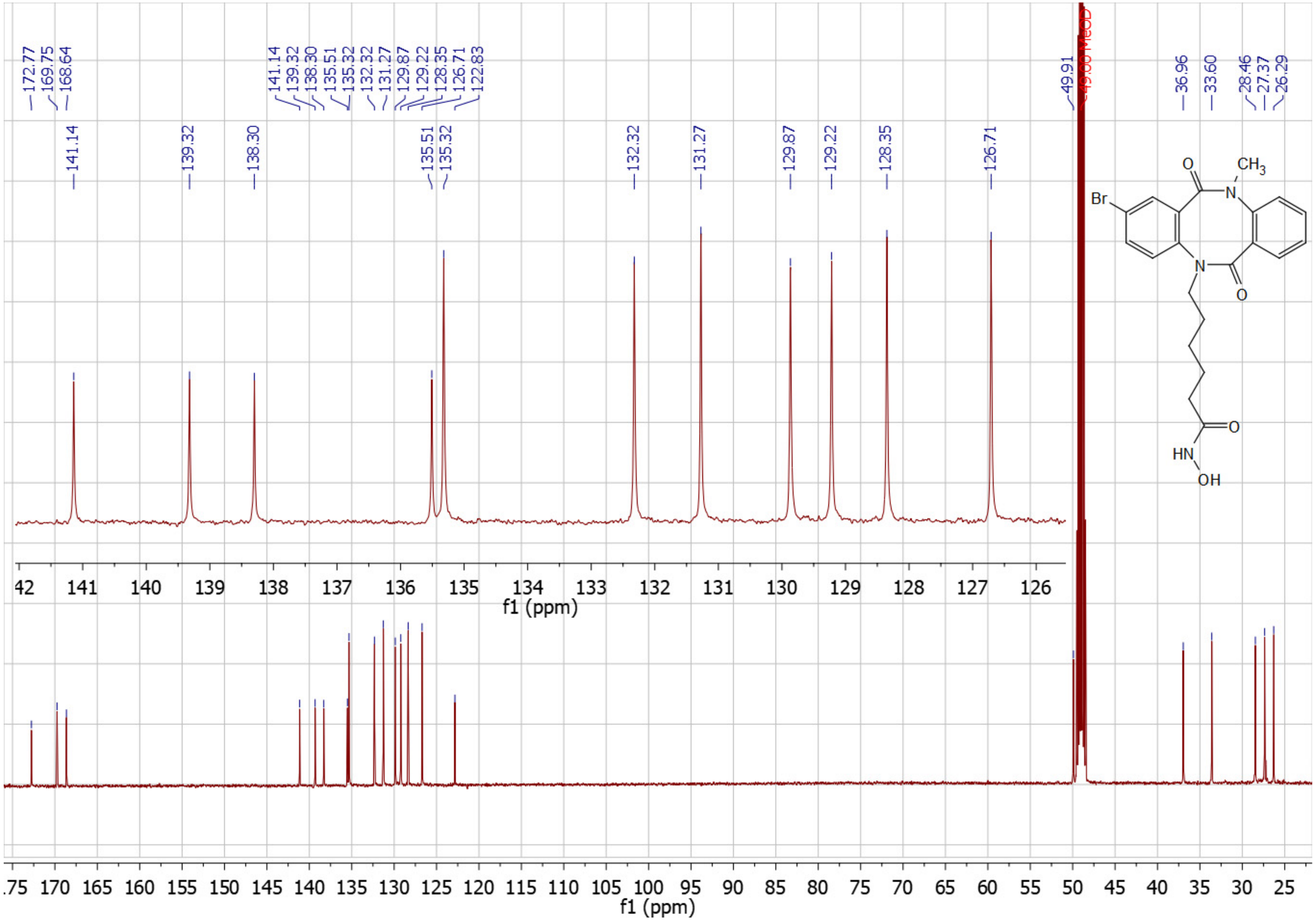

Figure S74. 13C NMR spectrum of *N*-hydroxy-6-(2-bromo-11-methyl-6,12-dioxo-11,12-dihydrodibenzo[*b*,*f*][1,5]diazocin-5(6*H*)-yl)hexanamide (**7r**).

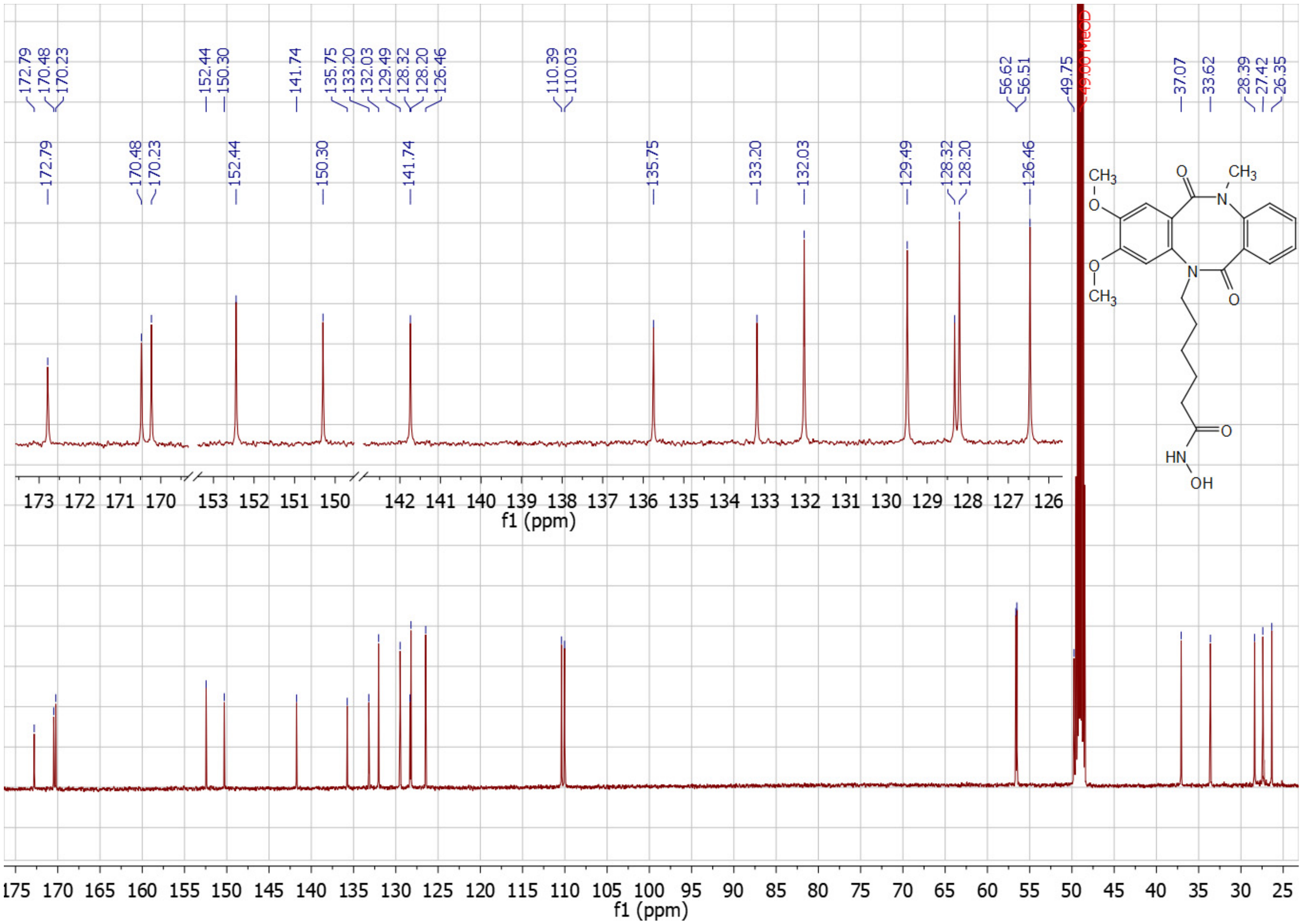

Figure S75. 13C NMR spectrum of *N*-hydroxy-6-(2,3-dimethoxy-11-methyl-6,12-dioxo-11,12-dihydrodibenzo[*b*,*f*][1,5]diazocin-5(6*H*)-yl)hexanamide (**7s**).

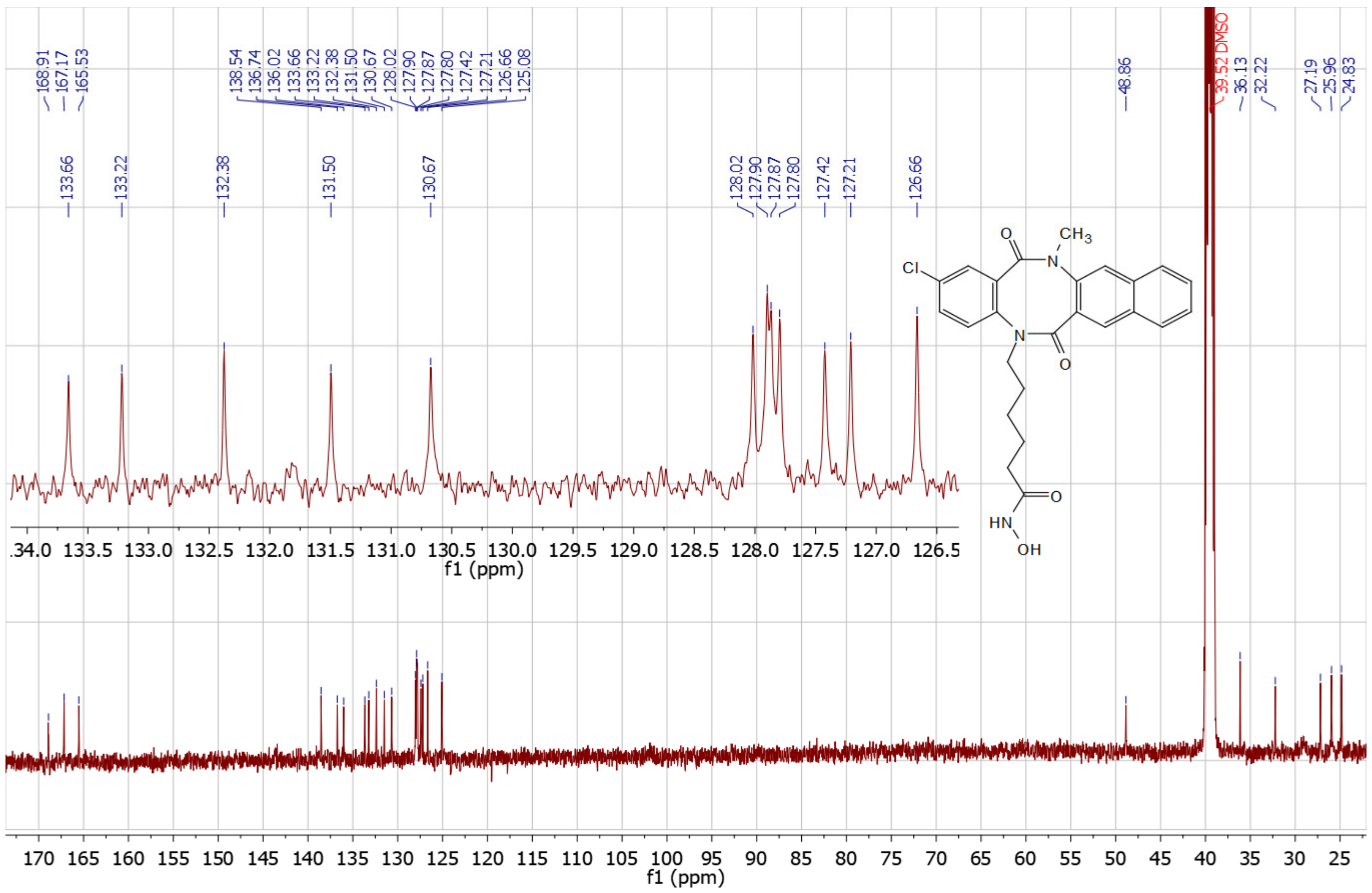

Figure S76. 13C NMR spectrum of *N*-hydroxy-6-(5-methyl-6,14-dioxo-5,14-dihydrobenzonaphtho[2,3-*f*][1,5]diazocin-13(6*H*)-yl)hexanamide (**7t**).

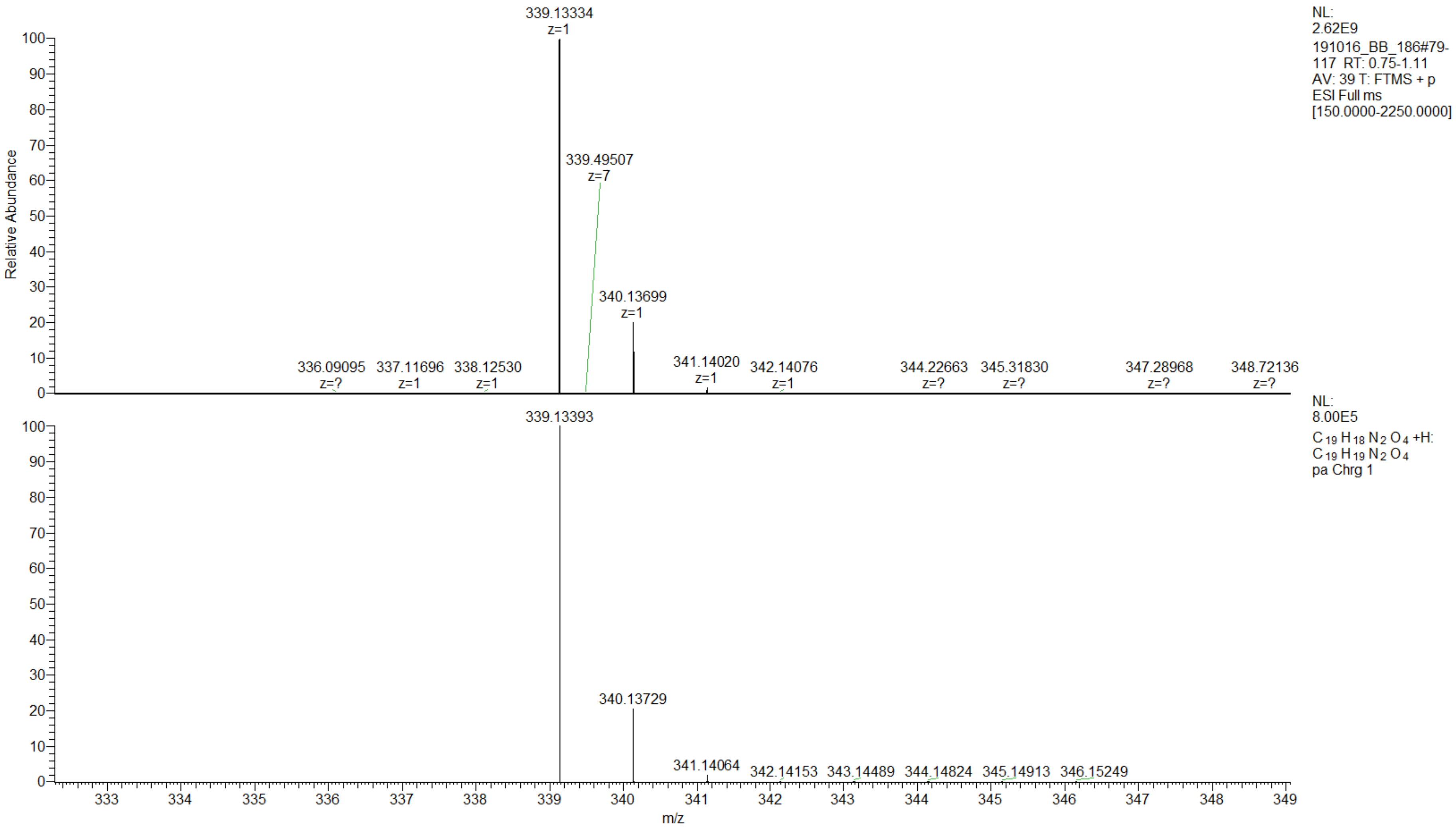

Figure S77. ESI HRMS spectrum of ethyl 2-(11-methyl-6,12-dioxo-11,12-dihydrodibenzo[*b*,*f*][1,5]diazocin-5(6*H*)-yl)acetate (**10a**).

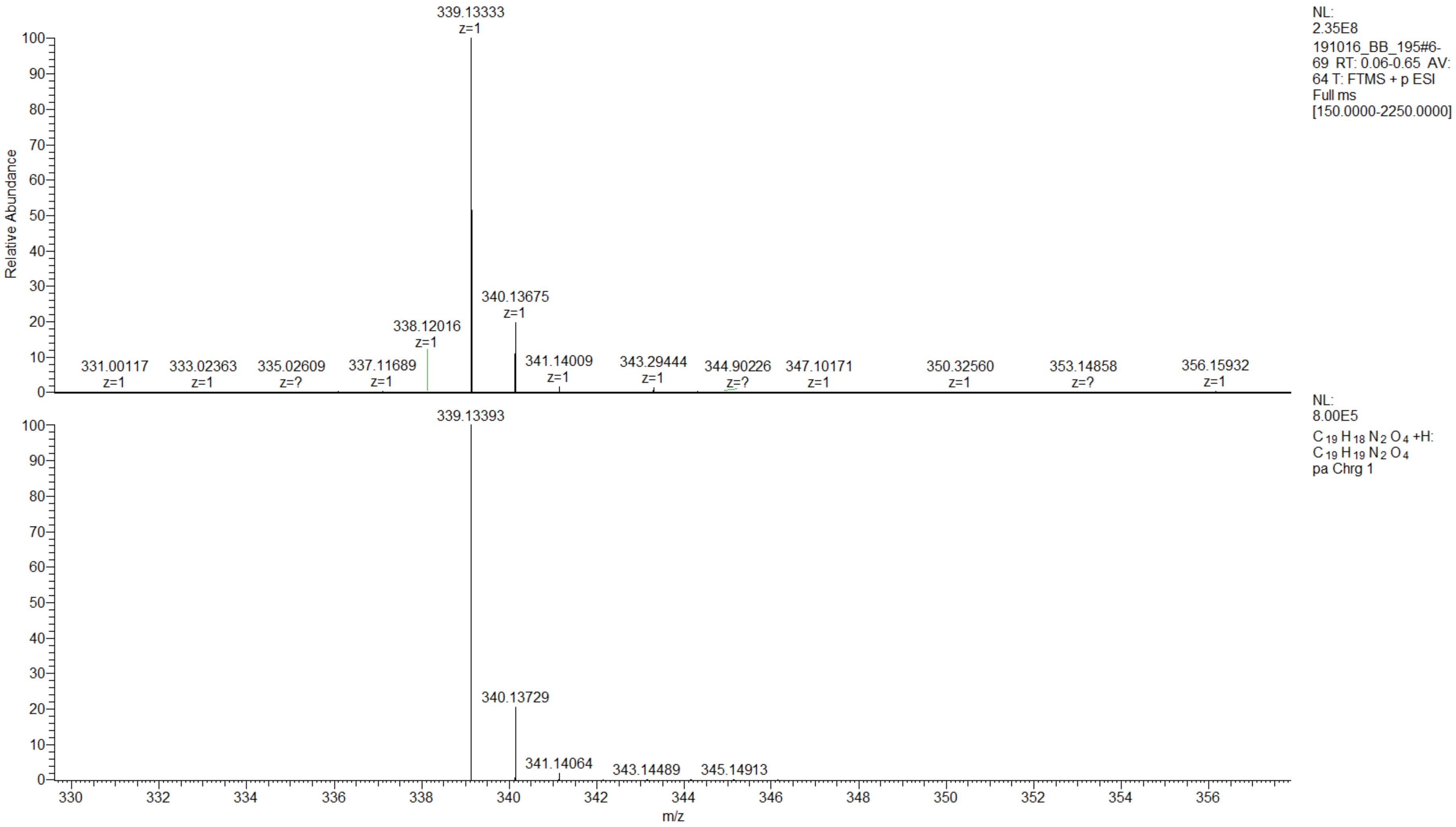

Figure S78. ESI HRMS spectrum of ethyl 3-(11-methyl-6,12-dioxo-11,12-dihydrodibenzo[*b*,*f*][1,5]diazocin-5(6*H*)-yl)propanoate (**10b**).

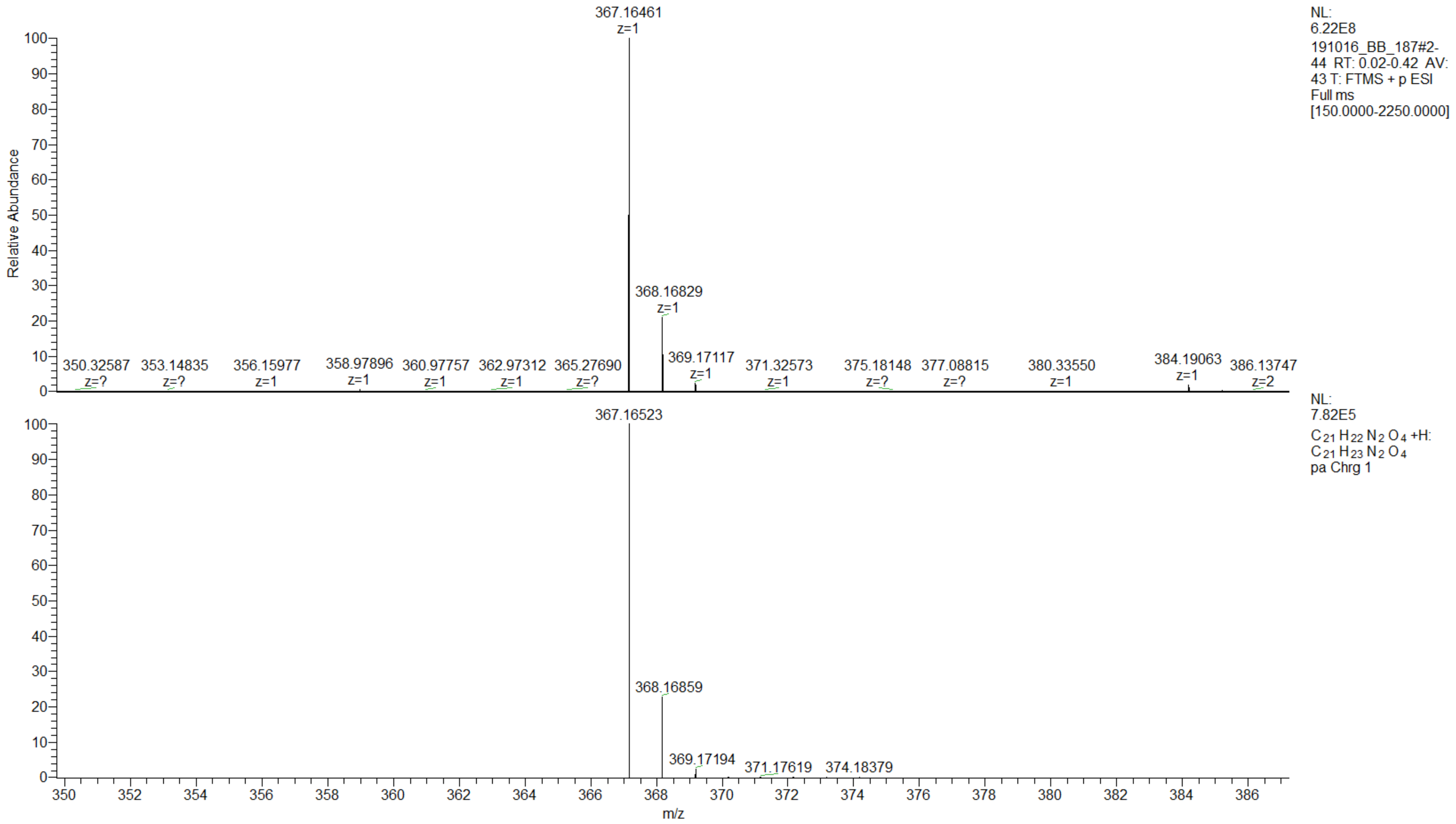

Figure S79. ESI HRMS spectrum of ethyl 4-(11-methyl-6,12-dioxo-11,12-dihydrodibenzo[*b*,*f*][1,5]diazocin-5(6*H*)-yl)butanoate (**10c**).

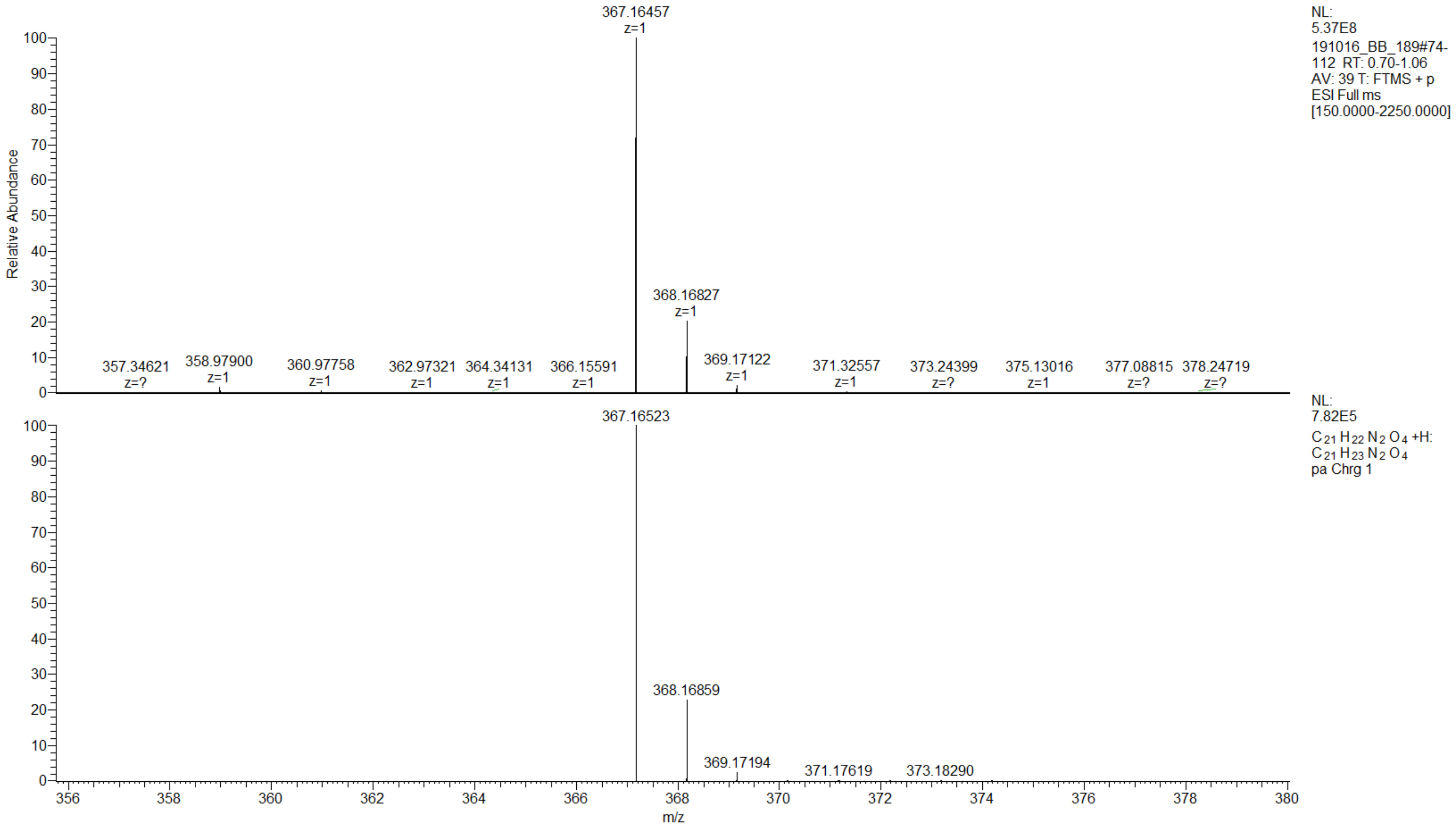

Figure S80. ESI HRMS spectrum of methyl 5-(11-methyl-6,12-dioxo-11,12-dihydrodibenzo[*b*,*f*][1,5]diazocin-5(6*H*)-yl)pentanoate (**10d**).

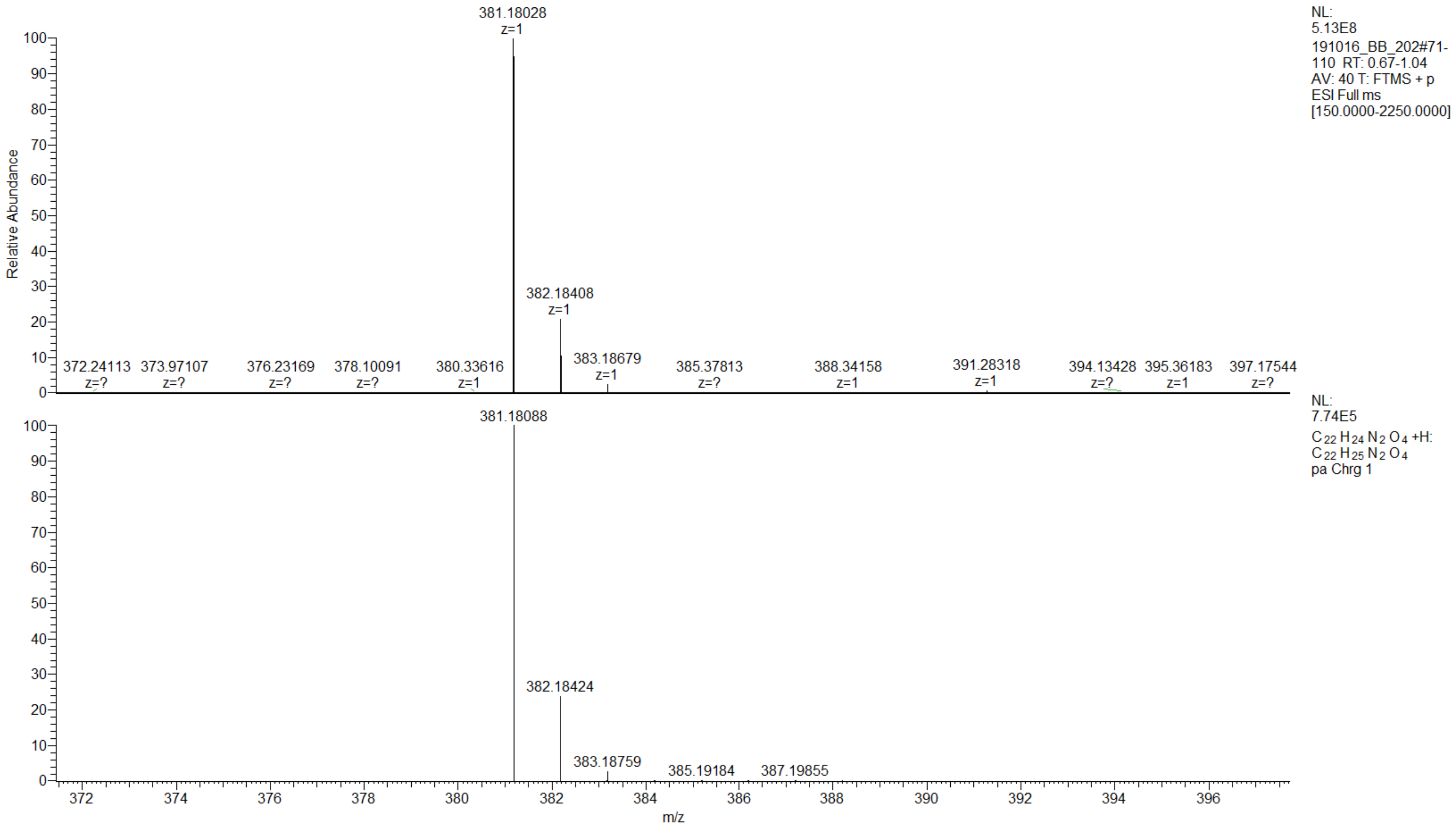

Figure S81. ESI HRMS spectrum of methyl 6-(11-methyl-6,12-dioxo-11,12-dihydrodibenzo[*b*,*f*][1,5]diazocin-5(6*H*)-yl)hexanoate (**10e**).

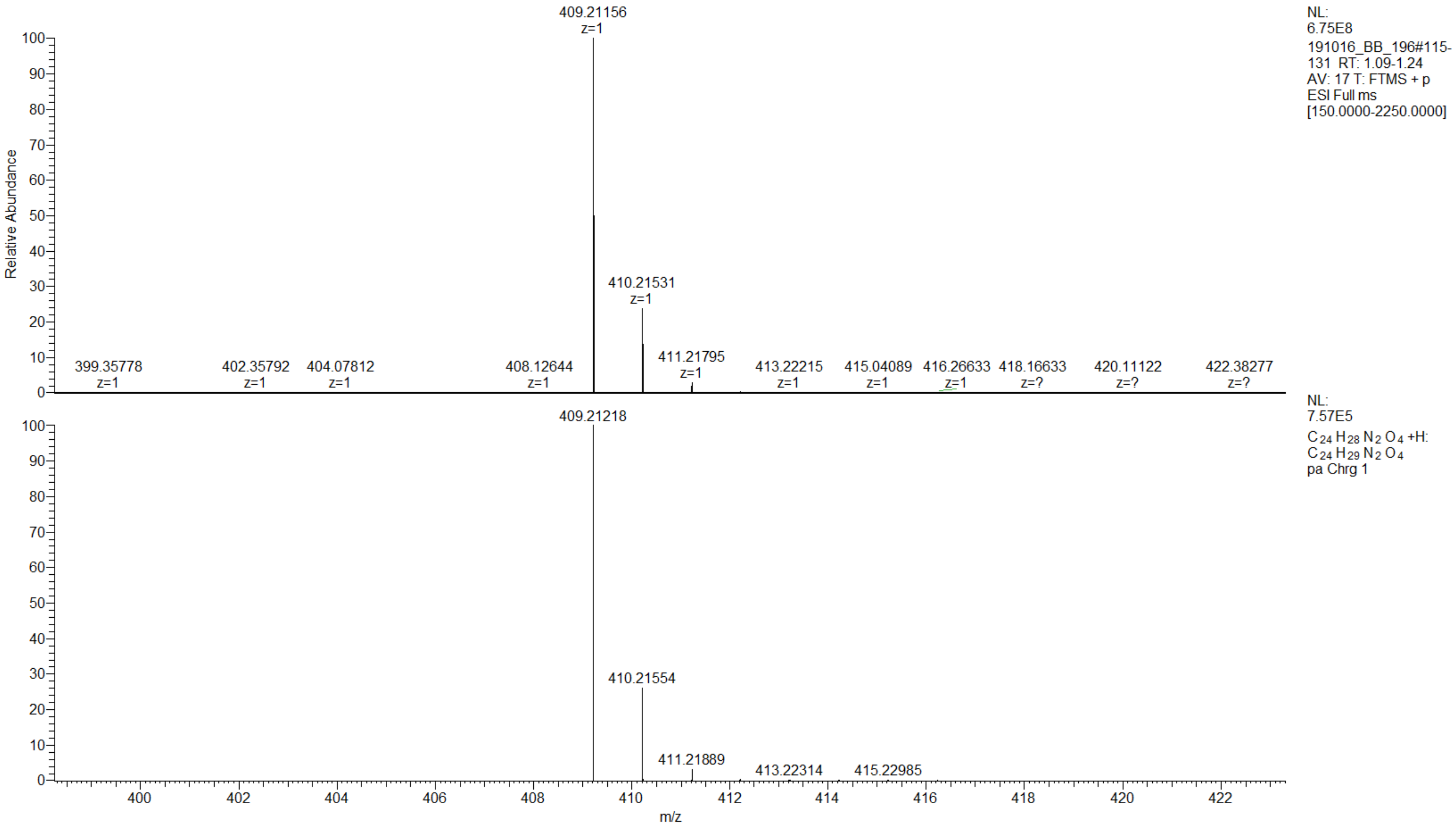

Figure S82. ESI HRMS spectrum of ethyl 7-(11-methyl-6,12-dioxo-11,12-dihydrodibenzo[*b*,*f*][1,5]diazocin-5(6*H*)-yl)heptanoate (**10f**).

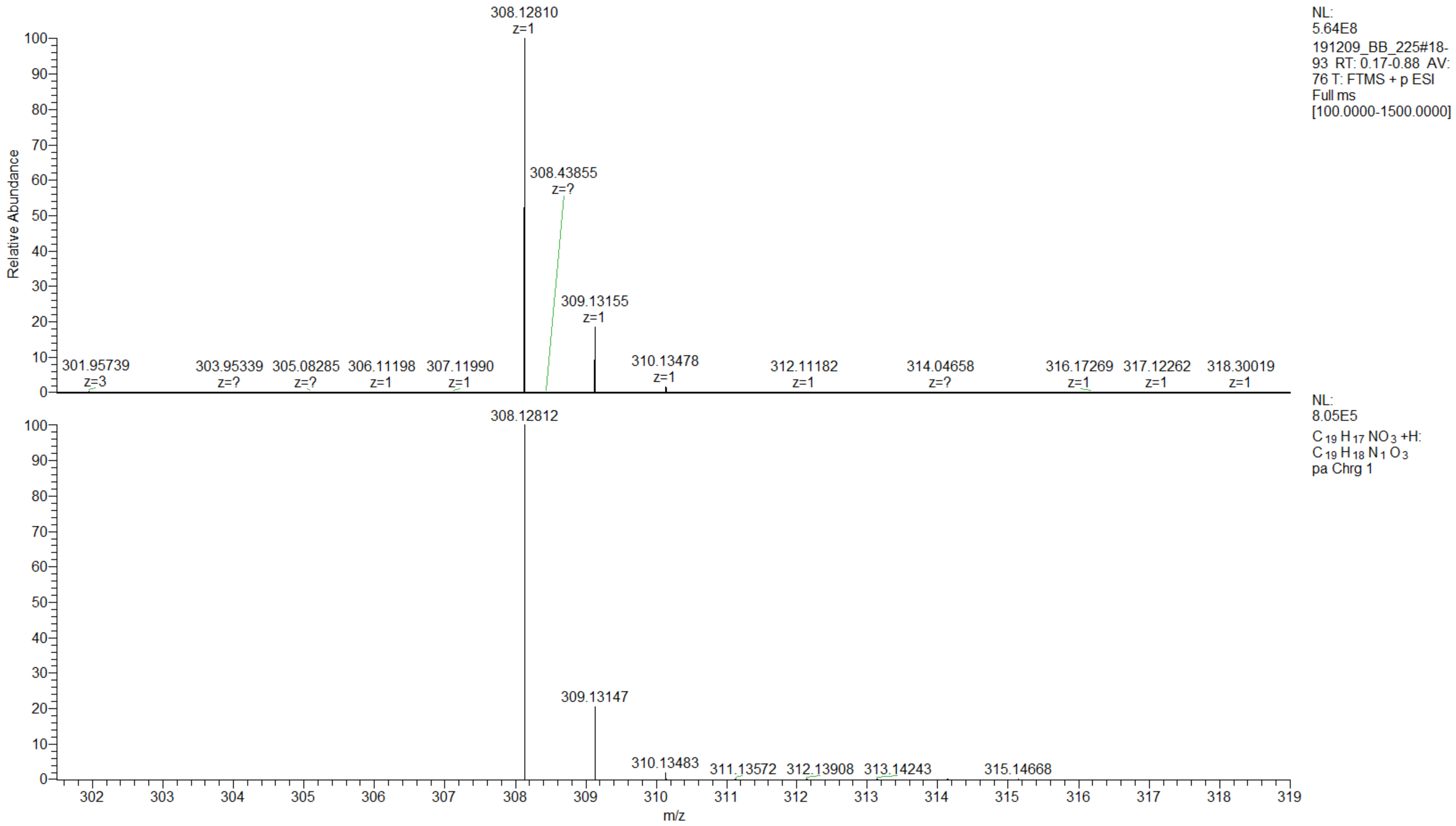

Figure S83. ESI HRMS spectrum of ethyl 2-(6-oxodibenzo[*b*,*f*]azocin-5(6*H*)-yl)acetate (**10g**).

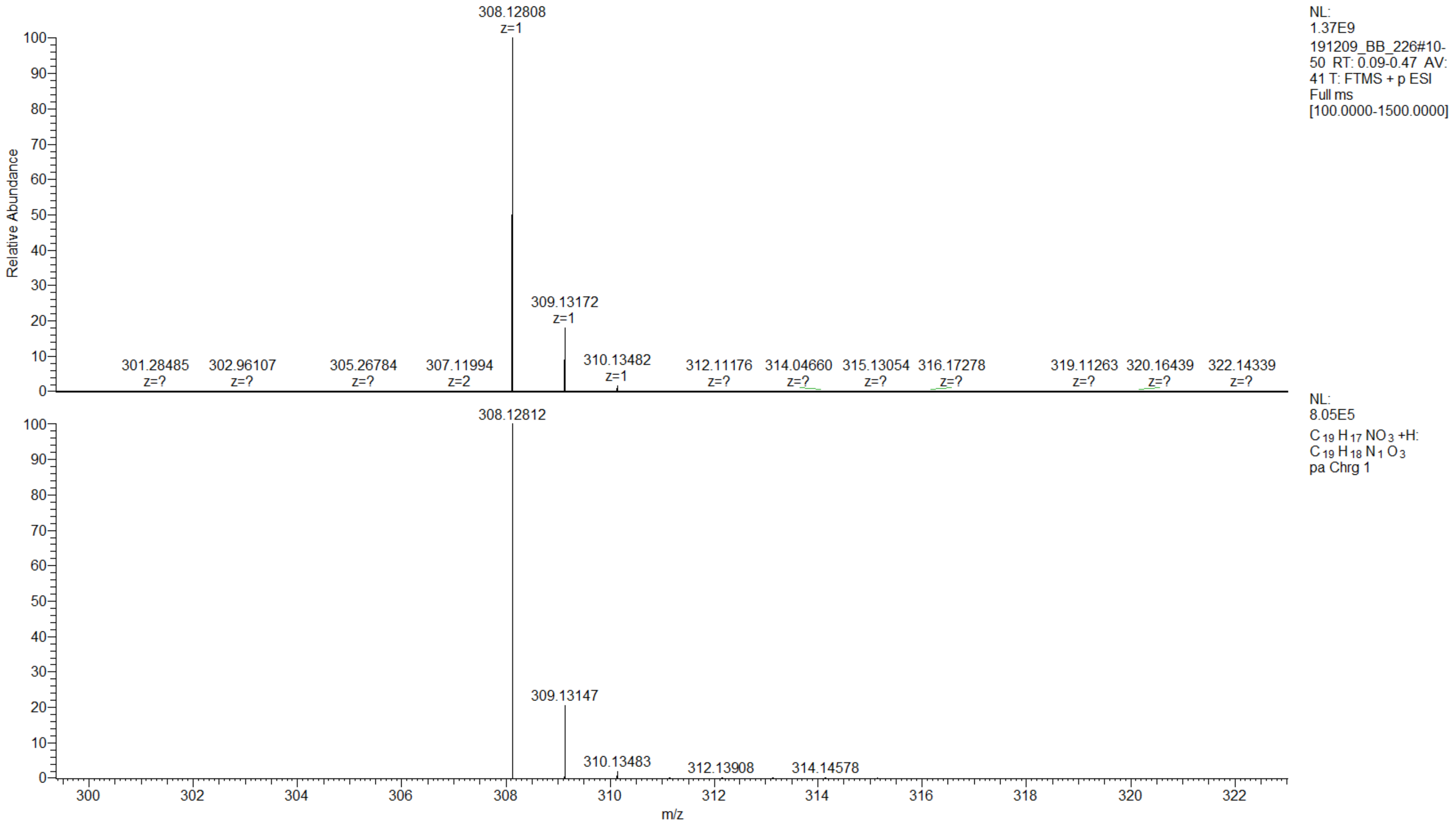

Figure S84. ESI HRMS spectrum of methyl 3-(6-oxodibenzo[*b,f*]azocin-5(6*H*)-yl)propanoate (**10h**).

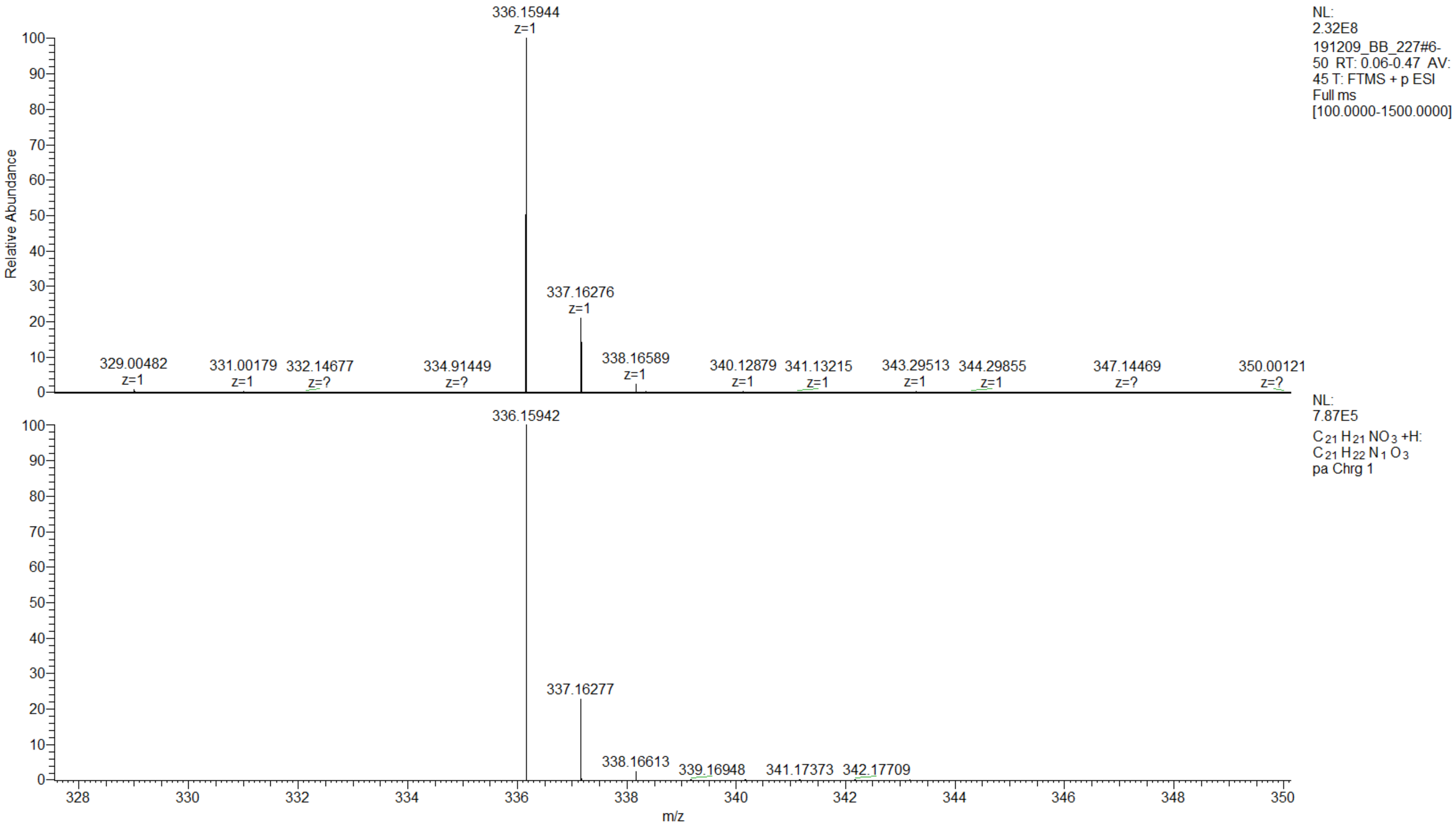

Figure S85. ESI HRMS spectrum of ethyl 4-(6-oxodibenzo[*b*,*f*]azocin-5(6*H*)-yl)butanoate (**10i**).

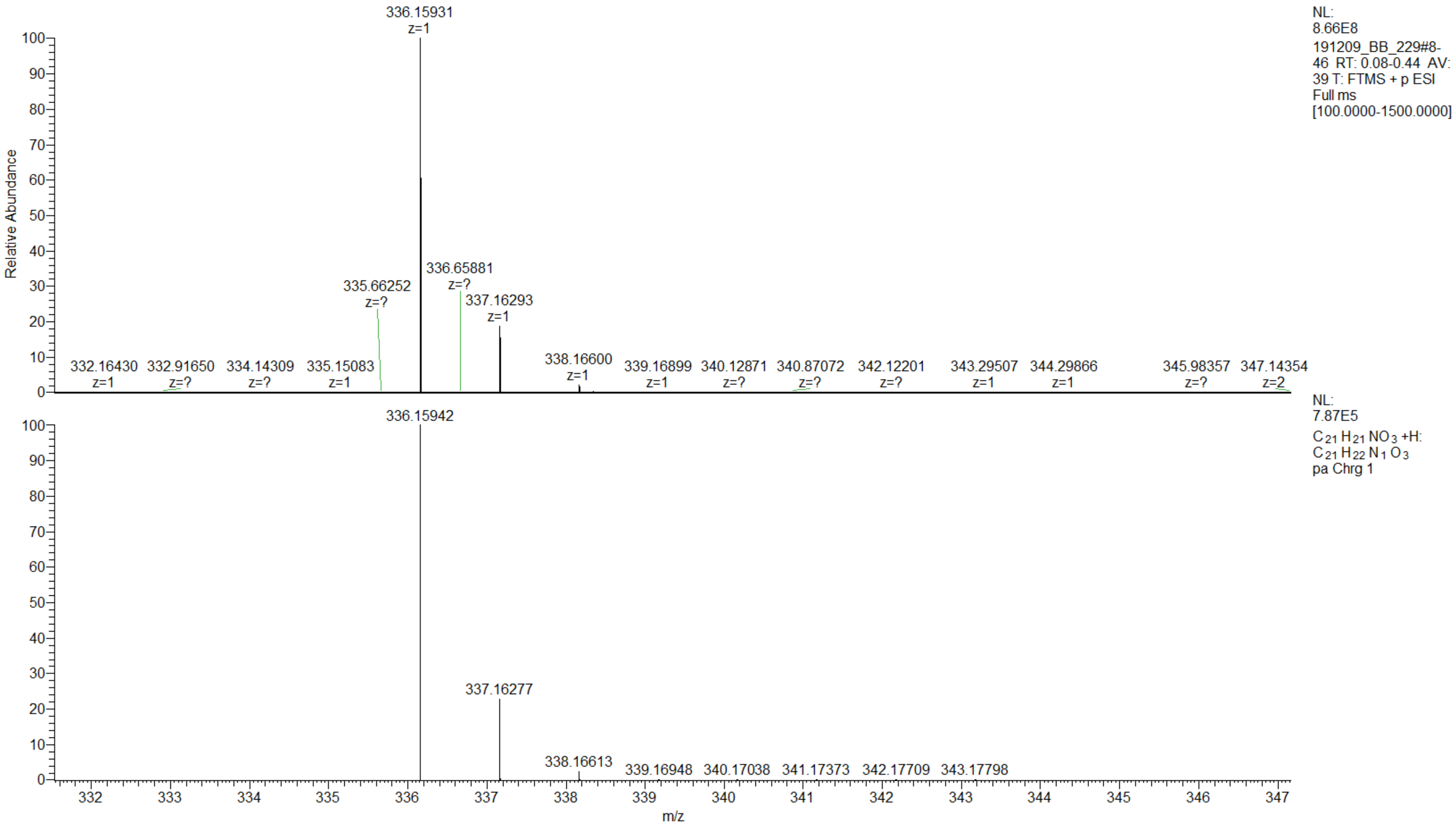

Figure S86. ESI HRMS spectrum of methyl 5-(6-oxodibenzo[*b*,*f*]azocin-5(6*H*)-yl)pentanoate (**10j**).

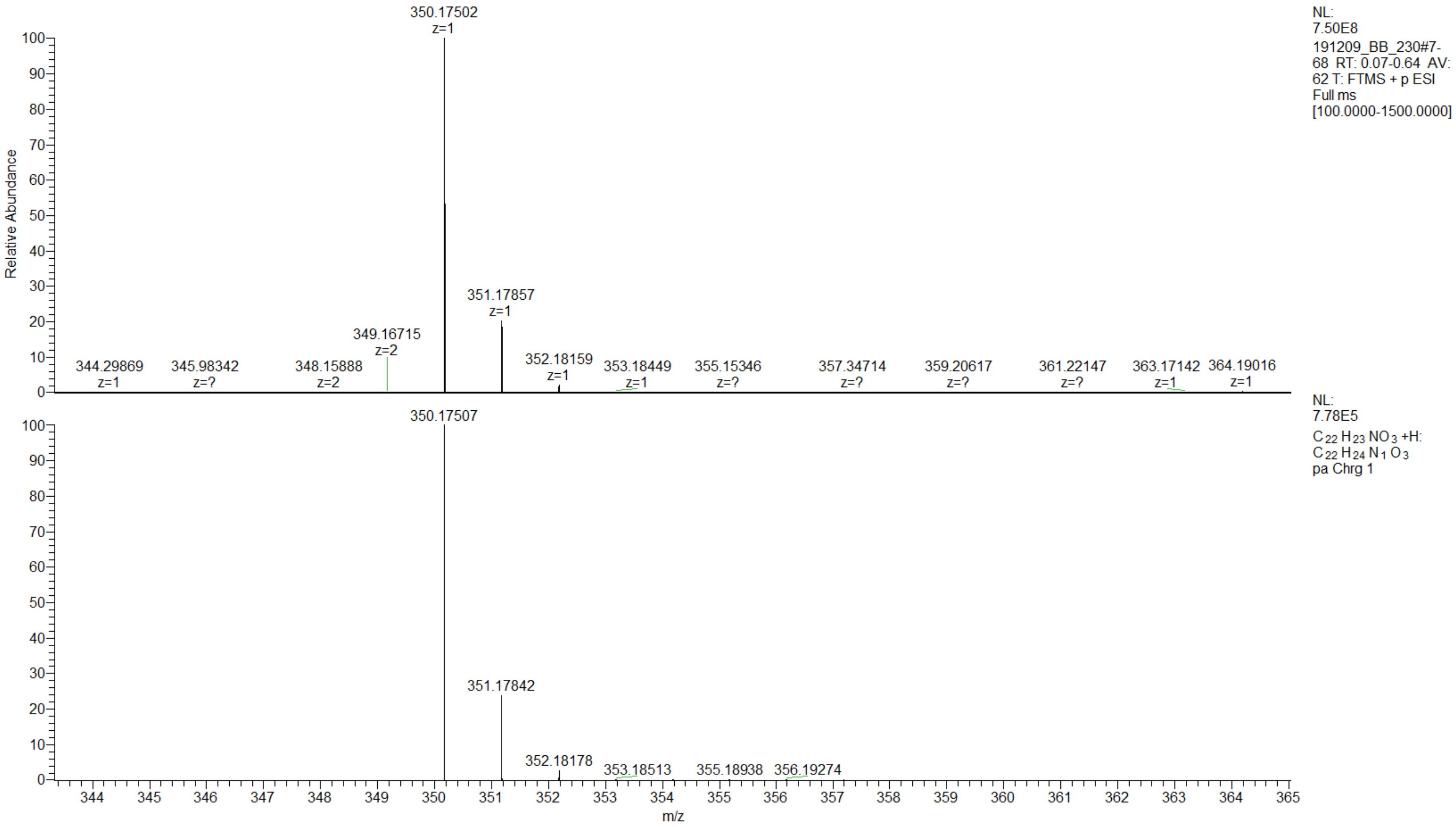

Figure S87. ESI HRMS spectrum of methyl 6-(6-oxodibenzo[*b*,*f*]azocin-5(6*H*)-yl)hexanoate (**10k**).

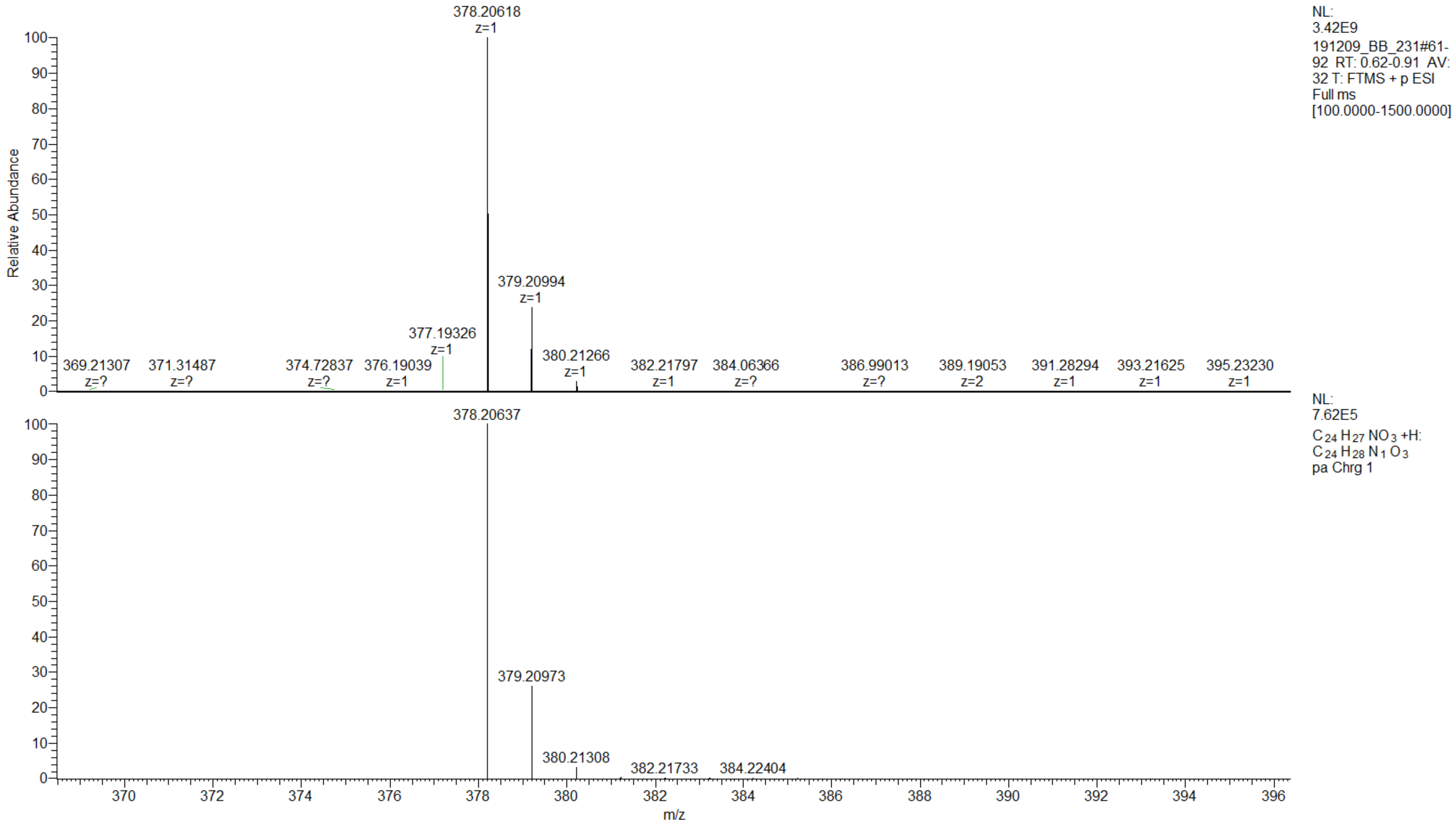

Figure S88. ESI HRMS spectrum of ethyl 7-(6-oxodibenzo[*b*,*f*]azocin-5(6*H*)-yl)heptanoate (**10l**).

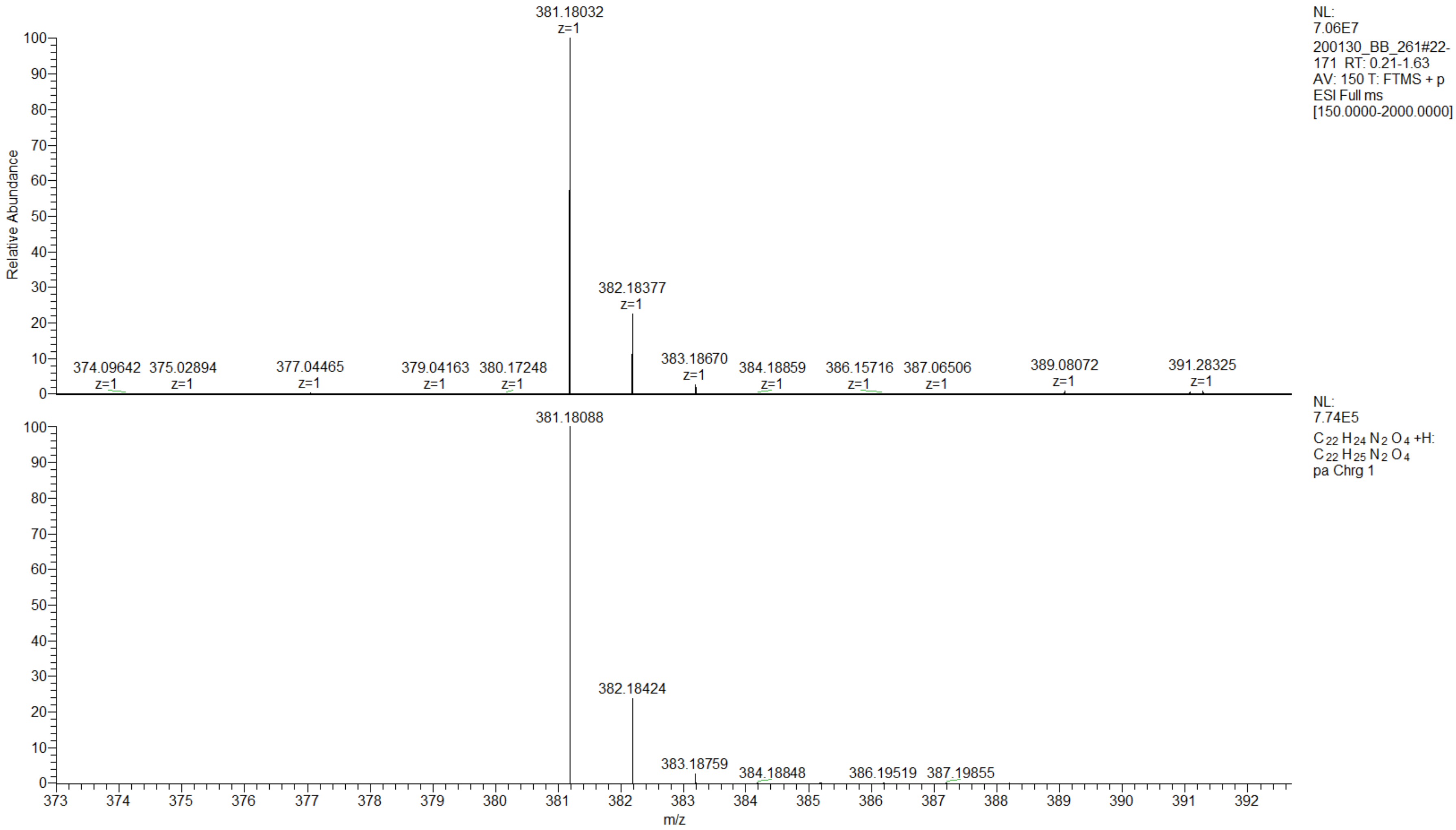

Figure S89. ESI HRMS spectrum of methyl 6-(12-methyl-6,11-dioxo-11,12-dihydrodibenzo[*b*,*f*][1,4]diazocin-5(6*H*)-yl)hexanoate (**10m**).

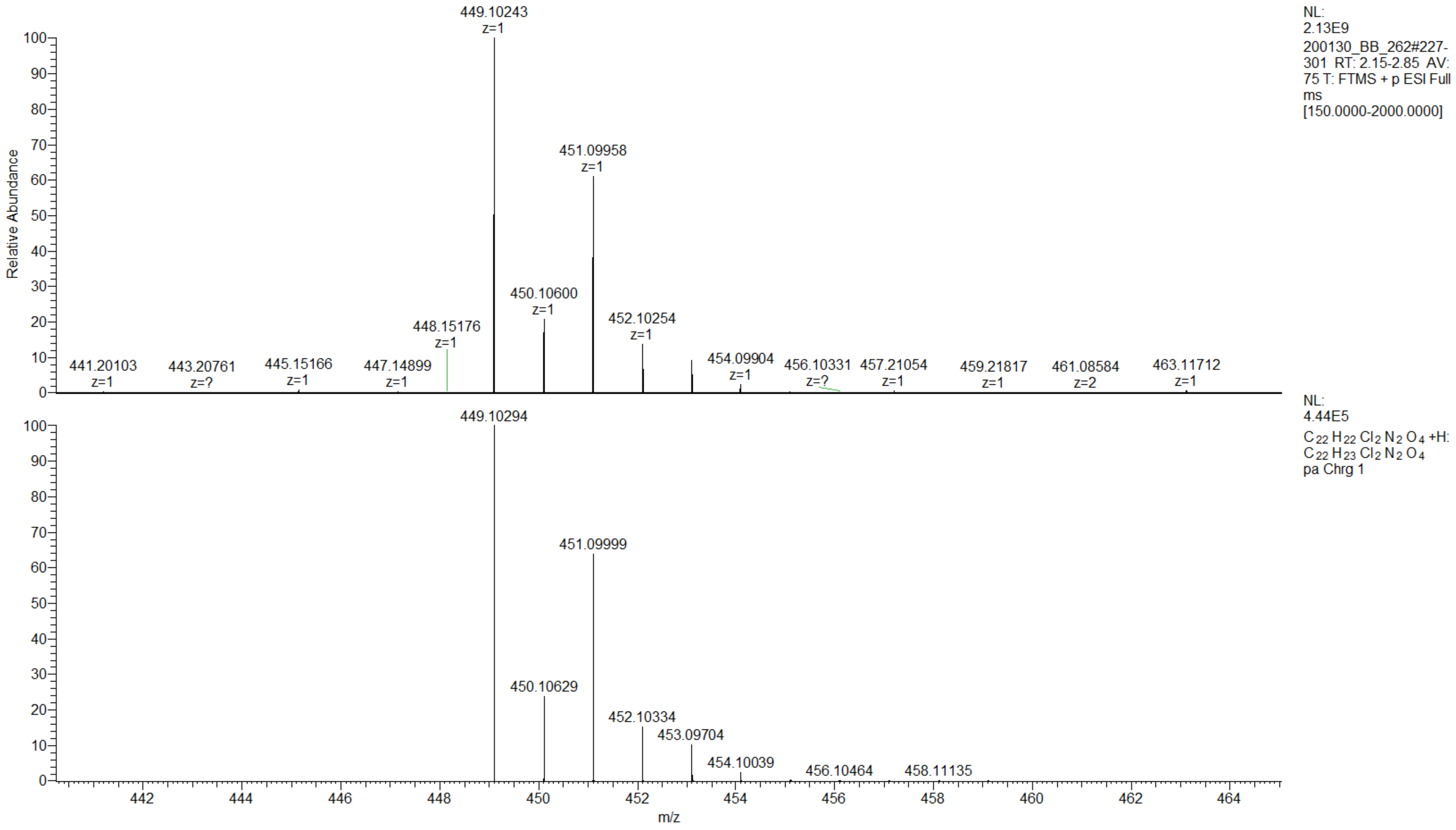

Figure S90. ESI HRMS spectrum of methyl 6-(8,9-dichloro-12-methyl-6,11-dioxo-11,12-dihydrodibenzo[*b*,*f*][1,4]diazocin-5(6*H*)-yl) hexanoate (**10n**).

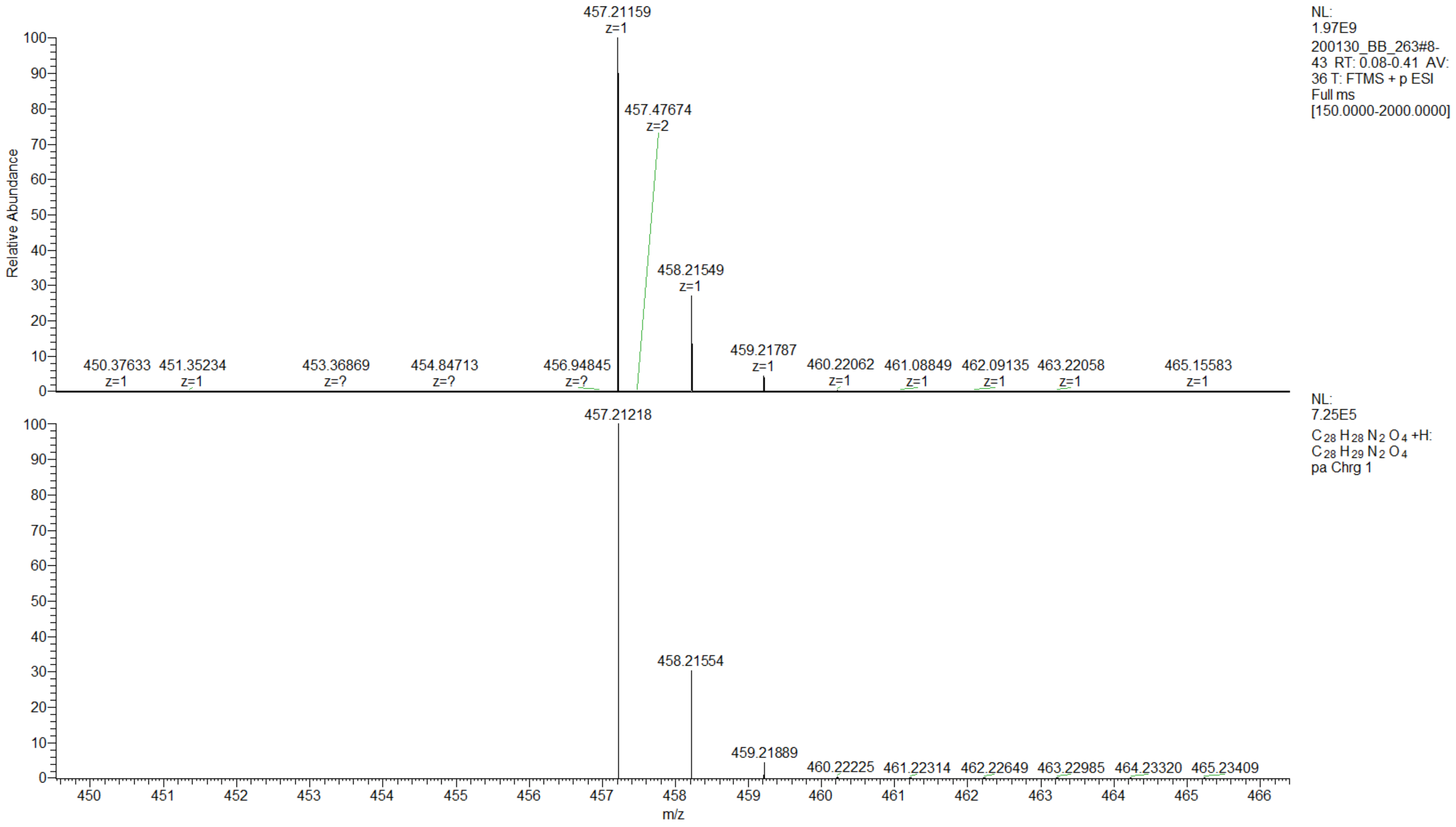

Figure S91. ESI HRMS spectrum of methyl 6-(12-benzyl-6,11-dioxo-11,12-dihydrodibenzo[*b*,*f*][1,4]diazocin-5(6*H*)-yl)hexanoate (**10o**).

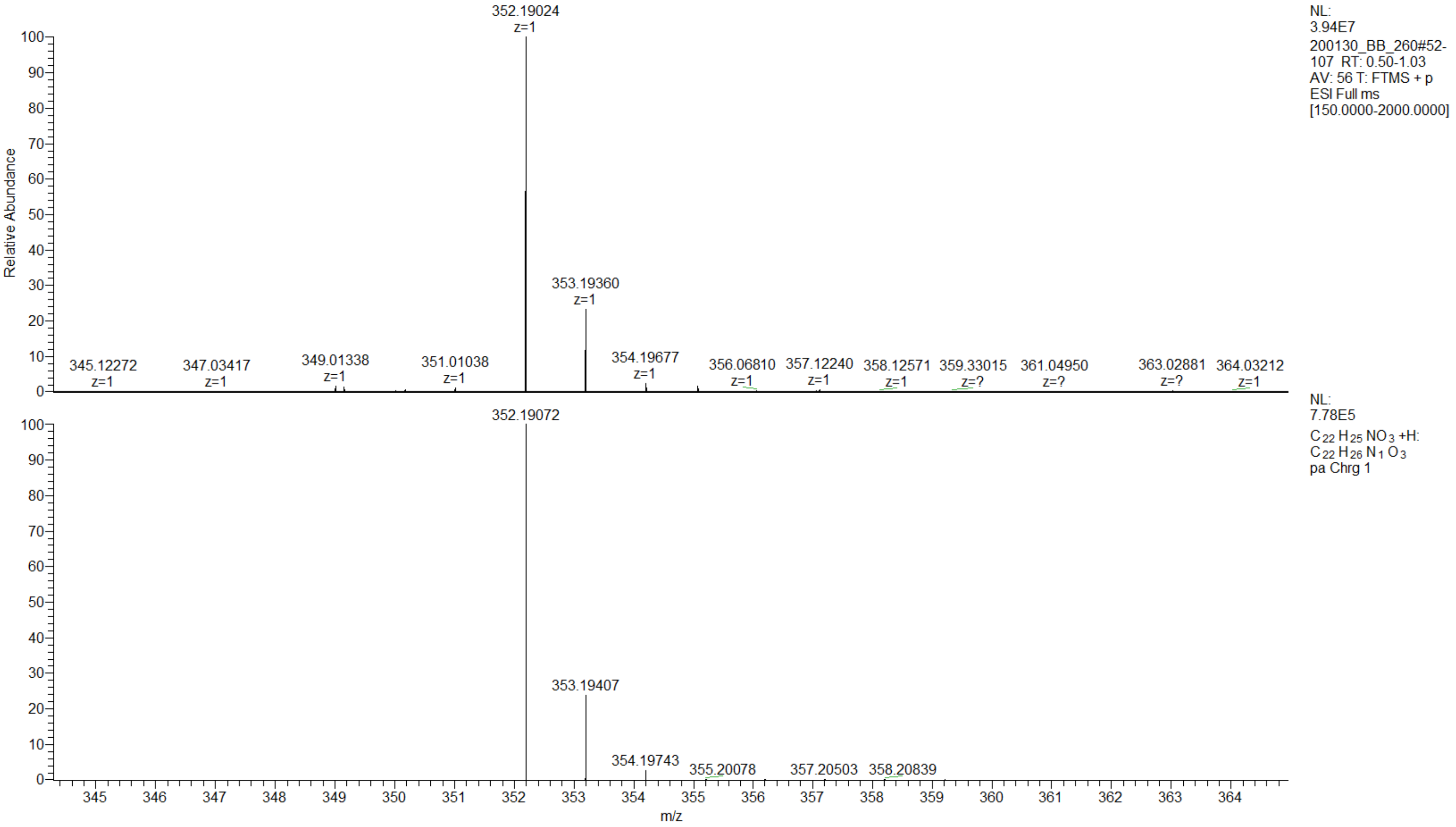

Figure S92. ESI HRMS spectrum of methyl 6-(6-oxo-11,12-dihydrodibenzo[*b*,*f*]azocin-5(6*H*)-yl)hexanoate (**10p**).

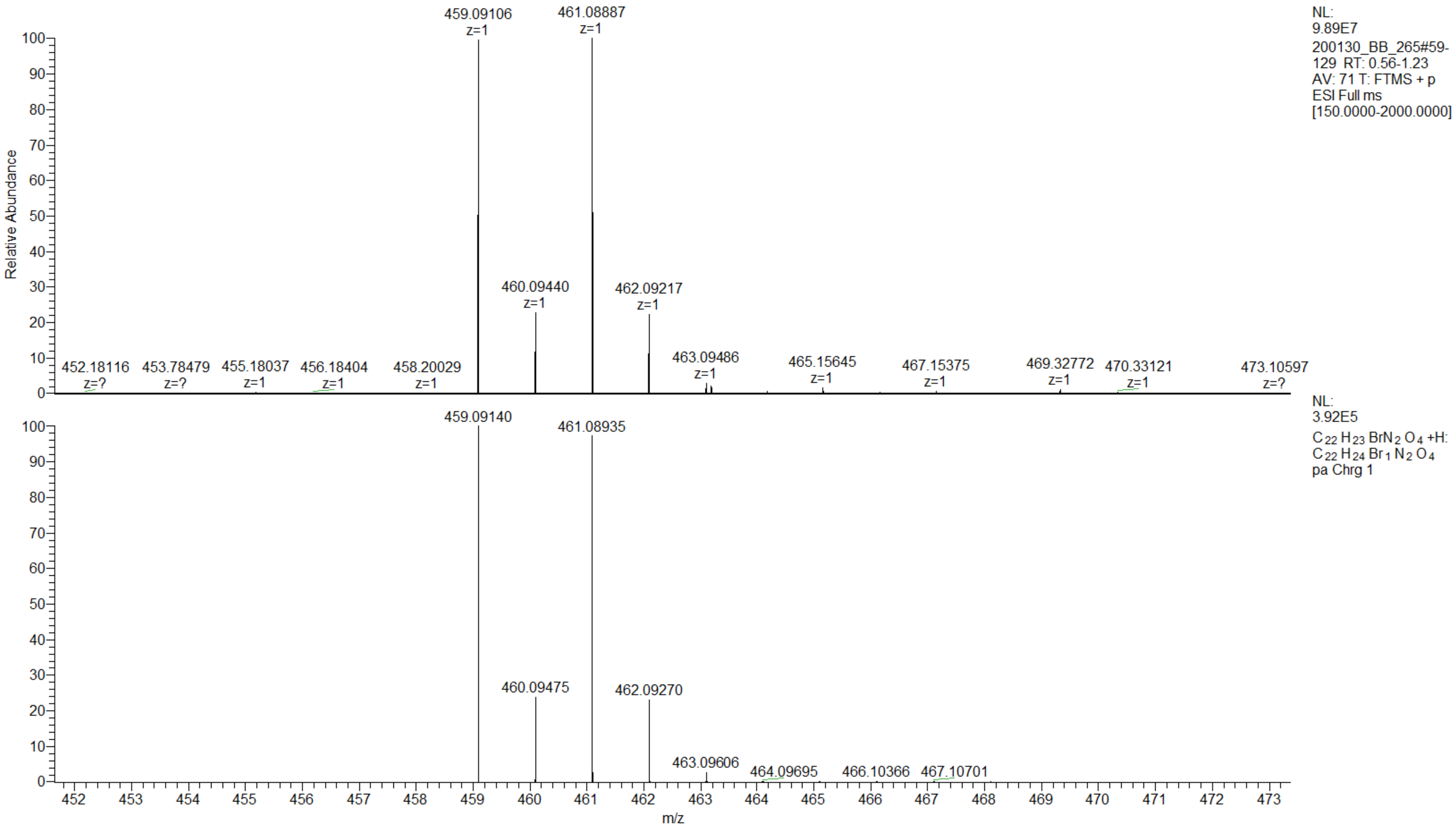

Figure S93. ESI HRMS spectrum of methyl 6-(2-bromo-11-methyl-6,12-dioxo-11,12-dihydrodibenzo[*b*,*f*][1,5]diazocin-5(6*H*)-yl)hexanoate (**10r**).

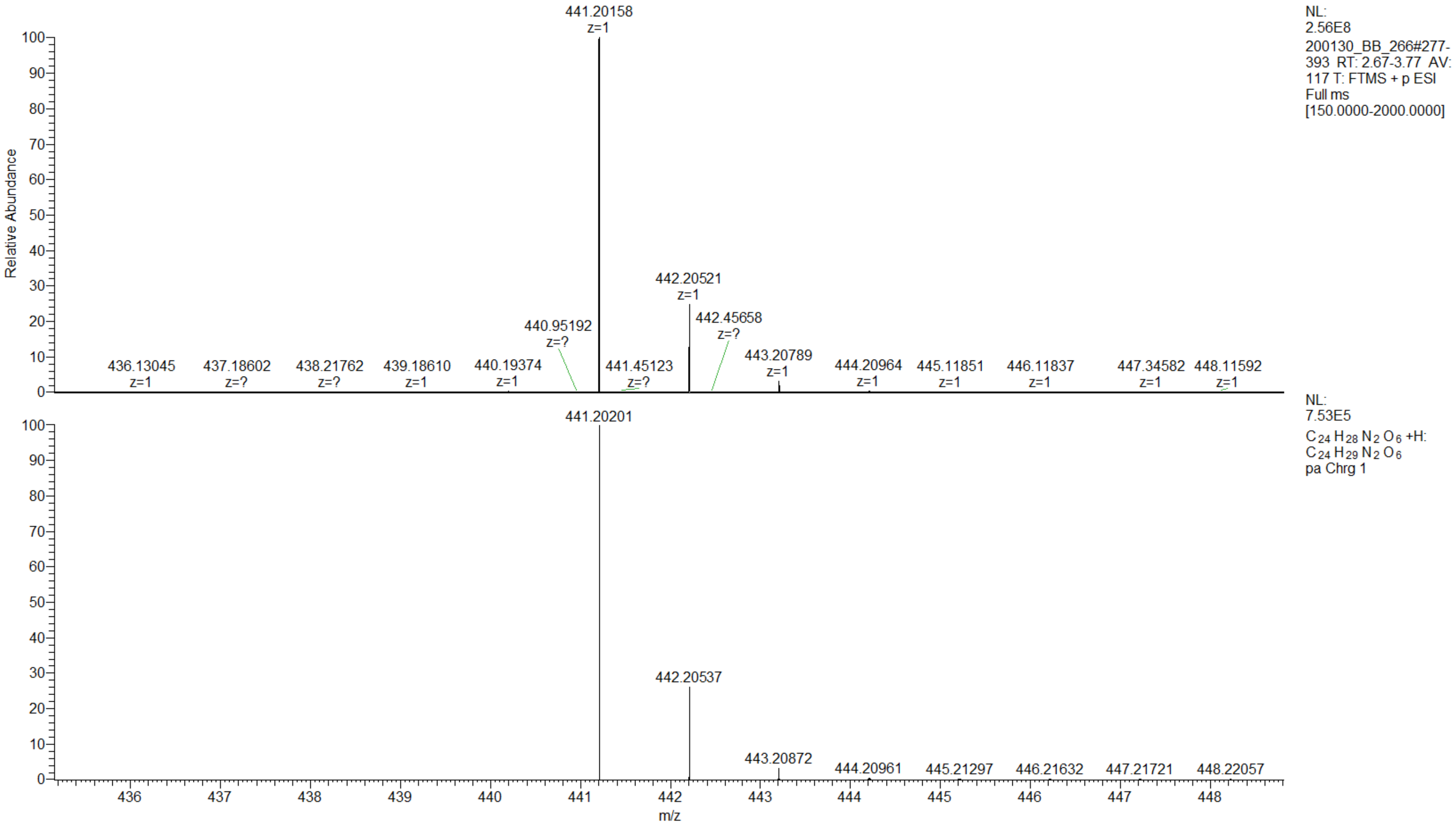

Figure S94. ESI HRMS spectrum of methyl 6-(2,3-dimethoxy-11-methyl-6,12-dioxo-11,12-dihydrodibenzo[*b*,*f*][1,5]diazocin-5(6*H*)-yl)hexanoate (**10s**).

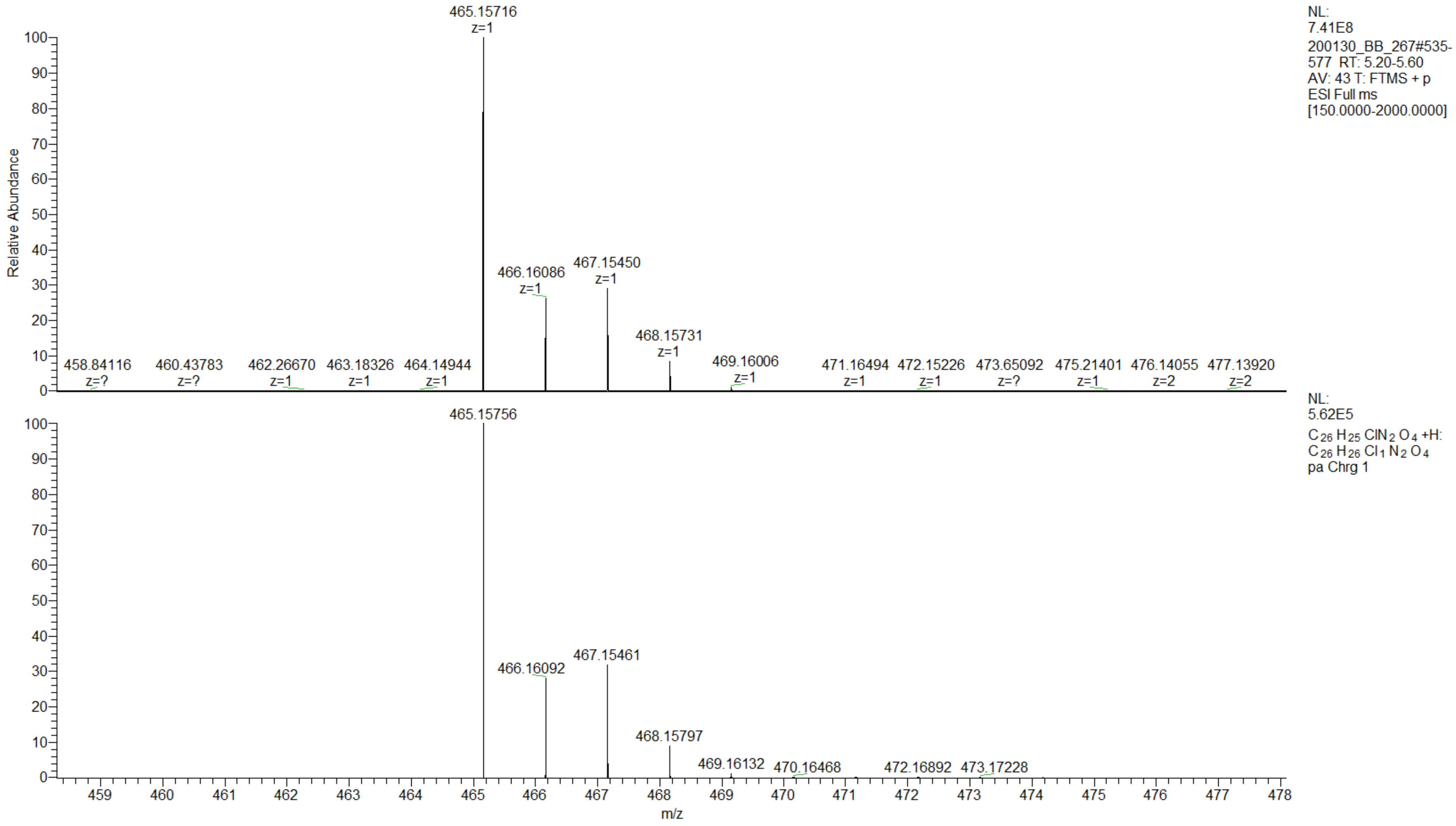

Figure S95. ESI HRMS spectrum of methyl 6-(5-methyl-6,14-dioxo-5,14-dihydrobenzonaphtho[2,3-*f*][1,5]diazocin-13(6*H*)-yl)hexanoate (**10t**).

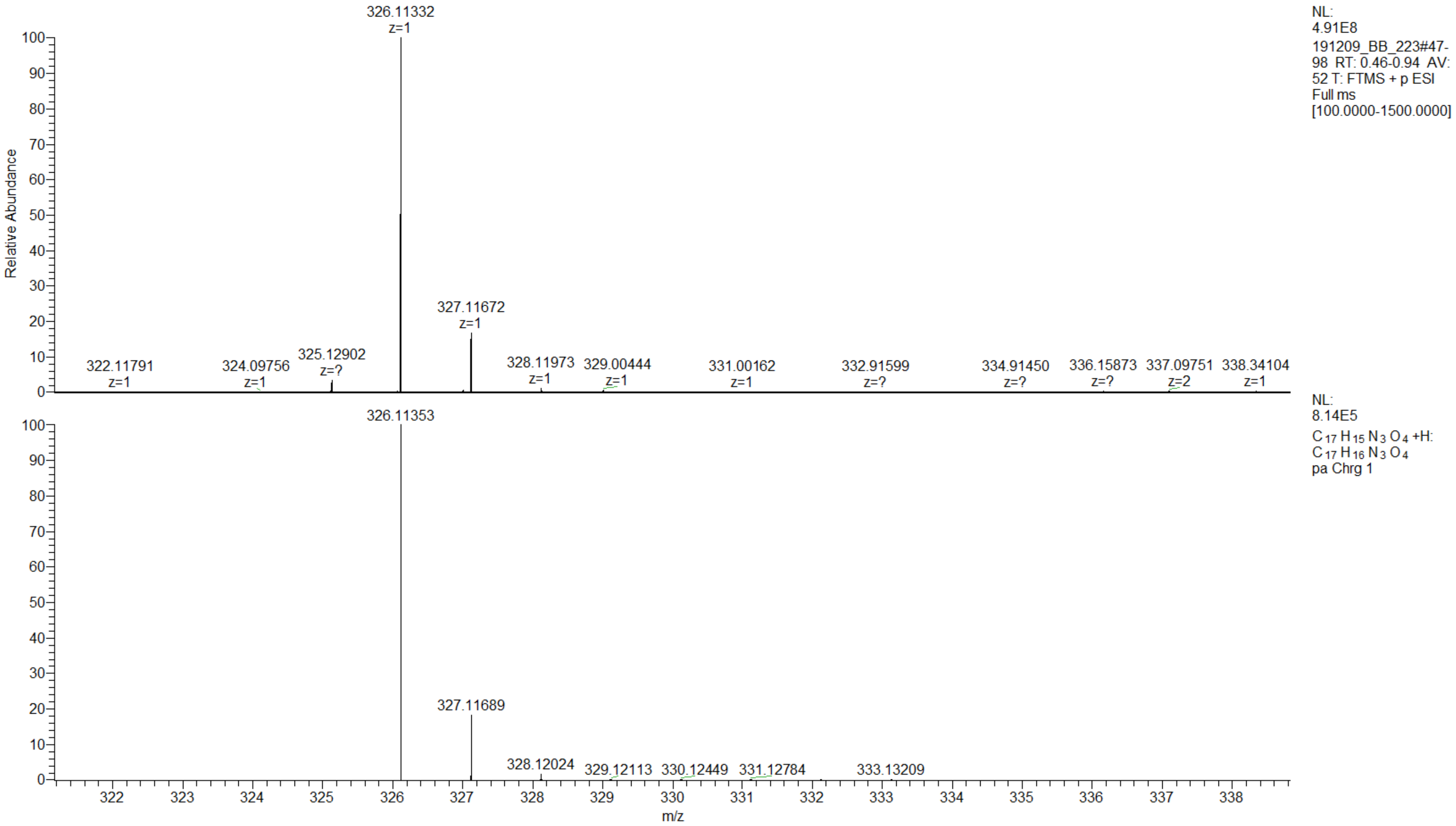

Figure S96. ESI HRMS spectrum of *N*-hydroxy-2-(11-methyl-6,12-dioxo-11,12-dihydrodibenzo[*b*,*f*][1,5]diazocin-5(6*H*)-yl)acetamide (**7a**).

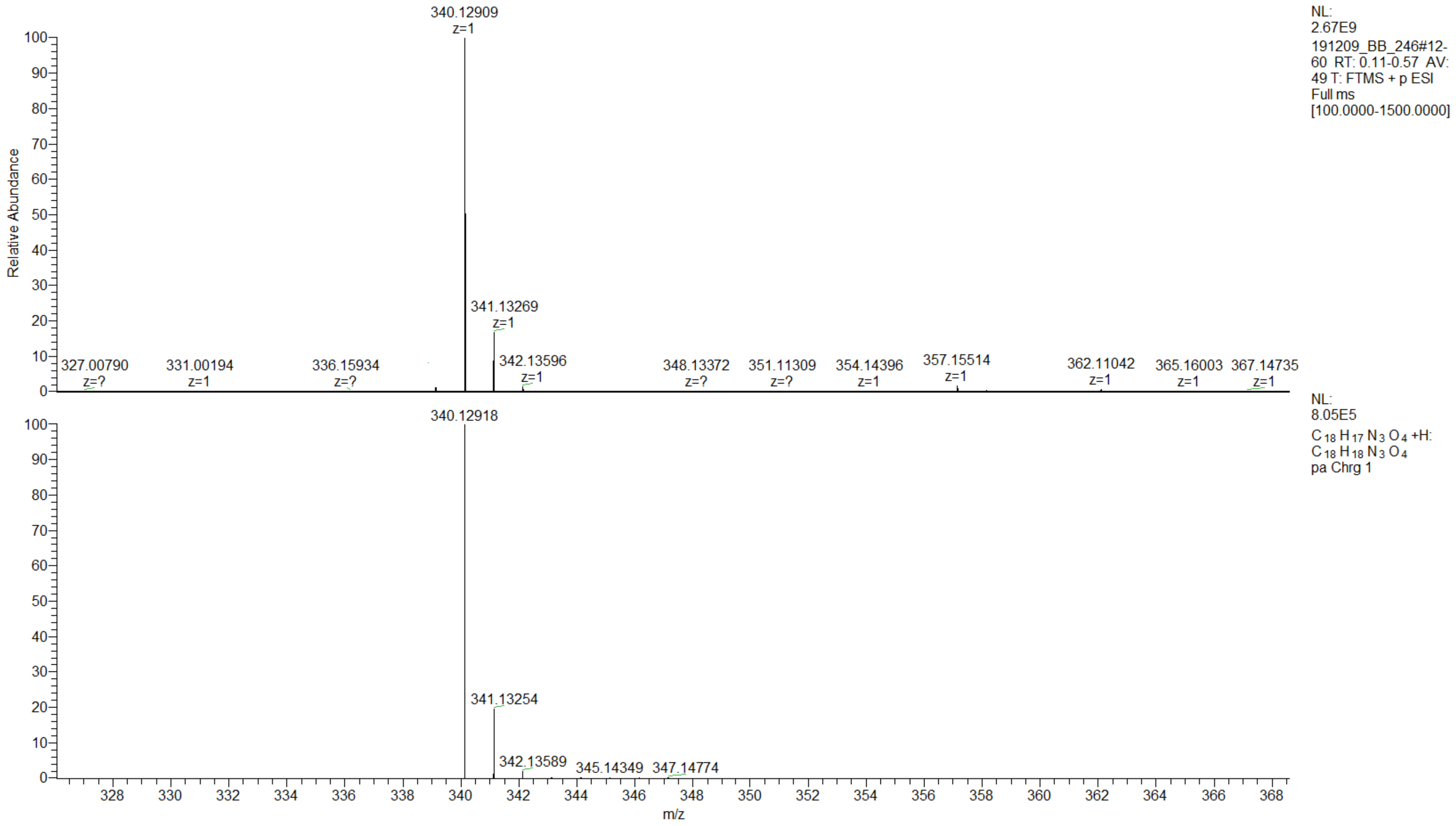

Figure S97. ESI HRMS spectrum of *N*-hydroxy-3-(11-methyl-6,12-dioxo-11,12-dihydrodibenzo[*b*,*f*][1,5]diazocin-5(6*H*)-yl)propanamide (**7b**).

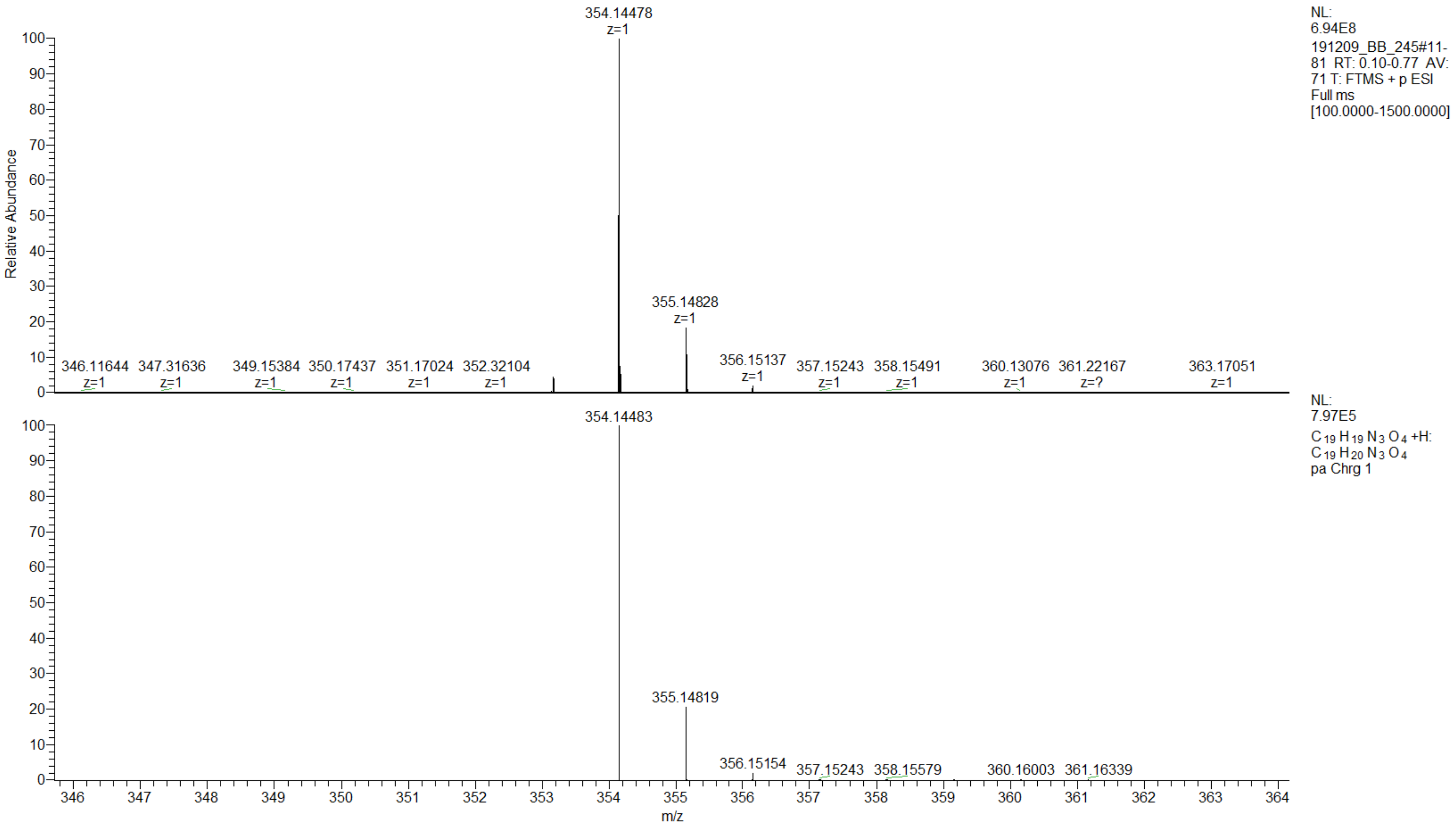

Figure S98. ESI HRMS spectrum of *N*-hydroxy-4-(11-methyl-6,12-dioxo-11,12-dihydrodibenzo[*b*,*f*][1,5]diazocin-5(6*H*)-yl)butanamide (**7c**).

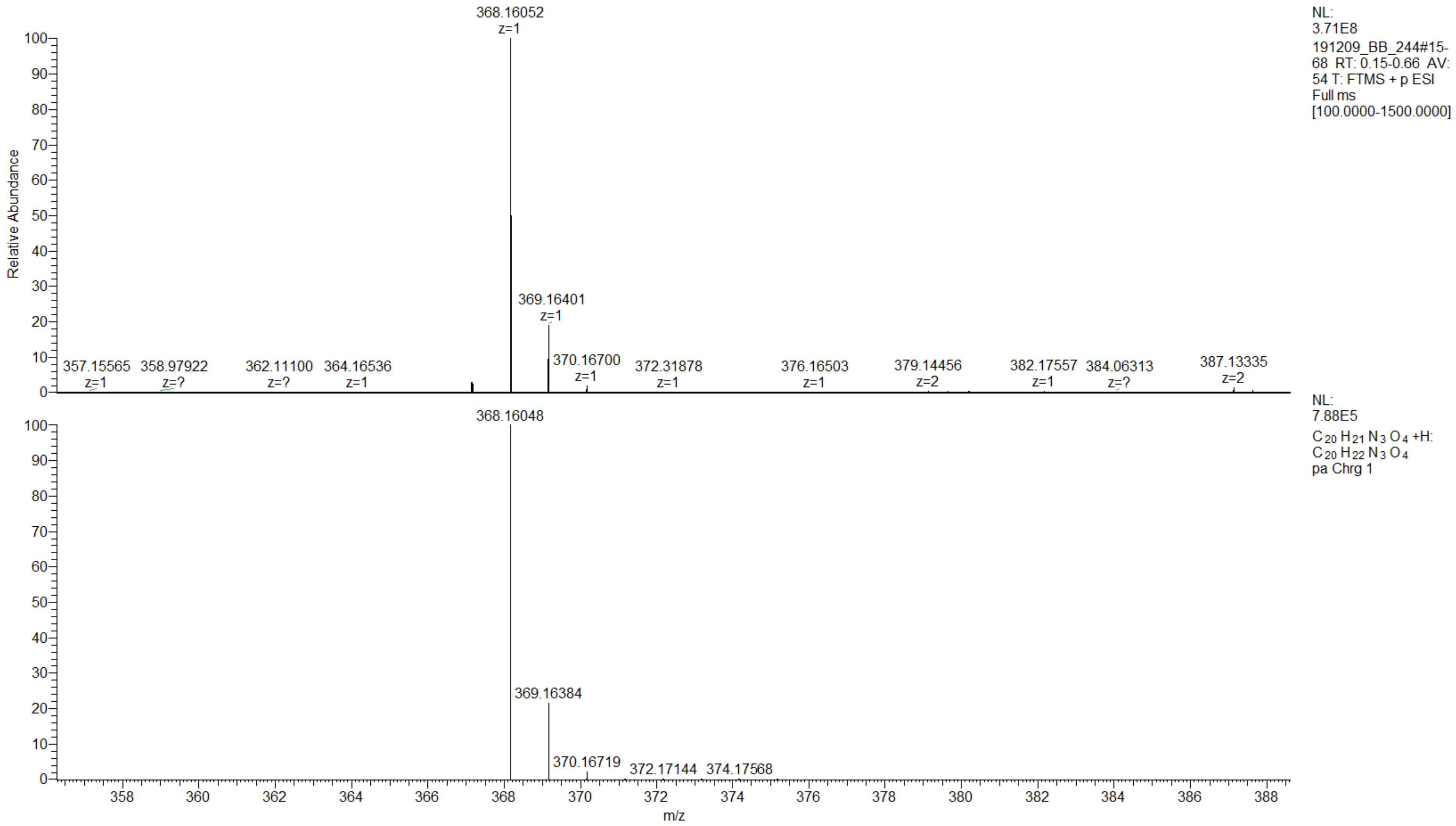

Figure S99. ESI HRMS spectrum of *N*-hydroxy-5-(11-methyl-6,12-dioxo-11,12-dihydrodibenzo[*b*,*f*][1,5]diazocin-5(6*H*)-yl)pentanamide (**7d**).

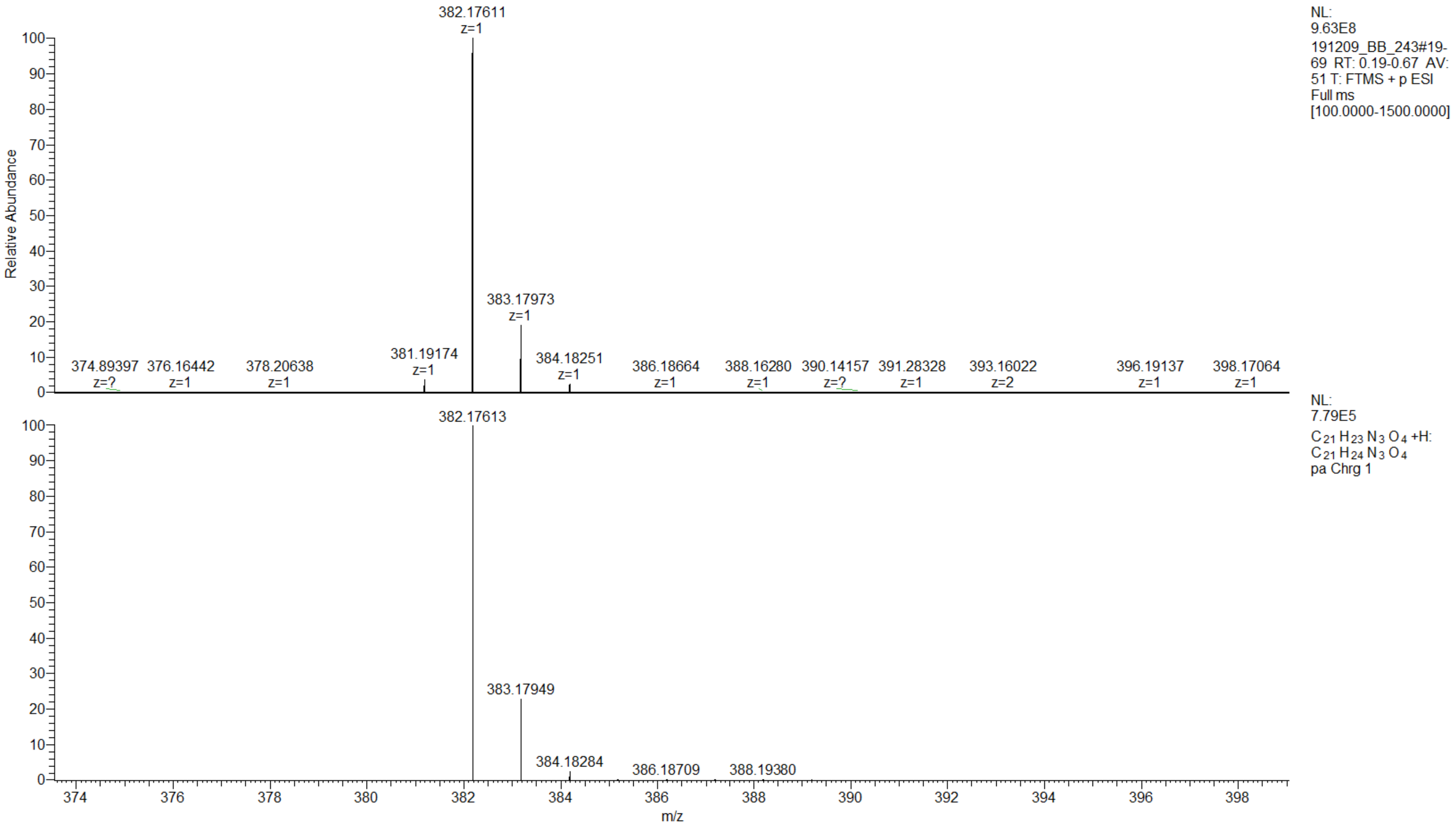

Figure S100. ESI HRMS spectrum of *N*-hydroxy-6-(11-methyl-6,12-dioxo-11,12-dihydrodibenzo[*b,f*][1,5]diazocin-5(6*H*)-yl)hexanamide (**7e**).

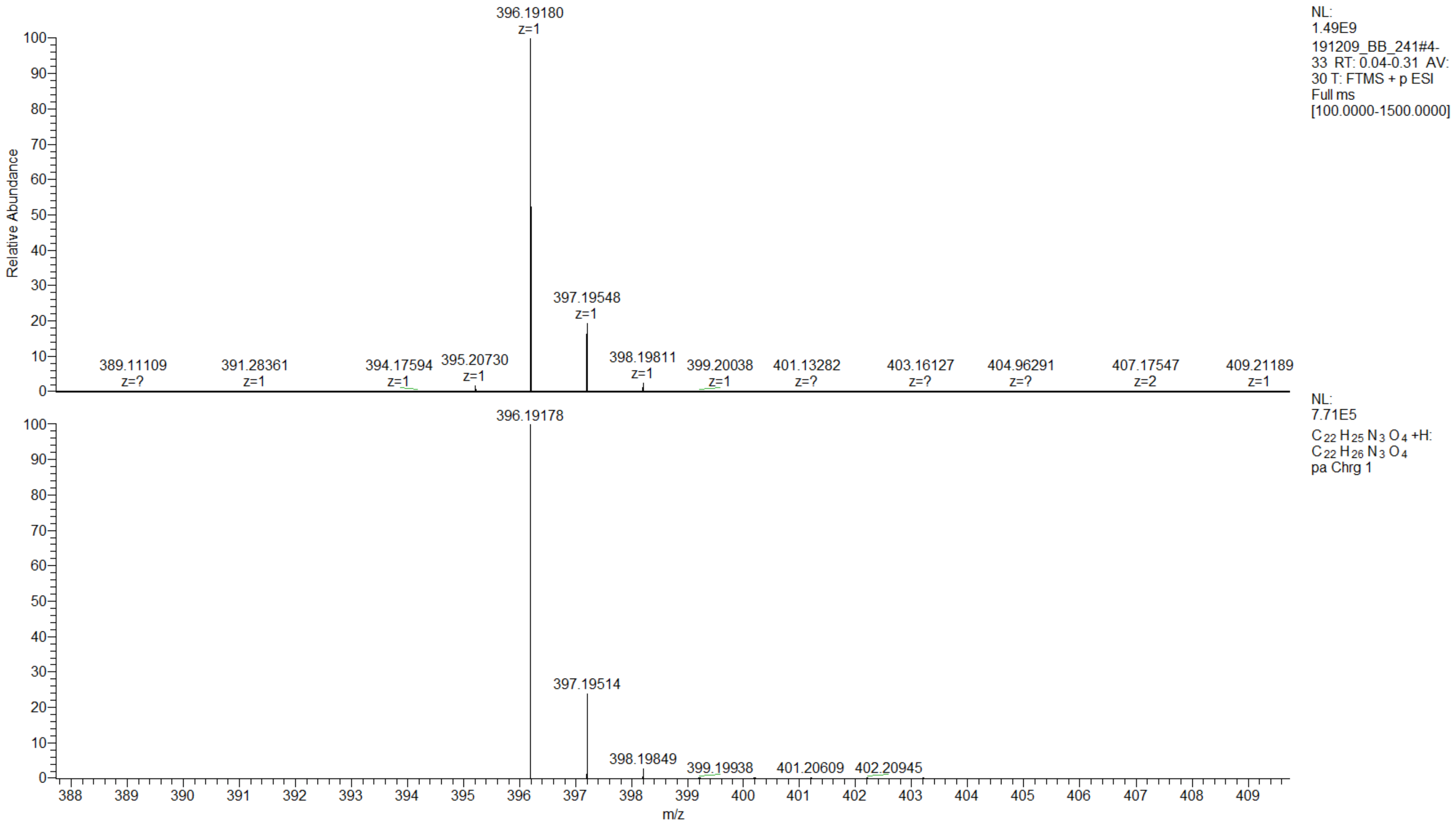

Figure S101. ESI HRMS spectrum of *N*-hydroxy-7-(11-methyl-6,12-dioxo-11,12-dihydrodibenzo[*b*,*f*][1,5]diazocin-5(6*H*)-yl)heptanamide (**7f**).

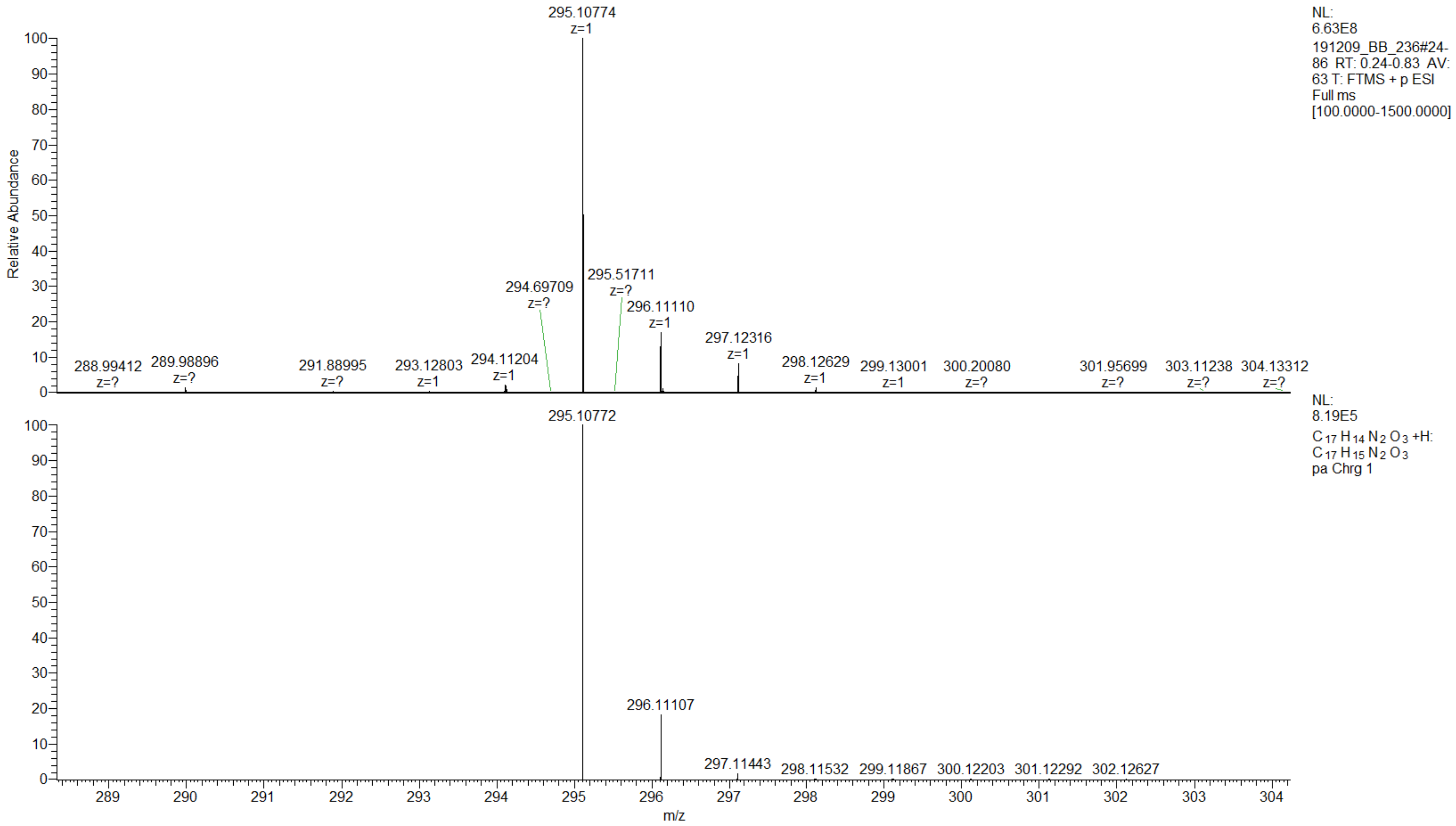

Figure S102. ESI HRMS spectrum of *N*-hydroxy-2-(6-oxodibenzo[*b*,f]azocin-5(6H)-yl)acetamide (**7g**).

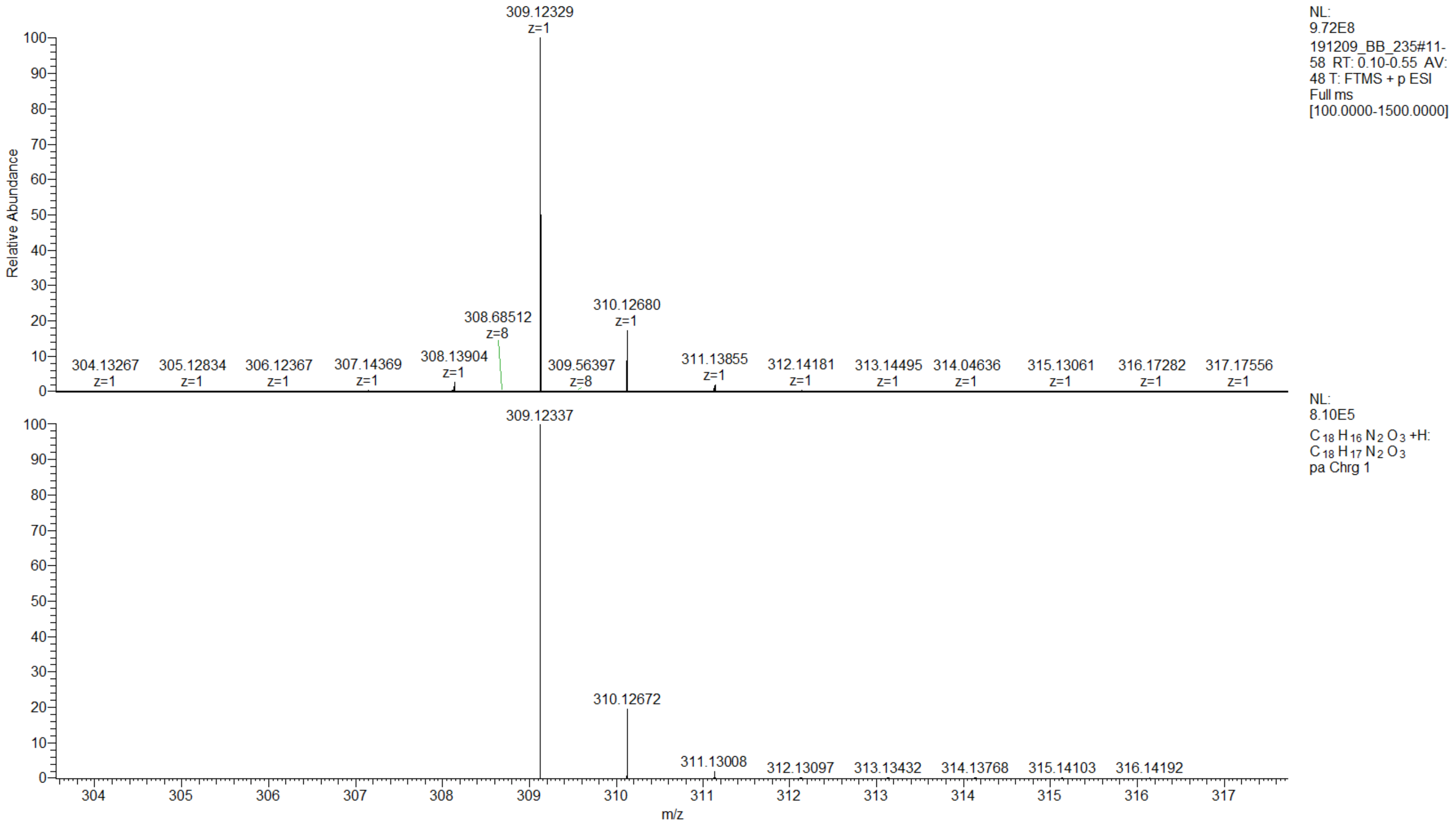

Figure S103. ESI HRMS spectrum of *N*-hydroxy-3-(6-oxodibenzo[*b,f*]azocin-5(6*H*)-yl)propanamide (**7h**).

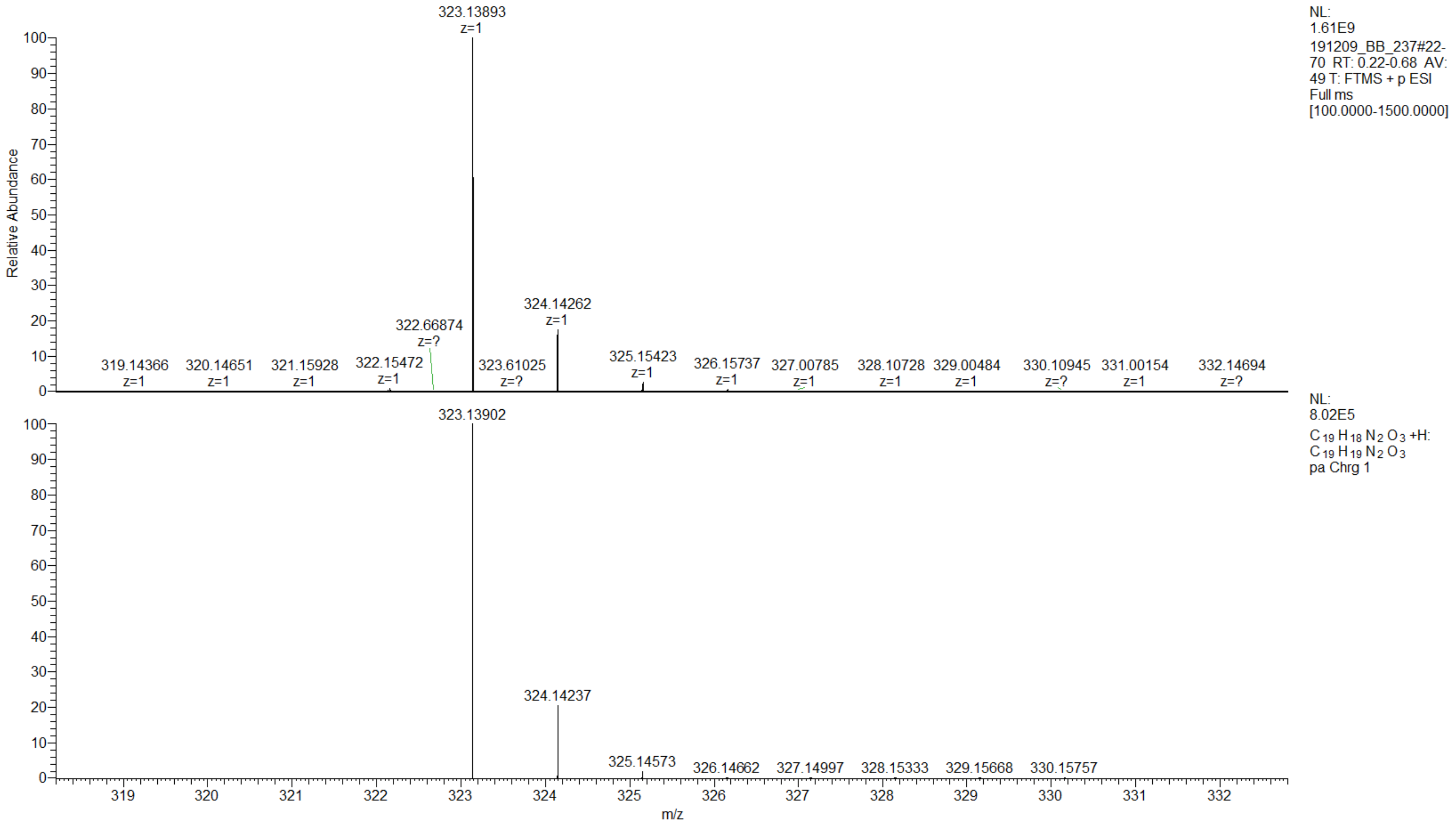

Figure S104. ESI HRMS spectrum of *N*-hydroxy-4-(6-oxodibenzo[*b*,*f*]azocin-5(6*H*)-yl)butanamide (**7i**).

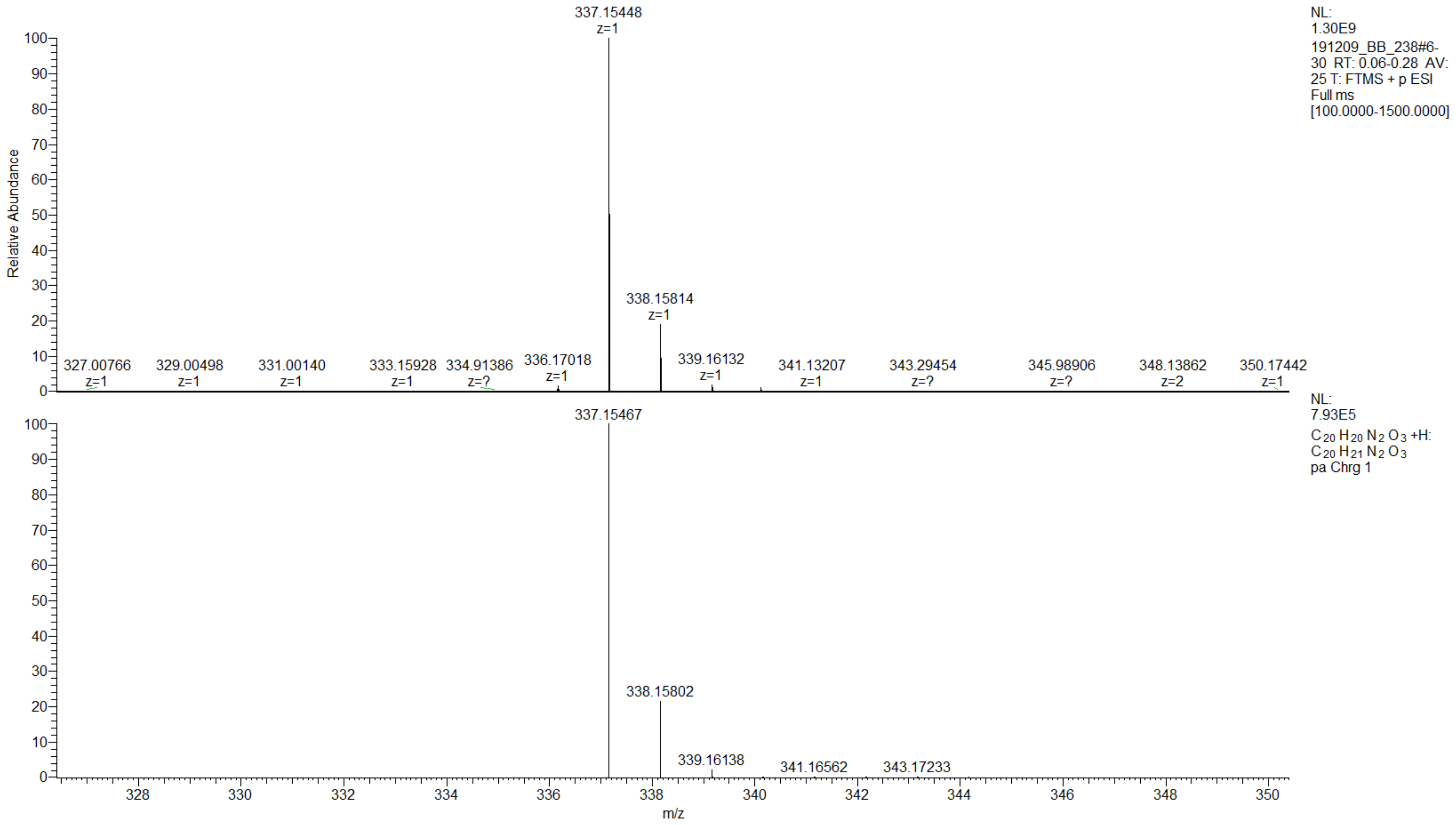

Figure S105. ESI HRMS spectrum of *N*-hydroxy-5-(6-oxodibenzo[*b*,*f*]azocin-5(6*H*)-yl)pentanamide (**7j**).

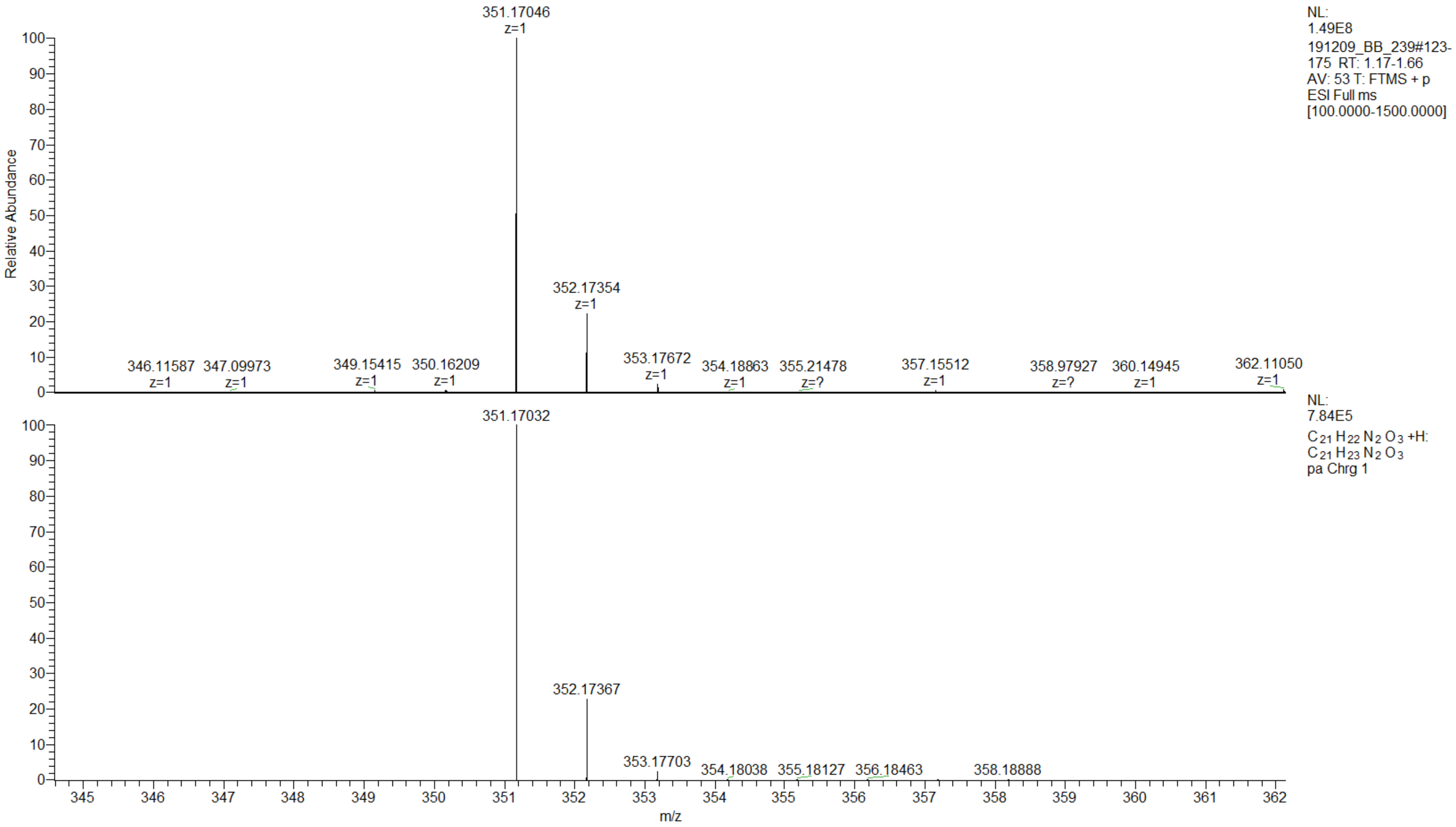

Figure S106. ESI HRMS spectrum of *N*-hydroxy-6-(6-oxodibenzo[*b*,*f*]azocin-5(6*H*)-yl)hexanamide (**7k**).

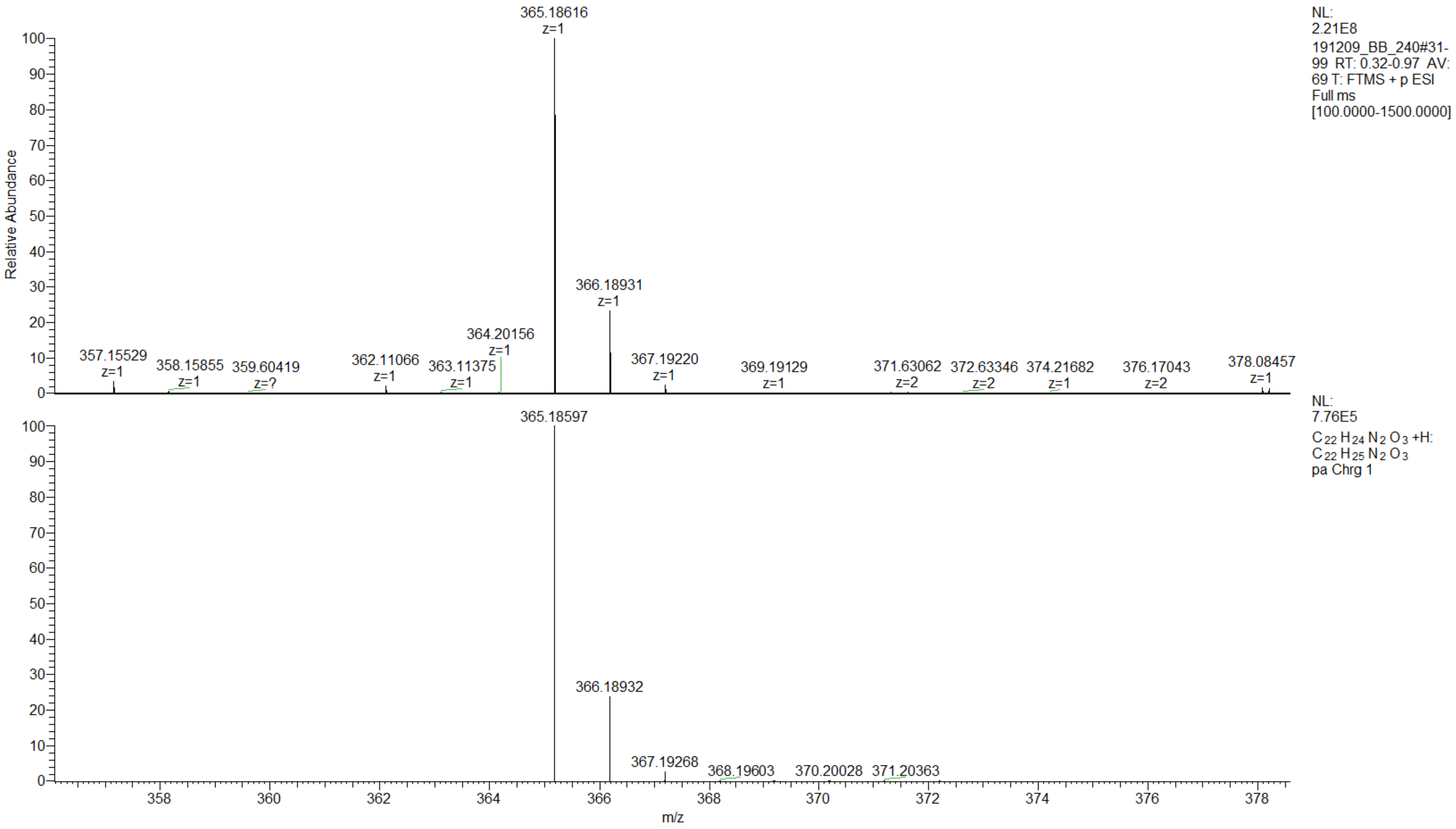

Figure S107. ESI HRMS spectrum of *N*-hydroxy-7-(6-oxodibenzo[*b*,*f*]azocin-5(6*H*)-yl)heptanamide (**7l**).

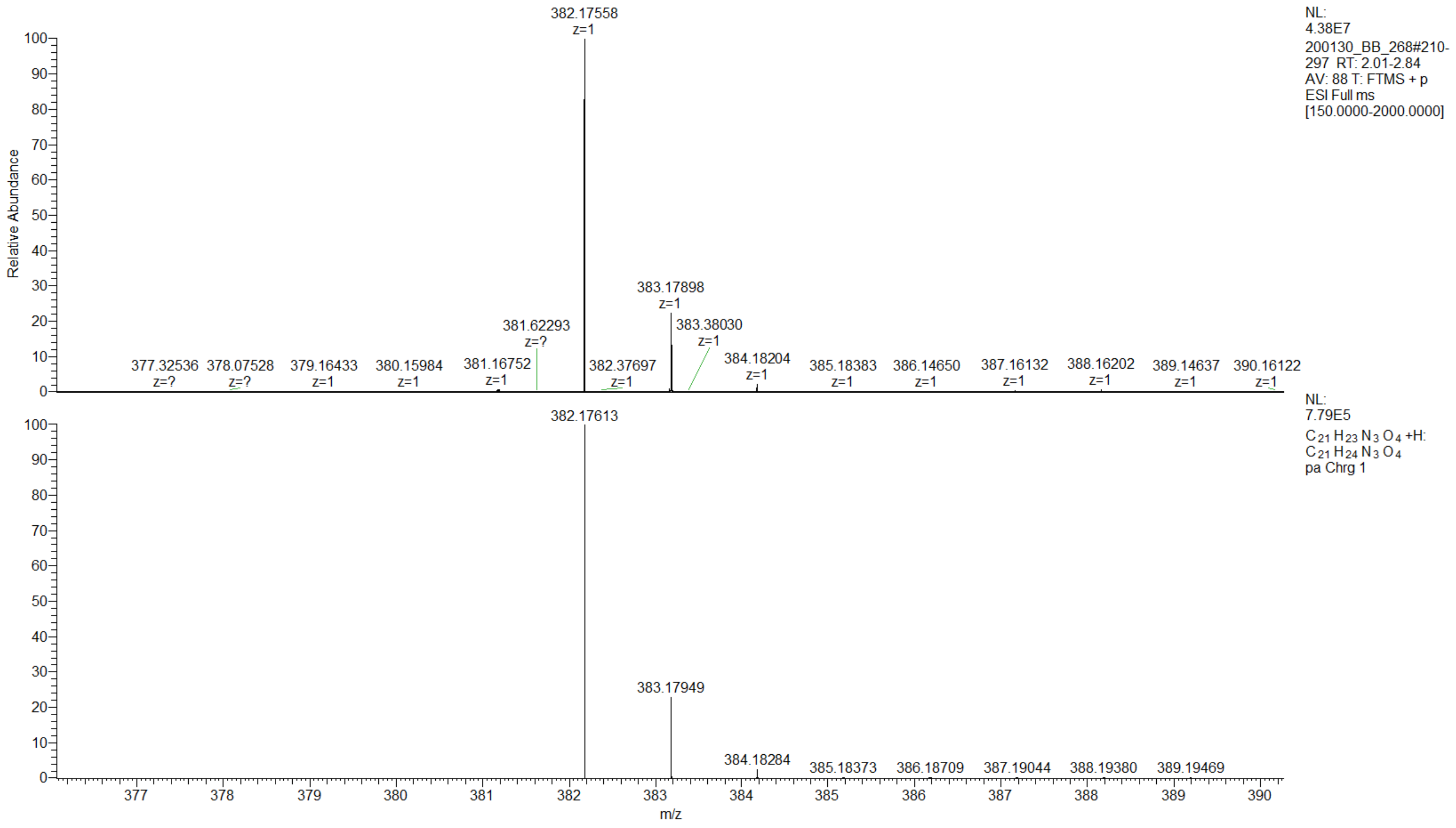

Figure S108. ESI HRMS spectrum of *N*-hydroxy-6-(12-methyl-6,11-dioxo-11,12-dihydrodibenzo[*b*,*f*][1,4]diazocin-5(6*H*)-yl)hexanamide (**7m**).

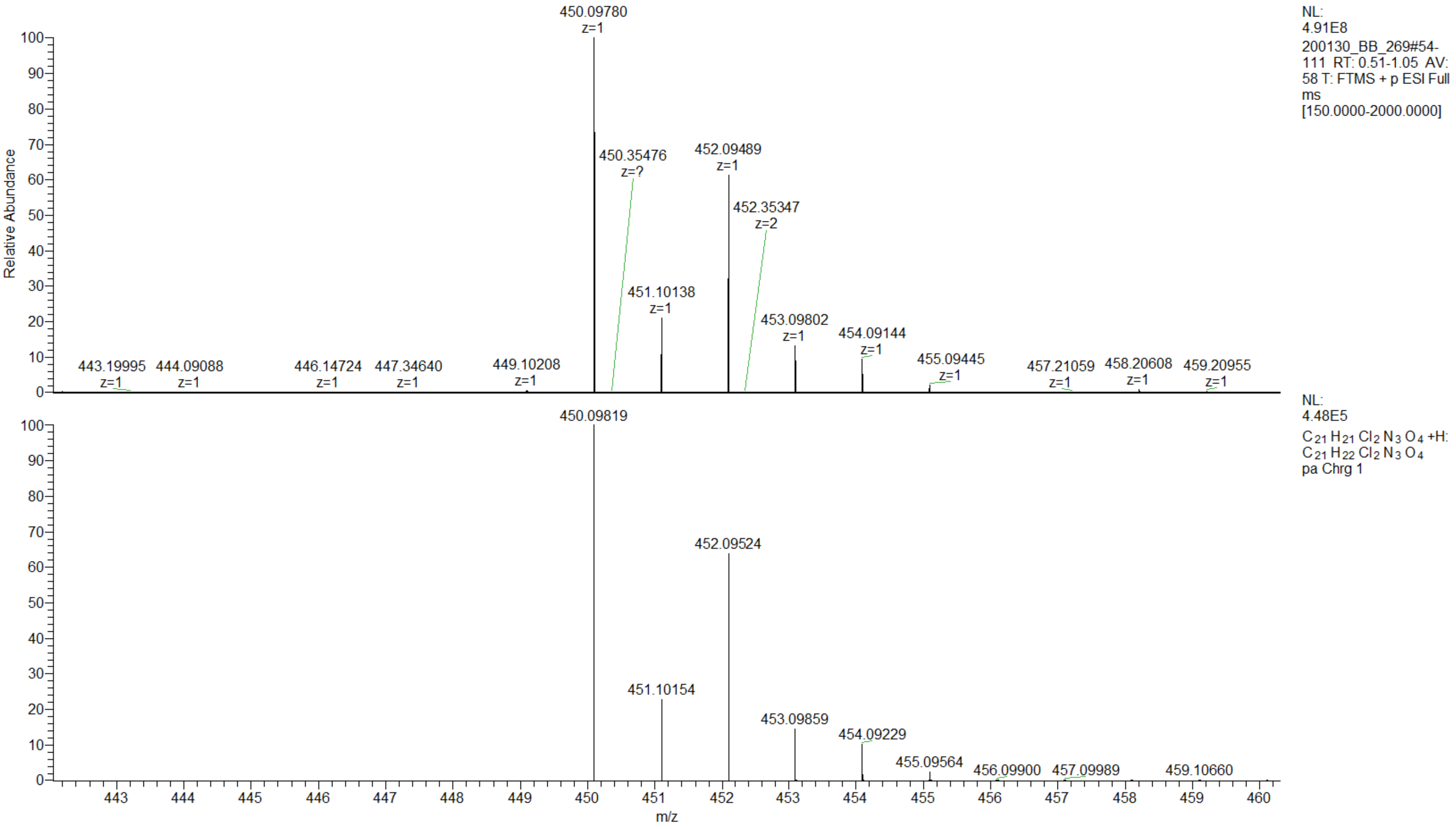

Figure S109. ESI HRMS spectrum of *N*-hydroxy-6-(8,9-dichloro-12-methyl-6,11-dioxo-11,12-dihydrodibenzo[*b*,*f*][1,4]diazocin-5(6*H*)-yl)hexanamide (**7n**).

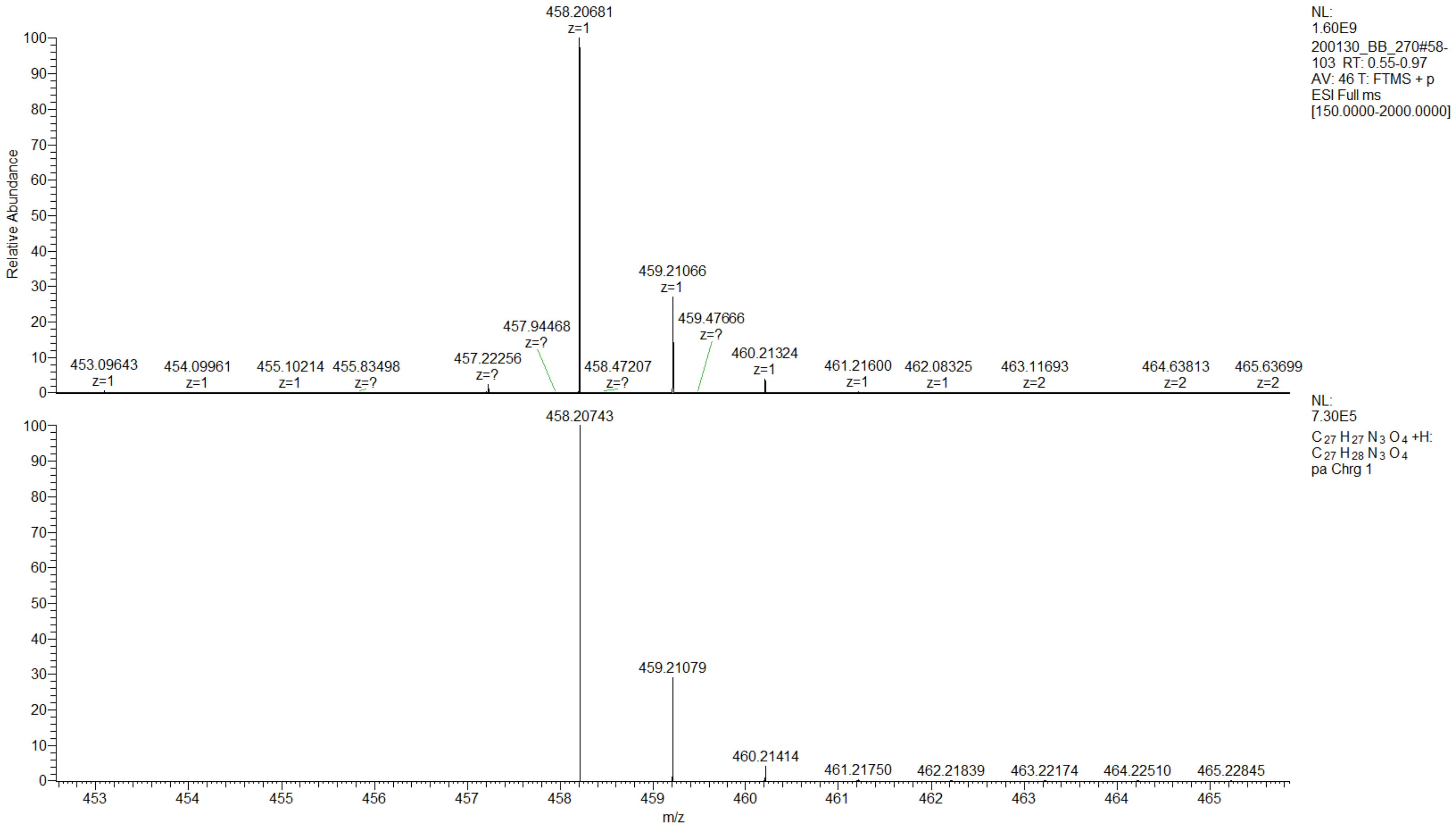

Figure S110. ESI HRMS spectrum of *N*-hydroxy-6-(12-benzyl-6,11-dioxo-11,12-dihydrodibenzo[*b*,*f*][1,4]diazocin-5(6*H*)-yl)hexanamide (**7o**).

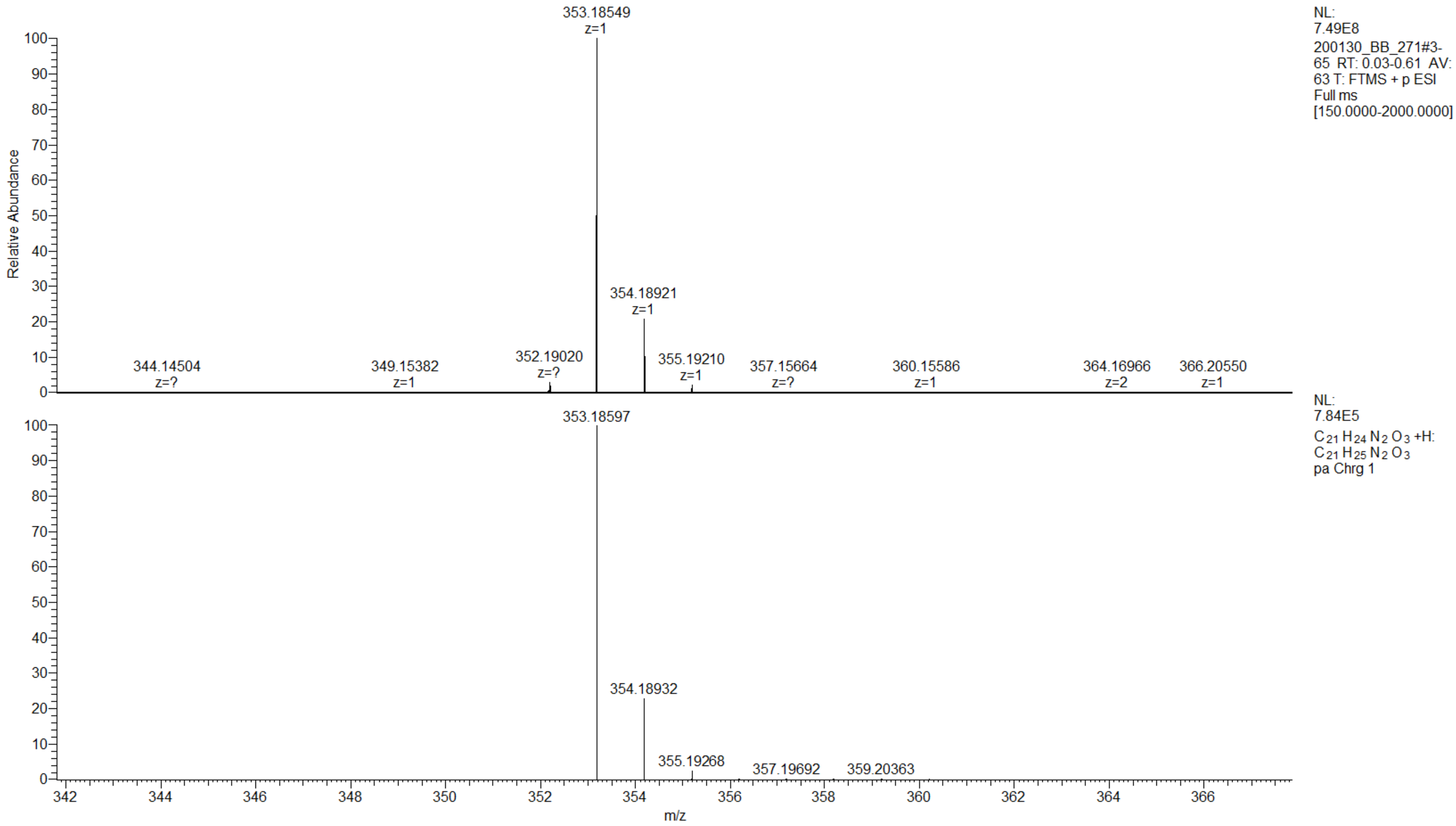

Figure S111. ESI HRMS spectrum of *N*-hydroxy-6-(6-oxo-11,12-dihydrodibenzo[*b*,*f*]azocin-5(6*H*)-yl)hexanamide (**7p**).

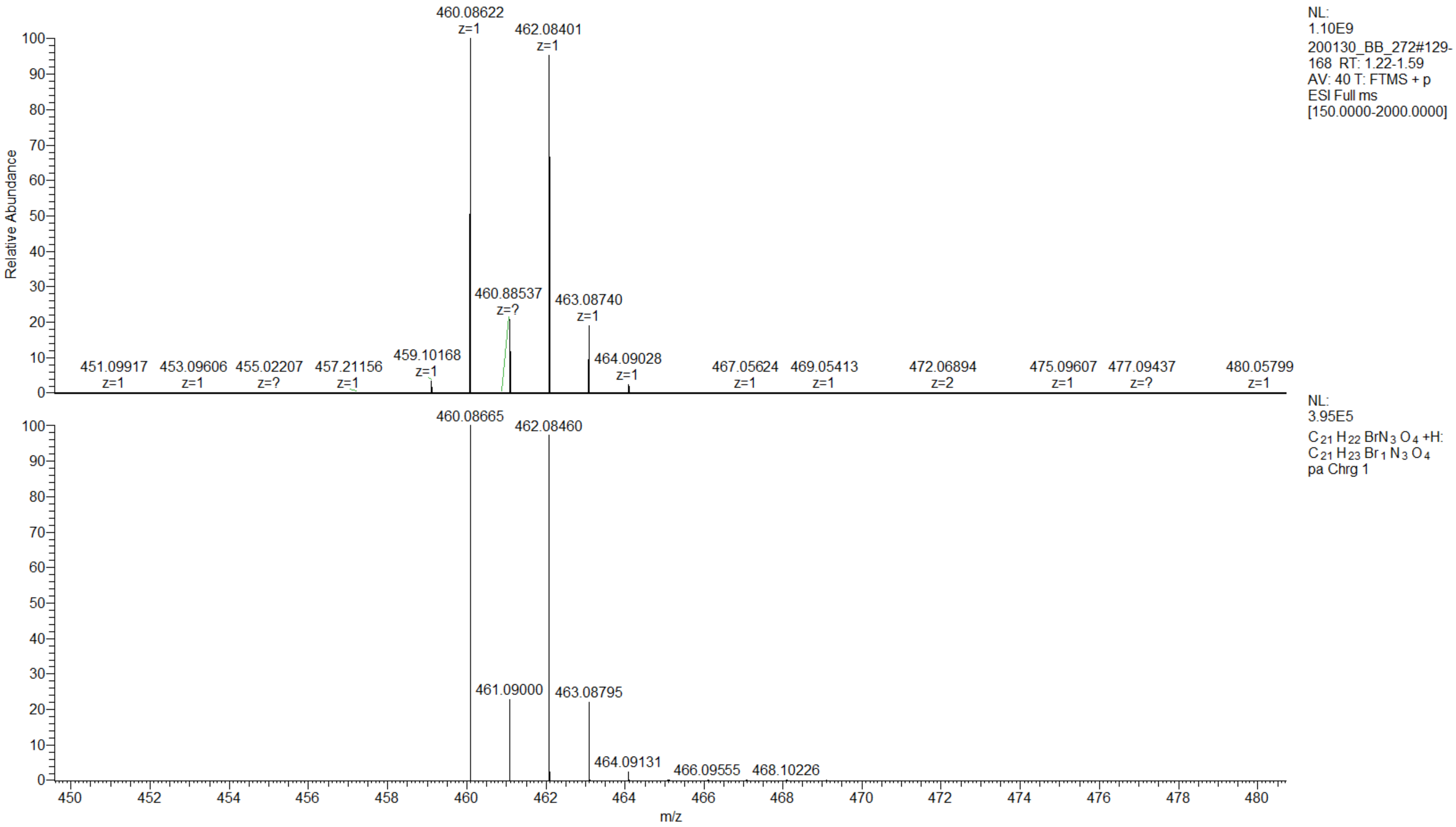

Figure S112. ESI HRMS spectrum of *N*-hydroxy-6-(2-bromo-11-methyl-6,12-dioxo-11,12-dihydrodibenzo[*b*,*f*][1,5]diazocin-5(6*H*)-yl)hexanamide (**7r**).

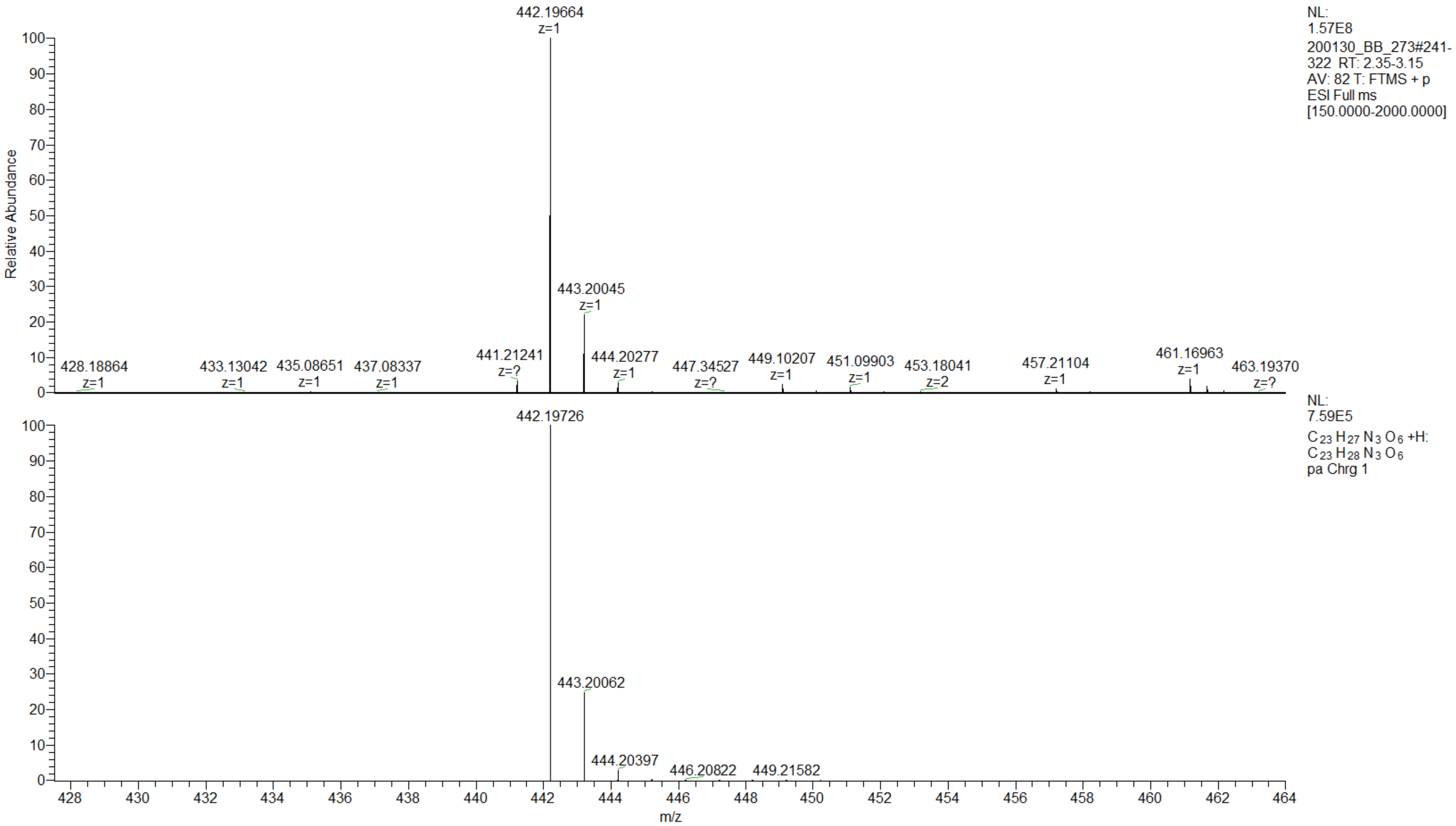

Figure S113. ESI HRMS spectrum of *N*-hydroxy-6-(2,3-dimethoxy-11-methyl-6,12-dioxo-11,12-dihydrodibenzo[*b*,*f*][1,5]diazocin-5(6*H*)-yl)hexanamide (**7s**).

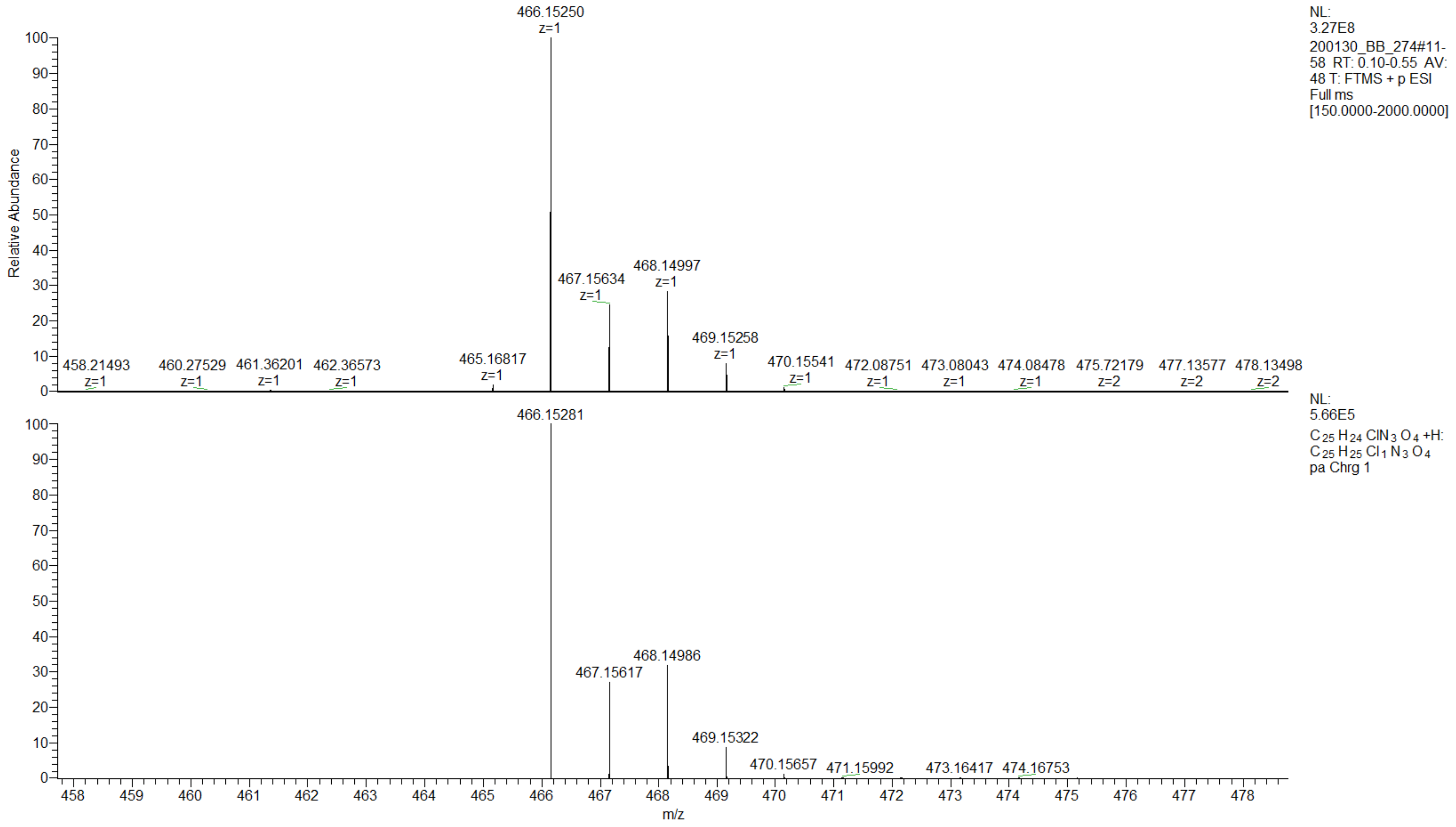

Figure S114. ESI HRMS spectrum of *N*-hydroxy-6-(5-methyl-6,14-dioxo-5,14-dihydrobenzonaphtho[2,3-*f*][1,5]diazocin-13(6*H*)-yl)hexanamide (**7t**).
